# Readability of English, German, and Russian Disease-Related Wikipedia Pages: Automated Computational Analysis

**DOI:** 10.2196/36835

**Published:** 2022-05-16

**Authors:** Jelizaveta Gordejeva, Richard Zowalla, Monika Pobiruchin, Martin Wiesner

**Affiliations:** 1 Department of Medical Informatics Heilbronn University Heilbronn Germany; 2 Consumer Health Informatics SIG German Association for Medical Informatics, Biometry & Epidemiology (GMDS e. V.) Cologne Germany; 3 Center for Machine Learning Heilbronn University Heilbronn Germany; 4 GECKO Institute for Medicine, Informatics & Economics Heilbronn University Heilbronn Germany

**Keywords:** readability, health literacy, health education, Wikipedia

## Abstract

**Background:**

Wikipedia is a popular encyclopedia for health- and disease-related information in which patients seek advice and guidance on the web. Yet, Wikipedia articles can be unsuitable as patient education materials, as investigated in previous studies that analyzed specific diseases or medical topics with a comparatively small sample size. Currently, no data are available on the average readability levels of all disease-related Wikipedia pages for the different localizations of this particular encyclopedia.

**Objective:**

This study aimed to analyze disease-related Wikipedia pages written in English, German, and Russian using well-established readability metrics for each language.

**Methods:**

Wikipedia database snapshots and Wikidata metadata were chosen as resources for data collection. Disease-related articles were retrieved separately for English, German, and Russian starting with the main concept of *Human Diseases and Disorders* (German: *Krankheit*; Russian: *Заболевания человека*). In the case of existence, the corresponding International Classification of Diseases, Tenth Revision (ICD-10), codes were retrieved for each article. Next, the raw texts were extracted and readability metrics were computed.

**Results:**

The number of articles included in this study for English, German, and Russian Wikipedia was n=6127, n=6024, and n=3314, respectively. Most disease-related articles had a Flesch Reading Ease (FRE) score <50.00, signaling difficult or very difficult educational material (English: 5937/6125, 96.93%; German: 6004/6022, 99.7%; Russian: 2647/3313, 79.9%). In total, 70% (7/10) of the analyzed articles could be assigned an ICD-10 code with certainty (English: 4235/6127, 69.12%; German: 4625/6024, 76.78%; Russian: 2316/3314, 69.89%). For articles with ICD-10 codes, the mean FRE scores were 28.69 (SD 11.00), 20.33 (SD 9.98), and 38.54 (SD 13.51) for English, German, and Russian, respectively. A total of 9 English ICD-10 chapters (11 German and 10 Russian) showed significant differences: chapter F (FRE 23.88, SD 9.95; *P*<.001), chapter E (FRE 25.14, SD 9.88; *P*<.001), chapter H (FRE 30.04, SD 10.57; *P*=.049), chapter I (FRE 30.05, SD 9.07; *P*=.04), chapter M (FRE 31.17, 11.94; *P*<.001), chapter T (FRE 32.06, SD 10.51; *P*=.001), chapter A (FRE 32.63, SD 9.25; *P*<.001), chapter B (FRE 33.24, SD 9.07; *P*<.001), and chapter S (FRE 39.02, SD 8.22; *P*<.001).

**Conclusions:**

Disease-related English, German, and Russian Wikipedia articles cannot be recommended as patient education materials because a major fraction is difficult or very difficult to read. The authors of Wikipedia pages should carefully revise existing text materials for readers with a specific interest in a disease or its associated symptoms. Special attention should be given to articles on *mental, behavioral,* and *neurodevelopmental disorders* (ICD-10 chapter F) because these articles were most difficult to read in comparison with other ICD-10 chapters. Wikipedia readers should be supported by editors providing a short and easy-to-read summary for each article.

## Introduction

### Overview

Many people consult the internet as a rapidly accessible resource to find information [1,2]. This applies to patients who want to educate themselves about a disease in a personal or family context [3-5]. Studies have shown that the internet has become a popular source of information for patients [6,7].

According to the available rankings, the web-based encyclopedia Wikipedia is a popular domain worldwide [8,9]. The web-based encyclopedia also appears among the top websites when searching for health-related information on search engines such as Google [10,11]. In December 2021, the English version of Wikipedia contained 6,423,416 articles [12]. It is a popular knowledge base that is consulted by many users to find out more about diseases and conditions, as well as for self-education purposes [13,14]. The target groups of Wikipedia are heterogeneous and include patients, students, practitioners, and the public [15].

Wikipedia articles can be written and edited by everyone, which adds to their popularity [15]. Farič et al [16] found that health-related content on Wikipedia is created by both health specialists and laypeople. Wikipedians are driven by values and beliefs, intrinsic motivation, and a certain sense of obligation [16]. Although Wikipedia is a popular resource for accessing medical knowledge [10,17], its readability is not assessed, quality assured, or controlled before publishing. This can lead to articles being difficult to read and understand [10,18,19], which can result in a lack of comprehensibility and an inability to help patients bridge the health literacy gap [20,21].

Patient education, for example, through internet searches, is an important step in medical compliance and patient empowerment [20,22,23]. Thus, it can influence the health care process and patient-physician relationship either positively or negatively [24,25]. However, to use texts as patient education materials, they must be understandable and easy to read. Readability can be defined as the number of school grades or years of formal education a person has received. In the United States, the recommended grade level for patient education materials is 7 to 8 [26].

The readability of a text can be computed using several established metrics. Several formulas are available for English texts [27-31], with adaptions for the German [32] or Russian language [33]. However, manually calculating the readability of texts is a resource- and time-consuming task.

This study assessed the readability of disease-related Wikipedia articles written in 3 different languages. Using an automated computation approach, one of our aims was to assess whether Wikipedia articles are suitable as patient education materials.

### Related Work

Several studies have analyzed the readability of health-related Wikipedia pages. Before this study, we conducted a systematic literature review to assess how readability metrics have been used to evaluate health-related Wikipedia articles. The details of the review are included in Multimedia Appendix 1 [10,11,17-19,34-46]. In total, 31 articles were closely evaluated. In most publications, the readability of texts was assessed using web-based accessible software (eg, [47]), to which texts of English Wikipedia articles were manually copied. Furthermore, only articles on certain diseases or health-related topics were analyzed (eg, anatomy [34] or pediatric ophthalmology [18] articles). In general, Wikipedia pages were difficult to read. Some selected publications related to this study are presented in the following paragraphs.

Brigo et al [35] assessed the readability of 41 Wikipedia articles on epilepsy. The selected Wikipedia articles were divided into two categories: (1) articles related to epilepsy (n=23) and (2) articles related to antiepileptic drugs (n=18). The authors found that average Flesch Reading Ease (FRE) values for these articles were 30.2 (SD 8.1) and 19.6 (SD 7.6) for epilepsy and antiepileptic drugs, respectively. Both values corresponded to texts that were difficult to read and understand. Other classic readability metrics were also calculated. On average, all analyzed Wikipedia articles “[...] correspond to a 14th academic grade level (14.3±1.7) and to 16.4±2.0 years of formal education required to easily understand the text on the first reading.”

In 2020, Suwannakhan et al [34] selected 40 *anatomy* articles from Wikipedia to analyze their readability. The assessment showed that, on average, these articles were difficult to understand and required at least a college education level (FRE: mean 42.4, SD 10.8; Flesch-Kincaid Grade Level [FKGL]: mean 12.3, SD 2.1).

In a recent study, Handler et al [36] compared the readability of Wikipedia articles on *pelvic floor disorders* with that of patient education leaflets. The authors collected 30 Wikipedia articles and 29 leaflets. They found that Wikipedia articles (Simple Measure of Gobbledygook [SMOG] 12.0) were significantly (*P*<.001) harder to read than the patient education material (SMOG 3.4). The authors also reported readability values for Wikipedia articles in different categories. The collected articles corresponded to a college-level education needed to adequately comprehend the text.

Hutchinson et al [10] investigated the readability of texts available on the web regarding *internal medical* diagnoses. In this study, Google was used to collect data. Wikipedia appeared among the top 5 resulting websites. The authors stated that texts acquired from Wikipedia had an average grade level of 14.6, which was the highest value among all sources.

Similarly, John et al [18] compared the readability of different information available on the web on *pediatric ophthalmology*. In this context, a Google search was performed, and Wikipedia, among other websites, was searched internally for relevant articles on this topic. A total of 34 articles were retrieved, including 10 (29%) from Wikipedia. The authors also found that Wikipedia was the most difficult to understand in comparison with other analyzed resources, with an average grade level of 17.4 (SD 1.18).

In 2020, Shetty et al [19] conducted a search for patient education material available on the web regarding *otitis media*. The authors then assessed the readability of 6 selected websites, including Wikipedia, with 24 patient education pages. Across all investigated resources, Wikipedia had the highest reading level (Gunning Frequency of Gobbledygook [FOG] 15.95, SMOG 14.6, FKGL 12.5, Coleman-Liau Index [CLI] 12.64, and Automated Readability Index [ARI] 11.92).

Of the 31 studies, 8 (26%) also compared the readability of Wikipedia with other available sources (eg, patient education brochures and websites) [11,37-43]. All of them reported Wikipedia to be the most difficult to read among the assessed sources.

In 2014, Kräenbring et al [44] analyzed the readability of Wikipedia articles on pharmacology written in German. In total, 100 curricular drugs were selected from the relevant textbooks. In the context of this study, only Wikipedia articles that overlapped with the information provided in the textbooks were included (n=95). The authors reported the modified FRE for the German language by Amstad [32] and the Vienna Formula (*Wiener SachTextFormel* [WSTF]) readability metrics for Wikipedia and textbooks, respectively: “[...]*R_Amstad_*: 7.1±1.7 vs. 7.4±1.8, *P*=0.9; *R_1. WSTF_*: 15.4±0.5 vs.14.5±0.2, *P*=0.07.” In their study, they found no significant difference between the readability of Wikipedia and the selected textbooks. Both sources provided information that was difficult to read and required tertiary education for an adequate understanding of the material.

### Aims of the Study

Compared with previous research in this field, this study does not focus on 1 particular medical subfield but includes all disease-related Wikipedia pages in 3 languages: English, German, and Russian [48-50]. The authors decided to focus on the Wikipedia category *Human Diseases and Disorders* (German: *Krankheit*; Russian: *Заболевания человека*) because all articles related to diseases are associated with this category.

In this context, four aims were defined: (1) to automatically collect articles from Wikipedia related to the category *Human Diseases and Disorders*, acquire the current state, and report descriptive statistics such as the number of articles, sentences, and words; (2) to categorize them into distinguishable medical subfields using the International Classification of Diseases, Tenth Revision (ICD-10) [51]; (3) to automatically evaluate the readability of retrieved articles with established readability metrics per language; and (4) to assess and compare the text difficulty among medical fields and languages.

In the context of the second aim, ICD-10 was chosen because (1) it has been widely adopted in many health care systems worldwide (“all WHO member states” [52]), and (2) respective codes are provided in many disease-related Wikipedia articles [53-55] to provide a precise reference to a stable classification system.

## Methods

### Study Design

This study comprised 2 stages. To answer the first 2 research aims, the authors separately collected articles from English, German, and Russian Wikipedia that belong to the category *Human Diseases and Disorders* (German: *Krankheit*; Russian: *Заболевания человека*). Furthermore, the data collection for each language was expanded based on the articles retrieved from the other 2 Wikipedia domains. Next, ICD-10 codes were retrieved automatically from Wikidata, a central, structured data knowledge base of related resources (ie, Wikipedia), which can be read by machines. For each language, the included articles were separated into two groups: (1) articles that were retrieved and had an ICD-10 code assigned (group A) and (2) articles without an ICD-10 code (group B).

Starting in 2020, a transition toward International Classification of Diseases, Eleventh Revision (ICD-11), [56] began [57], which is already being disseminated in the English Wikipedia edition; however, German or Russian Wikipedia authors still refer to ICD-10 in 2021. With no ICD-11 codes available for all 3 languages, the ICD-10 was chosen for stable comparisons.

Articles included in group A referred to *Human Diseases and Disorders* for which a corresponding ICD-10 code was either directly annotated or could be resolved. However, articles in group B were not excluded for several reasons. Some articles in this group referred to human diseases; however, the ICD-10 was not referred to by any of the authors of the article. Some articles were associated with a disease (eg, symptoms, root causes, and physiological processes) and did not have a specific ICD-10 code. However, those were nevertheless of importance to readers; for example, *cough* (German: *Husten*; Russian: *Кашель*) and *cytokine storm* (German: *Zytokinsturm*; Russian: *Цитокиновый шторм*).

In the subsequent stage, the authors used collected data from the first stage to perform an automated readability analysis to answer research aims 3 and 4.

### Study Setting

A total of 3 static snapshots of Wikipedia’s database, dated June 30, 2021, were used to build a category graph for *Human Diseases and Disorders* per language. Each of the 3 language-specific databases was queried to retrieve and analyze the readability of all relevant plain texts. For a detailed description of the preprocessing steps, see the *Computational Processing Steps* section.

### Readability Analysis

#### Definition

Readability [58] refers to the properties of written text with respect to readers’ competence, motivation, and understanding of a document [59]. It reflects the (1) complexity of a text’s structure, (2) sentence structure, and (3) chosen vocabulary.

#### FRE Scale

A well-known readability formula for English is the FRE metric [27]. To compute the FRE metric for a given text, the average sentence length (ASL) and average syllables per word (ASW) must be calculated. FRE relies on the observation that short words or sentences are usually easier to understand than longer words.

For this analysis, three versions of FRE were applied: (1) the original metric developed for the English language [27], (2) the modified FRE for the German language developed by Amstad [32], and (3) the modified FRE for the Russian language developed by Solovyev et al [33], as shown in the following equations:

FRE = 206.835 – (1.015 × ASL) – (84.6 × ASW)

FRE = 180 – ASL – (58.5 × ASW)

FRE = 208.7 – (2.6 × ASL) – (39.2 × ASW)

#### FKGL Metric

Another widely used readability metric for the English language is the FKGL readability test [60]. It is a modified version of the FRE and was developed to assess readability on the scale of US school grades. This formula, similar to FRE, is based on ASW and ASL.

In this study, the authors used two versions of FKGL: (1) the original FKGL metric for the English language and (2) the modified FKGL for the Russian language developed by Solovyev et al [33], as shown in the following equations:

FKGL = (0.39 × ASL) + (11.8 × ASW) – 15.59

FKGL = (0.36 × ASL) + (5.76 × ASW) – 11.97

#### The Gunning FOG Index

Gunning FOG is a measure of readability that also relies on the fact that shorter words and sentences are easier to understand. It was developed by Gunning [28] to measure the readability of English text. The formula is based on ASL and the percentage of hard words—that is, ≥3 syllables—in the text, as shown in the following equation:

Gunning FOG = 0.4 × (ASL + percentage of hard words)

#### SMOG Grade Level

Another established readability formula for the English language is SMOG. It was derived by McLaughlin [29]. It is based on the count of polysyllabic words (*p*) (ie, ≥3 syllables) in samples of 30 sentences, as shown in the following equation:




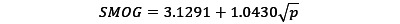




#### The ARI Metric

ARI is a readability scale derived from sentence difficulty and word difficulty. It was proposed by Senter and Smith [61]. Unlike the aforementioned metrics, word difficulty is calculated based on the character count of the word, not the syllable count, as shown in the following equation:

ARI = 4.71 × (characters / words) + 0.5 × (words / sentences) – 21.43

#### The CLI Metric

In the Coleman-Liau readability formula [30], similar to the ARI, the difficulty of a word is calculated based on the average number of letters per 100 words (*L*). In contrast, sentence difficulty is derived from the average number of sentences per 100 words (*S*), as shown in the following equation:

CLI = 0.0588 × *L* – 0.296 × *S* – 15.8

#### Vienna Formula (WSTF)

The authors applied this metric to measure the readability of German texts. In contrast to the FRE, the Vienna formula (WSTF) is not an adapted version of the German language. Instead, it relies on the work of Bamberger and Vanacek [62], who conducted an analysis based on German texts. They derived at least five versions of the Vienna formula for prose and nonfictional texts. Typically, the fourth WSTF was used for text analysis. This metric also relies on ASL and the proportion of (complex) words with ≥3 syllables (MS), as shown in the following equation:

Fourth WSTF = 0.2656 × ASL + 0.2744 × MS – 1.6939

#### Difficulty

Most metrics, apart from FRE, output school grades. This indicates the degree of education required to understand the text. For instance, a grade of 10 corresponds to an easily readable text, which is suitable for readers educated to at least 10th grade and corresponds to the age of 15 to 16 years in the US school system. The higher the grade level, the more difficult it is to understand.

The FRE metric yields values on a scale of 0 to 100; lower values indicate a text with a low level of readability that is difficult to read, whereas higher values reflect an easily readable text.

### Computational Processing Steps

#### Stage 1: Data Collection

The following subsections describe the steps that were conducted in stage 1 to build a data collection of relevant disease-related articles to be included in the study.

##### Step 1: Graph-Based Data Retrieval From Wikipedia

We used a static snapshot of the Wikipedia database for the 3 languages of interest obtained using the website [63]. Next, we constructed a graph data structure for each localization starting at the main concept of *Human Diseases and Disorders* (German: *Krankheit*, Russian: *Заболевания человека*). Graph statistics for each language (ie, the number of nodes and edges) can be found in Multimedia Appendix 2.

This graph contains typed *nodes* that correspond to the structure of Wikipedia and how different articles are interlinked among each other, which is referred to as edges. Subsequently, to filter articles that are related to the concept of interest according to Wikipedia’s categorization but are irrelevant for this study, a 4-fold filtering pipeline was used:

1. A wildcard-based category name filter to exclude full subcategories (eg, an overview list of *diseases and disorders by country* or *people with rare diseases*); see Multimedia Appendix 3
2. A given name filter to exclude articles about persons.
3. A geographical filter for articles related to specific countries, cities, or locations.
4. A stop words filter to target all articles that were not excluded by previous filters but were nevertheless not of interest for further evaluation (eg, different disease-related organizations and international disease days).

For every remaining article, the attributes *title*, *Wikipedia page ID*, and *text content* were collected for subsequent analyses.

##### Step 2: Article Retrieval From Wikidata

Article titles originating from English Wikipedia collected in *step 1* were used to retrieve corresponding articles from the other 2 Wikipedia editions (German and Russian). For this step, the authors used an open-source Java library, *Wikidata Toolkit* (version 0.12.1; Wikimedia) [64]. English titles were used to check for corresponding articles linked to German or Russian Wikipedia pages. If this was the case, the corresponding articles were retrieved and added to the respective language for data collection. Analogously, this process was conducted for the German and Russian articles collected in the first step.

These steps allowed us to balance the differences between collected articles from 3 different Wikipedia domains because of their different category structures and relations.

##### Step 3: ICD-10 Code Retrieval From Wikidata

The Wikidata resource comprises a structured set of metadata that can be found in related resources; for example, Wikipedia. This allowed the automated retrieval of corresponding ICD-10 codes for those articles for which the original authors or editors did not annotate an ICD-10 code for the Wikipedia page for a certain disease. This is provided through the *P4229* property in the Wikidata knowledge base.

Given every article from step 2, the processing software automatically checked whether an ICD-10 code was provided, and if so, it was added to the respective article in the study’s data collection.

For a subset of articles, the ICD-10 code was provided in merely 1 or 2 of 3 Wikipedia editions. In these cases, the available code was automatically added to the remaining corresponding articles, for which no ICD-10 code was found in the original Wikipedia database snapshot.

Some articles were identified as being associated with multiple ICD-10 codes from different chapters. For example, the article on *air embolism* was assigned ICD-10 codes O88.0 (obstetric air embolism) and T79.0 (air embolism [traumatic]). Therefore, each multi-associated article was allocated to all of its available ICD-10 main chapters. Each duplicate was assigned only to 1 ICD-10 main chapter. For instance, the article on *air embolism* was represented in the data collection twice: in ICD-10 chapters O and T.

At the end of the first stage, a collection of Wikipedia articles related to *Human Diseases and Disorders* with the following data was retrieved for the 3 languages: title, Wikipedia page ID, text content, and ICD-10 code in case available or resolved through Wikidata.

#### Stage 2: Data Analysis

In the second stage of the study, the collected data were analyzed.

For readability computations, the same analysis framework and related processing steps as presented in the study by Wiesner et al [65] was used.

In the context of this study, the raw texts of Wikipedia articles were used as input. Next, all readability metrics described in the *Readability Analysis* section were computed. A vocabulary analysis was not performed in this study.

The analysis was conducted on a Mac OS 10.15.7 64-bit computer with Java Runtime Environment (version 11.0.11; Oracle Corporation).

### Statistical Analysis

Data were analyzed using the statistical analysis software R (version 3.6.3; The R Foundation; February 29, 2020) on a MacOS 10.15.7 64-bit computer. The R package *ggplot2* [66] was used for visualization.

Several test scenarios were identified: (1) testing the readability values of 1 language pairwise against the other 2 languages to investigate whether there are significant differences in readability between languages, (2) testing the readability values of each ICD-10 chapter against the mean of all the articles per language, and (3) testing the readability values of each ICD-10 chapter against the recommended readability level of 7 (in the US grade scheme) for patient education material (only for English articles).

For the first and second scenarios, an unpaired 2-tailed *t* test was performed with the following test hypotheses, as shown in the following equations:

H_0_: µ_1_=µ_2_

H_a_: µ_1_≠µ_2_

For the third scenario, an unpaired 1-tailed *t* test was performed with the hypothesis that articles from all ICD-10 chapters in English (µ_1_) have significantly lower readability and thus a higher grade level than the recommended grade level (µ_2_), as shown in the following equations:

H_0_: µ_1_≤µ_2_

H_a_: µ_1_>µ_2_

For all 3 scenarios, a significance level of α=.05 was chosen. For the first scenario, *P* values were Holm adjusted [67] because multiple *t* tests were conducted with the same sample.

ICD-10 chapters were only included in the comparative analyses if the sample size was >25. This restriction was applied to ensure the requirements of the *t* test.

For statistical tests, the values of FRE were used because this was the only readability metric that could be computed for all languages with a compatible scale. This allowed for a comparison of text readability of different languages.

### Ethics Approval

This study does not include any studies with human participants performed by any of the authors. For this reason, no formal ethics approval was obtained for this study.

## Results

### Main Results

Wikipedia article selection and readability analysis were conducted on November 25, 2021 (German), November 30, 2021 (English), and December 3, 2021 (Russian).

**Figure 1:**
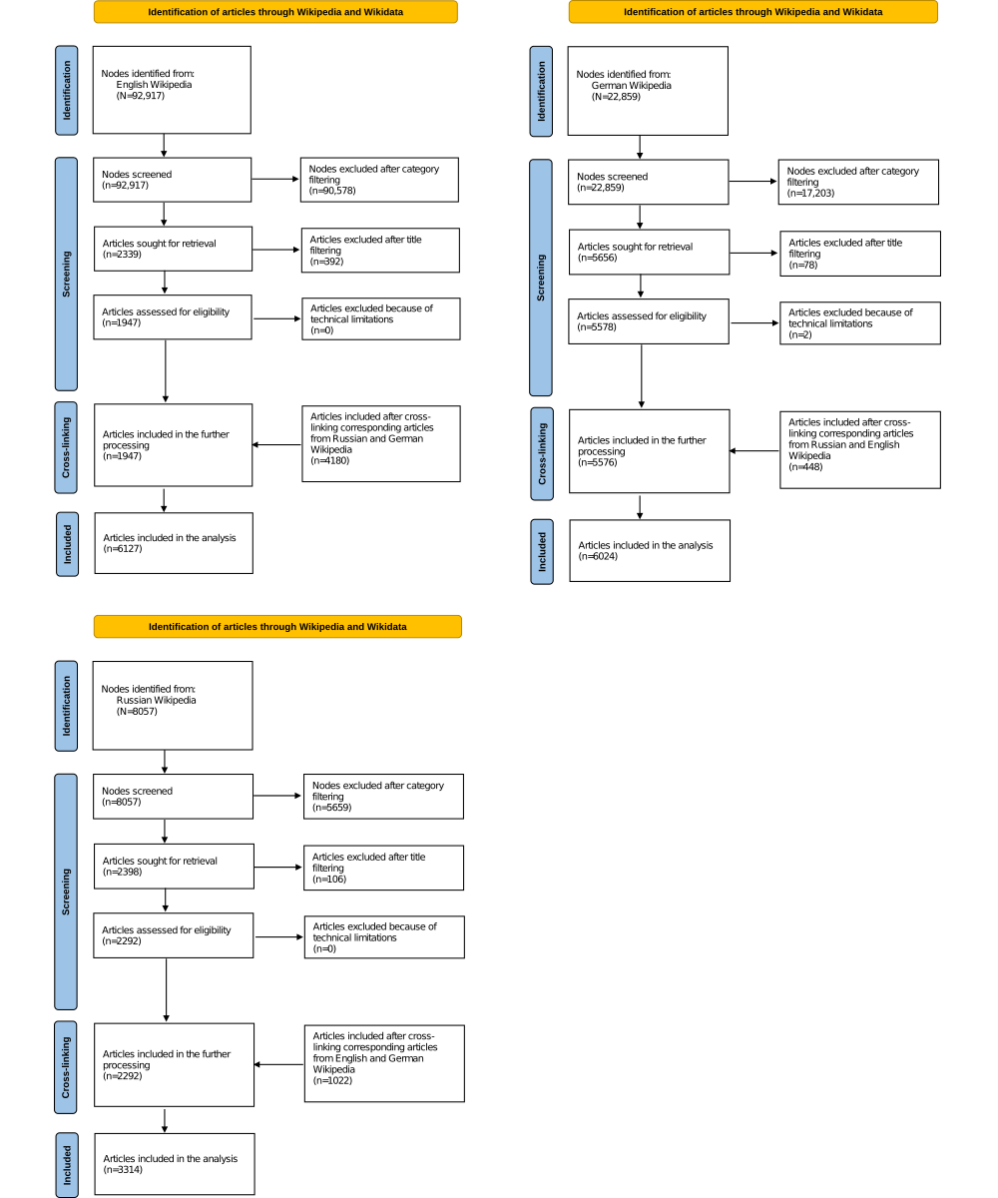
Flow diagrams for the process of data collection for English, German, and Russian.

After the application of filters, 1947 articles were collected for English, 5576 for German, and 2292 for Russian Wikipedia. These titles were used as input for *step 2* of the data collection (see the *Methods* section). In total, the number of articles included for further readability analysis for English, German, and Russian Wikipedia was n=6127, n=6024, and n=3314, respectively. A detailed summary per language is depicted in Figure 1.

### Sample Characteristics

The retrieved articles were categorized based on their ICD-10 codes into two groups: A and B.

The distribution of articles for each language in these groups is presented in Table 1.

On average, articles from English Wikipedia were the longest with regard to the number of sentences per article (group A: mean 68.49, SD 72.96; group B: mean 52.28, SD 62.02) and the number of words per article (group A: mean 1465.40, SD 1622.97; group B: mean 1213.17, SD 1497.12). Russian Wikipedia articles had the highest number of complex words per article (group A: mean 514.52, SD 734.08; group B: mean 353.89, SD 483.40).

Detailed statistics on the number of sentences, words, and complex words per language can be found in Table 2.

**Table 1:** Distribution of collected articles in groups A and B for English, German, and Russian languages.

Language	Total, N	Group A, n (%)	Group B, n (%)
English	6127	4235 (69.12)	1892 (30.88)
German	6024	4625 (76.78)	1399 (23.22)
Russian	3314	2316 (69.89)	998 (30.11)

**Table 2:** Range and mean (SD) values for the number of sentences, words, and complex words in groups A and B for English, German, and Russian languages.

Number of words and sentences and statistics	English, mean (SD; range)		German, mean (SD; range)		Russian, mean (SD; range)	
	Group A	Group B	Group A	Group B	Group A	Group B
Number of sentences	68.49 (72.96; 1-707)	52.28 (62.02; 1-466)	41.73 (61.56; 1-1032)	32.56 (51.15; 1-653)	49.70 (65.95; 2-826)	33.48 (44.63; 2-662)
Number of words	1465.40 (1622.97; 18-14,858)	1213.17 (1497.12; 15-11,987)	708.55 (1180.88; 16-21,531)	582.74 (1004.05; 18-11,515)	837.31 (1233.99; 31-16,352)	589.92 (815.02; 21-8790)
Number of complex words	362.77 (400.89; 3-3694)	299.16 (377.56; 4-3198)	274.91 (441.03; 9-6895)	216.65 (371.51; 4-4597)	514.52 (734.08; 20-9317)	353.89 (483.40; 15-5412)

### Readability Analysis

#### Overview

Readability analysis was performed for every article collected in stage 1 of this study. However, 5 articles in total (English: n=2, 40%; German: n=2, 40%; and Russian: n=1, 20%) were excluded from the analysis because of technical inability to compute the readability metrics for these articles.

Overall, 693 (English: n=212, 30.6%; German: n=390, 56.3%; and Russian: n=91, 13.1%) articles were identified as being associated with multiple ICD-10 codes from different chapters. Consequently, in the following subsections, the reported number of articles in different ICD-10 chapters and groups can differ from the data reported in Table 1.

The distribution of article difficulty for groups A and B, as well as per ICD-10 chapter, can be found in Multimedia Appendices 4-6. Box plots depicting value differences between groups A and B, as well as among ICD-10 chapters, can be found in Multimedia Appendices 7-9 for each computed readability metric.

#### English Wikipedia

All included articles from English Wikipedia were analyzed according to the readability metrics FRE, FKGL, SMOG, ARI, CLI, and Gunning FOG.

The highest number of articles was assigned to ICD-10 chapter Q (740/4471, 16.55%). The lowest number of articles was associated with chapter W (4/4471, 0.09%). In the context of a low number of articles per ICD-10 chapter, the respective chapters were excluded from the comparative analysis (see *Statistical Analysis* subsection).

The average number of sentences per article varied from 42.42 (ICD-10 chapter Q, SD 45.45) to 110.43 (ICD-10 chapter B, SD 97.90); the average number of words varied from 888.76 (ICD-10 chapter Q, SD 984.97) to 2346.28 (ICD-10 chapter F, SD 2216.12).

Chapter F had the highest grade level scores (FKGL 15.33, SD 1.53; ARI 13.87, SD 0.51; CLI 15.49, SD 0.85; SMOG 17.22, SD 1.38; and Gunning FOG 16.92. SD 0.42) and the lowest FRE score of 23.88 (SD 9.95) in comparison with other ICD-10 chapters, which indicates difficulty in reading texts.

The articles that were relatively easy to read were in chapter S with an FRE score of 39.02 (SD 8.22), and all grade level indices were <16 (FKGL 12.78, SD 1.58; ARI 13.22, SD 1.03; CLI 14.06, SD 1.15; SMOG 14.66, SD 1.34; and Gunning FOG 15.86, SD 1.29).

**Figure 2:**
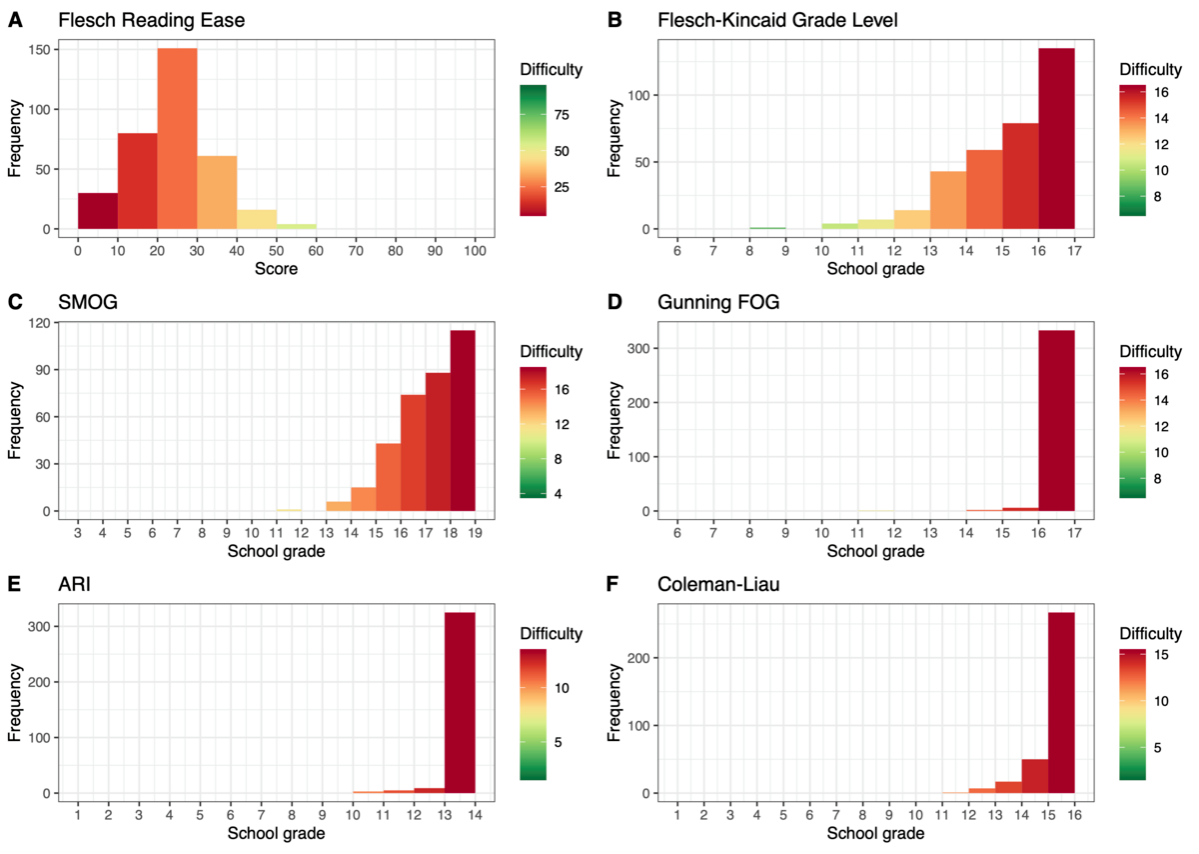
Distribution of values of all computed readability metrics (English) for articles from the International Classification of Diseases, Tenth Revision, chapter F (ICD-F). ARI: Automated Readability Index; FOG: Frequency of Gobbledygook; SMOG: Simple Measure of Gobbledygook.

**Figure 3:**
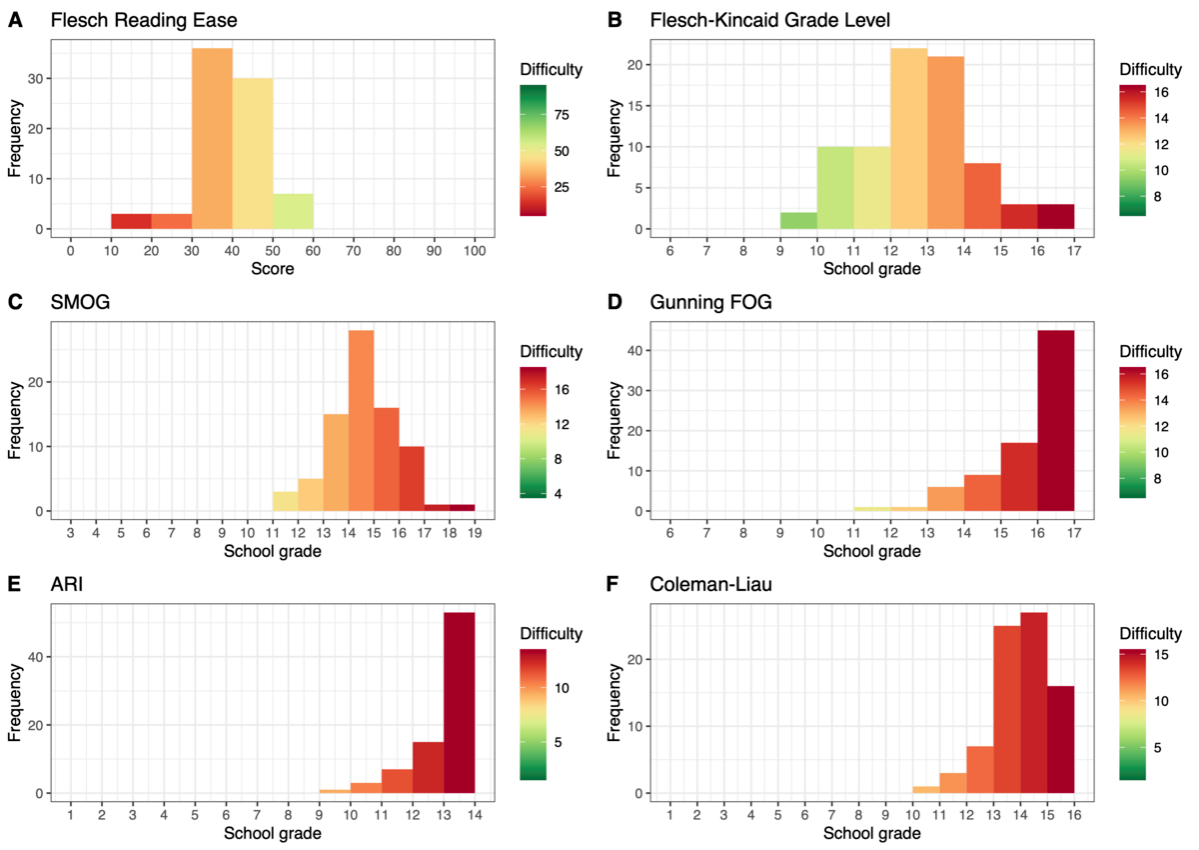
Distribution of values of all computed readability metrics (English) for articles from the International Classification of Diseases, Tenth Revision, chapter S (ICD-S). ARI: Automated Readability Index; FOG: Frequency of Gobbledygook; SMOG: Simple Measure of Gobbledygook.

Figures 2 and 3 depict the distribution of readability values for each article in chapters F and S, respectively. Each subfigure represents the values computed using different metrics for the same sample. In total, 76.3% (261/342) of the articles in chapter F had an FRE value <30.00; that is, they were very or extremely difficult to read and required a tertiary degree of education for adequate comprehension of the text. In chapter S, only 8% (6/79) of the articles had an FRE score <30.00, whereas 84% (66/79) of the articles had an FRE value between 30.00 and 50.00.

#### German Wikipedia

All included articles from German Wikipedia were analyzed according to the readability metrics FRE and WSTF.

The highest number of articles was assigned to ICD chapter Q (1030/5092, 20.23%). The lowest number of articles were in chapter X (3/5092, 0.06%). The lowest average number of sentences per article was in chapter Q (26.28, SD 27.45) and the highest was in chapter B (74.04, SD 94.26). The average word count varied from 398.84 (ICD-10 chapter Q, SD 526.23) to 1337.13 (ICD-10 chapter F, SD 1880.59).

For the FRE metric, the most difficult to read was chapter E, with a score of 17.43 (SD 9.80), and the one that was relatively easy to read was chapter S (24.60, SD 8.22). The highest WSTF value was observed in chapter I (13.62, SD 1.13) and the lowest was in chapter B (13.01, SD 1.24).

**Figure 4:**
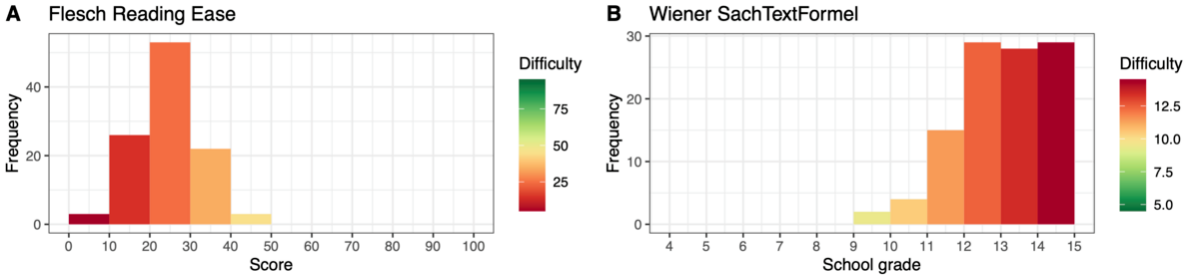
Distribution of Flesch Reading Ease and Fourth Vienna Formula values for German articles from the International Classification of Diseases, Tenth Revision, chapter S (ICD-S).

**Figure 5:**
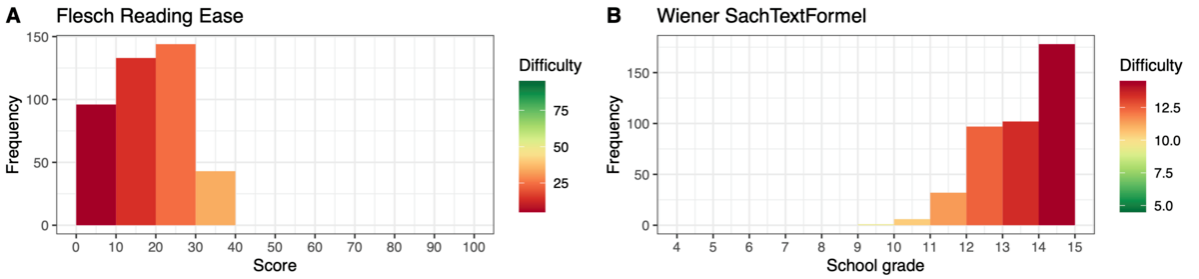
Distribution of Flesch Reading Ease and Fourth Vienna Formula values for German articles from the International Classification of Diseases, Tenth Revision, chapter E (ICD-E).

Figures 4 and 5 depict the distributions of the FRE and WSTF values for each article in chapters S and E, respectively. In chapter E, 89.7% (373/416) of the articles were very and extremely difficult to read (FRE<30.00) compared with 76.7% (82/107) of the articles in chapter S, which had an FRE score <30.00.

#### Russian Wikipedia

All included articles from Russian Wikipedia were analyzed according to the readability metrics FRE and FKGL.

The highest number of articles was assigned to ICD chapter F (275/2417, 11.38%); the lowest numbers were found for chapters W and Y (each 1/2417, 0.04%). The average sentence count per article varied from 30.42 (chapter Q, SD 32.05) to 80.60 (chapter A, SD 80.93); the average word count distribution was 489.13 (SD 571.41) to 1289.28 (SD 1468.39) in chapters Q and B, respectively.

Chapter E was, on average, the most difficult to understand (FRE 33.66, SD 12.73, and FKGL 13.35, SD 1.73), whereas the easiest to read articles were in chapter O, with an FRE score of 44.06 (SD 10.73) and an FKGL of 11.88 (SD 1.53), which were the highest average values for these metrics per chapter among all analyzed languages.

**Figure 6:**
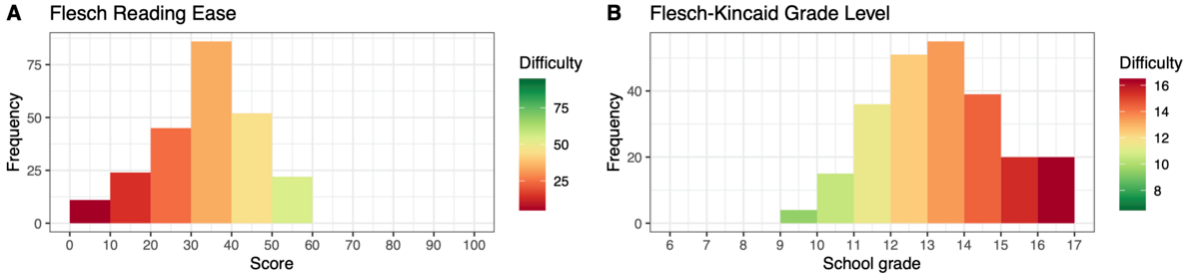
Distribution of Flesch Reading Ease and Flesch-Kincaid Grade Level values for Russian articles from the International Classification of Diseases, Tenth Revision, chapter E (ICD-E).

**Figure 7:**
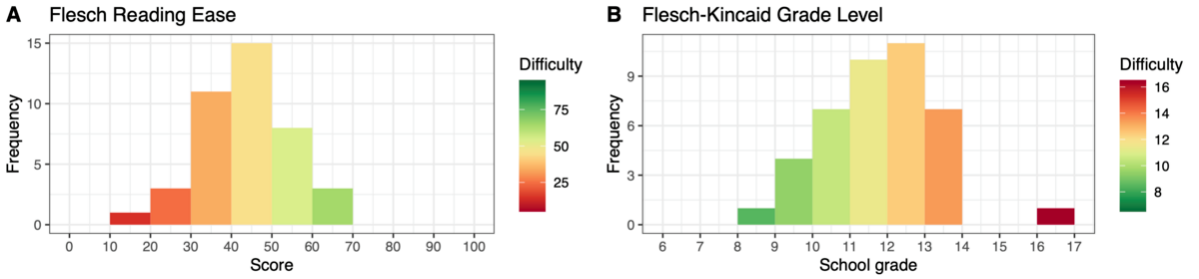
Distribution of Flesch Reading Ease and Flesch-Kincaid Grade Level values for Russian articles from the International Classification of Diseases, Tenth Revision, chapter O (ICD-O).

Figures 6 and 7 depict the distributions of the FRE and FKGL values for each article in chapters E and O, respectively. In total, 33.4% (80/240) of the articles in chapter E had an FRE value <30.00 (very or extremely difficult to read), whereas 57.5% (138/240) scored on a scale between 30.00 and 50.00 (difficult to read). A value >50.00 was observed for 9.2% (22/240) of the chapter E articles.

In chapter O, most articles (36/41, 8%) were difficult to read and required a college degree for comprehension. In total, 27% (11/41) had an FRE value >50.00, whereas 10% (4/41) had a value <30.00.

The following tables report the mean values and SDs of the readability metrics for each locale. The mean was calculated for groups A and B and every ICD-10 chapter (Tables 3-8). The tables also show the number of articles included in each category.

**Table 3:** Mean and SD of each readability metric for groups A and B for articles from English Wikipedia (N=6127).

Groups	Values, n (%)	Flesch Reading Ease, mean (SD)	Flesch-Kincaid Grade Level, mean (SD)	Automated Readability Index, mean (SD)	Coleman-Liau Index, mean (SD)	Simple Measure of Gobbledygook, mean (SD)	Gunning Frequency of Gobbledygook, mean (SD)
A^a^	4233 (69.11)	28.69 (11.00)	14.26 (1.80)	13.65 (0.85)	15.16 (1.08)	16.12 (1.53)	16.70 (0.78)
B	1892 (30.89)	29.18 (12.86)	14.47 (2.11)	13.57 (1.10)	14.87 (1.38)	16.33 (1.84)	16.61 (0.99)

^a^In group A, 2 articles were excluded from the analysis because of technical inability to compute the readability metrics.

**Table 4:** Mean and SD of each readability metric for each International Classification of Diseases, Tenth Revision, chapter individually for articles from English Wikipedia (N=4471).

International Classification of Diseases chapters	Values, n (%)	Flesch Reading Ease, mean (SD)	Flesch-Kincaid Grade Level, mean (SD)	Automated Readability Index, mean (SD)	Coleman-Liau Index, mean (SD)	Simple Measure of Gobbledygook, mean (SD)	Gunning Frequency of Gobbledygook, mean (SD)
A	107 (2.39)	32.63 (9.25)	13.69 (1.59)	13.67 (0.70)	15.04 (0.87)	15.71 (1.40)	16.60 (1.00)
B	95 (2.12)	33.24 (9.07)	13.53 (1.51)	13.55 (0.83)	14.80 (1.00)	15.60 (1.24)	16.64 (0.76)
C	216 (4.83)	29.91 (9.80)	14.03 (1.69)	13.66 (0.86)	15.20 (1.05)	15.80 (1.43)	16.66 (0.80)
D	281 (6.28)	27.67 (11.72)	14.28 (1.86)	13.58 (0.98)	15.17 (1.11)	16.07 (1.61)	16.65 (0.88)
E	414 (9.26)	25.14 (9.88)	14.81 (1.53)	13.83 (0.61)	15.48 (0.78)	16.44 (1.37)	16.87 (0.50)
F	342 (7.65)	23.88 (9.95)	15.33 (1.53)	13.87 (0.51)	15.49 (0.85)	17.22 (1.38)	16.92 (0.42)
G	324 (7.25)	27.90 (10.02)	14.57 (1.64)	13.78 (0.67)	15.21 (0.97)	16.37 (1.38)	16.80 (0.56)
H	239 (5.35)	30.04 (10.57)	13.93 (1.85)	13.48 (1.02)	14.99 (1.14)	15.86 (1.54)	16.64 (0.79)
I	183 (4.09)	30.05 (9.07)	14.29 (1.61)	13.77 (0.68)	15.25 (1.04)	16.20 (1.30)	16.79 (0.62)
J	118 (2.64)	29.79 (11.03)	13.94 (1.93)	13.50 (0.92)	15.22 (1.01)	15.75 (1.52)	16.52 (0.87)
K	200 (4.47)	27.48 (10.08)	14.37 (1.60)	13.76 (0.78)	15.37 (0.96)	16.20 (1.34)	16.79 (0.63)
L	192 (4.29)	29.38 (13.61)	13.79 (2.04)	13.40 (1.03)	15.04 (1.30)	15.85 (1.67)	16.54 (0.99)
M	258 (5.77)	31.17 (11.94)	13.67 (1.91)	13.44 (1.21)	15.02 (1.31)	15.59 (1.51)	16.52 (1.02)
N	158 (3.53)	30.11 (9.36)	13.93 (1.73)	13.61 (0.82)	15.31 (0.94)	15.79 (1.48)	16.61 (0.85)
O	85 (1.9)	30.21 (9.99)	14.24 (1.54)	13.72 (0.61)	14.98 (1.11)	16.36 (1.33)	16.82 (0.51)
P	73 (1.63)	30.22 (10.30)	14.30 (1.62)	13.75 (0.83)	14.95 (1.31)	16.27 (1.26)	16.79 (0.56)
Q	740 (16.55)	28.05 (11.95)	14.24 (1.83)	13.60 (0.86)	15.10 (1.10)	16.10 (1.57)	16.70 (0.76)
R	228 (5.1)	28.73 (10.54)	14.41 (1.74)	13.73 (0.72)	15.18 (1.00)	16.21 (1.45)	16.75 (0.72)
S	79 (1.77)	39.02 (8.22)	12.78 (1.58)	13.22 (1.03)	14.06 (1.15)	14.66 (1.34)	15.86 (1.29)
T	111 (2.48)	32.06 (10.51)	14.06 (1.86)	13.62 (0.85)	14.78 (1.12)	15.98 (1.51)	16.62 (0.94)
U	5 (0.11)	25.66 (5.18)	15.77 (0.77)	14.00 (0.00)	15.57 (0.48)	17.37 (0.66)	17.00 (0.00)
W	4 (0.09)	43.50 (9.89)	12.57 (1.26)	12.87 (0.80)	12.81 (2.62)	14.26 (1.38)	15.58 (1.05)
X	5 (0.11)	29.60 (8.49)	14.50 (1.45)	13.77 (0.51)	15.17 (1.12)	15.87 (1.10)	16.67 (0.75)
Y	5 (0.11)	28.41 (8.92)	15.01 (1.66)	14.00 (0.00)	15.20 (0.89)	16.73 (1.37)	16.89 (0.24)
Z	9 (0.2)	28.90 (7.43)	14.92 (1.14)	14.00 (0.00)	15.39 (0.99)	16.56 (0.87)	17.00 (0.00)

**Table 5:** Mean and SD of each readability metric for groups A and B for articles from German Wikipedia (N=6024).

Groups	Values, n (%)	Flesch Reading Ease, mean (SD)	Fourth Vienna Formula, mean (SD)
A	4625 (76.8)	20.33 (9.98)	13.43 (1.28)
B^a^	1397 (23.2)	23.91 (11.49)	13.05 (1.52)

^a^In group B, 2 articles were excluded from the analysis because of technical inability to compute the readability metrics.

**Table 6:** Mean and SD of each readability metric for each International Classification of Diseases, Tenth Revision, chapter individually for articles from German Wikipedia (N=5092).

International Classification of Diseases chapters	Values, n (%)	Flesch Reading Ease, mean (SD)	Fourth Vienna Formula, mean (SD)
A	112 (2.2)	22.91 (8.77)	13.10 (1.22)
B	99 (1.94)	23.71 (8.14)	13.01 (1.24)
C	227 (4.46)	19.83 (9.26)	13.45 (1.30)
D	311 (6.11)	18.54 (10.22)	13.52 (1.28)
E	416 (8.17)	17.43 (9.80)	13.59 (1.17)
F	312 (6.13)	23.10 (9.50)	13.38 (1.34)
G	408 (8.01)	20.07 (10.51)	13.43 (1.29)
H	282 (5.54)	21.91 (10.06)	13.23 (1.33)
I	202 (3.97)	18.60 (8.91)	13.62 (1.13)
J	118 (2.32)	19.90 (9.39)	13.53 (1.32)
K	224 (4.4)	21.08 (9.95)	13.27 (1.31)
L	206 (4.05)	21.25 (10.15)	13.30 (1.31)
M	297 (5.83)	19.80 (9.30)	13.48 (1.16)
N	173 (3.4)	18.70 (9.09)	13.56 (1.23)
O	74 (1.45)	22.61 (9.94)	13.37 (1.23)
P	65 (1.28)	19.79 (8.59)	13.60 (1.16)
Q	1030 (20.23)	19.25 (10.60)	13.54 (1.29)
R	268 (5.26)	20.37 (9.54)	13.61 (1.26)
S	107 (2.1)	24.60 (8.22)	13.06 (1.30)
T	130 (2.55)	23.27 (9.55)	13.31 (1.24)
U	6 (0.12)	20.76 (6.51)	13.93 (1.11)
W	5 (0.1)	24.97 (7.90)	13.34 (1.30)
X	3 (0.06)	26.47 (5.86)	12.83 (0.83)
Y	5 (0.1)	23.01 (14.15)	13.42 (2.16)
Z	12 (0.24)	17.15 (9.97)	13.76 (1.10)

**Table 7:** Mean and SD of each readability metric for groups A and B for articles from Russian Wikipedia (N=3314).

Groups	Values, n (%)	Flesch Reading Ease, mean (SD)	Flesch-Kincaid Grade Level, mean (SD)
A	2316 (69.91)	38.54 (13.51)	12.64 (1.84)
B^a^	997 (30.09)	38.82 (15.34)	12.58 (2.10)

^a^In group B, 1 article was excluded from the analysis because of technical inability to compute the readability metrics.

**Table 8:** Mean and SD of each readability metric for each International Classification of Diseases, Tenth Revision, chapter individually for articles from Russian Wikipedia (N=2417).

International Classification of Diseases chapters	Values, n (%)	Flesch Reading Ease, mean (SD)	Flesch-Kincaid Grade Level, mean (SD)
A	83 (3.43)	42.03 (11.38)	12.16 (1.50)
B	78 (3.23)	42.22 (9.22)	12.16 (1.32)
C	105 (4.34)	36.10 (13.01)	12.86 (1.75)
D	102 (4.22)	36.07 (13.58)	13.01 (1.83)
E	240 (9.93)	33.66 (12.73)	13.35 (1.73)
F	275 (11.38)	35.68 (14.78)	13.02 (2.02)
G	146 (6.04)	37.43 (12.76)	12.80 (1.72)
H	186 (7.7)	41.43 (13.59)	12.23 (1.85)
I	117 (4.84)	35.55 (13.69)	13.04 (1.76)
J	75 (3.1)	37.99 (12.26)	12.73 (1.69)
K	115 (4.76)	39.95 (12.03)	12.47 (1.68)
L	88 (3.64)	41.99 (12.36)	12.18 (1.72)
M	119 (4.92)	39.04 (12.72)	12.58 (1.72)
N	100 (4.14)	36.43 (12.43)	12.97 (1.75)
O	41 (1.7)	44.06 (10.73)	11.88 (1.53)
P	34 (1.41)	37.41 (14.67)	12.78 (2.00)
Q	234 (9.68)	41.52 (14.43)	12.21 (1.96)
R	162 (6.7)	39.12 (14.17)	12.55 (1.93)
S	29 (1.1.2)	42.97 (14.90)	11.99 (2.00)
T	76 (3.14)	42.87 (11.96)	12.04 (1.68)
U	5 (0.21)	37.52 (7.76)	12.77 (1.10)
W	1 (0.04)	55.33 (—^a^)	10.18 (—)
X	2 (0.08)	46.07 (1.43)	11.58 (0.24)
Y	1 (0.04)	14.07 (—)	16.16 (—)
Z	3 (0.12)	32.72 (8.61)	13.51 (1.14)

^a^No SD could be computed for 1 article.

Further descriptive values, such as minimum and maximum values of each readability metric, as well as mean (SD) and minimum and maximum values of sentence and word and complex word count for each ICD-10 chapter, can be found in Multimedia Appendix 10.

### Comparison of Readability

#### Comparison Among Languages

Interlanguage comparisons were conducted pairwise between each resulting pair of the 3 languages. For this purpose, the FRE values of all articles (ie, groups A and B) were considered. The readability of the Wikipedia texts (English: FRE=28.84; German: FRE=21.16; Russian: FRE=38.62) differed significantly among these values. The results of the corresponding unpaired 1-tailed *t* tests are presented in Table 9.

**Figure 8:**
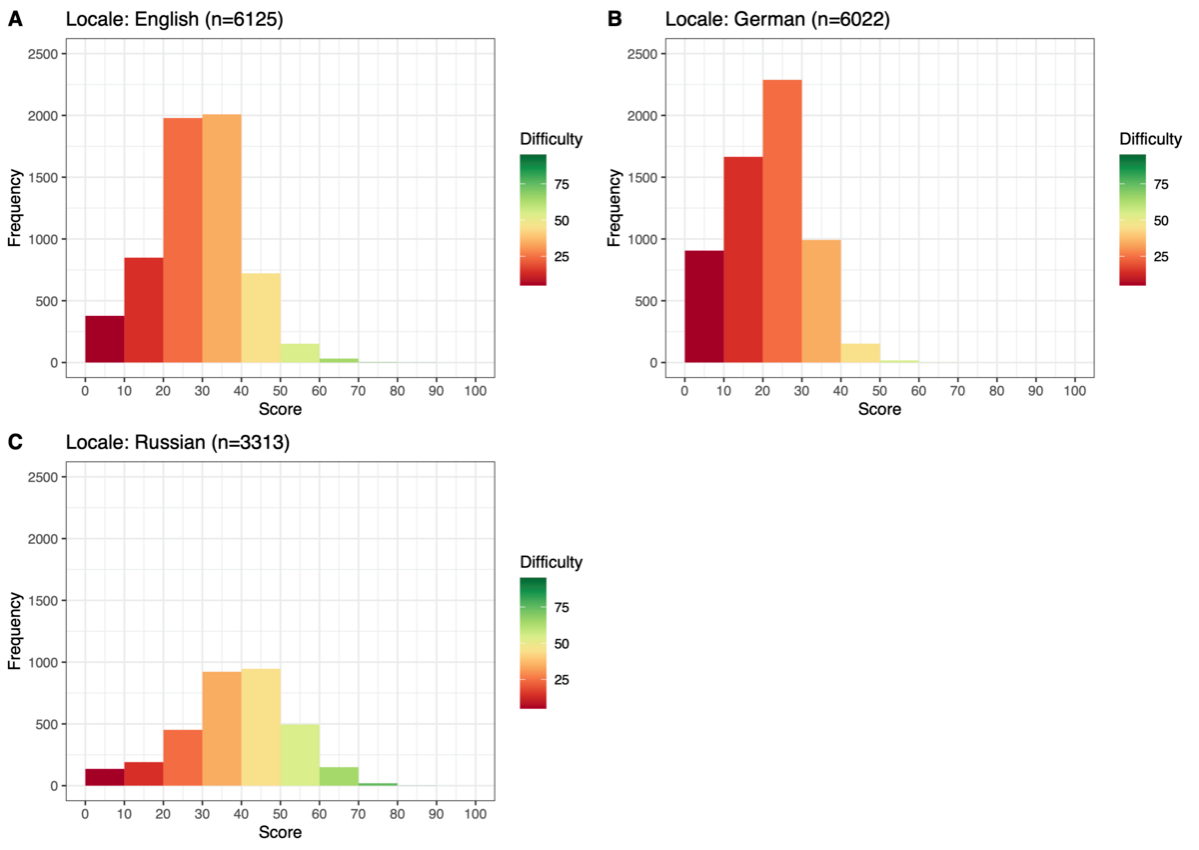
Distribution of Flesch Reading Ease (FRE) values of all articles included in the analysis from English (A), German (B), and Russian (C) Wikipedia.

The distribution of articles in English, German, and Russian Wikipedia based on their difficulty is depicted in Figure 8. The frequency of articles in Figure 8 is lower than that of the other 2 subplots, as the total number of articles from Russian Wikipedia is smaller than that from English and German Wikipedia.

More than 90% (9/10) of English Wikipedia articles (5937/6125, 96.93%) had an FRE value <50.00, which signals that they are difficult to extremely difficult to read. In total, 99.7% (6004/6022) of articles from German Wikipedia have an FRE value <50.00 and fall under the same category. Among the articles included in the analysis from Russian Wikipedia, 79.9% (2647/3313) had an FRE value <50.00.

**Table 9:** Comparison of Wikipedia articles in different languages for readability difficulty.

Comparison	Difference of means (95% CI)	*P* value
English vs German	7.68 (7.29 to 8.08)	<.001
English vs Russian	−9.78 (−10.34 to −9.22)	<.001
German vs Russian	−17.46 (−18.01 to −16.92)	<.001

#### Comparison Among ICD-10 Chapters

**Figure 9:**
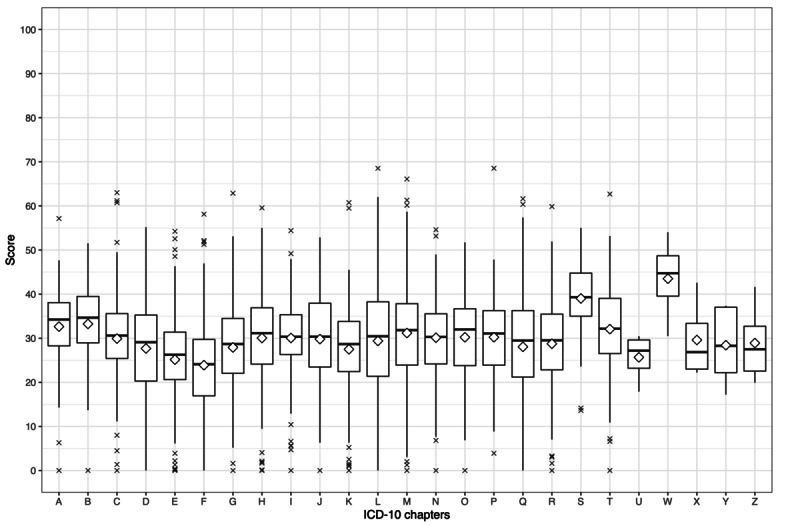
Box plot depicting Flesch Reading Ease values in each International Classification of Diseases, Tenth Revision (ICD-10), chapter for articles from English Wikipedia. The rhombus represents the mean Flesch Reading Ease value.

**Figure 10:**
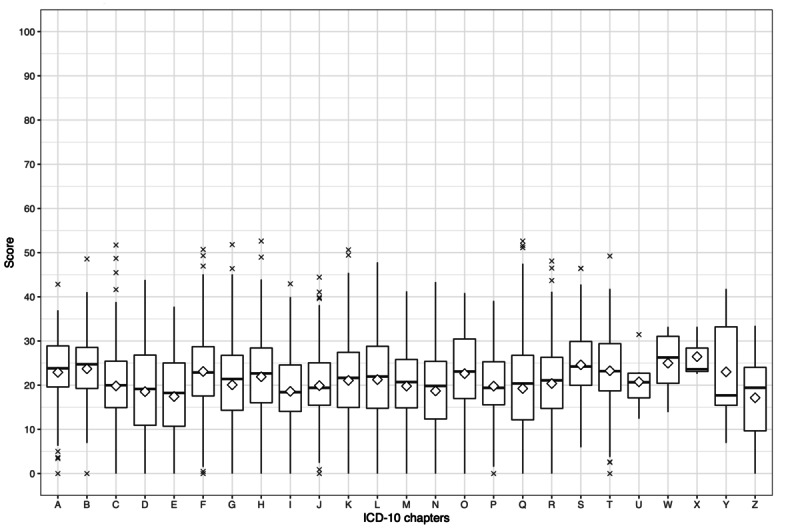
Box plot depicting Flesch Reading Ease values in each International Classification of Diseases, Tenth Revision (ICD-10) chapter for articles from German Wikipedia. The rhombus represents the mean Flesch Reading Ease value.

**Figure 11:**
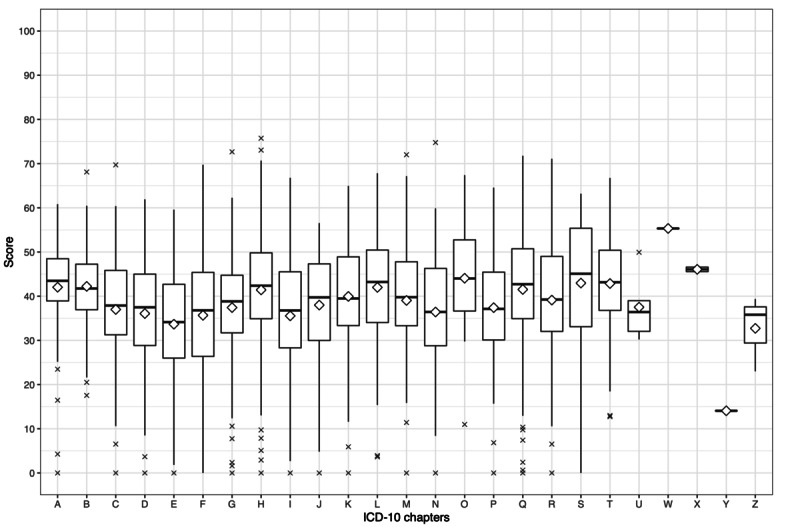
Box plot depicting Flesch Reading Ease values in each International Classification of Diseases, Tenth Revision (ICD-10), chapter for articles from Russian Wikipedia. The rhombus represents the mean Flesch Reading Ease value.

Unpaired 2-tailed *t* tests were conducted to investigate differences among ICD-10 chapters compared with the average FRE value of group A articles. This analysis was performed separately for each language. The average FRE values of group A articles were 28.69 (SD 11.00), 20.33 (SD 9.98), and 38.54 (SD 13.51) for English, German, and Russian Wikipedia, respectively (Tables 3, 5, and 7). Figure 9, Figure 10, and Figure 11 depict median and mean FRE values of every ICD-10 chapter for English, German, and Russian, respectively.

All values are below the FRE score of 70.00, which means that no articles in English group A were easy to read. Articles with the highest FRE scores required at least 8 or 9 years of education for adequate text comprehension.

Significant differences were found for 9 ICD-10 chapters in articles collected from English Wikipedia: chapter F (FRE 23.88, SD 9.95; *P*<.001), chapter E (FRE 25.14, SD 9.88; *P*<.001), chapter H (FRE 30.04, SD 10.57; *P*=.049), chapter I (FRE 30.05, SD 9.07; *P*=.04), chapter M (FRE 31.17, SD 11.94; *P*<.001), chapter T (FRE 32.06, SD 10.51; *P*=.001), chapter A (FRE 32.63, SD 9.25; *P*<.001), chapter B (FRE 33.24, SD 9.07; *P*<.001), and chapter S (FRE 39.02, SD 8.22; *P*<.001).

For the German group A, all articles scored below the FRE of 55.00 and, thus, were at least *fairly difficult* to *extremely difficult* to read. To adequately understand these articles, the reader needs at least 10 years of prior education.

In articles collected from German Wikipedia, the average readability values of 11 ICD-10 chapters differed significantly from the average value of group A articles: chapter E (FRE 17.43, SD 9.80; *P*<.001), chapter D (FRE 18.54, SD 10.22; *P*=.002), chapter I (FRE 18.60, SD 8.91; *P*=.006), chapter N (FRE 18.70, SD 9.09; *P*=.02), chapter Q (FRE 19.25, SD 10.60; *P*=.001), chapter H (FRE 21.91, SD 10.06; *P*=.009), chapter A (FRE 22.91, SD 8.77; *P*=.002), chapter F (FRE 23.10, SD 9.50; *P*<.001), chapter T (FRE 23.27, SD 9.55; *P*<.001), chapter B (FRE 23.71, SD 8.14; *P*<.001), and chapter S (FRE 24.60, SD 8.22; *P*<.001).

In total, 0.26% (6/2316) of group A articles from Russian Wikipedia had an FRE score between 70.00 and 80.00; that is, they were fairly easy to read and required 7 years of education for text comprehension.

For Russian Wikipedia articles, 10 ICD-10 chapters showed a significantly different difficulty in comparison with the group A average value: chapter E (FRE 33.66, SD 12.73; *P*<.001), chapter I (FRE 35.55, SD 13.69; *P*=.02), chapter F (FRE 35.68, SD 14.78; *P*=.001), chapter H (FRE 41.43, SD 13.59; *P*=.004), chapter Q (FRE 41.52, SD 14.43; *P*=.002), chapter L (FRE 41.99, SD 12.36; *P*=.01), chapter A (FRE 42.03, SD 11.38; *P*=.006), chapter B (FRE 42.22, SD 9.22; *P*<.001), chapter T (FRE 42.87, SD 11.96; *P*=.002), and chapter O (FRE 44.06, SD 10.73; *P*=.002).

For English and German, articles associated with the ICD-10 chapter S were, on average, significantly easier to read than all articles from group A. Articles from Russian Wikipedia were, on average, easier to read but showed no significant difference from the mean value. Chapter S codes represent *injury, poisoning, and certain other consequences of external causes.*

In contrast, articles associated with ICD-10 chapter F, which codes *mental, behavioral, and neurodevelopmental disorders*, were significantly harder to understand than an average group A article from English and Russian Wikipedia. The respective average value of this chapter in German Wikipedia was even lower than that in English Wikipedia but significantly higher than the average value of group A articles for German locale. Thus, in all 3 Wikipedia domains, articles related to mental, behavioral, and neurodevelopmental disorders were, on average, very difficult to read. For adequate comprehension, readers would need at least a college degree.

Chapter B (*certain infectious and parasitic diseases*), chapter T (*injury, poisoning, and certain other consequences of external causes*), chapter I (*diseases of the circulatory system*), and chapter H (*diseases of eye and adnexa*) had significant differences with the average readability of group A articles in all 3 languages. In Russian and German languages, chapters B, T, and H had significantly higher FRE values than the average, and chapter I had lower FRE values. For the data collection of English articles, all 4 chapters had significantly better readability than average.

Details for every ICD-10 chapter and each language can be found in Multimedia Appendix 10.

#### Comparison With Recommended Grade Level

For the English language, there is a recommended readability level of roughly *7-8* [26]. An unpaired 1-tailed *t* test was performed for articles from English Wikipedia. On average, every ICD-10 chapter had a significantly higher FKGL than the recommended grade level of 7 (*P*<.001).

Further details on FKGL results can be found in Multimedia Appendix 11.

## Discussion

### Principal Findings

Most articles were collected from English Wikipedia. Moreover, the original graph from English Wikipedia had the highest number of nodes in comparison with graphs generated from Russian and German Wikipedia. However, only 31.78% (1947/6127) of the articles included for the English locale were acquired in graph-based data collection (processing step 1). The remaining articles were collected in processing step 2 based on cross-linking from German and Russian Wikipedia. A possible reason for this may be the unsuitable category structure of English Wikipedia, which prevents the construction of an optimal graph for further processing. In contrast, German Wikipedia and Russian Wikipedia were found to have a better categorical structure, which led to most articles from these localizations being collected in the first graph-based data collection step.

Furthermore, Wikipedia articles provided good ICD-10 code coverage for disease-related articles. Most articles (English: 4235/6127, 69.12%; German: 4625/6024, 76.78%; Russian: 2316/3314, 69.89%) were assigned to group A because an ICD-10 code was provided.

The presence of an ICD-10 code associated with an article in Wikipedia may encourage readers to gather further information about the disease they are interested in. However, Wikipedia is not a medical textbook, and annotating ICD codes to an article is not strictly required. Moreover, it is questionable whether layperson readers or writers would be familiar with the concept of ICD codes. Although readers with a medical professional background could make use of it, the question remains whether those people are the target audience of Wikipedia.

On average, English articles in every single ICD-10 chapter failed to meet the readability of recommendation of the computed grade level of 7 [26].

In general, disease-related articles from all 3 Wikipedia domains were difficult and very difficult to read: pages from Russian Wikipedia were significantly easier to read with an overall FRE score of 38.62 (difficult to read), followed by pages from English (FRE 28.84) and German (FRE 21.16) Wikipedia with very difficult-to-understand texts. The relatively easier-to-understand ICD-10 chapter from German Wikipedia was still very difficult to read (FRE 24.60). On average, the least difficult chapters in English (FRE 39.02) and Russian (FRE 44.06) were identified as difficult to read. Most of the analyzed articles required a college to a professional level of education for adequate comprehension. Therefore, disease-related articles found on Wikipedia cannot be recommended as stand-alone educational materials for patients seeking information on the web. Nevertheless, patients will be confronted with this, as they use search engines that present Wikipedia pages in the top ranks [8-11].

### Limitations

One of the general limitations is the choice of the readability metric. In this study, classical readability metrics based on sentence and word structures were used for the analysis. However, such readability metrics provide insight into only one aspect of the understandability of a given text. Readability formulas ignore factors that can contribute to ease of reading but are not based on sentence structure and word length (eg, illustrations, sentence connection, and syntax). The role of the reader was also not taken into consideration [68]. Several studies have shown that other approaches to the analysis of readability have higher concordance with human assessment than the established readability formulas [45,46].

Moreover, the chosen readability metrics were mainly based on the ASL and number of syllables in each word, as well as language-specific weighting factors. However, estimating the number of syllables in a word is not a trivial task for German and Russian languages and does not always work reliably [69]. For this reason, the computed values could be affected by inaccuracies in the syllable count. In this context, it should be stressed that this affects all natural language processing analysis tools for German and Russian text materials.

There are also several technical limitations to be mentioned in the context of the study. First, Wikipedia graphs were generated based on data exported from June 2021. Therefore, our findings are valid only for that moment in time, as Wikipedia is updated daily and exports from June 2021 may not reflect the current situation.

Second, some relevant articles could have been missed because of the choice of the main concept and category filtering for each language. Similarly, some irrelevant articles could have been included in the analysis if they were incorrectly categorized by Wikipedia authors. However, irrelevant articles were mostly found in group B because their ICD-10 codes were not specified.

Furthermore, Wikipedia pages can be created by anyone and might not be strictly reviewed before publishing or making changes. Thus, inaccuracies and inconsistencies might be the outcomes for the readers of a page. In this context, the ICD-10 code could have been specified incorrectly. Some of the retrieved ICD-10 codes were manually excluded or adjusted by the authors. For instance, an article from German Wikipedia—*Computerspielabhängigkeit* (English: video game addiction; Russian: *Зависимость от комьютерных игор*)—was retrieved with an ICD-10 code *C51*, which stands for malignant neoplasms. In this case, the ICD-10 code template was used incorrectly by the authors of the article, who instead assigned an ICD-11 code (6C51 gaming disorder) to this article. There were also articles with falsely retrieved ICD-10 codes; for example, *0.00* was retrieved instead of *Q0.00* for the English article *Anencephaly* (German: *Anenzephalie*; Russian: *Aнэнцефалия*).

### Comparison With Prior Work

Previous studies have investigated readability for a relatively small sample of disease- or health-related English Wikipedia pages [10,18,19,34-36]. All studies consistently found that these are difficult to read and require at least a college degree or higher level of education. Our study confirms that English Wikipedia pages (still) require a college graduate degree on average.

Kräenbring et al [44] investigated the readability of German Wikipedia pages related to pharmacology. They found that, on average, related articles had a WSTF value of 15.04. In our study, disease-related pages in German showed an average WSTF value of 13.43 (group A, SD 1.28) and 13.05 (group B, SD 1.52). Our findings confirm that higher education is necessary to understand German Wikipedia articles.

To the best of the authors’ knowledge, this study is the first to analyze the disease-related content of Russian Wikipedia. No further studies have investigated the level of readability in comparison with other languages. As reported for English and German localizations, our findings indicate that readers of Russian articles require a comparatively high level of education.

In contrast to previous studies, this study analyzed the readability of 3 different Wikipedia languages, with each sample containing thousands of articles. In addition, this study presents a detailed comparison of all medical subfields based on the internationally adopted ICD-10 classification. All details of the analyses are available in Multimedia Appendices 3-11.

### Future Directions

In future work, the readability of other popular languages such as Spanish, French, and Chinese could be investigated to check whether differences exist. Furthermore, vocabulary analysis of Wikipedia pages can be conducted to add another dimension of understandability to established readability metrics, as demonstrated in the study by Zowalla et al [69].

Moreover, a consecutive study could draw a direct comparison of simplified versions of Wikipedia, such as *Simple English* (available on the web [70]). In this context, readability levels are expected to be significantly easier than those of regular versions.

Although readability analysis provides valuable insights into the understandability of texts, an investigation of content quality (eg, using DISCERN) could be beneficial for assessing the suitability of Wikipedia articles as educational material for patients. Similarly, the analysis of visual elements and information depictions could allow a more thorough investigation in the context of this topic. However, visual interpretation is subject to personal preferences, varies substantially, and requires tremendous manual effort. From a technical perspective, this is a challenge for even modern image-processing libraries.

To increase the readability of articles, Wikipedia can provide a built-in readability check that can serve two purposes: (1) informing authors about the readability values of the text and, thus, encouraging them to provide easier formulations and descriptions and (2) informing the reader about the difficulty of the currently displayed article.

### Conclusions

For the English, German, and Russian editions, disease-related Wikipedia pages were difficult to read and understand. For adequate comprehension, a college degree is required to understand articles from Russian Wikipedia, and a graduate degree is required for readers of the English and German Wikipedia editions.

Therefore, Wikipedia in all 3 languages cannot be recommended as a stand-alone patient education material. It does not meet the recommended readability for such materials written in English. Although no such recommendations are available for German and Russian, our findings confirm the low readability of pages for all 3 Wikipedia localizations.

The authors of Wikipedia pages should carefully revise existing text materials for readers with a specific interest in a disease or its associated symptoms. Special attention should be given to articles on *mental, behavioral, and neurodevelopmental disorders* (ICD-10 chapter F) as these articles were most difficult to read (FKGL 15.33, ARI 13.87, CLI 15.49, SMOG 17.22, and Gunning FOG 16.92) in comparison with other ICD-10 chapters.

A built-in readability indicator could be useful for authors contributing to Wikipedia. This would increase readability at the text production stage and, thus, allow more people to comprehend medical knowledge through encyclopedias, which are freely available on the internet.

## Appendix

Multimedia Appendix 1 Systematic literature review.

Multimedia Appendix 2 General statistics for disease-related Wikipedia graphs.

Multimedia Appendix 3 Terms and wildcards applied for category filtering.

Multimedia Appendix 4 Distributions of all computed readability metrics in English.

Multimedia Appendix 5 Distributions of all computed readability metrics in German.

Multimedia Appendix 6 Distributions of all computed readability metrics in Russian.

Multimedia Appendix 7 Box plots with readability values for the English sample.

Multimedia Appendix 8 Box plots with readability values for the German sample.

Multimedia Appendix 9 Box plots with readability values for the Russian sample.

Multimedia Appendix 10 Tables containing mean (SD) and minimum and maximum values for all computed readability metrics for articles in groups A and B, as well as for every International Classification of Diseases, Tenth Revision, chapter separately.

Multimedia Appendix 11 *P* and respective mean values of all conducted t tests for the second and third test scenarios.

## Abbreviations

ARI: Automated Readability Index
ASL: average sentence length
ASW: average syllables per word
CLI: Coleman-Liau Index
FKGL: Flesch-Kincaid Grade Level
FOG: Frequency of Gobbledygook
FRE: Flesch Reading Ease
ICD-10: International Classification of Diseases, Tenth Revision
ICD-11: International Classification of Diseases, Eleventh Revision
MS: proportion of (complex) words with ≥3 syllables
SMOG: Simple Measure of Gobbledygook
WSTF: Wiener SachTextFormel

## References

[1] Marton C, Wei Choo C (2012). A review of theoretical models of health information seeking on the web. J Doc.

[2] Jacobs W, Amuta AO, Jeon KC (2017). Health information seeking in the digital age: an analysis of health information seeking behavior among US adults. Cogent Soc Sci.

[3] Keinki C, Zowalla R, Wiesner M, Koester MJ, Huebner J (2018). Understandability of patient information booklets for patients with cancer. J Cancer Educ.

[4] Sillence E, Briggs P, Harris PR, Fishwick L (2007). How do patients evaluate and make use of online health information?. Soc Sci Med.

[5] Moreland J, French TL, Cumming GP (2015). The prevalence of online health information seeking among patients in Scotland: a cross-sectional exploratory study. JMIR Res Protoc.

[6] Bjelke M, Martinsson AK, Lendahls L, Oscarsson M (2016). Using the Internet as a source of information during pregnancy - a descriptive cross-sectional study in Sweden. Midwifery.

[7] Shea-Budgell MA, Kostaras X, Myhill KP, Hagen NA (2014). Information needs and sources of information for patients during cancer follow-up. Curr Oncol.

[8] (2021). The top 500 sites on the web. Alexa.

[9] Top-500 Registered Domains of the Latest Main Crawl. Common Crawl.

[10] Hutchinson N, Baird GL, Garg M (2016). Examining the reading level of Internet medical information for common internal medicine diagnoses. Am J Med.

[11] Seth AK, Vargas CR, Chuang DJ, Lee BT (2016). Readability assessment of patient information about lymphedema and its treatment. Plast Reconstr Surg.

[12] Wikipedia: size of Wikipedia. Wikipedia - The Free Encyclopedia.

[13] Chrzanowski J, Sołek J, Fendler W, Jemielniak D (2021). Assessing public interest based on Wikipedia's most visited medical articles during the SARS-CoV-2 outbreak: search trends analysis. J Med Internet Res.

[14] Matheson D, Matheson-Monnet C (2017). Wikipedia as informal self-education for clinical decision-making in medical practice. Open Med J.

[15] Smith DA (2020). Situating Wikipedia as a health information resource in various contexts: a scoping review. PLoS One.

[16] Farič N, Potts HW (2014). Motivations for contributing to health-related articles on Wikipedia: an interview study. J Med Internet Res.

[17] Volsky PG, Baldassari CM, Mushti S, Derkay CS (2012). Quality of Internet information in pediatric otolaryngology: a comparison of three most referenced websites. Int J Pediatr Otorhinolaryngol.

[18] John AM, John ES, Hansberry DR, Thomas PJ, Guo S (2015). Analysis of online patient education materials in pediatric ophthalmology. J AAPOS.

[19] Shetty KR, Wang RY, Shetty A, Levi J, Aaronson NL (2020). Quality of patient education sections on Otitis media across different website platforms. Ann Otol Rhinol Laryngol.

[20] Garcia SF, Hahn EA, Jacobs EA (2010). Addressing low literacy and health literacy in clinical oncology practice. J Support Oncol.

[21] Alpay L, Verhoef J, Xie B, Te'eni D, Zwetsloot-Schonk JH (2009). Current challenge in consumer health informatics: bridging the gap between access to information and information understanding. Biomed Inform Insights.

[22] Fox NJ, Ward KJ, O'Rourke AJ (2005). The 'expert patient': empowerment or medical dominance? The case of weight loss, pharmaceutical drugs and the Internet. Soc Sci Med.

[23] Broom A (2005). Virtually he@lthy: the impact of Internet use on disease experience and the doctor-patient relationship. Qual Health Res.

[24] Tan SS, Goonawardene N (2017). Internet health information seeking and the patient-physician relationship: a systematic review. J Med Internet Res.

[25] Oh HJ, Lee B (2012). The effect of computer-mediated social support in online communities on patient empowerment and doctor-patient communication. Health Commun.

[26] How to Write Easy-to-Read Health Materials. US National Library of Medicine.

[27] Flesch R (1948). A new readability yardstick. J Appl Psychol.

[28] Gunning R (1968). The Technique of Clear Writing.

[29] McLaughlin GH (1969). SMOG grading: a new readability formula. J Read.

[30] Coleman M, Liau TL (1975). A computer readability formula designed for machine scoring. J Appl Psychol.

[31] Fry E (1977). Fry's readability graph: clarifications, validity, and extension to level 17. J Read.

[32] Amstad T (1978). Wie verständlich sind unsere Zeitungen?.

[33] Solovyev V, Ivanov V, Solnyshkina M (2018). Assessment of reading difficulty levels in Russian academic texts: approaches and metrics. J Intell Fuzzy Syst.

[34] Suwannakhan A, Casanova-Martínez D, Yurasakpong L, Montriwat P, Meemon K, Limpanuparb T (2020). The quality and readability of English Wikipedia anatomy articles. Anat Sci Educ.

[35] Brigo F, Otte WM, Igwe SC, Tezzon F, Nardone R (2015). Clearly written, easily comprehended? The readability of websites providing information on epilepsy. Epilepsy Behav.

[36] Handler SJ, Eckhardt SE, Takashima Y, Jackson AM, Truong C, Yazdany T (2021). Readability and quality of Wikipedia articles on pelvic floor disorders. Int Urogynecol J.

[37] McEnteggart GE, Naeem M, Skierkowski D, Baird GL, Ahn SH, Soares G (2015). Readability of online patient education materials related to IR. J Vasc Interv Radiol.

[38] McInnes N, Haglund BJ (2011). Readability of online health information: implications for health literacy. Inform Health Soc Care.

[39] Miles RC, Baird GL, Choi P, Falomo E, Dibble EH, Garg M (2019). Readability of online patient educational materials related to breast lesions requiring surgery. Radiology.

[40] Modiri O, Guha D, Alotaibi NM, Ibrahim GM, Lipsman N, Fallah A (2018). Readability and quality of Wikipedia pages on neurosurgical topics. Clin Neurol Neurosurg.

[41] Punia V, Dagar A, Agarwal N, He W, Hillen M (2014). Comparison of neurological healthcare oriented educational resources for patients on the Internet. J Clin Neurosci.

[42] Rajagopalan MS, Khanna VK, Leiter Y, Stott M, Showalter TN, Dicker AP, Lawrence YR (2011). Patient-oriented cancer information on the Internet: a comparison of Wikipedia and a professionally maintained database. J Oncol Pract.

[43] Tulbert BH, Snyder CW, Brodell RT (2011). Readability of patient-oriented online dermatology resources. J Clin Aesthet Dermatol.

[44] Kräenbring J, Monzon Penza T, Gutmann J, Muehlich S, Zolk O, Wojnowski L, Maas R, Engelhardt S, Sarikas A (2014). Accuracy and completeness of drug information in Wikipedia: a comparison with standard textbooks of pharmacology. PLoS One.

[45] Zheng J, Yu H (2018). Assessing the readability of medical documents: a ranking approach. JMIR Med Inform.

[46] Kauchak D, Leroy G, Hogue A (2017). Measuring text difficulty using parse-tree frequency. J Assoc Inf Sci Technol.

[47] Readability Formulas.

[48] Wikipedia - The Free Encyclopedia.

[49] Willkommen bei Wikipedia.

[50] Добро пожаловать в Википедию.

[51] (2016). ICD-10: International statistical classification of diseases and related health problems: 10th revision. World Health Organization.

[52] Frequently asked questions. World Health Organization.

[53] (2022). Template:ICD-10. Wikipedia, the free encyclopedia.

[54] (2022). Vorlage:Infobox ICD. Wikipedia, the free encyclopedia.

[55] (2022). Шаблон:ICD10. Wikipedia, the free encyclopedia.

[56] Harrison JE, Weber S, Jakob R, Chute CG (2021). ICD-11: an international classification of diseases for the twenty-first century. BMC Med Inform Decis Mak.

[57] (2021). International Statistical Classification of Diseases and Related Health Problems (ICD). World Health Organization.

[58] Klare GR (1974). Assessing readability. Read Res Q.

[59] Klare GR, Zakaluk BL, Samuels SJ (1988). The formative years. Readability: its past, present, and future.

[60] Kincaid JP, Fishburne Jr RP, Rogers RL, Chissom BS (1975). Derivation Of New Readability Formulas (Automated Readability Index, Fog Count And Flesch Reading Ease Formula) For Navy Enlisted Personnel. Institute for Simulation and Training.

[61] Smith EA, Senter RJ (1967). Automated readability index. AMRL TR.

[62] Bamberger R, Vanecek E (1984). Lesen - Verstehen - Lernen - Schreiben: die Schwierigkeitsstufen von Texten in deutscher Sprache.

[63] Wikimedia Downloads.

[64] Wikidata Toolkit. MediaWiki.

[65] Wiesner M, Zowalla R, Pobiruchin M (2020). The difficulty of German information booklets on psoriasis and psoriatic arthritis: automated readability and vocabulary analysis. JMIR Dermatol.

[66] Wickham H (2016). ggplot2: Elegant Graphics For Data Analysis. 2nd edition.

[67] Holm S (1979). A simple sequentially rejective multiple test procedure. Scand J Stat.

[68] (2010). Using readability formulas: a cautionary note. Centers for Medicare & Medicaid Services, U.S. Department of Health and Human Services.

[69] Zowalla R, Wiesner M, Pfeifer D (2014). Automatically assessing the expert degree of online health content using SVMs. Stud Health Technol Inform.

[70] Simple English Wikipedia.

