# Supplementary material for: Readability of English, German, and Russian Disease-Related Wikipedia Pages: Automated Computational Analysis
Source: J Med Internet Res. 2022 May 16;24(5):e36835. doi: 10.2196/36835 (PMC9152717; doi:10.2196/36835)
Supplement: Multimedia Appendix 1 [file jmir_v24i5e36835_app1.pdf]

Multimedia Appendix 1: Systematic Literature Review

Eligibility criteria

Category	*Criterion*	*Inclusion*	*Exclusion*
C1	Language	English, German, Russian	Any other language
C2	Time period	Any	-
C3	Aim	Readability analysis of Wikipedia (either solely or among other factors)	Developing a readability tool or ML model, etc. (incl. when Wikipedia articles were used in the process)
C4	Readability measures used	Any known / well-established readability formula	Any other method for measuring readability (e.g. via measuring fixation of eyesight); measure was not clear or transparent
C5	Methodology of readability analysis	Reports used methods in a clear and transparent manner	Was not reported clearly or transparently
C6	Analyzed texts	Wikipedia articles (either solely or among other sources)	Any other texts (EHR notes, other webpages)
C7	Reported results	Clear, transparent and detailed reporting on readability of Wikipedia articles	Summarized readability statistics over different texts, no transparent reporting using numerical values

Information sources

Databases used in this review were:

- PubMed/MEDLINE - https://pubmed.ncbi.nlm.nih.gov
- ACM Digital Library - https://dl.acm.org
- IEEEXplore - https://ieeexplore.ieee.org

Search strategies

Search string for PubMed/MEDLINE

```
(wikipedia[Title/Abstract]) AND (readability[Title/Abstract])
```

Search string for ACM Digital Library

```
[[Publication Title: wikipedia] OR [Abstract: wikipedia]] AND
[[Publication Title: readability] OR [Abstract: readability]]
```

Search string for IEEEXplore

```
(wikipedia) AND (readability)
```

Collection process

The primary author (JG) conducted the search and retrieval process on 20-09-2021. The full list of search results was downloaded and stored in a literature database for the screening process, see "List of all screened articles".
All authors discussed and agreed on excluded articles.

Study selection

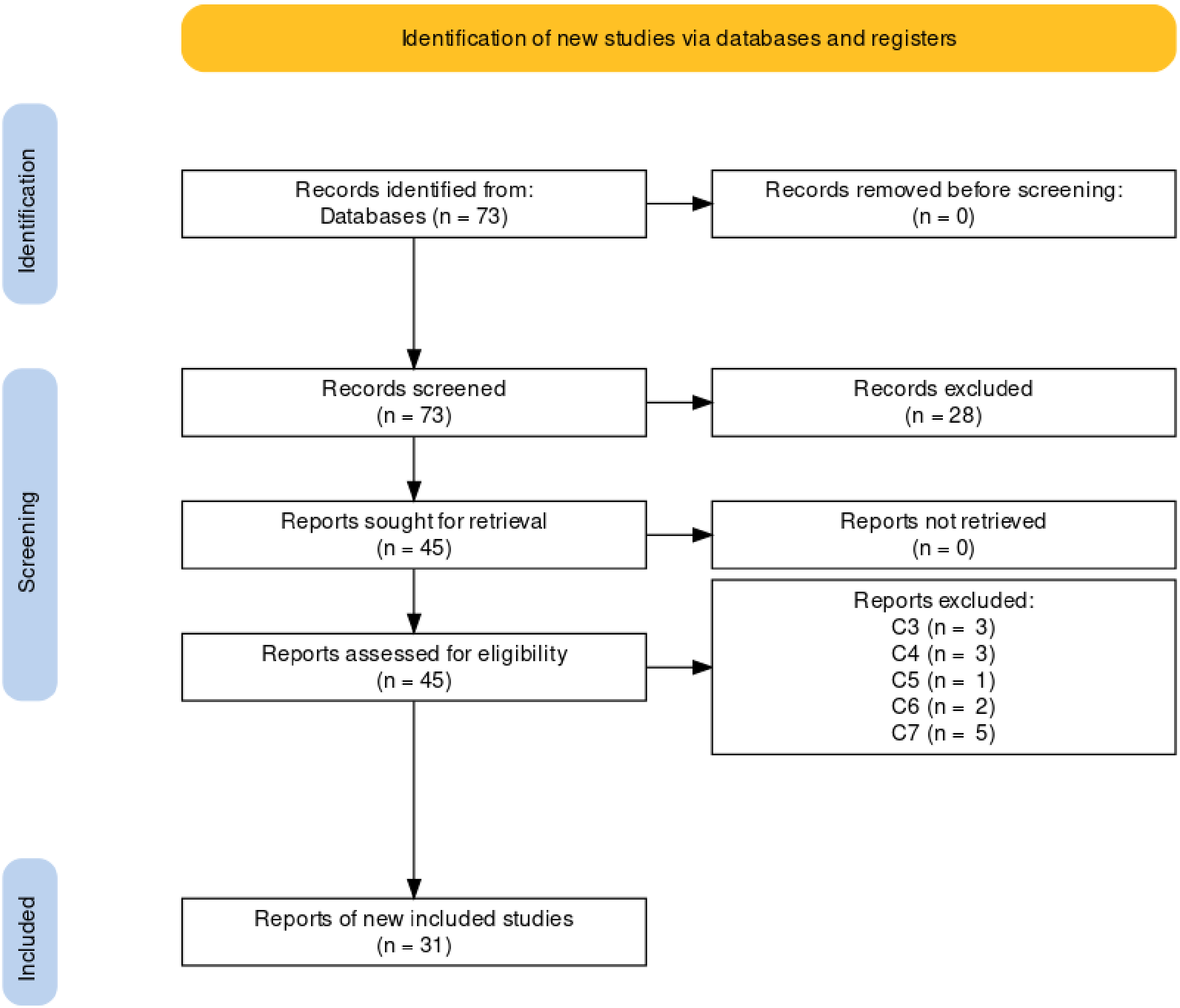

Details on excluded records (n=28) during screening:

Citations	*Criterion*
[19], [26],[42], [46]-[49], [53], [56]-[59], [61]-[71], [73]	C3
[1] [6] [28]	C6
[72]	Duplicate of [57]

Details on excluded reports (n=14) during assessment for eligibility:

Citations	*Criterion*
[54] [55] [50]	C3
[5] [50] [51]	C4
[9]	C5
[43] [45]	C6
[12] [13] [14] [18] [11]	C7

Study characteristics

Citations	*Title*	*Year*	*Methods*	*Results*
[2]	Evaluation of gastroenterology and hepatology articles on Wikipedia: are they suitable as learning resources for medical students?	2014	Wikipedia was searched for gastroenterology and hepatology articles (based on textbooks). Total: 39 articles.	Wikipedia articles are geared more to college level than to the general public. FK: range of -8.0+/-55.7 to 44.4+/-1.4; average - 26+/-9.0 (mean+/-SD).
[3]	AIs Wikipedia a reliable learning resource for medical students? Evaluating respiratory topics.	2015	Wikipedia was searched for respiratory articles (based on textbooks). Total: 40 articles.	FK: range of 9.4 ± 1.8 – 22.6 ± 10.7; CL: range of 10.0 ± 2.6–19.6 ± 8.3. Wikipedia articles were geared to a college level rather than to the public.
[4]	Accuracy and readability of cardiovascular entries on Wikipedia: are they reliable learning resources for medical students?	2015	Wikipedia was searched for cardiovascular articles (based on textbooks). Total: 47 articles.	FK: range of 10.6±1.1 - 20.6±8.3. The mean: 14.3±1.7 (mean±SD). The results are consistent with a college reading level.
[7]	The readability of the English Wikipedia article on Parkinson's disease.	2015	Wikipedia article on Parkinson's disease was analyzed.	FRE: 30.31; other: grade level 13.37 to 15.16 with 16.22 years of formal education. Overall low grade of readability.
[8]	Clearly written, easily comprehended? The readability of websites providing information on epilepsy.	2015	41 Wikipedia article on epilepsy were selected (additionally to other sources).	FRE avg: 25.6 ± 9.5. Other scales: 14th academic grade level (14.3 ± 1.7) and 16.4 ± 2.0 years of formal education.

[10]	Completeness, accuracy, and readability of Wikipedia as a reference for patient medication information.	2017	33 drugs from top 200 selected: Wikipedia articles + medication guides.	Wikipedia pages had a significantly higher reading level compared with medication guides. FRE: 52.93 vs. 33.24; FK: 10.26 vs. 6.86
[15]	Readability and quality of Wikipedia articles on pelvic floor disorders.	2021	Wikipedia articles on pelvic floor disorders were selected (based on the series of patient education leaflets published by the IUGA). Total: 30 articles vs 29 leaflets.	SMOG: 12.0 ± 2.14 (Wiki) vs 3.4 ± 0.30. Wikipedia articles required between an 11th- and 13th-grade education.
[16]	Examining the Reading Level of Internet Medical Information for Common Internal Medicine Diagnoses.	2016	Google was searched for 9 common internal med. diagnoses. Wikipedia among top 5 websites.	Avg grade level: 14.6 - highest among all sources.
[17]	Analysis of online patient education materials in pediatric ophthalmology	2015	Google was searched for paediatric ophthalmology diagnoses. Wikipedia was searched additionally for this topic.	Wikipedia avg. grade level: 17.4 ± 1.18 - highest among all sources.
[20]	Complementary and alternative medicine on wikipedia: opportunities for improvement.	2014	Terms included in the "list of branches of alternative medicine" on the English Wikipedia were identified. Total: 97 included articles.	Median SMOG: 12.7 (CAM) vs 14.4 (conventional med.).
[21]	Accuracy and completeness of drug information in Wikipedia: a comparison with standard textbooks of pharmacology.	2014	100 drugs selected from 300 curricular drugs. Included were Wikipedia articles that overlapped with information in two textbooks. For each drug, three text passages ranging between 90 and 120 words were randomly selected in the German language version of Wikipedia and in the textbooks.	No significant difference between Wikipedia and textbooks (RAmstad: 7.1±1.7 vs. 7.4±1.8, p = 0.9; R1. WSTF: 15.4±0.5 vs.14.5±0.2, p = 0.07;) -> difficult-to-read texts that require tertiary levels of education.

[22]	Readability of Online Patient Education Materials Related to IR.	2015	Google was searched for key disease/procedure phrases (topic: interventional radiology). Seven websites incl. Wikipedia were chosen.	Wikipedia avg. grade level: 16 - highest among all sources.
[23]	Readability of online health information: implications for health literacy. Inform Health Soc Care.	2011	5 search engines were searched for 13 unique causes of burden and mortality.	Wikipedia pages (avg. reading grade 15.21, 95% CI 1⁄4 14.44–15.99) were significantly harder to read than others.
[24]	Readability of Online Patient Educational Materials Related to Breast Lesions Requiring Surgery.	2019	Google was searched for 11 breast lesions. Wikipedia was included among 8 other websites.	Wikipedia had the highest average grade level readability score (ie, 14.2).
[25]	Readability and quality of wikipedia pages on neurosurgical topics.	2018	Wikipedia articles on neurosurgery were selected (based on the series of online patient information articles that are published by the American Association of Neurological Surgeons (AANS)). Total: 55 articles.	FK (mean): 31.10. Wikipedia articles were more difficult to read across every scale comparing to AANS articles.
[27]	Analysis of the accuracy and readability of herbal supplement information on Wikipedia.	2014	Wikipedia articles for the top 19 dietary herbal supplements identified by the National Center for Health Statistics (NCHS) were selected. Total: 19 articles.	FK avg. : 13.48; range of 9.9 - 16.9.
[29]	Comparison of neurological healthcare oriented educational resources for patients on the internet.	2014	Google was search for each disease with OPEM (online patient education material) available on the AAN (American Academy of Neurology) website. Wikipedia and 3 other resources were analyzed.	FRE: (with exception of tremors) all OPEM scored below 30, meaning they were "very confusing". Wikipedia had highest grade level among all resources.

[30]	Patient-oriented cancer information on the internet: a comparison of wikipedia and a professionally maintained database.	2011	Wikipedia articles where analyzed and compared to patient-oriented Physician Data Query maintained by the National Cancer Institute (NCI) for 10 different cancer types.	PDQ grade level of 9.6 ± 1.5 SD vs Wikipedia of 14.1 ± 0.5. Wikipedia less readable.
[31]	Quality of information sources about mental disorders: a comparison of Wikipedia with centrally controlled web and printed sources.	2012	Wikipedia articles (and other resources) were analyzed in comparison to Encyclopaedia Britannica, and a psychiatry textbook (based on the top 10 Google search results for either of the terms ‘depression’ or ‘schizophrenia’).	Wikipedia: FK (depression): ~14.5; FK (schizophrenia): ~16.1 (see graphic) -> requiring higher levels of education than completion of secondary schooling.
[32]	Assessment of Patient Information about Lymphedema and Its Treatment.	2016	Google was searched for the term "lymphedema". Top 12 websites (incl. Wikipedia) were analyzed.	Wikipedia had highest avg. readability score: 16.7
[33]	Quality of Patient Education Sections on Otitis Media Across Different Website Platforms.	2020	A search was conducted for patient materials pertaining to otitis media across 6 different websites including Wikipedia.	Across all six sites, Wikipedia had the highest reading level at a high school senior to college level: FK - 12.5; GFog - 15.95; SMOG - 14.6; CL - 12.64; ARI - 11.92.
[34]	The Quality and Readability of English Wikipedia Anatomy Articles.	2020	Anatomy articles selected from Wikipedia. Total: 40 articles.	FK avg.: 12.3 ± 2.1; FRE avg.: 42.4 ± 10.8 -> understandable to college students.
[35]	An evaluation of Wikipedia as a resource for patient	2013	Wikipedia articles on nephrology were selected (corresponding to ICD-10 codes). Total: 69 articles of the 95 ICD-10 codes.	Mean FK, ARI, FRE: 15.1 ± 0.3, 13.8 ± 0.4, and 19.4 ± 2.0 -> college degree level.

	education in nephrology.			
[36]	Readability of Patient-oriented Online Dermatology Resources.	2011	Google was searched for 4 topics on dermatology (based on educational pamphlets). Wikipedia included among other sources.	Wikipedia had the worst readability with respect to both FKGL (11.8) and FRE (35.5) -> significantly worse readability than the AAD Pamphlets, WebMD, and Medicine Online ($p<0.01$).
[37]	Quality of Internet information in pediatric otolaryngology: a comparison of three most referenced websites.	2012	Google was searched for 24 common diagnoses in pediatric otolaryngology. Wikipedia among top three occurring sites.	Wikipedia avg. FK: 12.8 (second highest among the three sites).
[38]	Readability of Wikipedia Pages on Autoimmune Disorders: Systematic Quantitative Assessment.	2017	Wikipedia articles on 134 autoimmune diseases selected (based on American Autoimmune Related Diseases Association (AARDA) website). Total: 134 articles.	The mean FRE: 24.34 (SD 10.73) -> very difficult to read and understand. Other scores: 14th to 15th academic grade level -> suitable for a university graduate reading level / low level of readability.
[39]	Wikipedia in Vascular Surgery Medical Education: Comparative Study.	2020	Wikipedia was searched for 8 topics in vascular surgery (based on Medical Council of Canada Objectives for the Qualifying Examination). In comparison to corresponding chapters of Schwartz Principles of Surgery. Total: 8 articles.	FRE avg.: 30.5; SD 8.4 (Wikipedia) vs 20.2; SD 9.0 (Schwartz); mean GL: 14.2; SD 1.3 (Wikipedia) vs 15.9; SD 1.4 (Schwartz).
[40]	A practical approach to language complexity: a Wikipedia case study.	2012	Corpora was built from the dumps of Simple and Main Wikipedias. Simple corpus: all texts of Simple Wikipedia; main corpus: randomly selected texts having the same sizes as the Simple articles.	Gfog avg.: 10.8±0.2 (Simple) vs 15.8±0.4 (Main) -> Main is much harder to read.
[41]	Readability Formulas and User Perceptions of Electronic Health	2017	Documents about diabetes collected from Wikipedia (Diabetes category was traversed) and EHR notes.	Wikipedia avg. FK; SMOG; GFog: 14.75; 11.07; 12.33 -> similar levels to EHR.

	Records Difficulty: A Corpus Study.			
[44]	Is wikipedia too difficult? comparative analysis of readability of wikipedia, simple wikipedia and britannica.	2012	Wikipedia articles compared in readability to Simple Wikipedia and Britannica. Wikipedia vs Simple Wiki – 25,970 corresponding article pairs selected. Wikipedia Categories readability assessed in 8 categories with up to 500 articles in each.	The percentage difference of readability is relatively stable for most of the used measures (average difference of 26%) apart for the case of FRE (66% difference) -> Simple Wikipedia is more readable than Wikipedia. Different categories have sometimes different readability levels and the familiarity-based metrics tend to produce more varying results than the syntactical readability measures (for detailed results see graphics in Word file).
[52]	(Don't) Mention the War: A Comparison of Wikipedia and Britannica Articles on National Histories.	2018	Readability of Britannica was compared to readability of Wikipedia (Historical articles extracted via mentioned years; focus on the history of 193 countries which are the current UN memberstates).	FRE avg.: 46.67±6.3 (Wikipedia) vs 42.9±5.9 (Britannica). Other scores for Wikipedia: range of 8.8±0.8 - 14.5±1.5.

List of all screened publications

1. Arts H, Lemetyinen H, Edge D. Readability and quality of online eating disorder information-Are they sufficient? A systematic review evaluating websites on anorexia nervosa using DISCERN and Flesch Readability. Int J Eat Disord. 2020;53(1):128-32. doi: 10.1002/eat.23173.
2. Azer SA. Evaluation of gastroenterology and hepatology articles on Wikipedia: are they suitable as learning resources for medical students? Eur J Gastroenterol Hepatol. 2014;26(2):155-63. doi: 10.1097/MEG.0000000000000003.
3. Azer SA. Is Wikipedia a reliable learning resource for medical students? Evaluating respiratory topics. Adv Physiol Educ. 2015;39(1):5-14. doi: 10.1152/advan.00110.2014.
4. Azer SA, AlSwaidan NM, Alshwairikh LA, AlShammari JM. Accuracy and readability of cardiovascular entries on Wikipedia: are they reliable learning resources for medical students? BMJ Open. 2015;5(10):e008187. doi: 10.1136/bmjopen-2015-008187.
5. Azzam A, Bresler D, Leon A, Maggio L, Whitaker E, Heilman J, et al. Why Medical Schools Should Embrace Wikipedia: Final-Year Medical Student Contributions to Wikipedia Articles for Academic Credit at One School. Acad Med. 2017;92(2):194-200. doi: 10.1097/ACM.0000000000001381.
6. Behmer Hansen R, Gold J, Lad M, Gupta R, Ganapa S, Mammis A. Health literacy among neurosurgery and other surgical subspecialties: Readability of online patient materials found with Google. Clin Neurol Neurosurg. 2020;197:106141. doi: 10.1016/j.clineuro.2020.106141.
7. Brigo F, Erro R. The readability of the English Wikipedia article on Parkinson's disease. Neurol Sci. 2015;36(6):1045-6. doi: 10.1007/s10072-015-2077-5.
8. Brigo F, Otte WM, Igwe SC, Tezzon F, Nardone R. Clearly written, easily comprehended? The readability of websites providing information on epilepsy. Epilepsy Behav. 2015;44:35-9. doi: 10.1016/j.yebeh.2014.12.029.
9. Burgos C, Bot A, Ring D. Evaluating the effectiveness of a wiki internet site for medical topics. J Hand Microsurg. 2012;4(1):21-4. doi: 10.1007/s12593-012-0064-0.
10. Candelario DM, Vazquez V, Jackson W, Reilly T. Completeness, accuracy, and readability of Wikipedia as a reference for patient medication information. J Am Pharm Assoc (2003). 2017;57(2):197-200.e1. doi: 10.1016/j.japh.2016.12.063.
11. Dunne SS, Cummins NM, Hannigan A, Shannon B, Dunne C, Cullen W. Generic medicines: an evaluation of the accuracy and accessibility of information available on the Internet. BMC Med Inform Decis Mak. 2013;13:115. doi: 10.1186/1472-6947-13-115.
12. Garcia M, Daugherty C, Ben Khallouq B, Maugans T. Critical assessment of pediatric neurosurgery patient/parent educational information obtained via the Internet. J Neurosurg Pediatr. 2018;21(5):535-41. doi: 10.3171/2017.10.PEDS17177.
13. Grohol JM, Slimowicz J, Granda R. The quality of mental health information commonly searched for on the Internet. Cyberpsychol Behav Soc Netw. 2014;17(4):216-21. doi: 10.1089/cyber.2013.0258.
14. Guitton MJ. Online maritime health information: an overview of the situation. Int Marit Health. 2015;66(3):139-44. doi: 10.5603/IMH.2015.0028.
15. Handler SJ, Eckhardt SE, Takashima Y, Jackson AM, Truong C, Yazdany T. Readability and quality of Wikipedia articles on pelvic floor disorders. Int Urogynecol J. 2021. doi: 10.1007/s00192-021-04776-0.
16. Hutchinson N, Baird GL, Garg M. Examining the Reading Level of Internet Medical Information for Common Internal Medicine Diagnoses. Am J Med. 2016;129(6):637-9. doi: 10.1016/j.amjmed.2016.01.008.
17. John AM, John ES, Hansberry DR, Thomas PJ, Guo S. Analysis of online patient education materials in pediatric ophthalmology. J aapos. 2015;19(5):430-4. doi: 10.1016/j.jaapos.2015.07.286.

18. Kang R, Lipner S. Assessment of internet sources on subungual melanoma. Melanoma Res. 2020;30(4):416-9. doi: 10.1097/CMR.0000000000000508.
19. Kauchak D, Leroy G, Hogue A. Measuring Text Difficulty Using Parse-Tree Frequency. J Assoc Inf Sci Technol. 2017;68(9):2088-100. doi: 10.1002/asi.23855.
20. Koo M. Complementary and alternative medicine on wikipedia: opportunities for improvement. Evid Based Complement Alternat Med. 2014;2014:105186. doi: 10.1155/2014/105186.
21. Kräenbring J, Monzon Penza T, Gutmann J, Muehlich S, Zolk O, Wojnowski L, et al. Accuracy and completeness of drug information in Wikipedia: a comparison with standard textbooks of pharmacology. PLoS One. 2014;9(9):e106930. doi: 10.1371/journal.pone.0106930.
22. McEnteggart GE, Naeem M, Skierkowski D, Baird GL, Ahn SH, Soares G. Readability of Online Patient Education Materials Related to IR. J Vasc Interv Radiol. 2015;26(8):1164-8. doi: 10.1016/j.jvir.2015.03.019.
23. McInnes N, Haglund BJ. Readability of online health information: implications for health literacy. Inform Health Soc Care. 2011;36(4):173-89. doi: 10.3109/17538157.2010.542529.
24. Miles RC, Baird GL, Choi P, Falomo E, Dibble EH, Garg M. Readability of Online Patient Educational Materials Related to Breast Lesions Requiring Surgery. Radiology. 2019;291(1):112-8. doi: 10.1148/radiol.2019182082.
25. Modiri O, Guha D, Alotaibi NM, Ibrahim GM, Lipsman N, Fallah A. Readability and quality of wikipedia pages on neurosurgical topics. Clin Neurol Neurosurg. 2018;166:66-70. doi: 10.1016/j.clineuro.2018.01.021.
26. Mukherjee P, Leroy G, Kauchak D, Rajanarayanan S, Romero Diaz DY, Yuan NP, et al. NegAIT: A new parser for medical text simplification using morphological, sentential and double negation. J Biomed Inform. 2017;69:55-62. doi: 10.1016/j.jbi.2017.03.014.
27. Phillips J, Lam C, Palmisano L. Analysis of the accuracy and readability of herbal supplement information on Wikipedia. J Am Pharm Assoc (2003). 2014;54(4):406-14. doi: 10.1331/JAPhA.2014.13181.
28. Polepalli Ramesh B, Houston T, Brandt C, Fang H, Yu H. Improving patients' electronic health record comprehension with NoteAid. Stud Health Technol Inform. 2013;192:714-8. doi: 10.3233/978-1-61499-289-9-714
29. Punia V, Dagar A, Agarwal N, He W, Hillen M. Comparison of neurological healthcare oriented educational resources for patients on the internet. J Clin Neurosci. doi: 10.1016/j.jocn.2014.05.043. 2014;21(12):2179-83.
30. Rajagopalan MS, Khanna VK, Leiter Y, Stott M, Showalter TN, Dicker AP, et al. Patient-oriented cancer information on the internet: a comparison of wikipedia and a professionally maintained database. J Oncol Pract. 2011;7(5):319-23. doi: 10.1200/JOP.2010.000209.
31. Reavley NJ, Mackinnon AJ, Morgan AJ, Alvarez-Jimenez M, Hetrick SE, Killackey E, et al. Quality of information sources about mental disorders: a comparison of Wikipedia with centrally controlled web and printed sources. Psychol Med. 2012;42(8):1753-62. doi: 10.1017/S003329171100287X.
32. Seth AK, Vargas CR, Chuang DJ, Lee BT. Readability Assessment of Patient Information about Lymphedema and Its Treatment. Plast Reconstr Surg. 2016;137(2):287e-95e. doi: 10.1097/01.prs.0000475747.95096.ab.
33. Shetty KR, Wang RY, Shetty A, Levi J, Aaronson NL. Quality of Patient Education Sections on Otitis Media Across Different Website Platforms. Ann Otol Rhinol Laryngol. 2020;129(6):591-8. doi: 10.1177/0003489420902183.
34. Suwannakhan A, Casanova-Martínez D, Yurasakpong L, Montriwat P, Meemon K, Limpanuparb T. The Quality and Readability of English Wikipedia Anatomy Articles. Anat Sci Educ. 2020;13(4):475-87. doi: 10.1002/ase.1910.
35. Thomas GR, Eng L, de Wolff JF, Grover SC. An evaluation of Wikipedia as a resource for patient education in nephrology. Semin Dial. 2013;26(2):159-63. doi: 10.1111/sdi.12059.

36. Tulbert BH, Snyder CW, Brodell RT. Readability of Patient-oriented Online Dermatology Resources. J Clin Aesthet Dermatol. 2011;4(3):27-33. PMID: 21464884.
37. Volsky PG, Baldassari CM, Mushti S, Derkay CS. Quality of Internet information in pediatric otolaryngology: a comparison of three most referenced websites. Int J Pediatr Otorhinolaryngol. 2012;76(9):1312-6. doi: 10.1016/j.ijporl.2012.05.026.
38. Watad A, Bragazzi NL, Brigo F, Sharif K, Amital H, McGonagle D, et al. Readability of Wikipedia Pages on Autoimmune Disorders: Systematic Quantitative Assessment. J Med Internet Res. 2017;19(7):e260. doi: 10.2196/jmir.8225.
39. Yacob M, Lotfi S, Tang S, Jetty P. Wikipedia in Vascular Surgery Medical Education: Comparative Study. JMIR Med Educ. 2020;6(1):e18076. doi: 10.2196/18076.
40. Yasseri T, Kornai A, Kertész J. A practical approach to language complexity: a Wikipedia case study. PLoS One. 2012;7(11):e48386. doi: 10.1371/journal.pone.0048386.
41. Zheng J, Yu H. Readability Formulas and User Perceptions of Electronic Health Records Difficulty: A Corpus Study. J Med Internet Res. 2017;19(3):e59. doi: 10.2196/jmir.6962.
42. Zheng J, Yu H. Assessing the Readability of Medical Documents: A Ranking Approach. JMIR Med Inform. 2018;6(1):e17. doi: 10.2196/medinform.8611.
43. Liu Y, Medlar A, Glowacka D. Can Language Models Identify Wikipedia Articles with Readability and Style Issues? Proceedings of the ACM SIGIR International Conference on Theory of Information Retrieval; Virtual Event, Canada: Association for Computing Machinery; 2021. p. 113–7. doi: 10.1145/3471158.3472234.
44. Jatowt A, Tanaka K. Is wikipedia too difficult? comparative analysis of readability of wikipedia, simple wikipedia and britannica. Proceedings of the 21st ACM international conference on Information and knowledge management; Maui, Hawaii, USA: Association for Computing Machinery; 2012. p. 2607–10. doi: 10.1145/2396761.2398703.
45. Schedl M. Genre Differences of Song Lyrics and Artist Wikis: An Analysis of Popularity, Length, Repetitiveness, and Readability. Proceedings of the World Wide Web Conference; San Francisco, CA, USA: Association for Computing Machinery; 2019. p. 3201–7. doi: 10.1145/3308558.3313604.
46. Antunes H, Lopes CT. Proposal and Comparison of Health Specific Features for the Automatic Assessment of Readability. Proceedings of the 43rd International ACM SIGIR Conference on Research and Development in Information Retrieval: Association for Computing Machinery; 2020. p. 1973–6. doi: 10.1145/3397271.3401187.
47. Piccardi T, Catasta M, Zia L, West R. Structuring Wikipedia Articles with Section Recommendations. Proceedings of the 41st International ACM SIGIR Conference on Research & Development in Information Retrieval: Association for Computing Machinery; 2018. p. 665–74. doi: 10.1145/3209978.3209984.
48. Yuncong C, Fung P. Unsupervised synthesis of multilingual Wikipedia articles. Proceedings of the 23rd International Conference on Computational Linguistics; Beijing, China: Association for Computational Linguistics; 2010. p. 197–205.
49. Shirakawa M, Nakayama K, Hara T, Nishio S. Concept vector extraction from Wikipedia category network. Proceedings of the 3rd International Conference on Ubiquitous Information Management and Communication; Suwon, Korea: Association for Computing Machinery; 2009. p. 71–9. doi: 10.1145/1516241.1516255.
50. Rello L, Pielot M, Marcos M-C. Make It Big! The Effect of Font Size and Line Spacing on Online Readability. Proceedings of the CHI Conference on Human Factors in Computing Systems: Association for Computing Machinery; 2016. p. 3637–48. doi: 10.1145/2858036.2858204.
51. Rello L, Pielot M, Marcos M-C, Carlini R. Size matters (spacing not): 18 points for a dyslexic-friendly Wikipedia. Proceedings of the 10th International Cross-Disciplinary Conference on Web Accessibility: Association for Computing Machinery; 2013. p. Article 17. doi: 10.1145/2461121.2461125.

52. Samoilenko A, Lemmerich F, Zens M, Jadidi M, Génois M, Strohmaier M. (Don't) Mention the War: A Comparison of Wikipedia and Britannica Articles on National Histories. Proceedings of the 2018 World Wide Web Conference; Lyon, France: International World Wide Web Conferences Steering Committee; 2018. p. 843–52. doi: 10.1145/3178876.3186132.
53. Sarjant S, Legg C, Robinson M, Medelyan O. "All You Can Eat" Ontology-Building: Feeding Wikipedia to Cyc. Proceedings of the IEEE/WIC/ACM International Joint Conference on Web Intelligence and Intelligent Agent Technology - Volume 01: IEEE Computer Society; 2009. p. 341–8. doi: 10.1109/WI-IAT.2009.60.
54. Zhu Z, Bernhard D, Gurevych I. A monolingual tree-based translation model for sentence simplification. Proceedings of the 23rd International Conference on Computational Linguistics; Beijing, China: Association for Computational Linguistics; 2010. p. 1353–61.
55. Nakatani M, Jatowt A, Tanaka K. Easiest-first search: towards comprehension-based web search. Proceedings of the 18th ACM conference on Information and knowledge management; Hong Kong, China: Association for Computing Machinery; 2009. p. 2057–60. doi: 10.1145/1645953.1646300.
56. Wubben S, Bosch Avd, Krahmer E. Sentence simplification by monolingual machine translation. Proceedings of the 50th Annual Meeting of the Association for Computational Linguistics: Long Papers - Volume 1; Jeju Island, Korea: Association for Computational Linguistics; 2012. p. 1015–24.
57. Shen A, Salehi B, Baldwin T, Qi J. A joint model for multimodal document quality assessment. Proceedings of the 18th Joint Conference on Digital Libraries: IEEE Press; 2019. p. 107–10. doi: 10.1109/JCDL.2019.00024.
58. Filippova K, Strube M. Sentence fusion via dependency graph compression. Proceedings of the Conference on Empirical Methods in Natural Language Processing; Honolulu, Hawaii: Association for Computational Linguistics; 2008. p. 177–85.
59. Gaio L, Besten Md, Rossi A, Dalle J-M. Wikibugs: using template messages in open content collections. Proceedings of the 5th International Symposium on Wikis and Open Collaboration; Orlando, Florida: Association for Computing Machinery; 2009. p. Article 14.
60. Nakatani M, Jatowt A, Tanaka K. Adaptive ranking of search results by considering user's comprehension. Proceedings of the 4th International Conference on Uniqitous Information Management and Communication; Suwon, Republic of Korea: Association for Computing Machinery; 2010. p. Article 27. doi: 10.1145/2108616.2108649.
61. Sen P. Collective context-aware topic models for entity disambiguation. Proceedings of the 21st international conference on World Wide Web; Lyon, France: Association for Computing Machinery; 2012. p. 729–38. dDoi: 10.1145/2187836.2187935.
62. Li Y, Wang C, Han F, Han J, Roth D, Yan X. Mining evidences for named entity disambiguation. Proceedings of the 19th ACM SIGKDD international conference on Knowledge discovery and data mining; Chicago, Illinois, USA: Association for Computing Machinery; 2013. p. 1070–8. doi: 10.1145/2487575.2487681.
63. Wang Q, Cao Z, Xu J, Li H. Group matrix factorization for scalable topic modeling. Proceedings of the 35th international ACM SIGIR conference on Research and development in information retrieval; Portland, Oregon, USA: Association for Computing Machinery; 2012. p. 375–84. doi: 10.1145/2348283.2348335.
64. Aker A, Gaizauskas R. Generating image descriptions using dependency relational patterns. Proceedings of the 48th Annual Meeting of the Association for Computational Linguistics; Uppsala, Sweden: Association for Computational Linguistics; 2010. p. 1250–8.
65. Hecht B, Carton SH, Quaderi M, Schöning J, Raubal M, Gergle D, et al. Explanatory semantic relatedness and explicit spatialization for exploratory search. Proceedings of the 35th international ACM SIGIR conference on Research and development in information retrieval; Portland, Oregon, USA: Association for Computing Machinery; 2012. p. 415–24. doi: 10.1145/2348283.2348341.

66. Torres SD, Weber I, Hiemstra D. Analysis of Search and Browsing Behavior of Young Users on the Web. ACM Trans Web. 2014;8(2):Article 7. doi: 10.1145/2555595.
67. Weichselbraun A, Kuntschik P, Braşoveanu AMP. Mining and Leveraging Background Knowledge for Improving Named Entity Linking. Proceedings of the 8th International Conference on Web Intelligence, Mining and Semantics; Novi Sad, Serbia: Association for Computing Machinery; 2018. p. Article 27. doi: 10.1145/3227609.3227670.
68. Liu X, Duh K, Matsumoto Y. Multilingual Topic Models for Bilingual Dictionary Extraction. ACM Trans Asian Low-Resour Lang Inf Process. 2015;14(3):Article 11. doi: 10.1145/2699939.
69. Inkeaw P, Chaijaruwanich J, Soonthornthum B. Digital Library for Thai Astronomical History Study on French Document Resource. Proceedings of the 3rd International Conference on Digital Technology in Education; Yamanashi, Japan: Association for Computing Machinery; 2019. p. 246–53. doi: 10.1145/3369199.3369236.
70. Dang Q, Ignat C, Measuring Quality of Collaboratively Edited Documents: The Case of Wikipedia. Proceedings of the IEEE 2nd International Conference on Collaboration and Internet Computing (CIC); Pittsburgh, PA, USA: IEEE; 2016. p. 266–75, doi: 10.1109/CIC.2016.044.
71. Thruesen P, Čechák J, Sezñec B, Castaño R, Kanhabua N, To link or not to link: Ranking hyperlinks in Wikipedia using collective attention. Proceedings of the IEEE International Conference on Big Data (Big Data); Washington, DC, USA: IEEE; 2016. p. 1709–18, doi: 10.1109/BigData.2016.7840785.
72. Shen A, Salehi B, Baldwin T, Qi J. A Joint Model for Multimodal Document Quality Assessment. Proceedings of the 2019 ACM/IEEE Joint Conference on Digital Libraries (JCDL); Champaign, IL, USA: IEEE; 2019. p. 107-110, doi: 10.1109/JCDL.2019.00024.
73. Den Besten M, Gaio L, Rossi A, Dalle J-M, Using Metadata Signals to Support Stigmergy. Proceeding of the Fourth IEEE International Conference on Self-Adaptive and Self-Organizing Systems Workshop; Budapest, Hungary: IEEE; 2010. p. 131–5, doi: 10.1109/SASOW.2010.28.
