# Supplementary material for: Readability of English, German, and Russian Disease-Related Wikipedia Pages: Automated Computational Analysis
Source: J Med Internet Res. 2022 May 16;24(5):e36835. doi: 10.2196/36835 (PMC9152717; doi:10.2196/36835)
Supplement: Multimedia Appendix 2 [file jmir_v24i5e36835_app2.pdf]

# Multimedia Appendix 2: General statistics for disease-related Wikipedia graphs

Table 1. Disease graph statistics for each language (EN, DE, RU)

| Attribute | | | |
|---|---|---|---|
| Language | English | German | Russian |
| Main concept | Human diseases and disorders | Krankheit | Заболевания человека |
| Node count | 92,917 | 22,859 | 8,057 |
| Edge count | 207,026 | 51,778 | 24,374 |
| Generation date | 17 Nov 2021 | 13 Oct 2021 | 16 Nov 2021 |
| Wikipedia snapshot date | 03 Jul 2021 | 27 Jun 2021 | 27 Jun 2021 |
