# Supplementary material for: Readability of English, German, and Russian Disease-Related Wikipedia Pages: Automated Computational Analysis
Source: J Med Internet Res. 2022 May 16;24(5):e36835. doi: 10.2196/36835 (PMC9152717; doi:10.2196/36835)
Supplement: Multimedia Appendix 3 [file jmir_v24i5e36835_app3.pdf]

Multimedia Appendix 3:
Terms and Wildcards applied for Category Filtering

English language

Diseases and disorders in
Diseases and disorders by
disorders in
Diseases and disorders by country
health in
based in
based on
outbreaks
Outbreaks
Disability in
deaths
death
Deaths
creatures
dog
Dog
people
People
sportspeople
Sportspeople
Intersex men
women
children with
Children with
researchers
educators
Educators
activists
academics
amputees
Amputees
recipients
Recipients
writers
Writers
Artists
actors
Actors
works
Works

series
shows
films
Films
episodes
Television
Poems
Books
Novels
Songs
stories
literature
Literature
comics
Comics
characters
fiction
Fictional
treatment
Treatment
services
scales for
instruments
equipment
classifications
Accessible
rehabilitation
Rehabilitation
substances
procedures
drugs
by type
by year
by former country
by country
by nationality
organizations
Organizations
organisations
Organisations
Schools
facilities
hospitals
Hospitals
centers
Centers
products

surgery
Association
associations
journals
sports
Sports
parasports
Parasports
hockey
*media *
Media
theatre
Society
Order of
HIV/AIDS in
Cancer in
Leprosy in
Obesity in
Haemophilia in
Narcolepsy in
Tuberculosis in
lists
Lists
language
charities
Charities
franchise
games
amputations
Amputations
awards
animals
Animal
survivors
Survivors
fundraisers
Plays
musicals
Churches
Theories
software
users
History
consortium
studies
devices
technology

pandemic
manufacturers
programs
by continent
rights
royalty
Royalty
Accessibility
incidents
providers
Entertainers
awareness
International
affiliates
mutes
templates
robots
musicians
Members
members
culture
politics
epidemic
Disability stubs
Smoking
Sexual fetishism
Incest

German language

Demenz im Film
Epidemie im Film
Pädophilie im Film
Krankheitsbild in der Tiermedizin
Liste (Medizin)
Epidemie
Pädophilenbewegung
Grooming
Pädophilie im Internet
Pflanzenmykose

Russian language

Фильмы о психических заболеваниях
Умершие от сахарного диабета
Умершие от болезни почек
Умершие от болезни Альцгеймера
Умершие от сердечной недостаточности
Умершие от болезни печени

Умершие от эпилепсии
Умершие от деменции
СПИД-активисты
Слепые
Люди с ВИЧ/СПИДом
Движение по отрицанию ВИЧ/СПИДа
Исследователи ВИЧ/СПИДа
Аборт
Антидепрессанты
Лекарства от болезни Альцгеймера
Средства для лечения ВИЧ
Люди с редкими заболеваниями
Средства реабилитации для людей с нарушением зрения
Произведения о зависимости
Персонификация безумия
Саванты
Стерилизация (размножение)
Люди с сахарным диабетом 1 типа
Лечение зависимости от психоактивных веществ
Люди со спинальной мышечной атрофией
Люди с сахарным диабетом 2 типа
Акромегалия
