# Supplementary material for: Readability of English, German, and Russian Disease-Related Wikipedia Pages: Automated Computational Analysis
Source: J Med Internet Res. 2022 May 16;24(5):e36835. doi: 10.2196/36835 (PMC9152717; doi:10.2196/36835)
Supplement: Multimedia Appendix 4 [file jmir_v24i5e36835_app4.pdf]

Multimedia Appendix 4: Distributions of all computed readability metrics in English

Group A

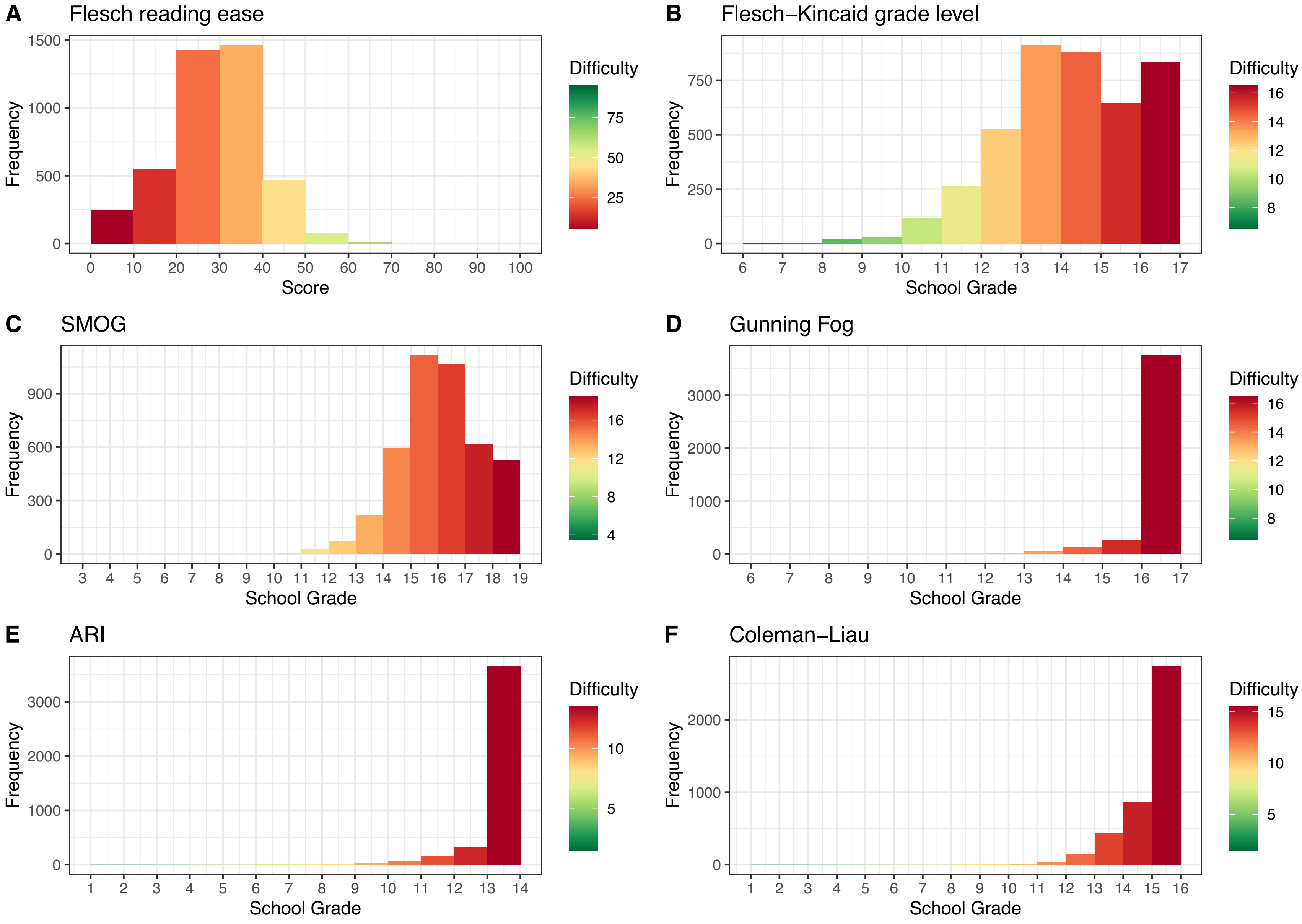
A
Flesch reading ease
Frequency
Score
Difficulty
B
Flesch−Kincaid grade level
School Grade
C
SMOG
D
Gunning Fog
E
ARI
F
Coleman−Liau

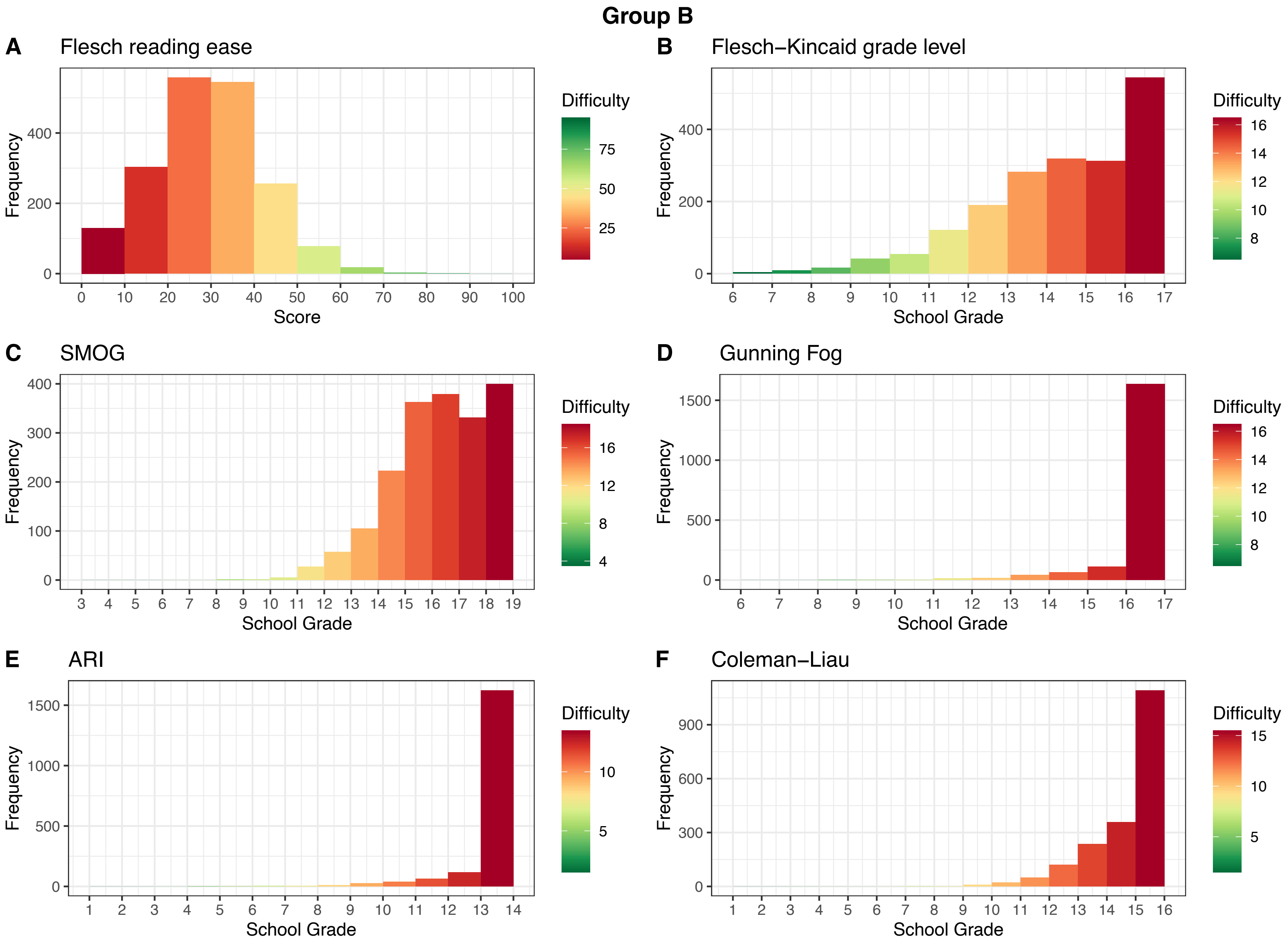

Group B
A
Flesch reading ease
Frequency
Score
Difficulty
B
Flesch−Kincaid grade level
School Grade
C
SMOG
D
Gunning Fog
E
ARI
F
Coleman−Liau

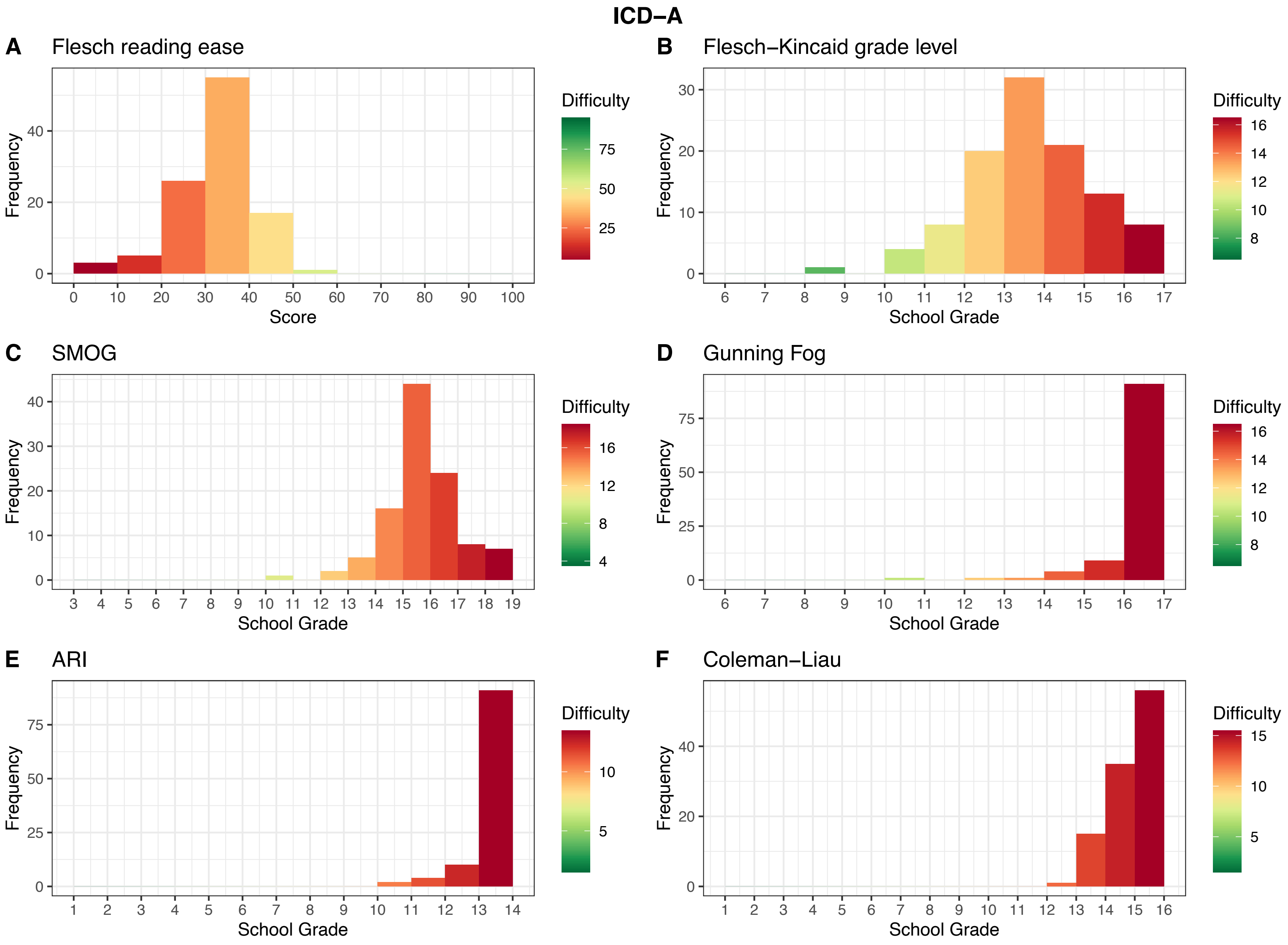
ICD–A
A Flesch reading ease
Frequency
Score
Difficulty
B Flesch–Kincaid grade level
School Grade
C SMOG
D Gunning Fog
E ARI
F Coleman–Liau

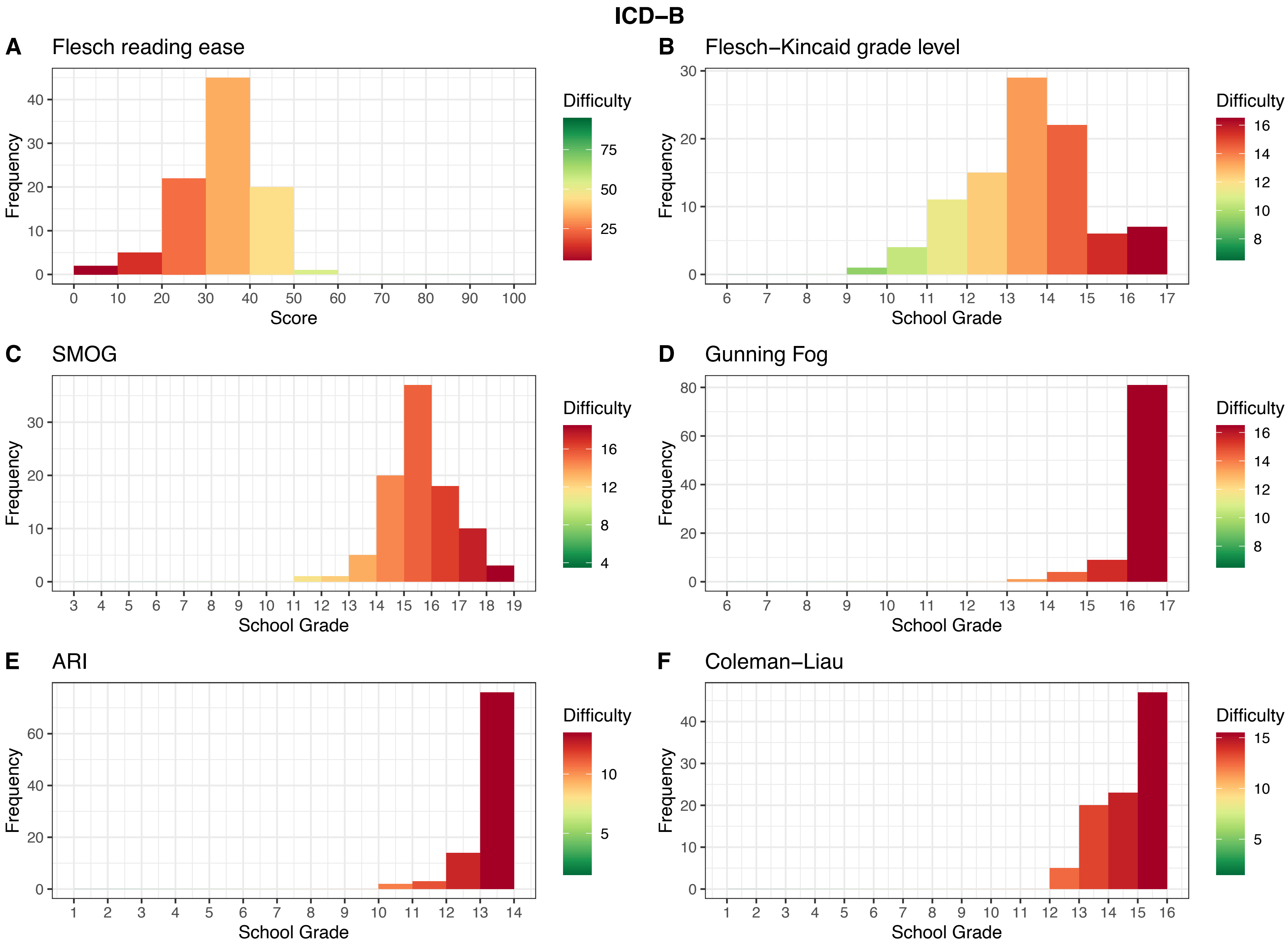
ICD−B
A
Flesch reading ease
Frequency
Score
Difficulty
B
Flesch−Kincaid grade level
Frequency
School Grade
Difficulty
C
SMOG
Frequency
School Grade
Difficulty
D
Gunning Fog
Frequency
School Grade
Difficulty
E
ARI
Frequency
School Grade
Difficulty
F
Coleman−Liau
Frequency
School Grade
Difficulty

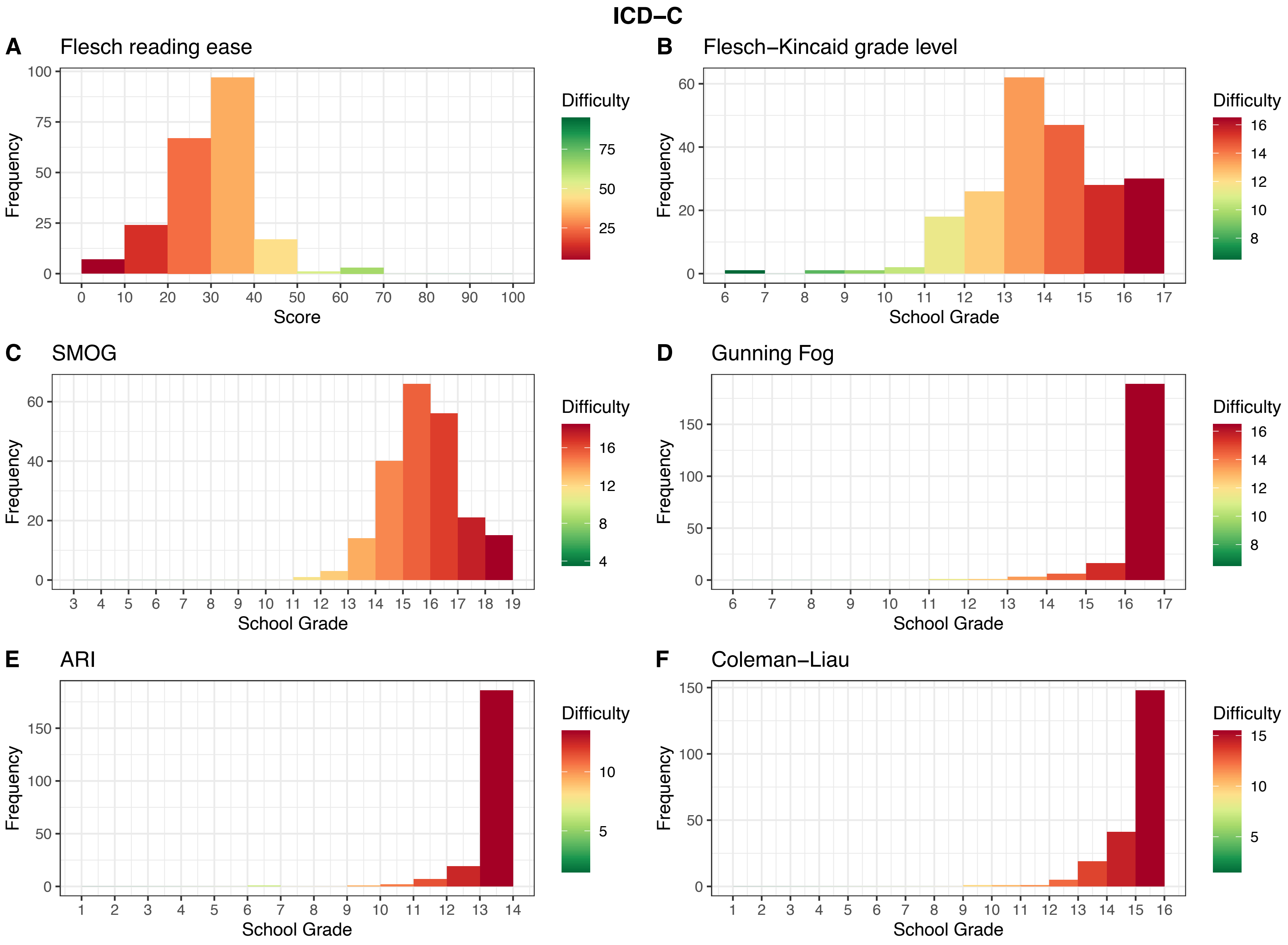
ICD–C
A Flesch reading ease
Frequency
Score
Difficulty
B Flesch–Kincaid grade level
School Grade
C SMOG
D Gunning Fog
E ARI
F Coleman–Liau

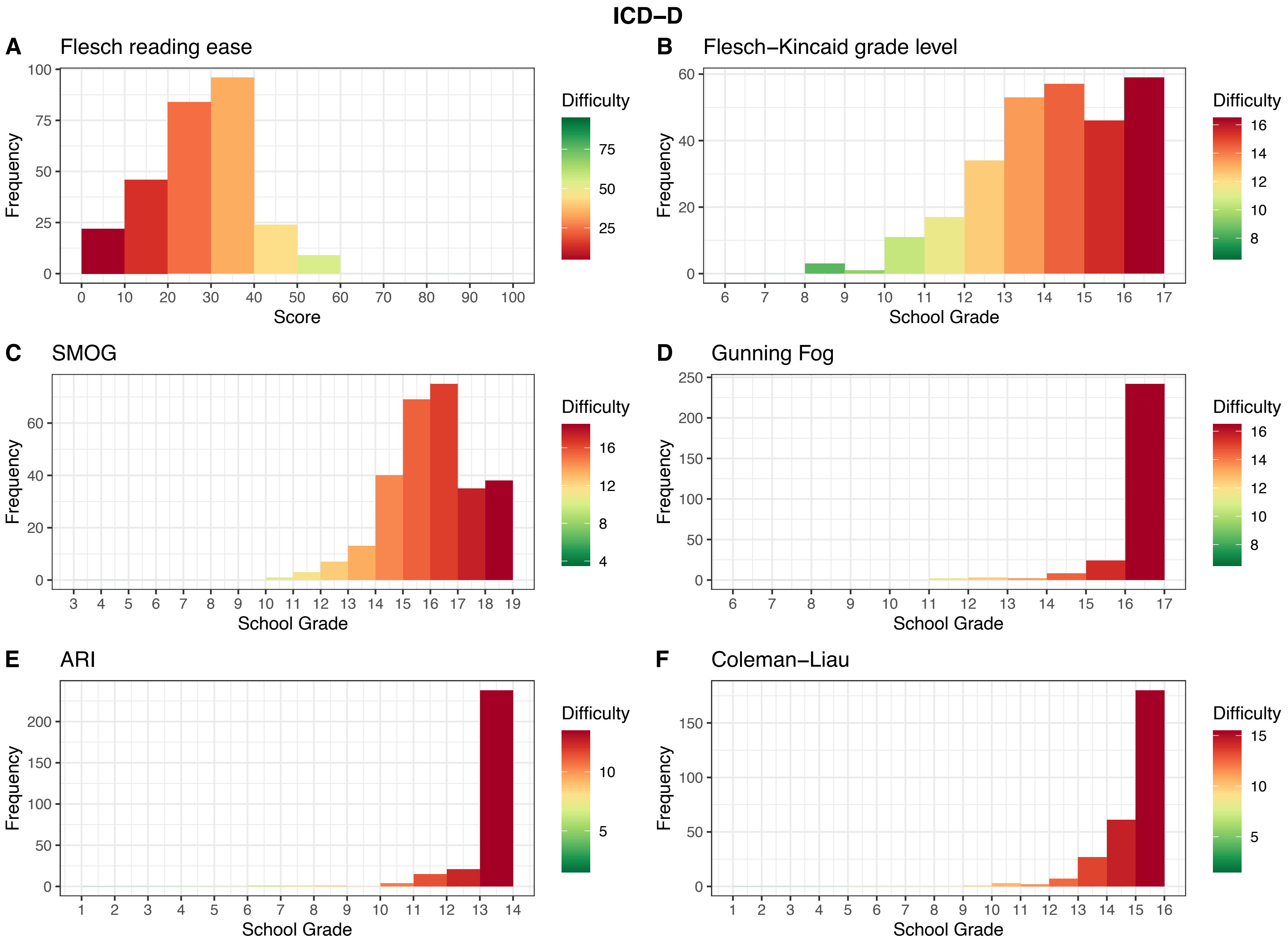

ICD–D
A
Flesch reading ease
Frequency
Score
Difficulty
B
Flesch–Kincaid grade level
Frequency
School Grade
Difficulty
C
SMOG
Frequency
School Grade
Difficulty
D
Gunning Fog
Frequency
School Grade
Difficulty
E
ARI
Frequency
School Grade
Difficulty
F
Coleman–Liau
Frequency
School Grade
Difficulty

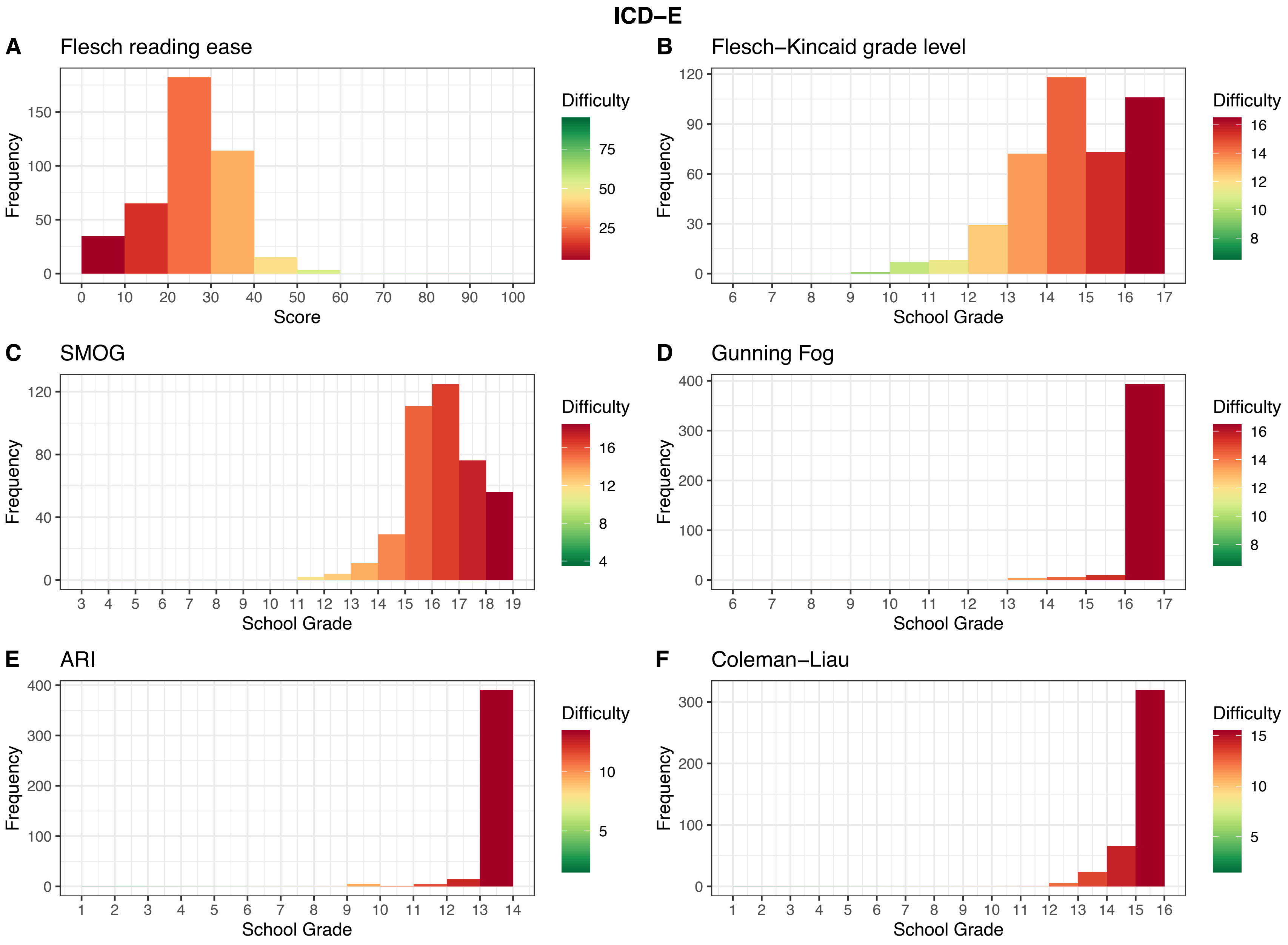
ICD−E
A
Flesch reading ease
Frequency
Score
Difficulty
75
50
25
B
Flesch−Kincaid grade level
School Grade
16
14
12
10
8
C
SMOG
D
Gunning Fog
E
ARI
F
Coleman−Liau

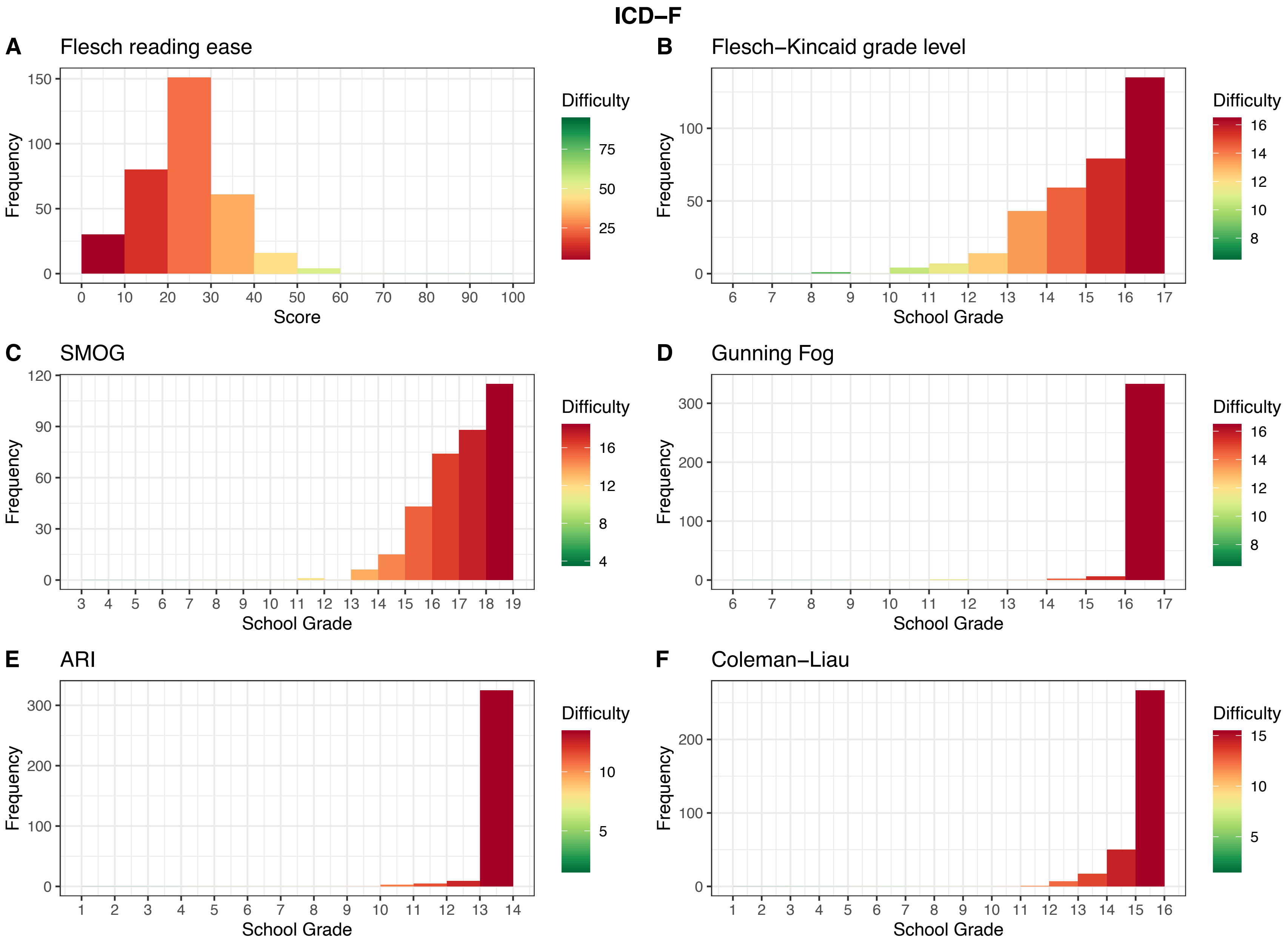
ICD–F
A
Flesch reading ease
Frequency
Score
Difficulty
75
50
25
B
Flesch–Kincaid grade level
School Grade
16
14
12
10
8
C
SMOG
D
Gunning Fog
E
ARI
F
Coleman–Liau

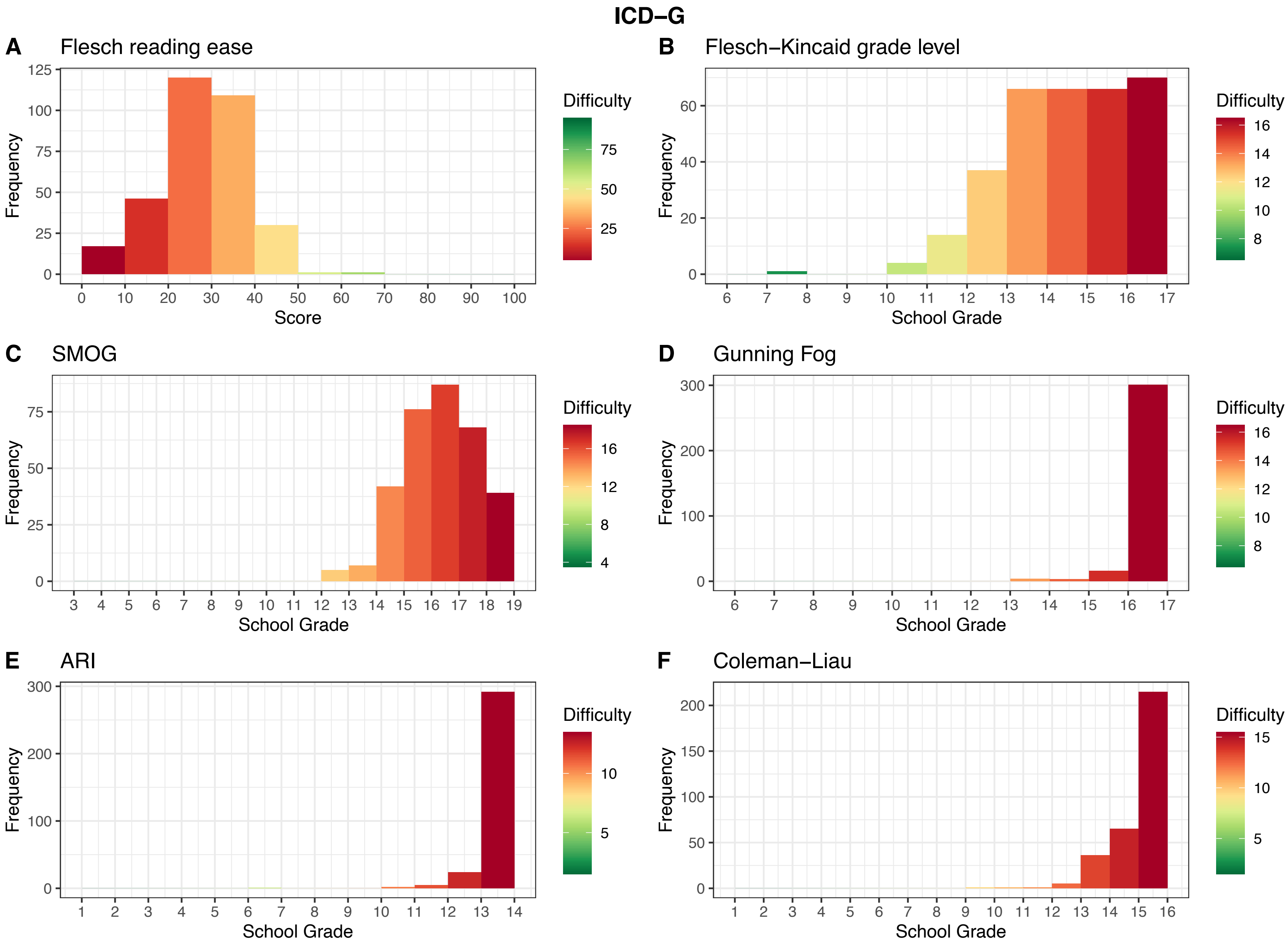
ICD–G
A
Flesch reading ease
Frequency
Score
Difficulty
B
Flesch–Kincaid grade level
School Grade
C
SMOG
D
Gunning Fog
E
ARI
F
Coleman–Liau

ICD–H

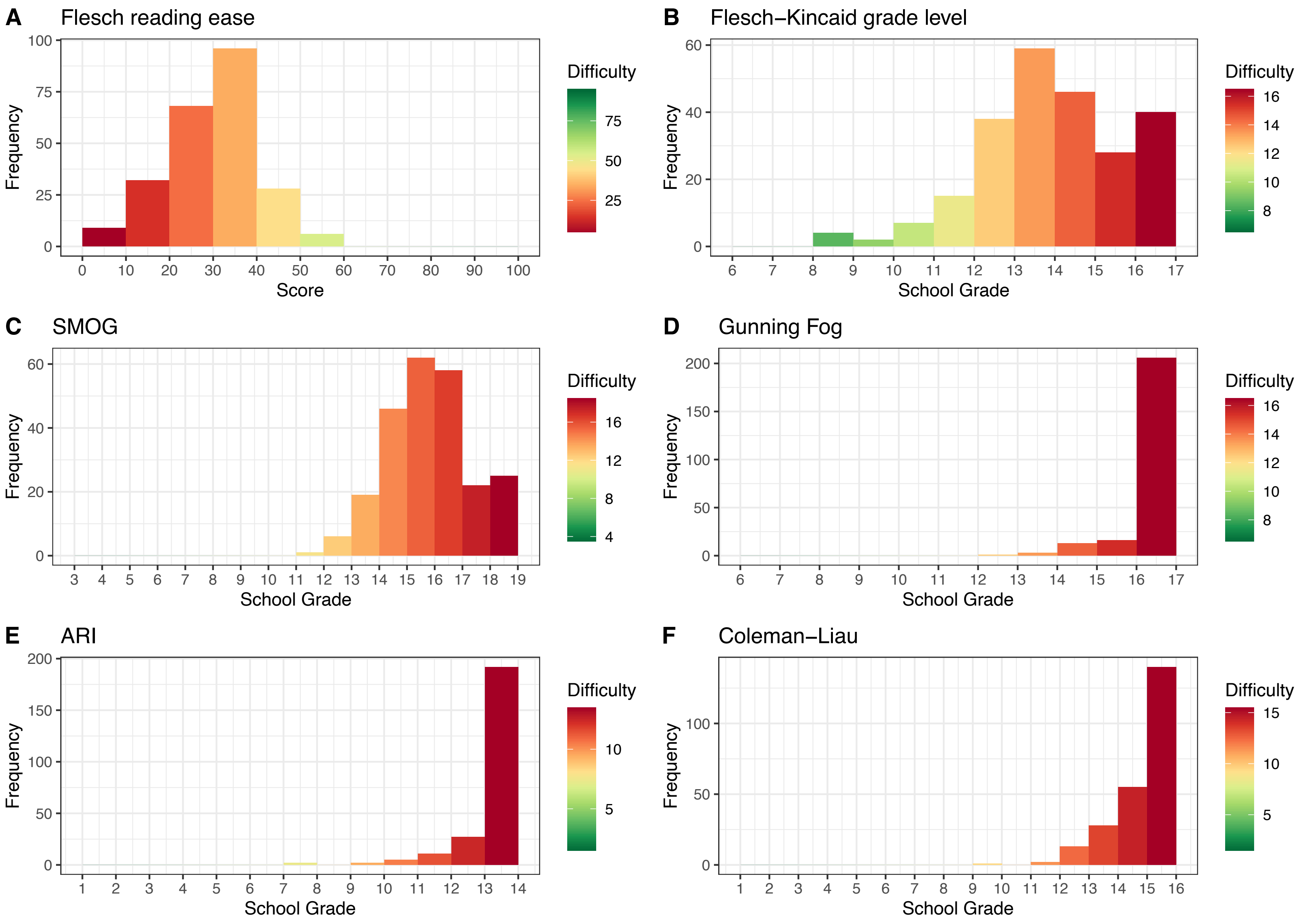
A
Flesch reading ease
Frequency
Score
Difficulty
75
50
25
B
Flesch–Kincaid grade level
Frequency
School Grade
Difficulty
C
SMOG
Frequency
School Grade
Difficulty
D
Gunning Fog
Frequency
School Grade
Difficulty
E
ARI
Frequency
School Grade
Difficulty
F
Coleman–Liau
Frequency
School Grade
Difficulty

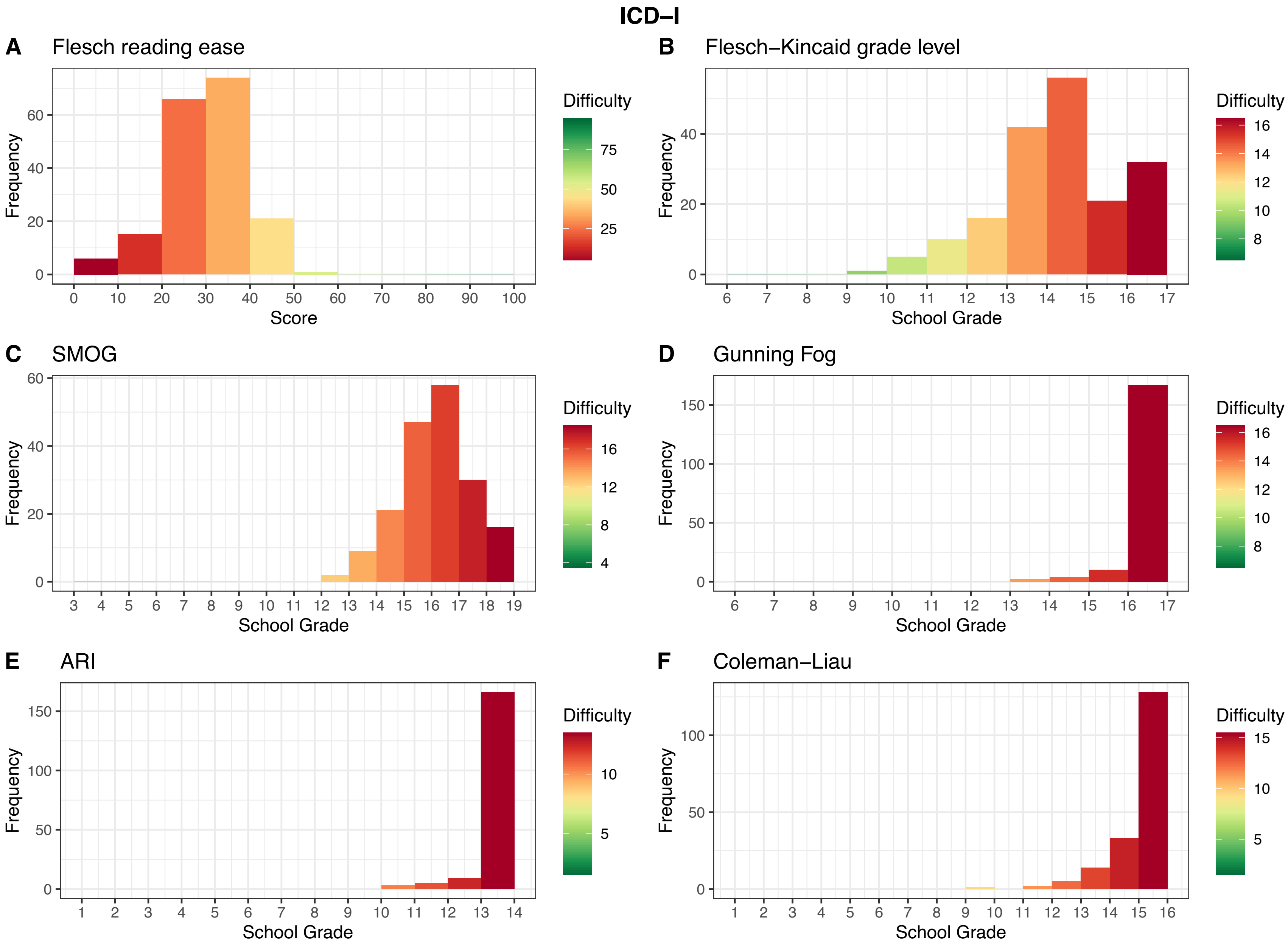

ICD–I
A Flesch reading ease
Frequency
Score
Difficulty
B Flesch–Kincaid grade level
School Grade
C SMOG
D Gunning Fog
E ARI
F Coleman–Liau

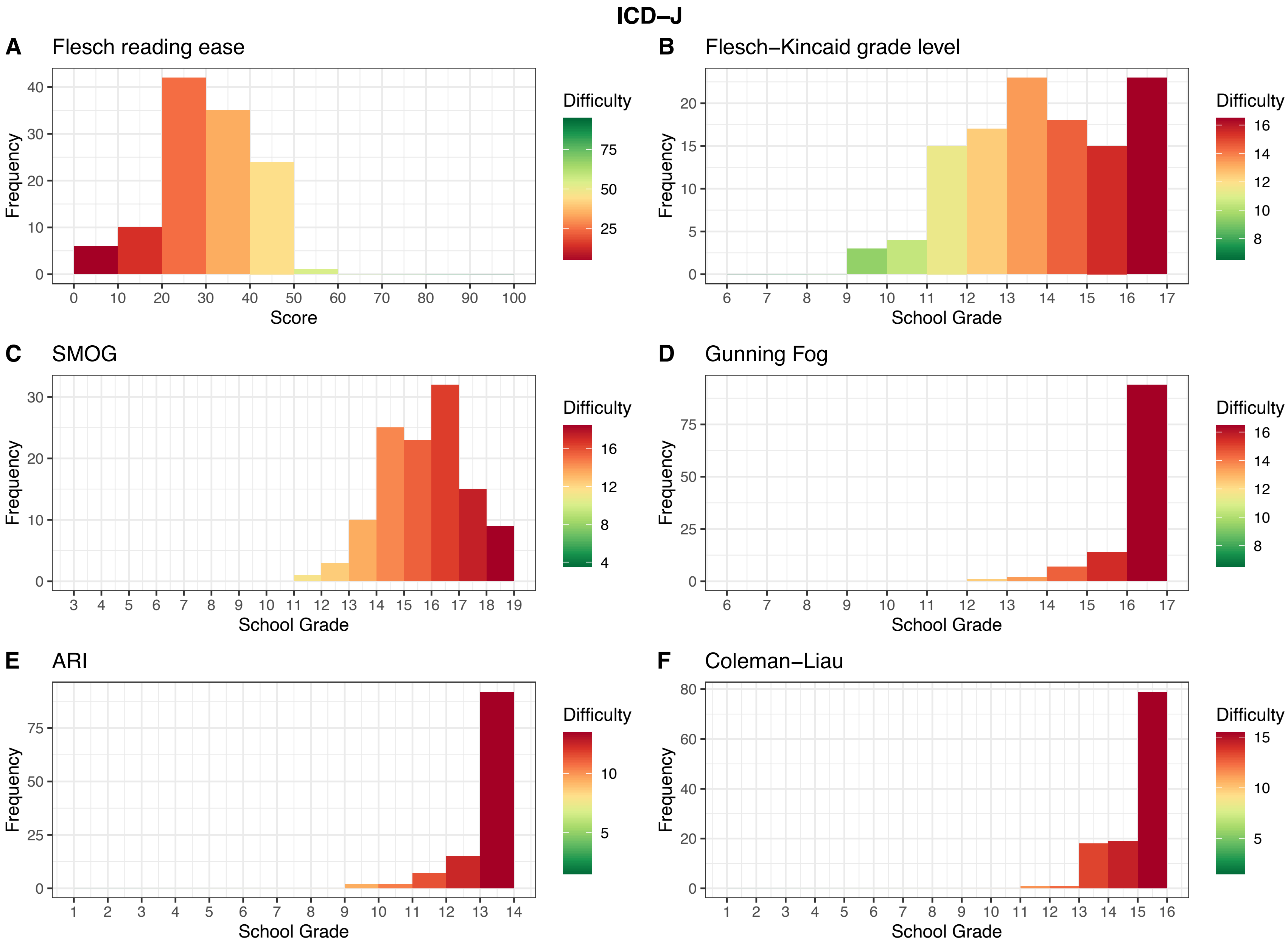

ICD–J
A Flesch reading ease
Frequency
Score
Difficulty
75
50
25
B Flesch–Kincaid grade level
School Grade
C SMOG
D Gunning Fog
E ARI
F Coleman–Liau

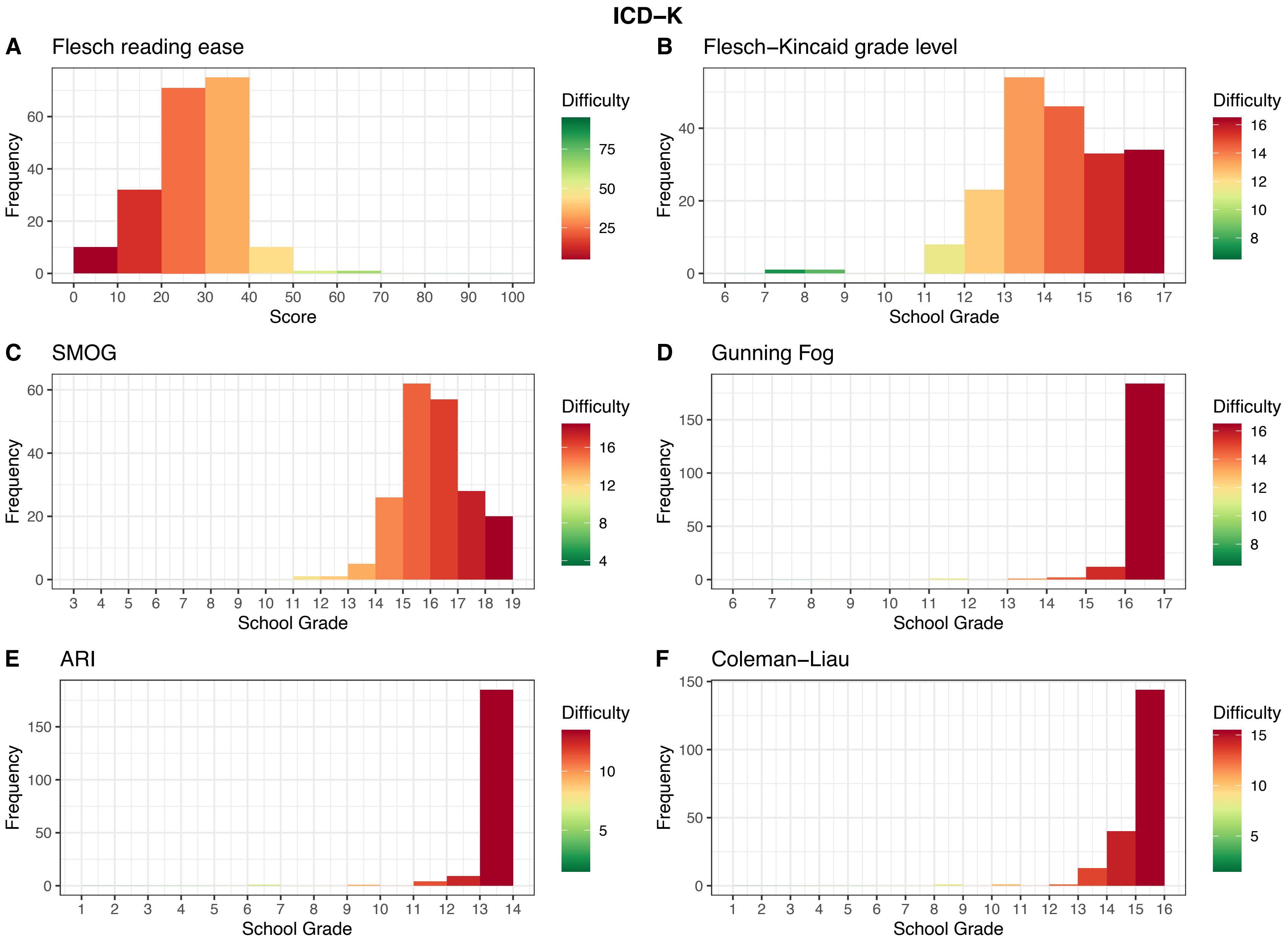
ICD–K
A
Flesch reading ease
Frequency
Score
0 10 20 30 40 50 60 70 80 90 100
0 20 40 60
Difficulty
75 50 25
B
Flesch–Kincaid grade level
Frequency
School Grade
6 7 8 9 10 11 12 13 14 15 16 17
0 20 40
Difficulty
16 14 12 10 8
C
SMOG
Frequency
School Grade
3 4 5 6 7 8 9 10 11 12 13 14 15 16 17 18 19
0 20 40 60
Difficulty
16 12 8 4
D
Gunning Fog
Frequency
School Grade
6 7 8 9 10 11 12 13 14 15 16 17
0 50 100 150
Difficulty
16 14 12 10 8
E
ARI
Frequency
School Grade
1 2 3 4 5 6 7 8 9 10 11 12 13 14
0 50 100 150
Difficulty
10 5
F
Coleman–Liau
Frequency
School Grade
1 2 3 4 5 6 7 8 9 10 11 12 13 14 15 16
0 50 100 150
Difficulty
15 10 5

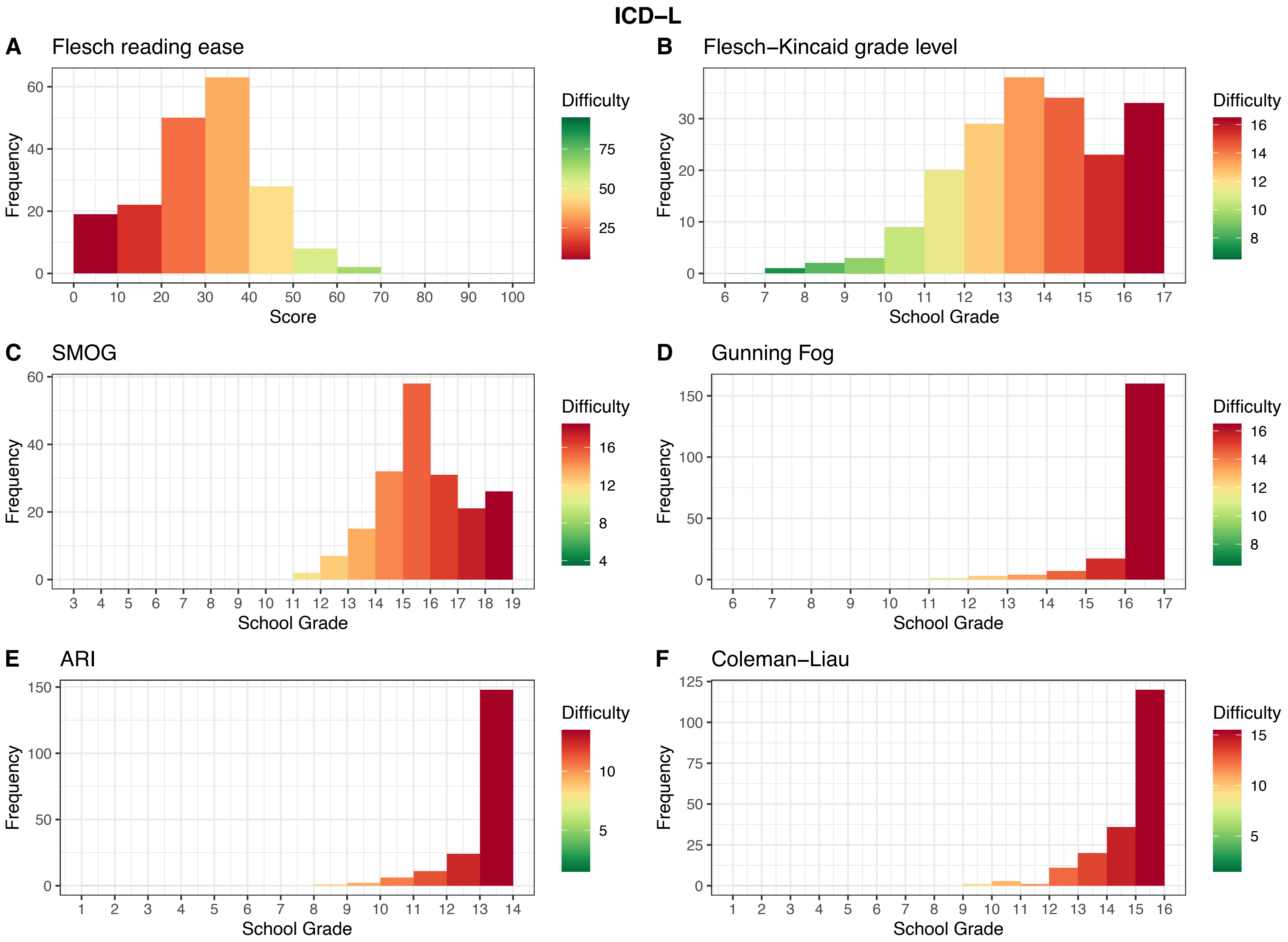
ICD–L
A Flesch reading ease
Frequency
Score
Difficulty
B Flesch–Kincaid grade level
School Grade
C SMOG
D Gunning Fog
E ARI
F Coleman–Liau

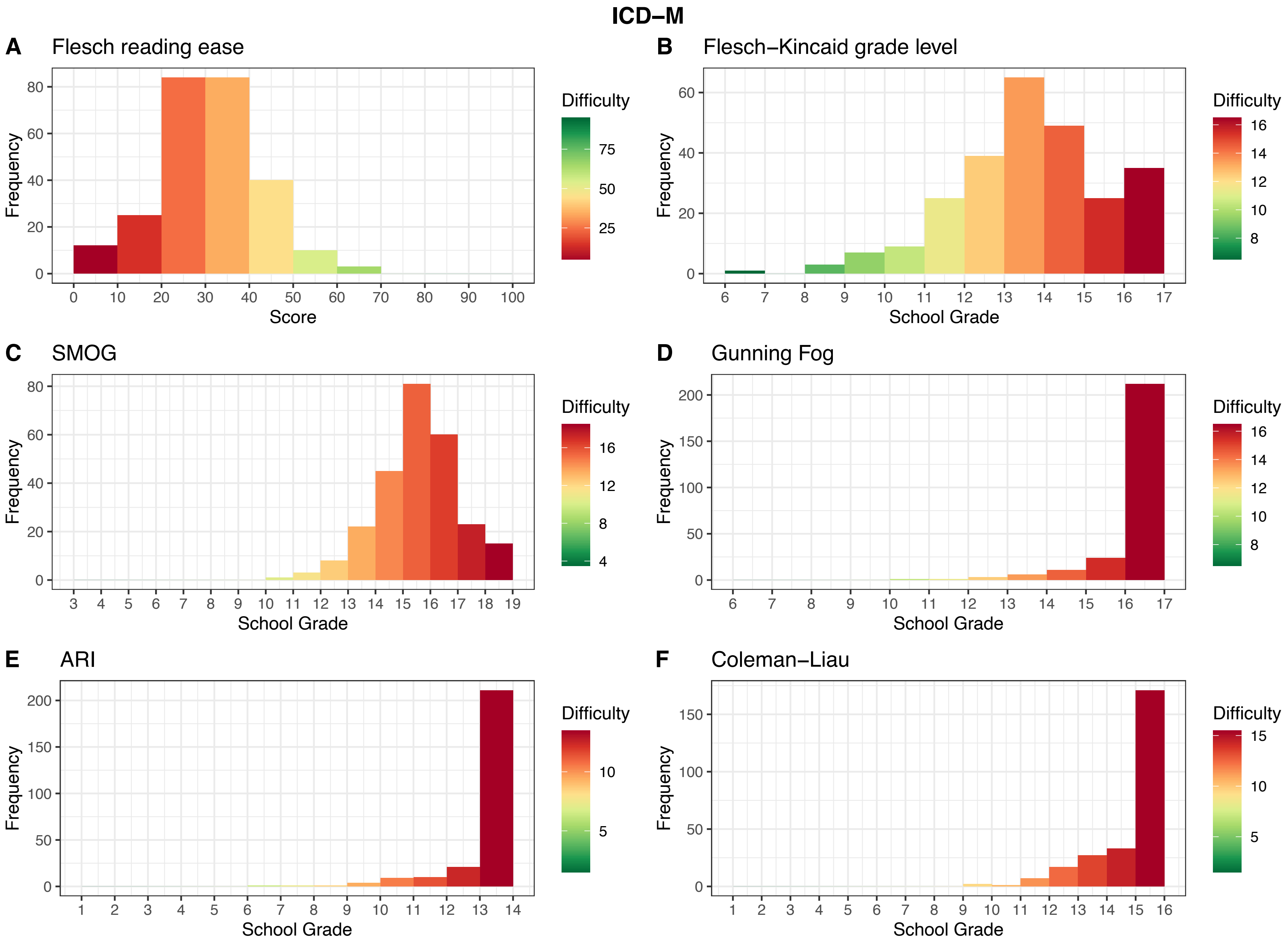

ICD–M
A
Flesch reading ease
Frequency
Score
Difficulty
B
Flesch–Kincaid grade level
Frequency
School Grade
Difficulty
C
SMOG
Frequency
School Grade
Difficulty
D
Gunning Fog
Frequency
School Grade
Difficulty
E
ARI
Frequency
School Grade
Difficulty
F
Coleman–Liau
Frequency
School Grade
Difficulty

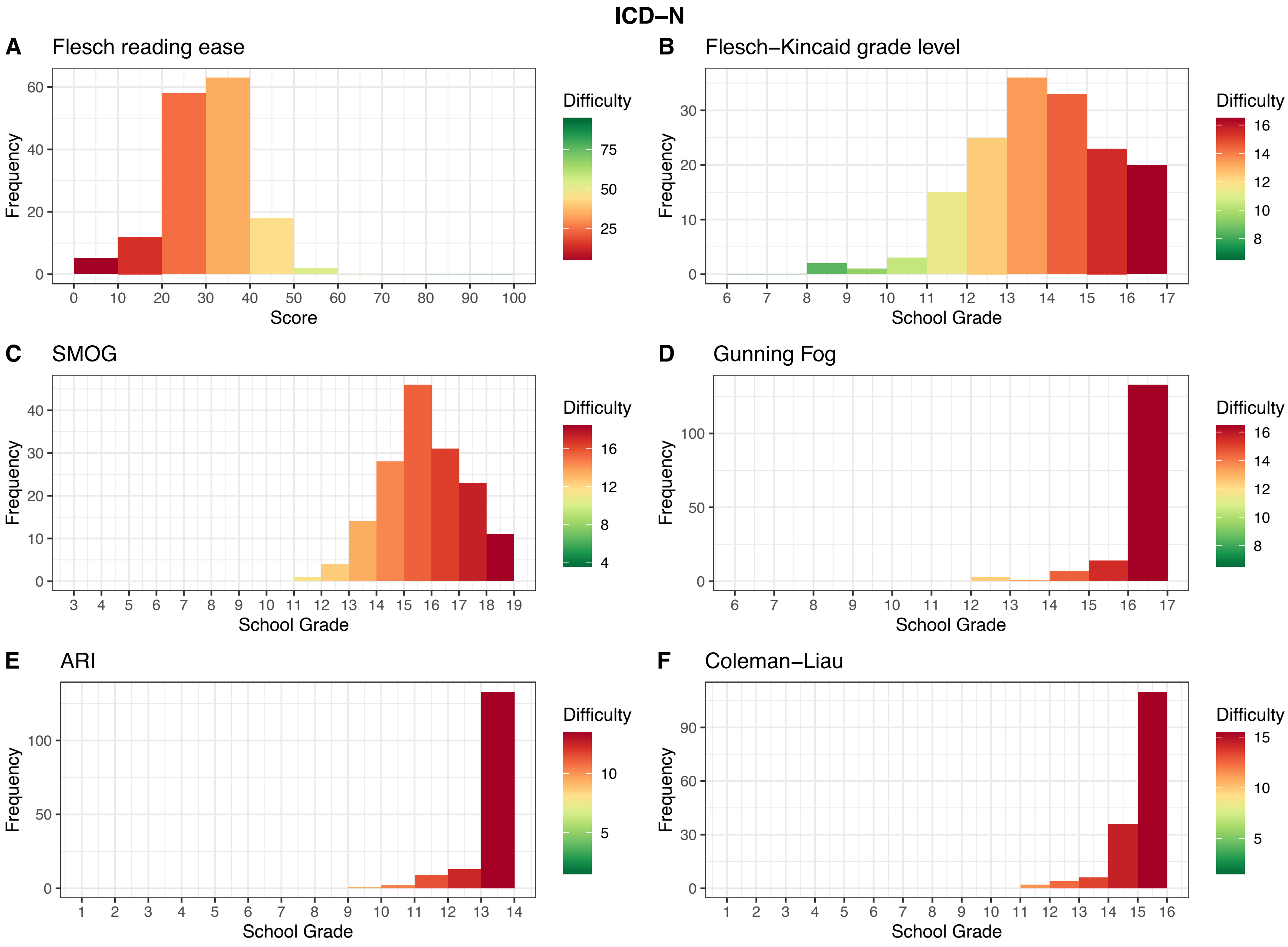
ICD–N
A Flesch reading ease
B Flesch–Kincaid grade level
C SMOG
D Gunning Fog
E ARI
F Coleman–Liau
Frequency
Score
School Grade
Difficulty

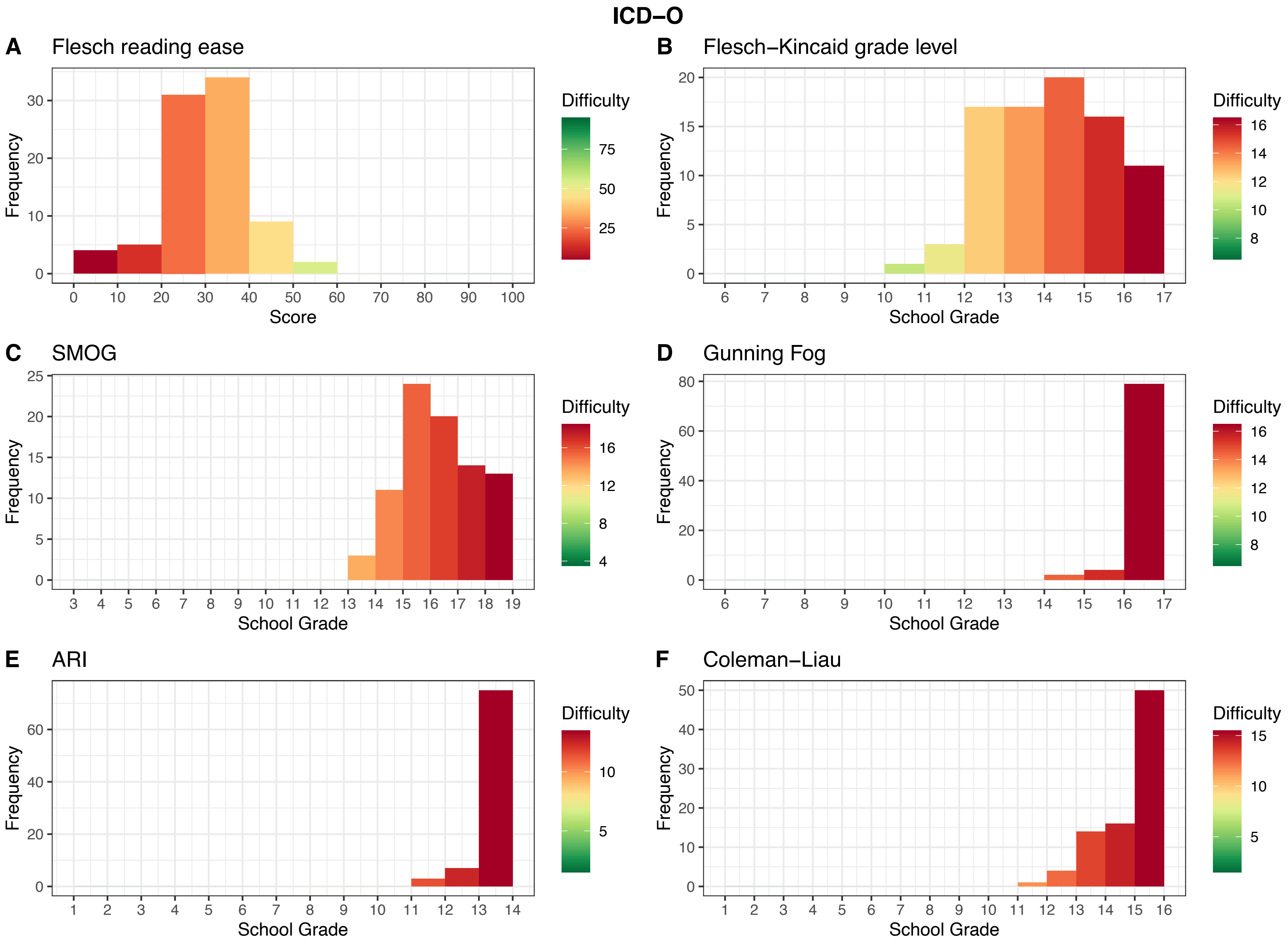
ICD–O
A
Flesch reading ease
Frequency
Score
0 10 20 30 40 50 60 70 80 90 100
0 10 20 30
Difficulty
75 50 25
B
Flesch–Kincaid grade level
Frequency
School Grade
6 7 8 9 10 11 12 13 14 15 16 17
0 5 10 15 20
Difficulty
16 14 12 10 8
C
SMOG
Frequency
School Grade
3 4 5 6 7 8 9 10 11 12 13 14 15 16 17 18 19
0 5 10 15 20 25
Difficulty
16 12 8 4
D
Gunning Fog
Frequency
School Grade
6 7 8 9 10 11 12 13 14 15 16 17
0 20 40 60 80
Difficulty
16 14 12 10 8
E
ARI
Frequency
School Grade
1 2 3 4 5 6 7 8 9 10 11 12 13 14
0 20 40 60
Difficulty
10 5
F
Coleman–Liau
Frequency
School Grade
1 2 3 4 5 6 7 8 9 10 11 12 13 14 15 16
0 10 20 30 40 50
Difficulty
15 10 5

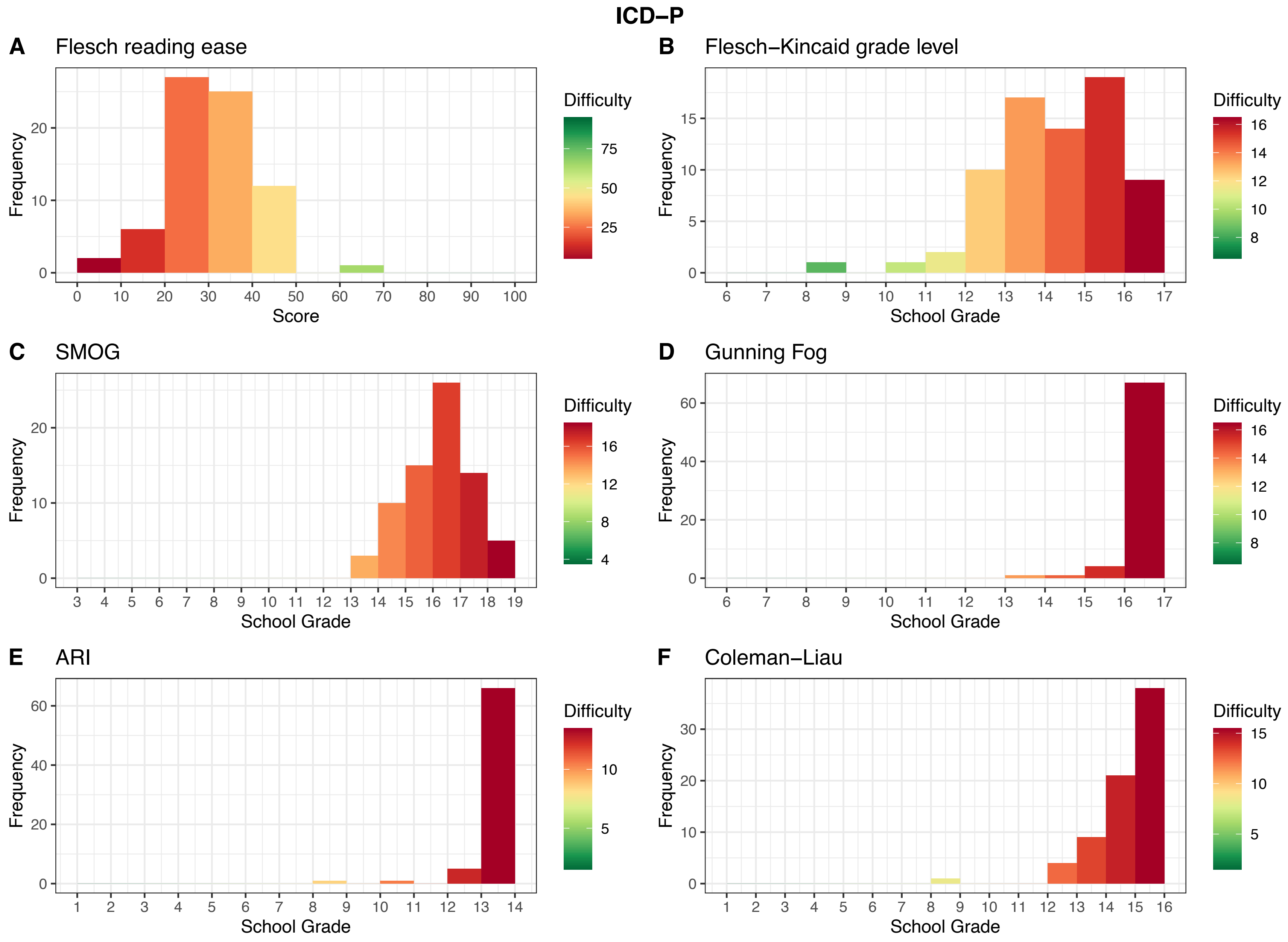
ICD–P
A
Flesch reading ease
Frequency
Score
Difficulty
B
Flesch–Kincaid grade level
School Grade
C
SMOG
D
Gunning Fog
E
ARI
F
Coleman–Liau

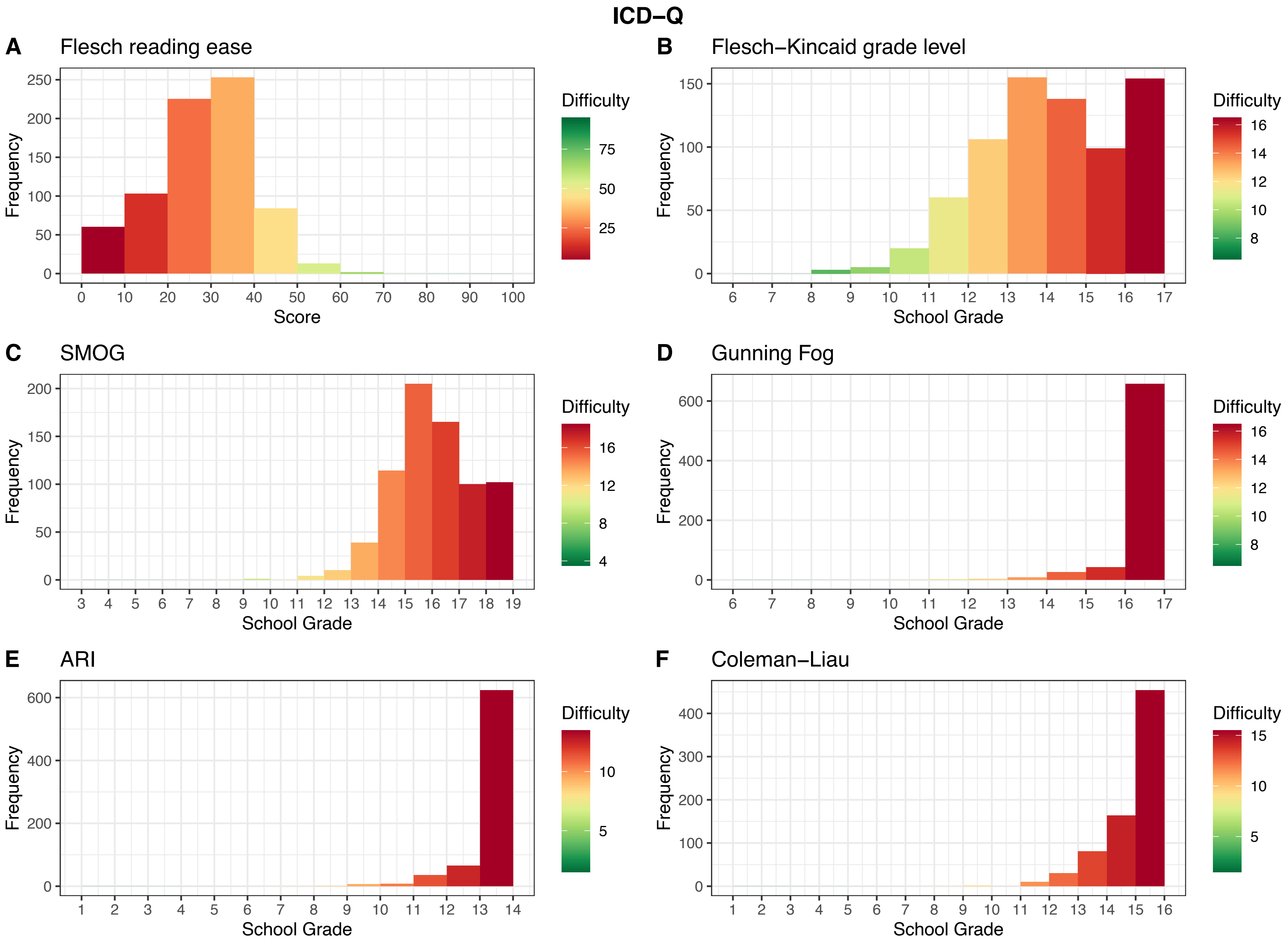

ICD–Q
A
Flesch reading ease
Frequency
Score
Difficulty
75
50
25
B
Flesch–Kincaid grade level
Frequency
School Grade
Difficulty
16
14
12
10
8
C
SMOG
Frequency
School Grade
Difficulty
16
12
8
4
D
Gunning Fog
Frequency
School Grade
Difficulty
16
14
12
10
8
E
ARI
Frequency
School Grade
Difficulty
10
5
F
Coleman–Liau
Frequency
School Grade
Difficulty
15
10
5

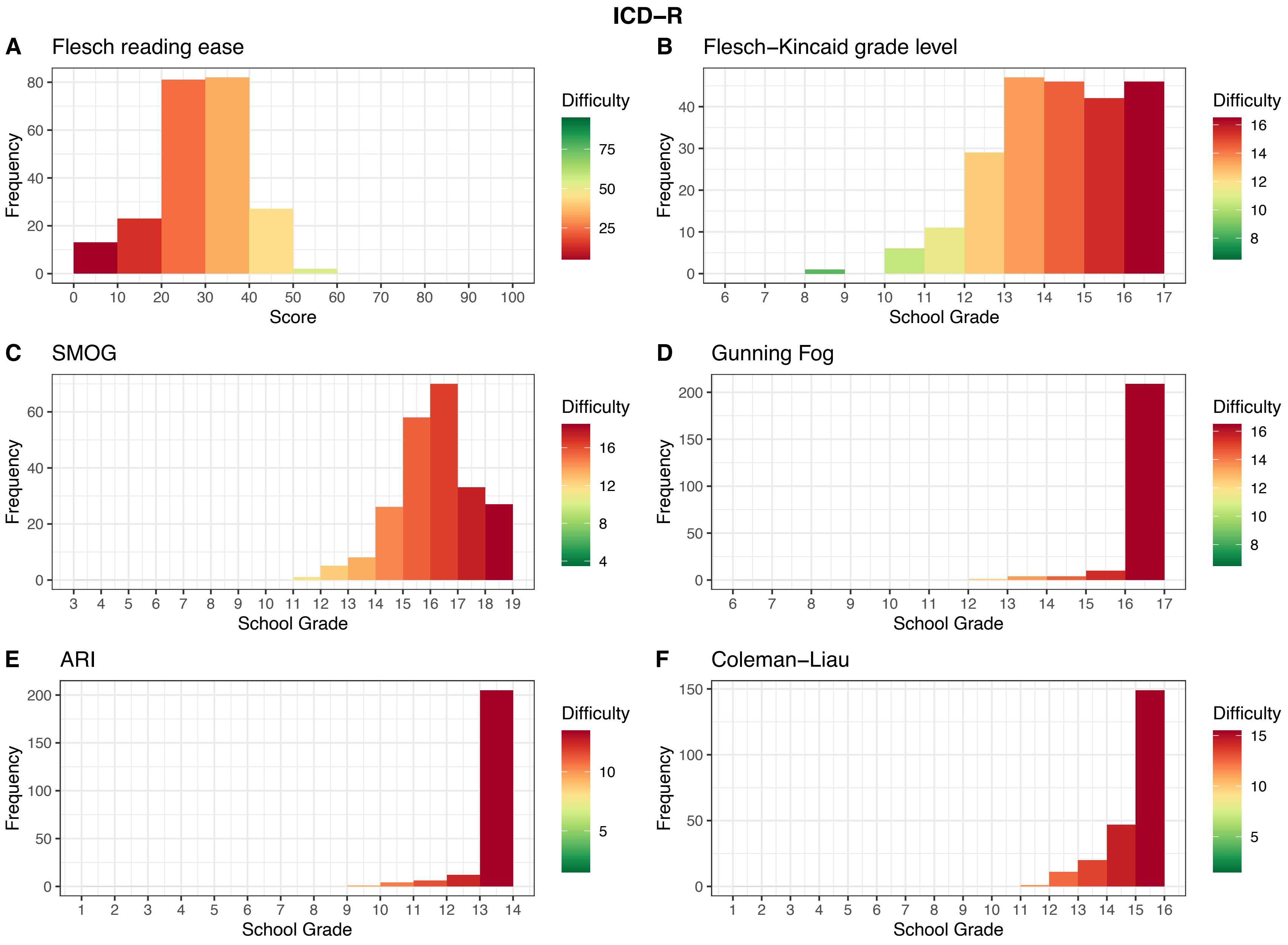
ICD–R
A Flesch reading ease
Frequency
Score
Difficulty
75
50
25
B Flesch–Kincaid grade level
Frequency
School Grade
Difficulty
16
14
12
10
8
C SMOG
Frequency
School Grade
Difficulty
16
12
8
4
D Gunning Fog
Frequency
School Grade
Difficulty
16
14
12
10
8
E ARI
Frequency
School Grade
Difficulty
10
5
F Coleman–Liau
Frequency
School Grade
Difficulty
15
10
5

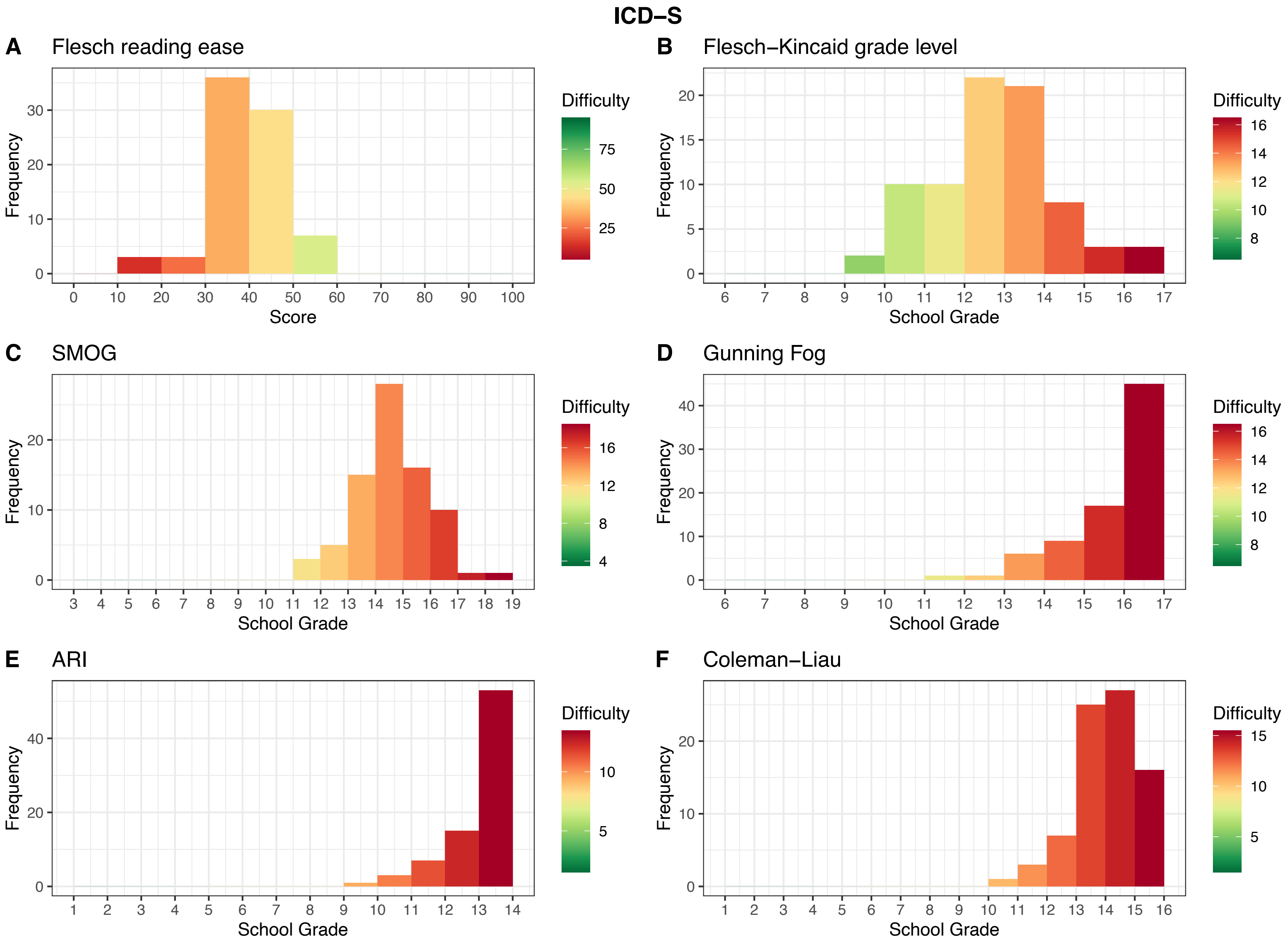
ICD–S
A
Flesch reading ease
Frequency
Score
Difficulty
75
50
25
B
Flesch–Kincaid grade level
Frequency
School Grade
Difficulty
16
14
12
10
8
C
SMOG
Frequency
School Grade
Difficulty
16
12
8
4
D
Gunning Fog
Frequency
School Grade
Difficulty
16
14
12
10
8
E
ARI
Frequency
School Grade
Difficulty
10
5
F
Coleman–Liau
Frequency
School Grade
Difficulty
15
10
5

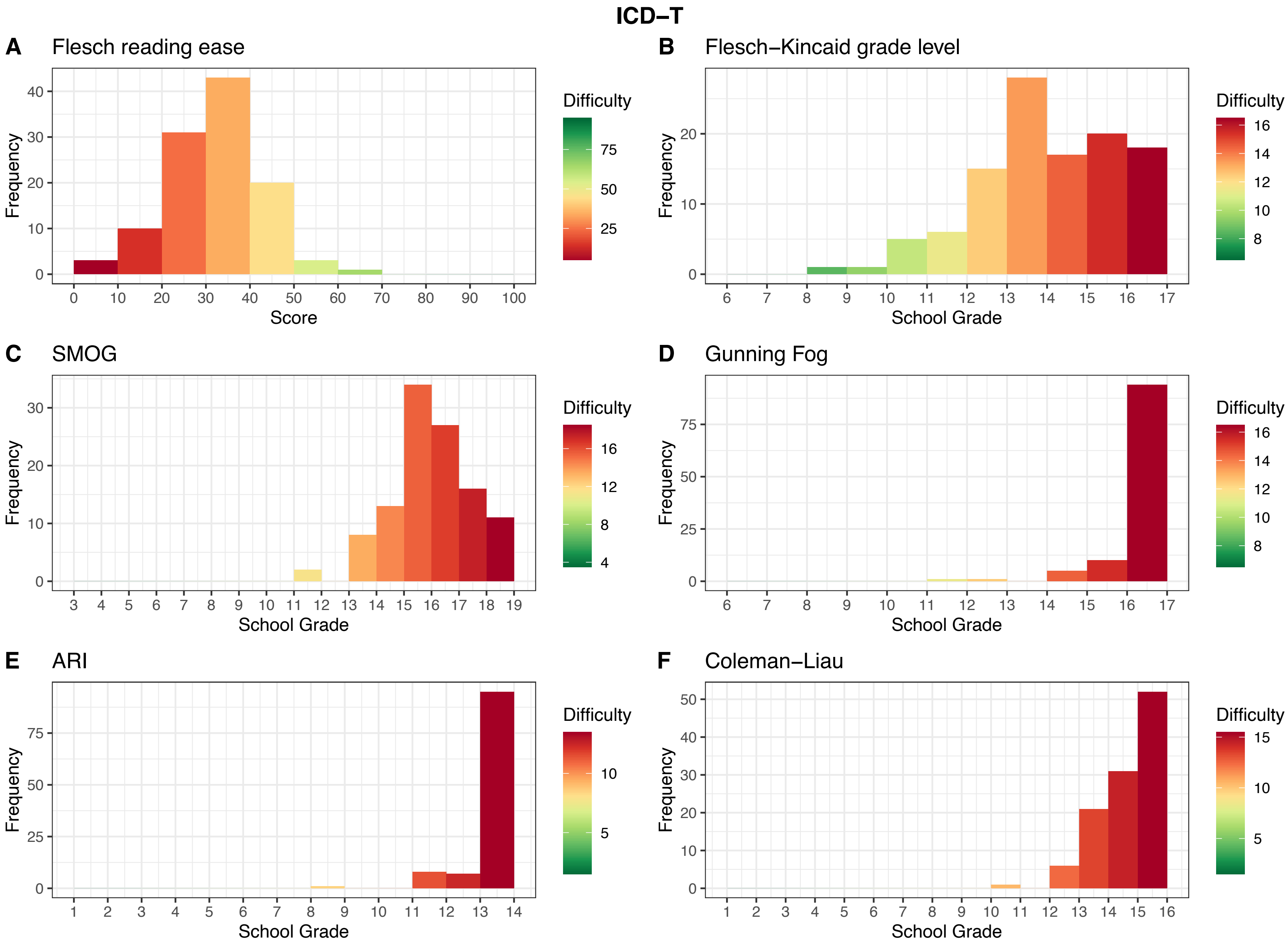

ICD–T
A
Flesch reading ease
Frequency
Score
Difficulty
B
Flesch–Kincaid grade level
School Grade
C
SMOG
D
Gunning Fog
E
ARI
F
Coleman–Liau

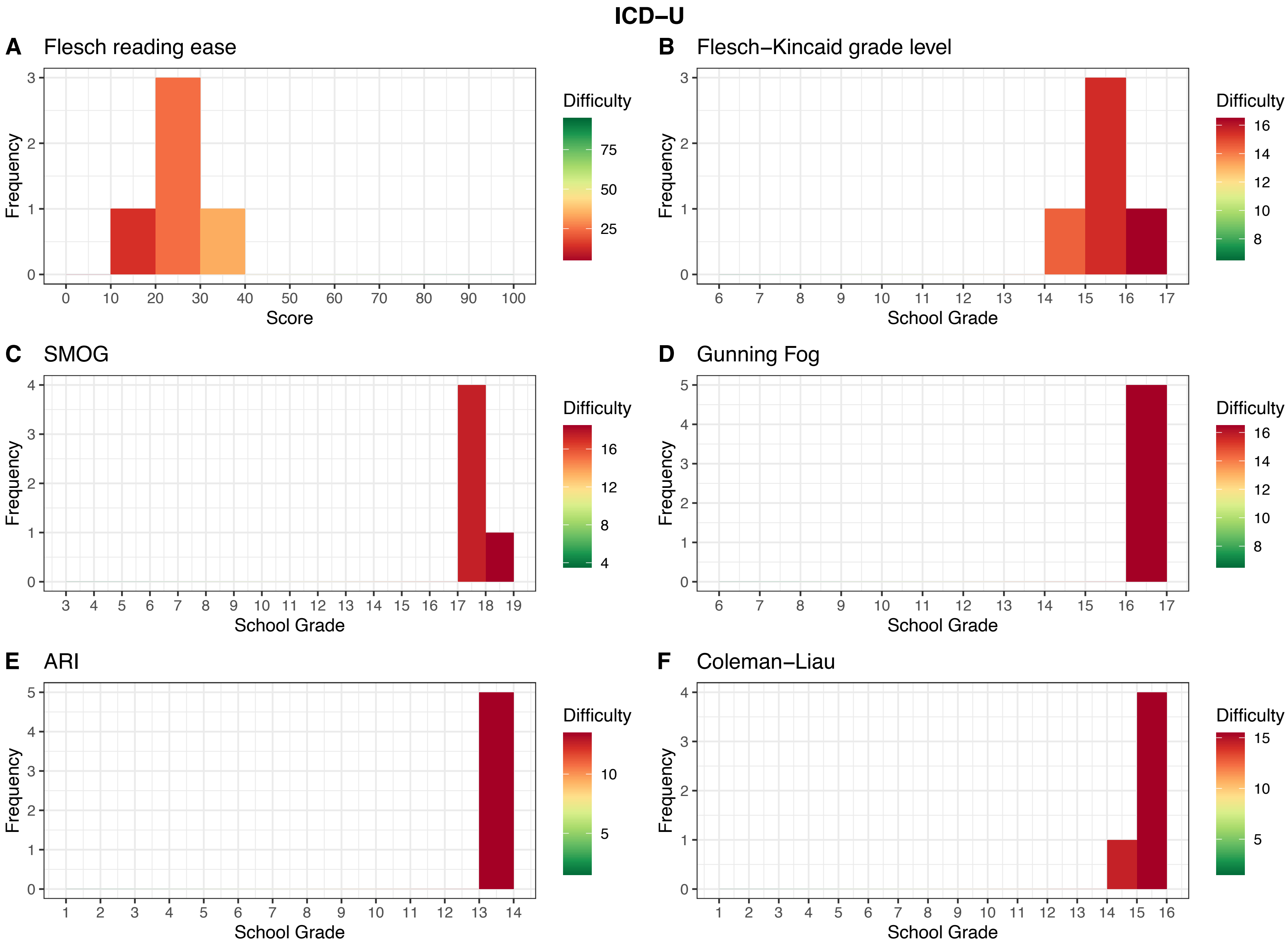

ICD–U
A Flesch reading ease
Frequency
Score
Difficulty
75
50
25
B Flesch–Kincaid grade level
School Grade
16
14
12
10
8
C SMOG
D Gunning Fog
E ARI
F Coleman–Liau

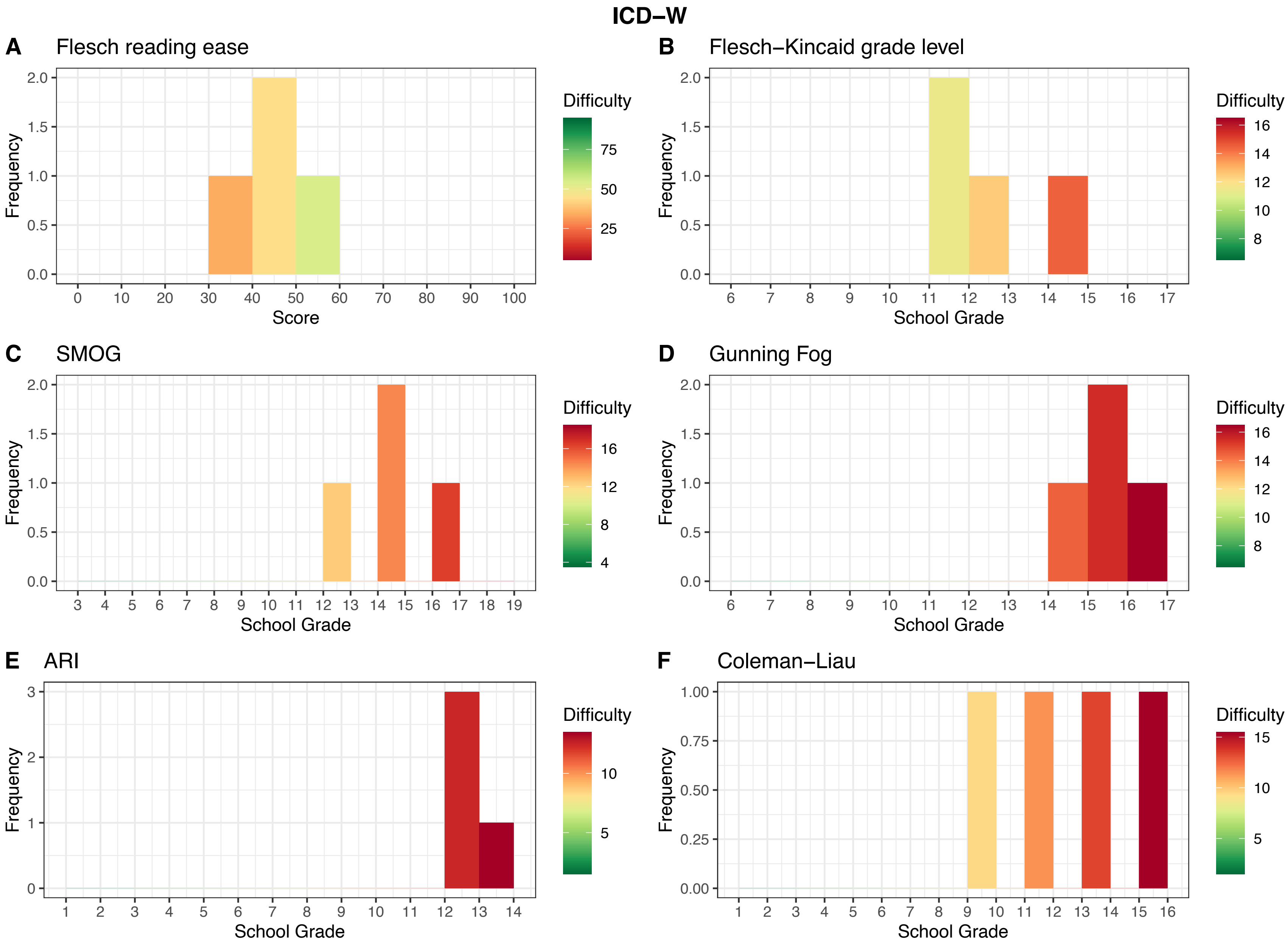
ICD–W
A Flesch reading ease
B Flesch–Kincaid grade level
C SMOG
D Gunning Fog
E ARI
F Coleman–Liau
Frequency
Score
School Grade
Difficulty

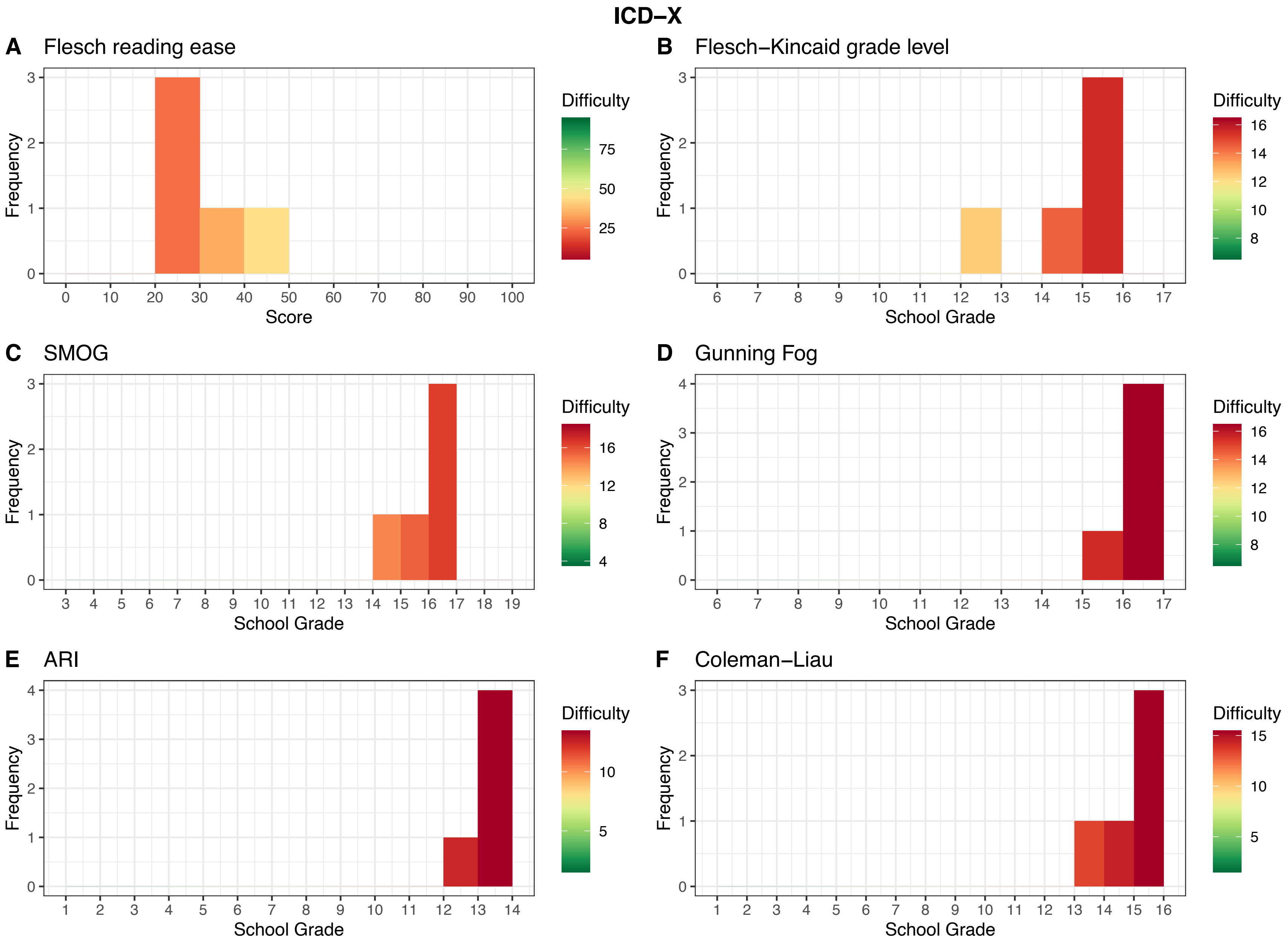
ICD–X
A Flesch reading ease
Frequency
Score
Difficulty
B Flesch–Kincaid grade level
School Grade
C SMOG
D Gunning Fog
E ARI
F Coleman–Liau

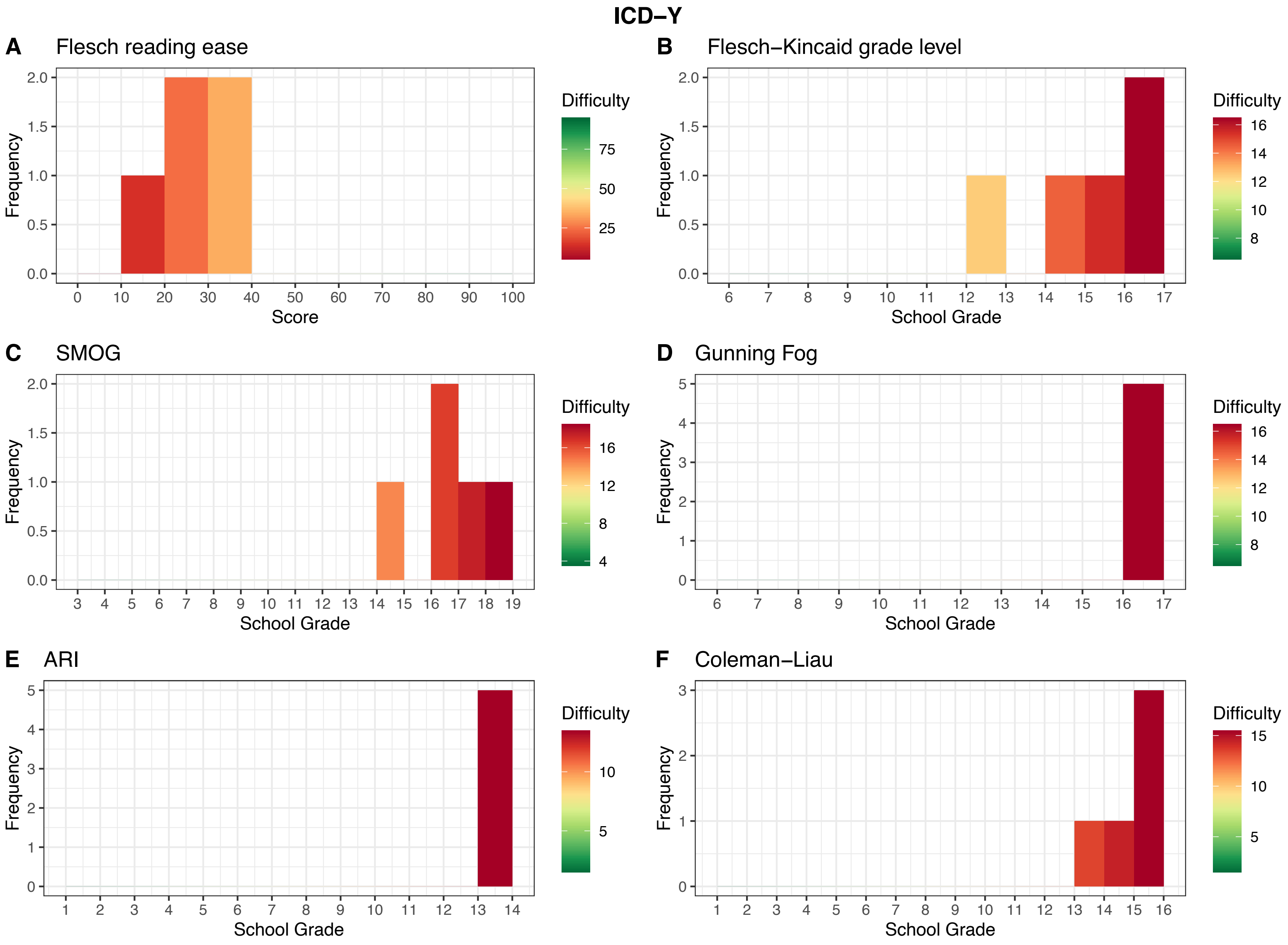
ICD–Y
A Flesch reading ease
Difficulty
75
50
25
Frequency
Score
B Flesch–Kincaid grade level
Difficulty
School Grade
C SMOG
Difficulty
School Grade
D Gunning Fog
Difficulty
School Grade
E ARI
Difficulty
School Grade
F Coleman–Liau
Difficulty
School Grade

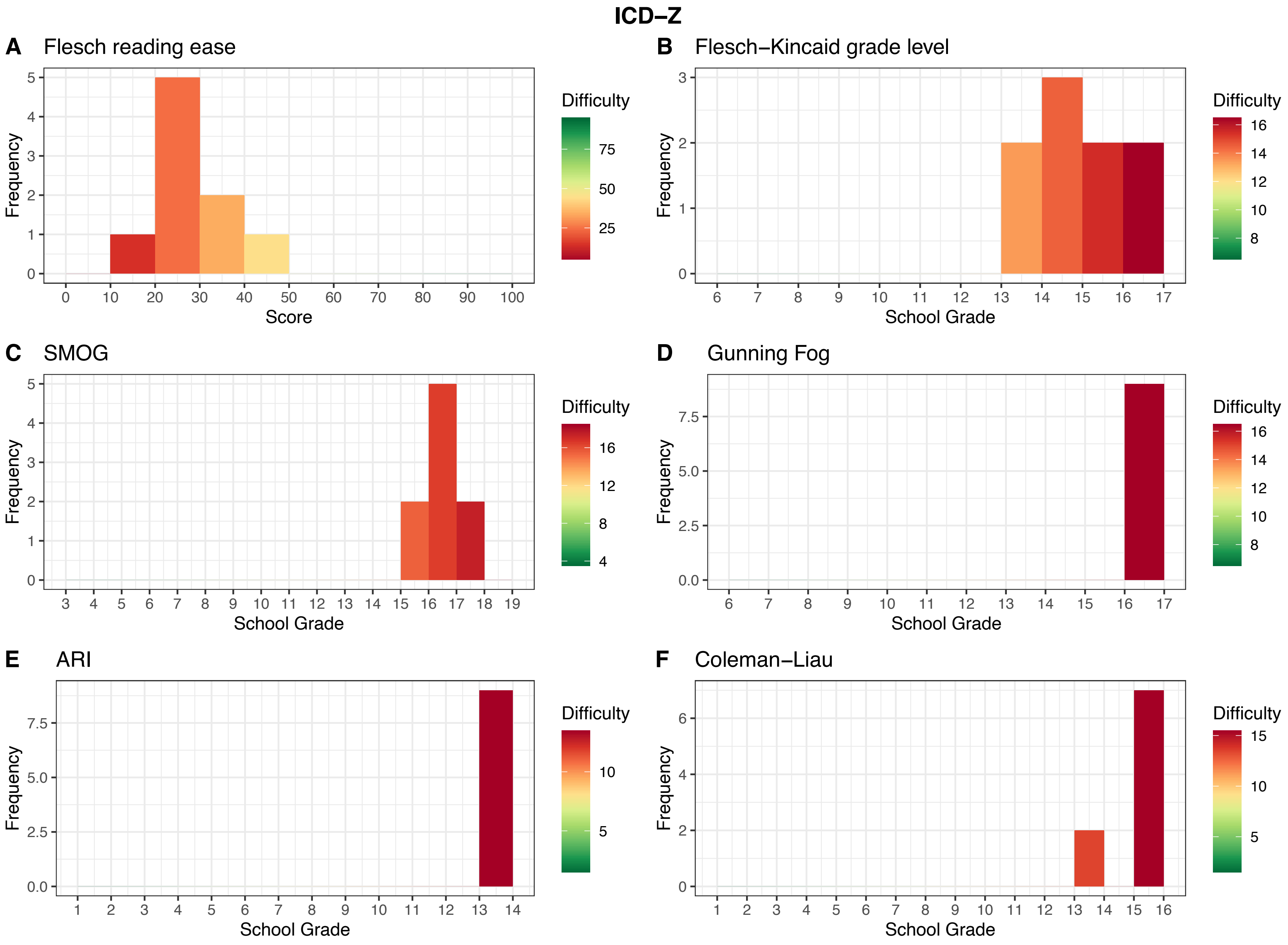
ICD–Z
A Flesch reading ease
Frequency
Score
Difficulty
75
50
25
B Flesch–Kincaid grade level
Frequency
School Grade
Difficulty
16
14
12
10
8
C SMOG
Frequency
School Grade
Difficulty
16
12
8
4
D Gunning Fog
Frequency
School Grade
Difficulty
16
14
12
10
8
E ARI
Frequency
School Grade
Difficulty
10
5
F Coleman–Liau
Frequency
School Grade
Difficulty
15
10
5
