# Supplementary material for: Readability of English, German, and Russian Disease-Related Wikipedia Pages: Automated Computational Analysis
Source: J Med Internet Res. 2022 May 16;24(5):e36835. doi: 10.2196/36835 (PMC9152717; doi:10.2196/36835)
Supplement: Multimedia Appendix 5 [file jmir_v24i5e36835_app5.pdf]

Multimedia Appendix 5: Distributions of all computed readability metrics in German

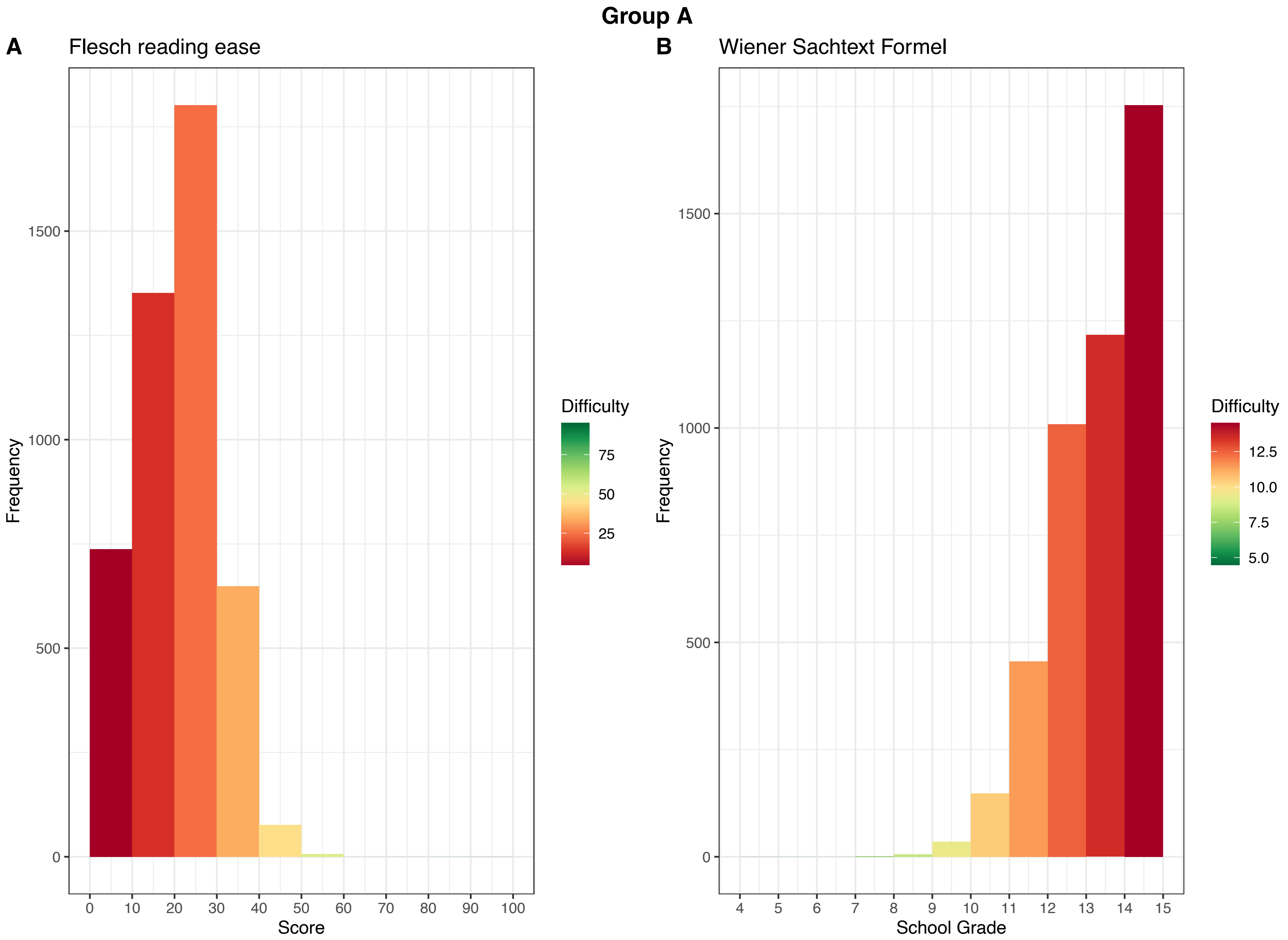
Group A
A
Flesch reading ease
Frequency
Score
Difficulty
75
50
25
B
Wiener Sachtext Formel
Frequency
School Grade
Difficulty
12.5
10.0
7.5
5.0

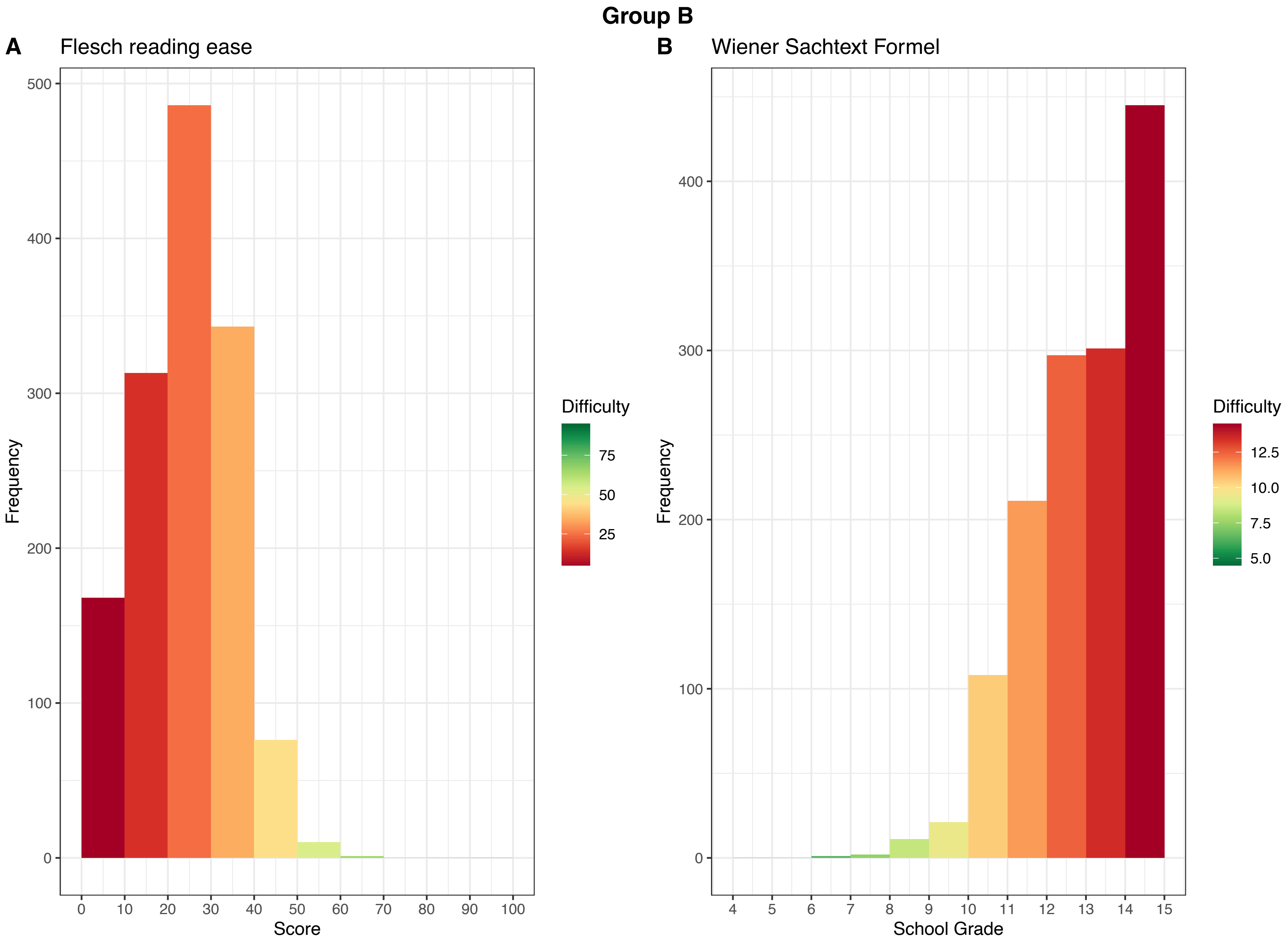
Group B
A
Flesch reading ease
Frequency
500
400
300
200
100
0
0
10
20
30
40
50
60
70
80
90
100
Score
Difficulty
75
50
25
B
Wiener Sachtext Formel
Frequency
400
300
200
100
0
4
5
6
7
8
9
10
11
12
13
14
15
School Grade
Difficulty
12.5
10.0
7.5
5.0

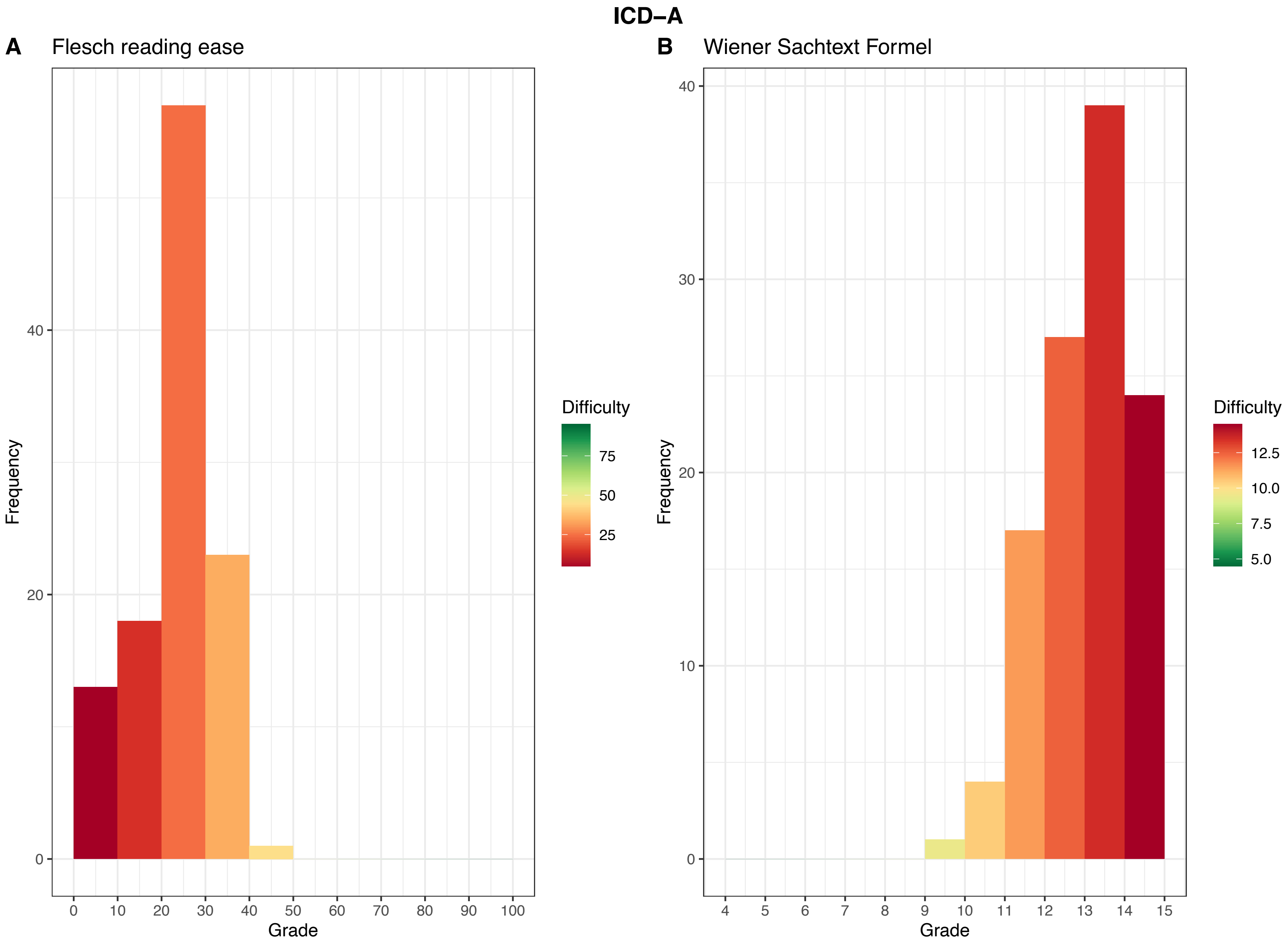
ICD–A
A
Flesch reading ease
Frequency
Grade
0
20
40
0
10
20
30
40
50
60
70
80
90
100
Difficulty
75
50
25
B
Wiener Sachtext Formel
Frequency
Grade
0
10
20
30
40
4
5
6
7
8
9
10
11
12
13
14
15
Difficulty
12.5
10.0
7.5
5.0

ICD–B

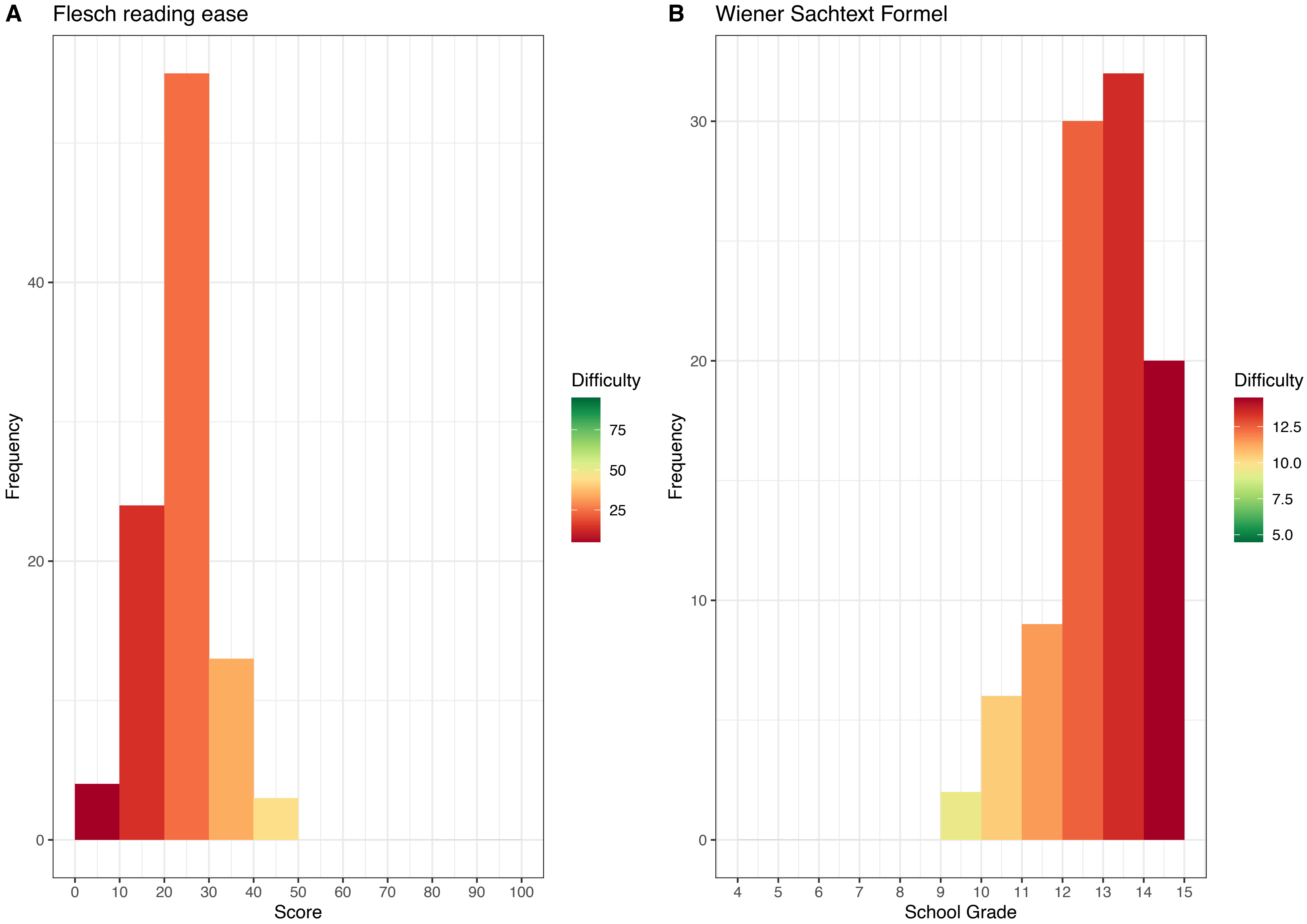

A
Flesch reading ease
Frequency
Score
Difficulty
75
50
25
0
20
40
0
10
20
30
40
50
60
70
80
90
100
B
Wiener Sachtext Formel
Frequency
School Grade
Difficulty
12.5
10.0
7.5
5.0
0
10
20
30
4
5
6
7
8
9
10
11
12
13
14
15

ICD–C

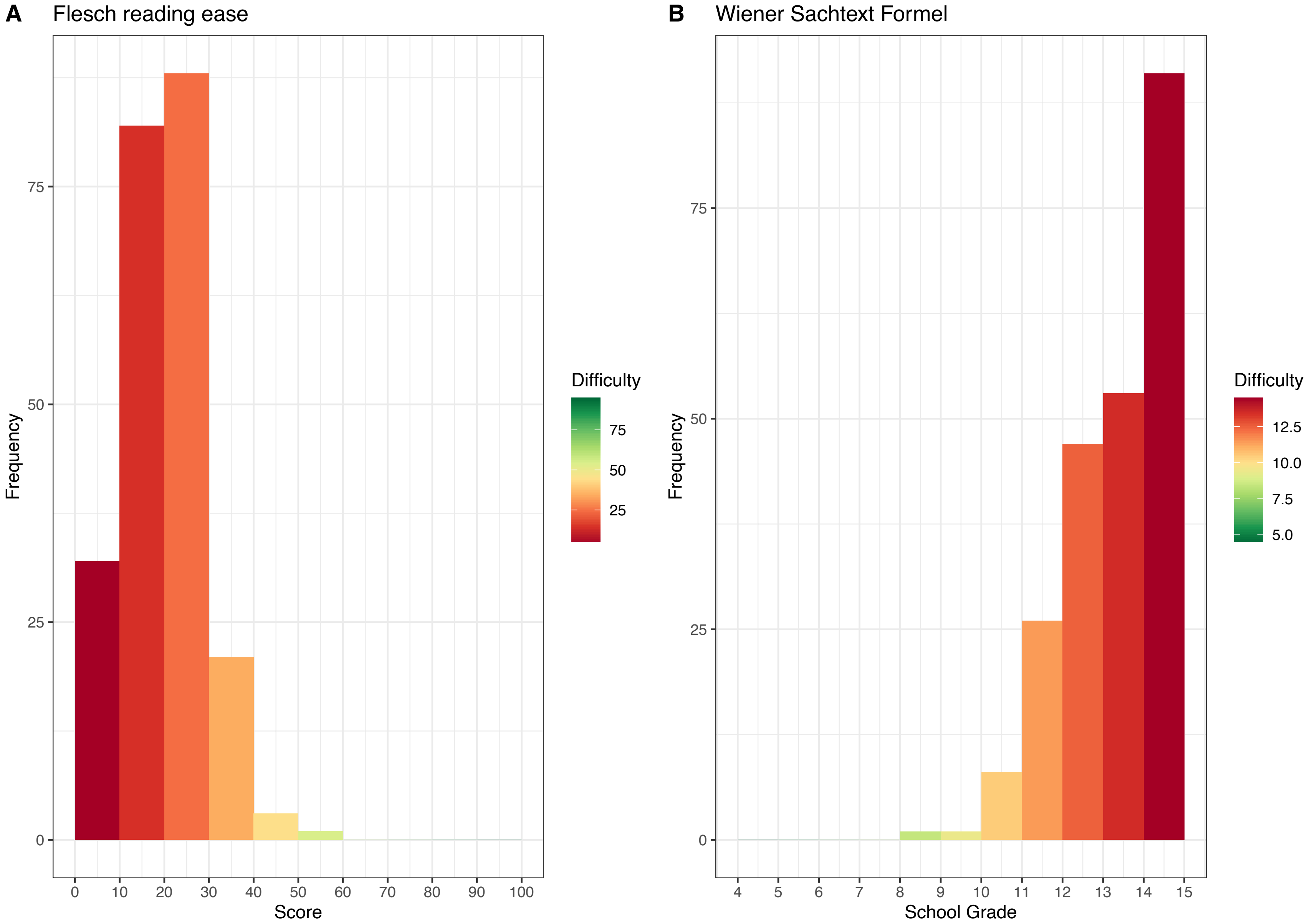
A
Flesch reading ease
Frequency
Score
0
25
50
75
0
10
20
30
40
50
60
70
80
90
100
Difficulty
75
50
25
B
Wiener Sachtext Formel
Frequency
School Grade
0
25
50
75
4
5
6
7
8
9
10
11
12
13
14
15
Difficulty
12.5
10.0
7.5
5.0

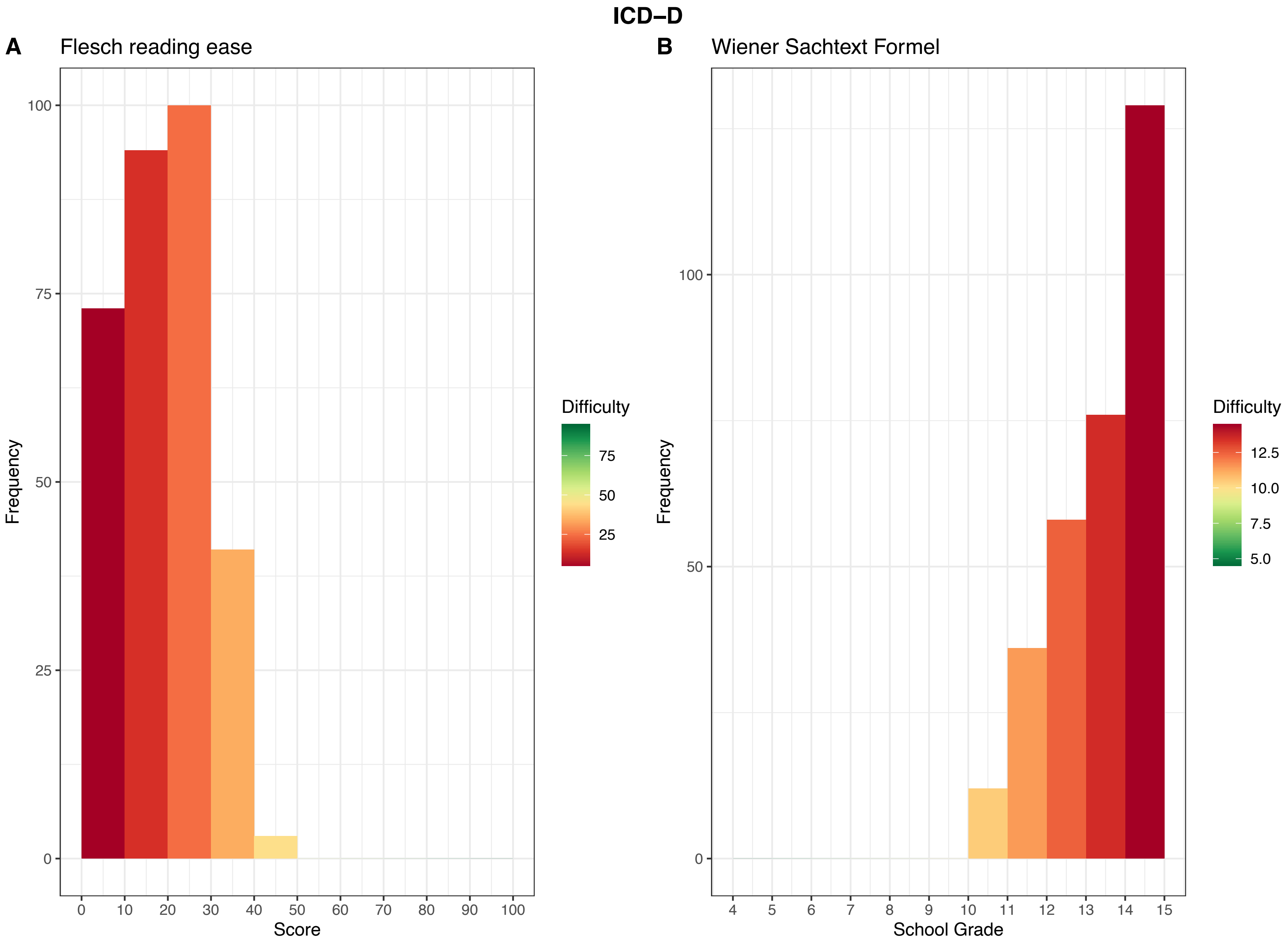
ICD–D
A
Flesch reading ease
Frequency
Score
Difficulty
75
50
25
B
Wiener Sachtext Formel
Frequency
School Grade
Difficulty
12.5
10.0
7.5
5.0

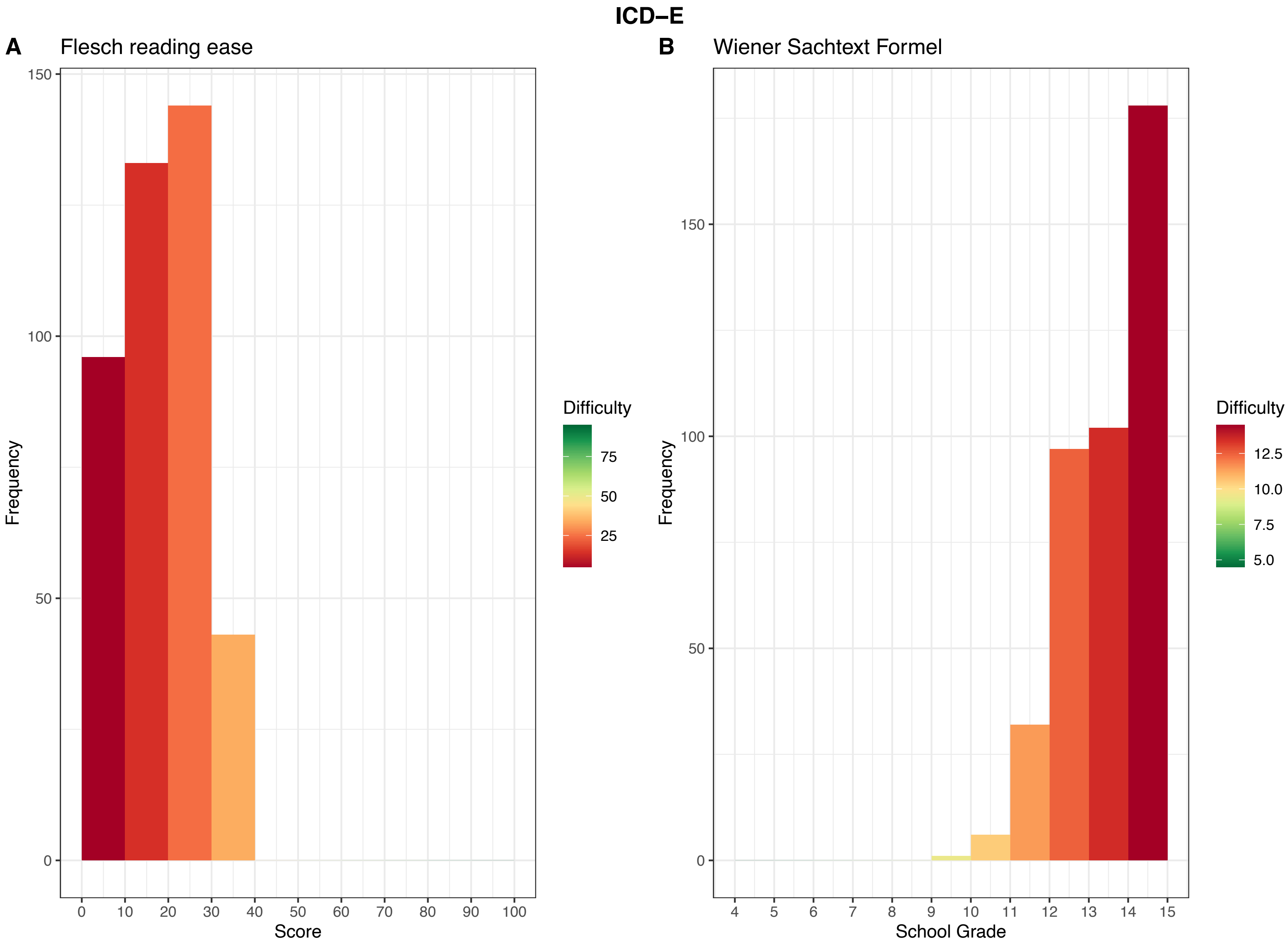
ICD–E
A
Flesch reading ease
Frequency
Score
Difficulty
75
50
25
0
50
100
150
0 10 20 30 40 50 60 70 80 90 100
B
Wiener Sachtext Formel
Frequency
School Grade
Difficulty
12.5
10.0
7.5
5.0
0
50
100
150
4 5 6 7 8 9 10 11 12 13 14 15

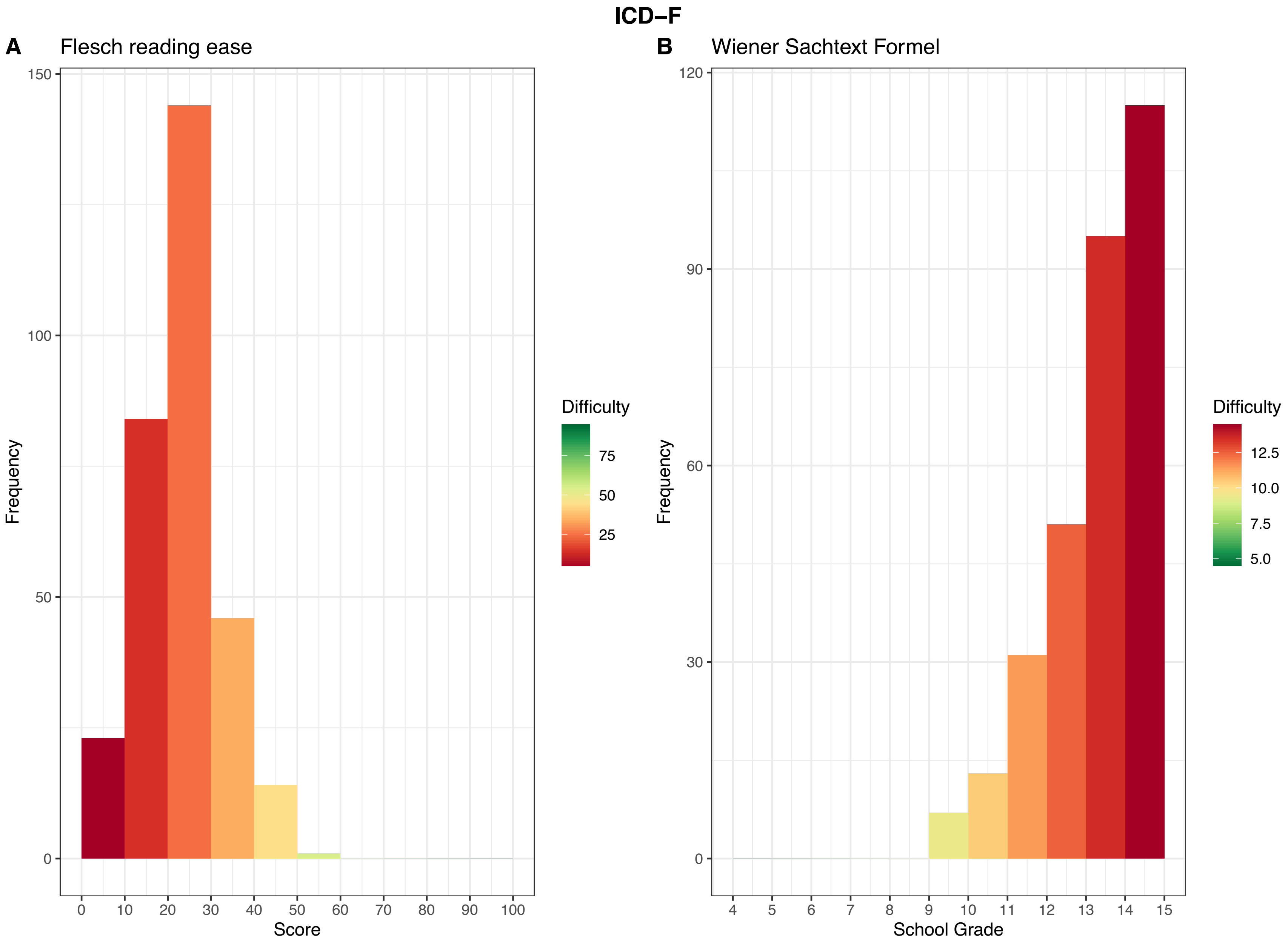
ICD–F
A
Flesch reading ease
Frequency
150
100
50
0
0
10
20
30
40
50
60
70
80
90
100
Score
Difficulty
75
50
25
B
Wiener Sachtext Formel
Frequency
120
90
60
30
0
4
5
6
7
8
9
10
11
12
13
14
15
School Grade
Difficulty
12.5
10.0
7.5
5.0

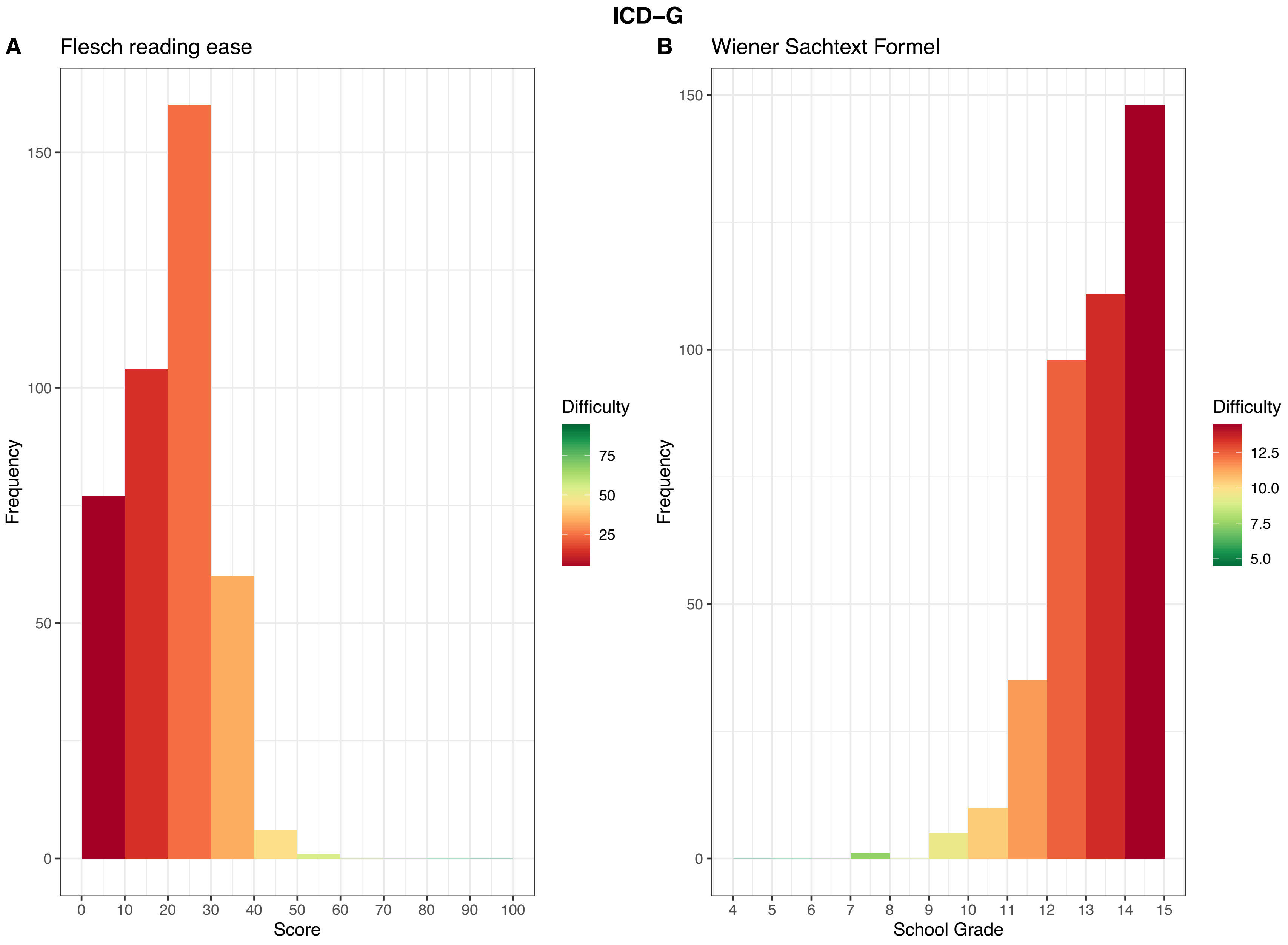
ICD–G
A
Flesch reading ease
150
100
50
0
Frequency
0
10
20
30
40
50
60
70
80
90
100
Score
Difficulty
75
50
25
B
Wiener Sachtext Formel
150
100
50
0
Frequency
4
5
6
7
8
9
10
11
12
13
14
15
School Grade
Difficulty
12.5
10.0
7.5
5.0

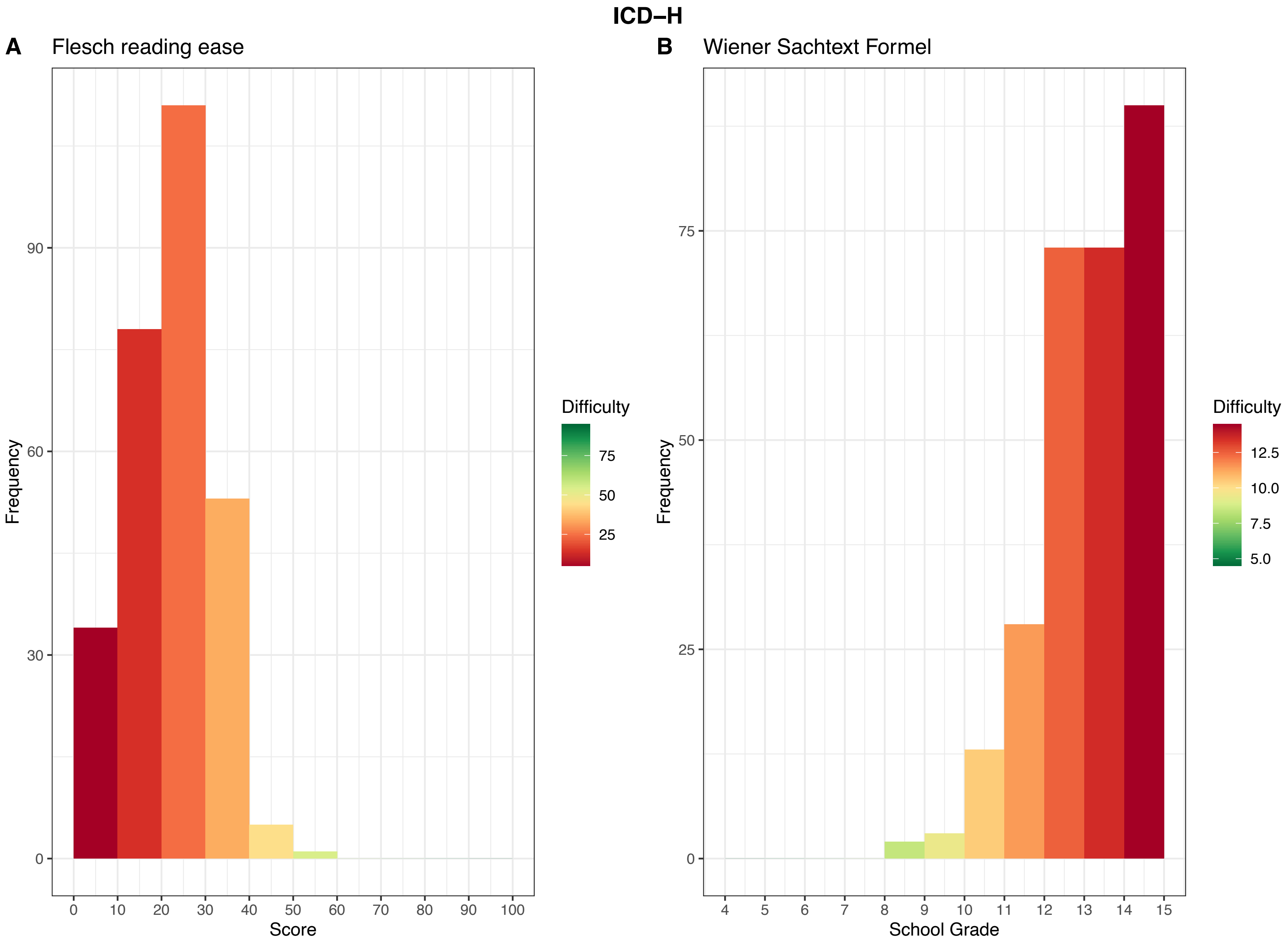
ICD–H
A
Flesch reading ease
Frequency
Score
0
30
60
90
0
10
20
30
40
50
60
70
80
90
100
Difficulty
75
50
25
B
Wiener Sachtext Formel
Frequency
School Grade
0
25
50
75
4
5
6
7
8
9
10
11
12
13
14
15
Difficulty
12.5
10.0
7.5
5.0

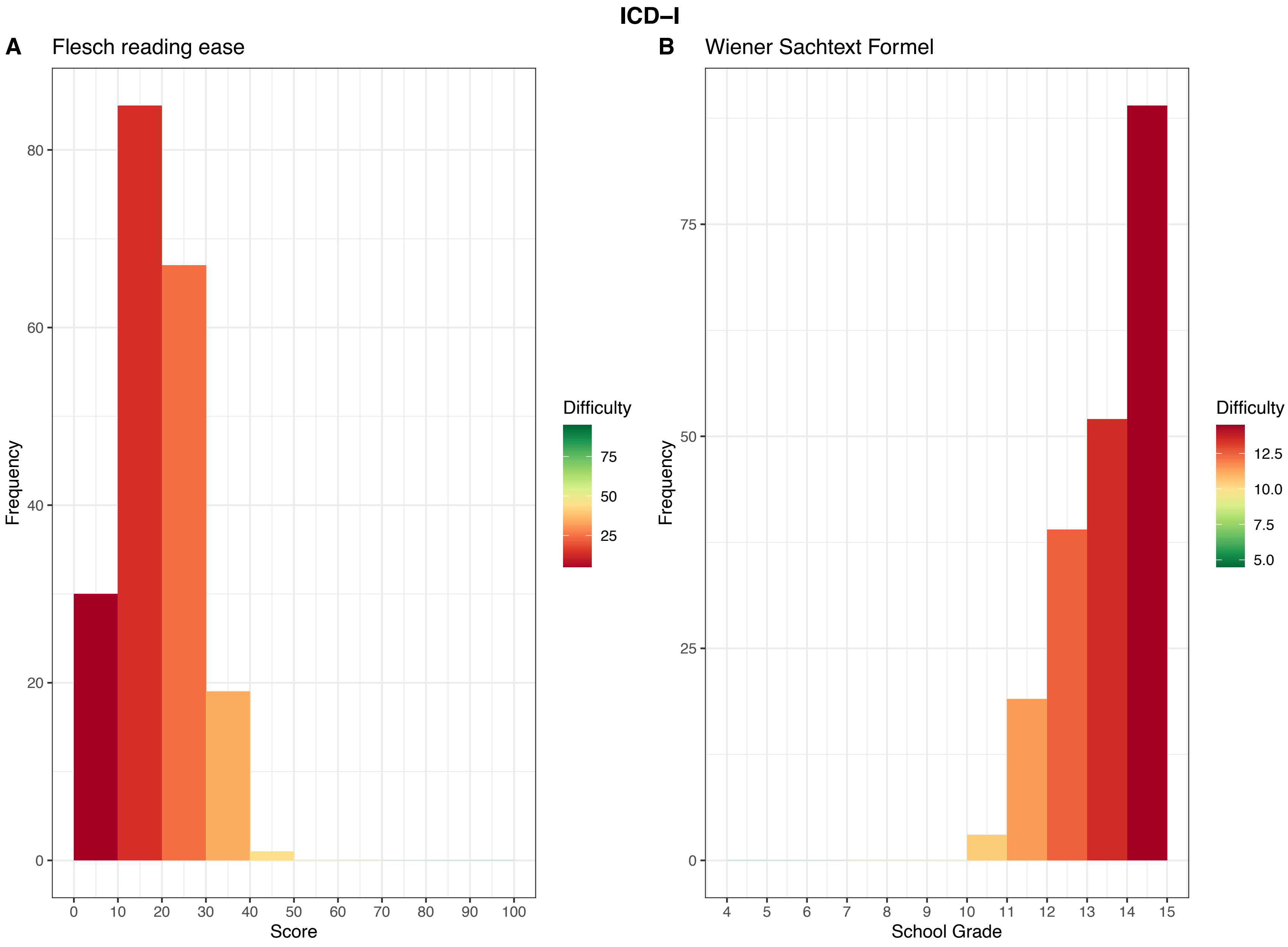

ICD–I
A
Flesch reading ease
Frequency
Score
Difficulty
75
50
25
B
Wiener Sachtext Formel
Frequency
School Grade
Difficulty
12.5
10.0
7.5
5.0

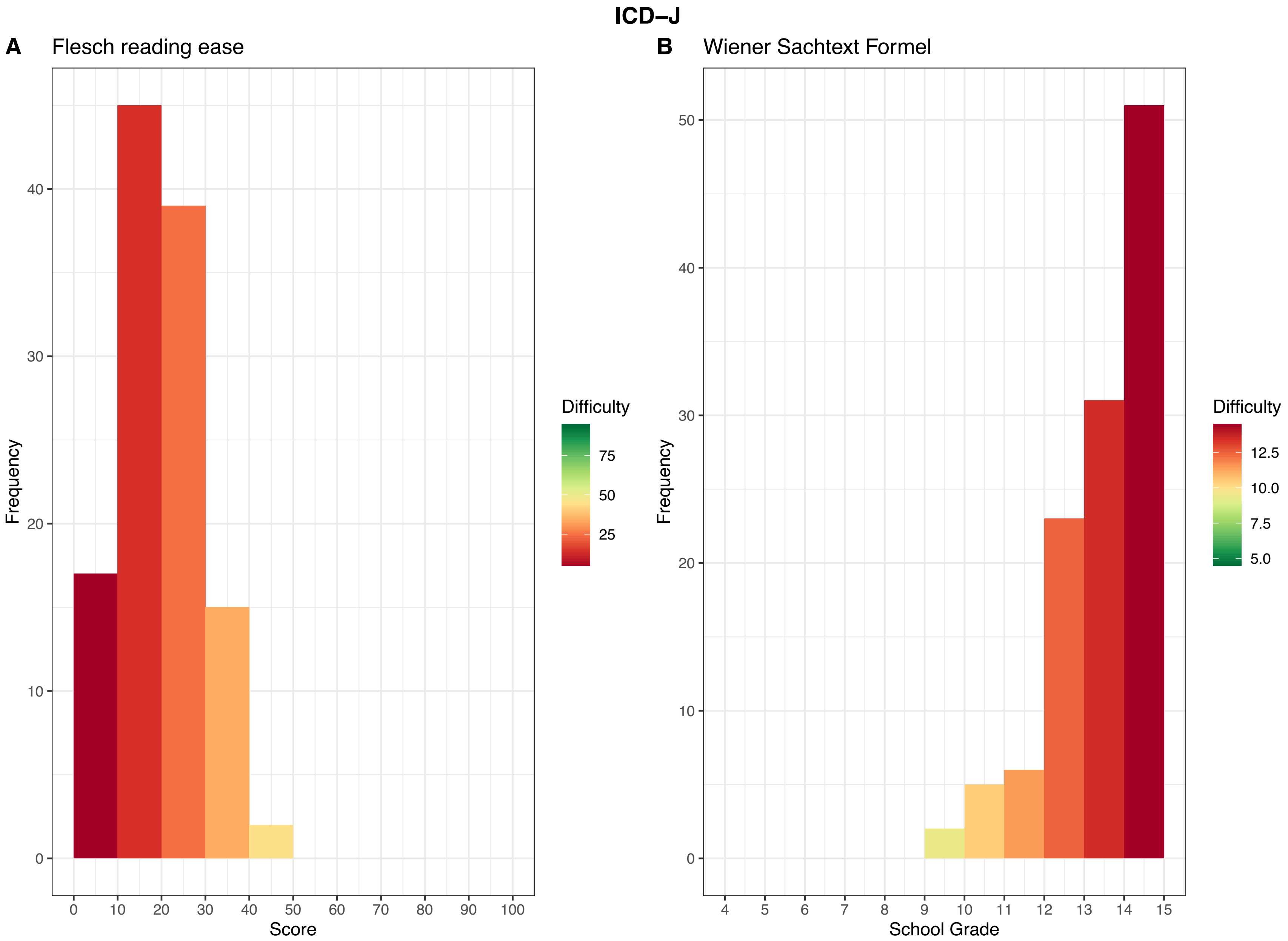
ICD–J
A Flesch reading ease
Frequency
Score
Difficulty
75
50
25
B Wiener Sachtext Formel
Frequency
School Grade
Difficulty
12.5
10.0
7.5
5.0

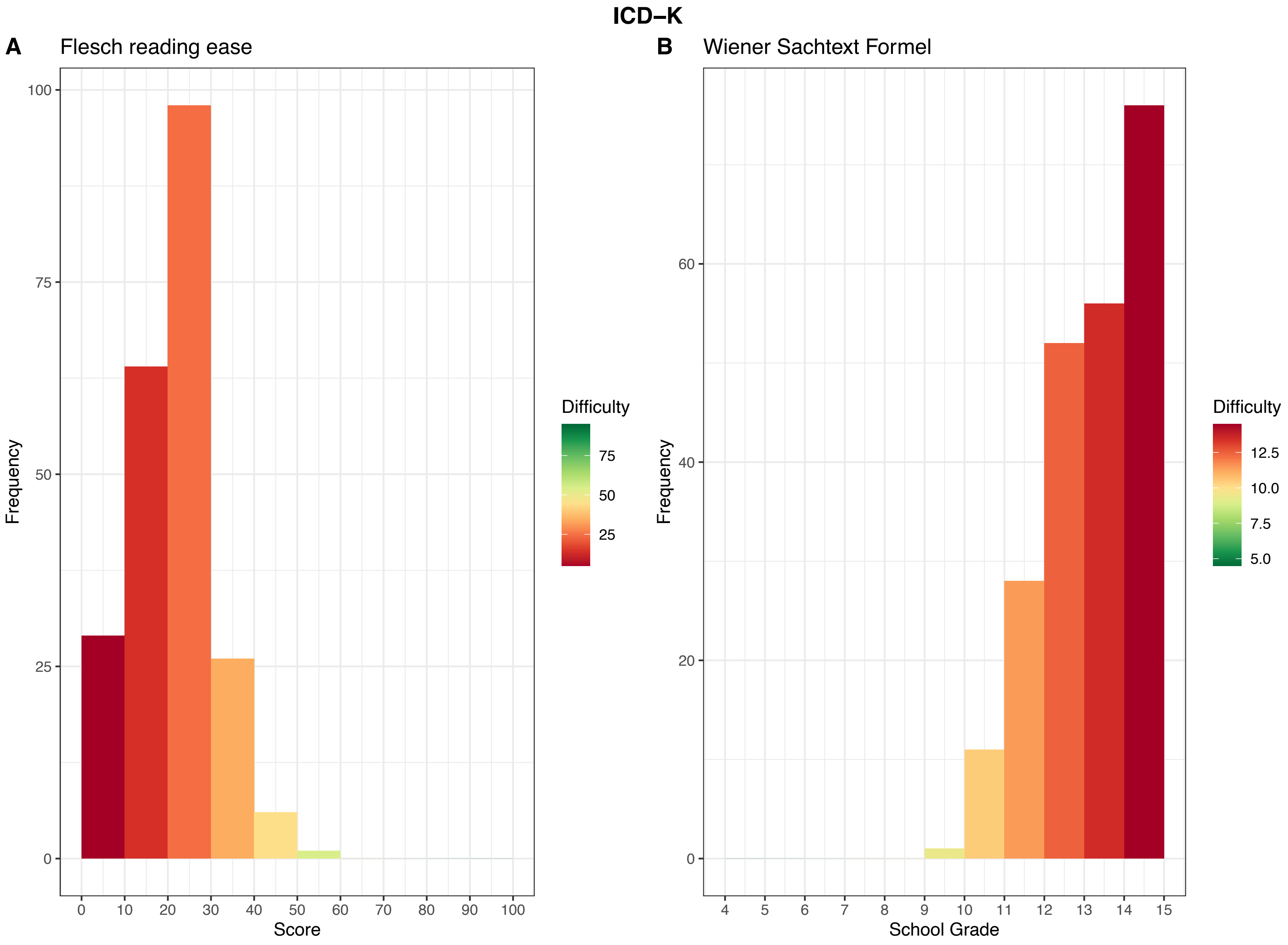
ICD–K
A
Flesch reading ease
Frequency
Score
0
25
50
75
100
0
10
20
30
40
50
60
70
80
90
100
Difficulty
75
50
25
B
Wiener Sachtext Formel
Frequency
School Grade
0
20
40
60
4
5
6
7
8
9
10
11
12
13
14
15
Difficulty
12.5
10.0
7.5
5.0

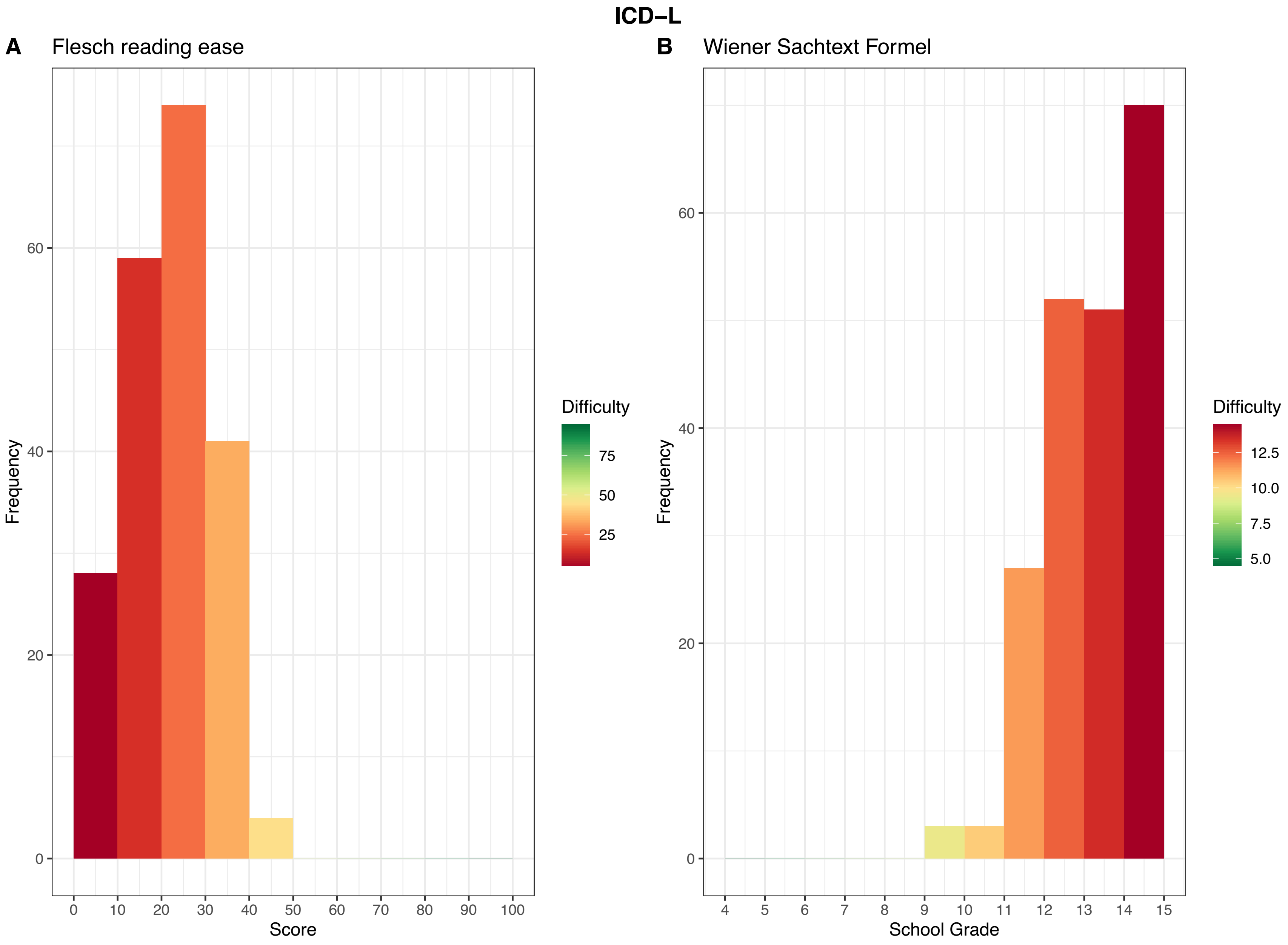

ICD–L
A
Flesch reading ease
Frequency
Score
Difficulty
75
50
25
B
Wiener Sachtext Formel
Frequency
School Grade
Difficulty
12.5
10.0
7.5
5.0

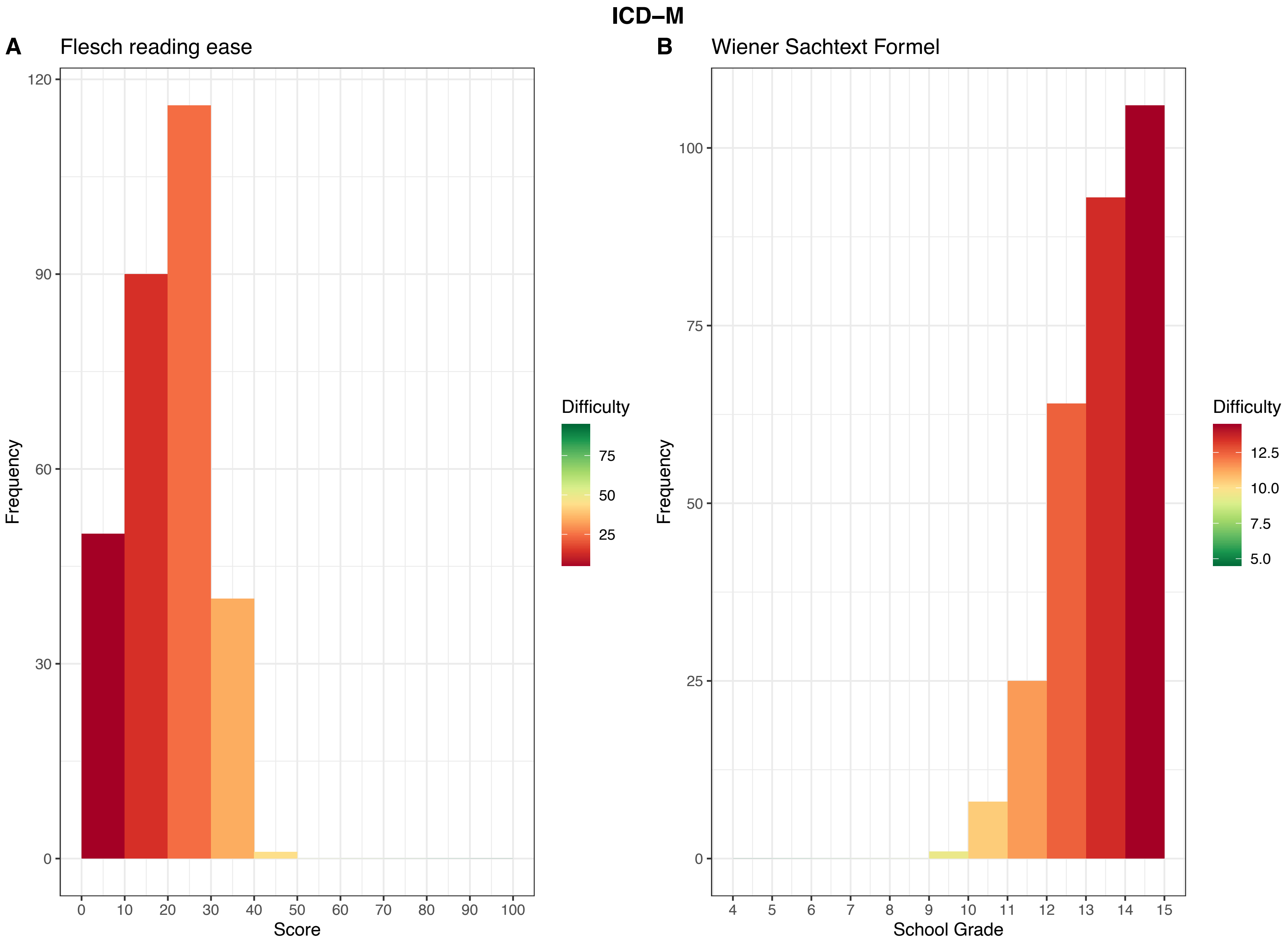

ICD–M
A
Flesch reading ease
Frequency
Score
Difficulty
75
50
25
0
10
20
30
40
50
60
70
80
90
100
120
90
60
30
0
B
Wiener Sachtext Formel
Frequency
School Grade
Difficulty
12.5
10.0
7.5
5.0
4
5
6
7
8
9
10
11
12
13
14
15
100
75
50
25
0

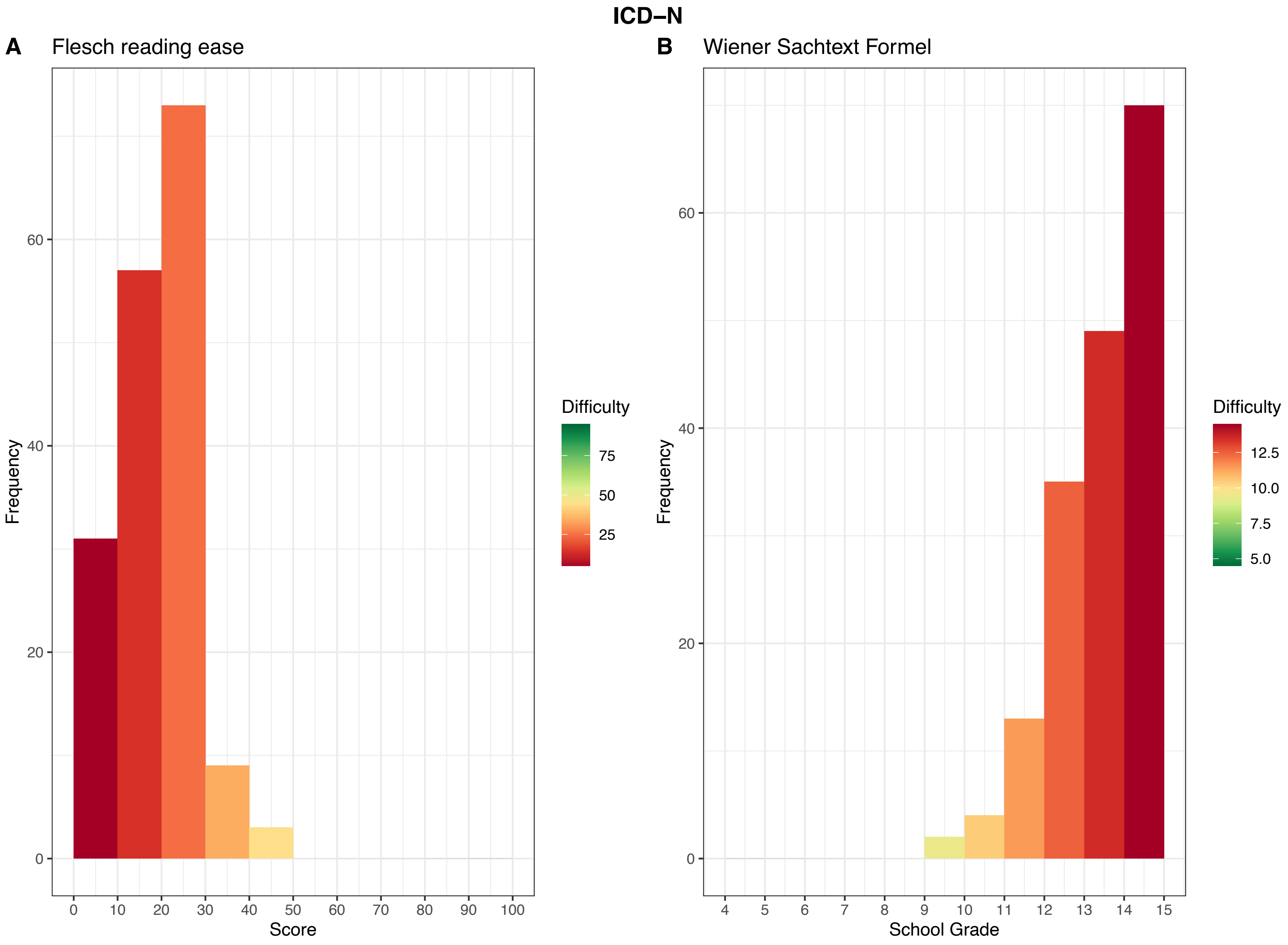
ICD–N
A
Flesch reading ease
Frequency
Score
Difficulty
75
50
25
0
20
40
60
0
10
20
30
40
50
60
70
80
90
100
B
Wiener Sachtext Formel
Frequency
School Grade
Difficulty
12.5
10.0
7.5
5.0
0
20
40
60
4
5
6
7
8
9
10
11
12
13
14
15

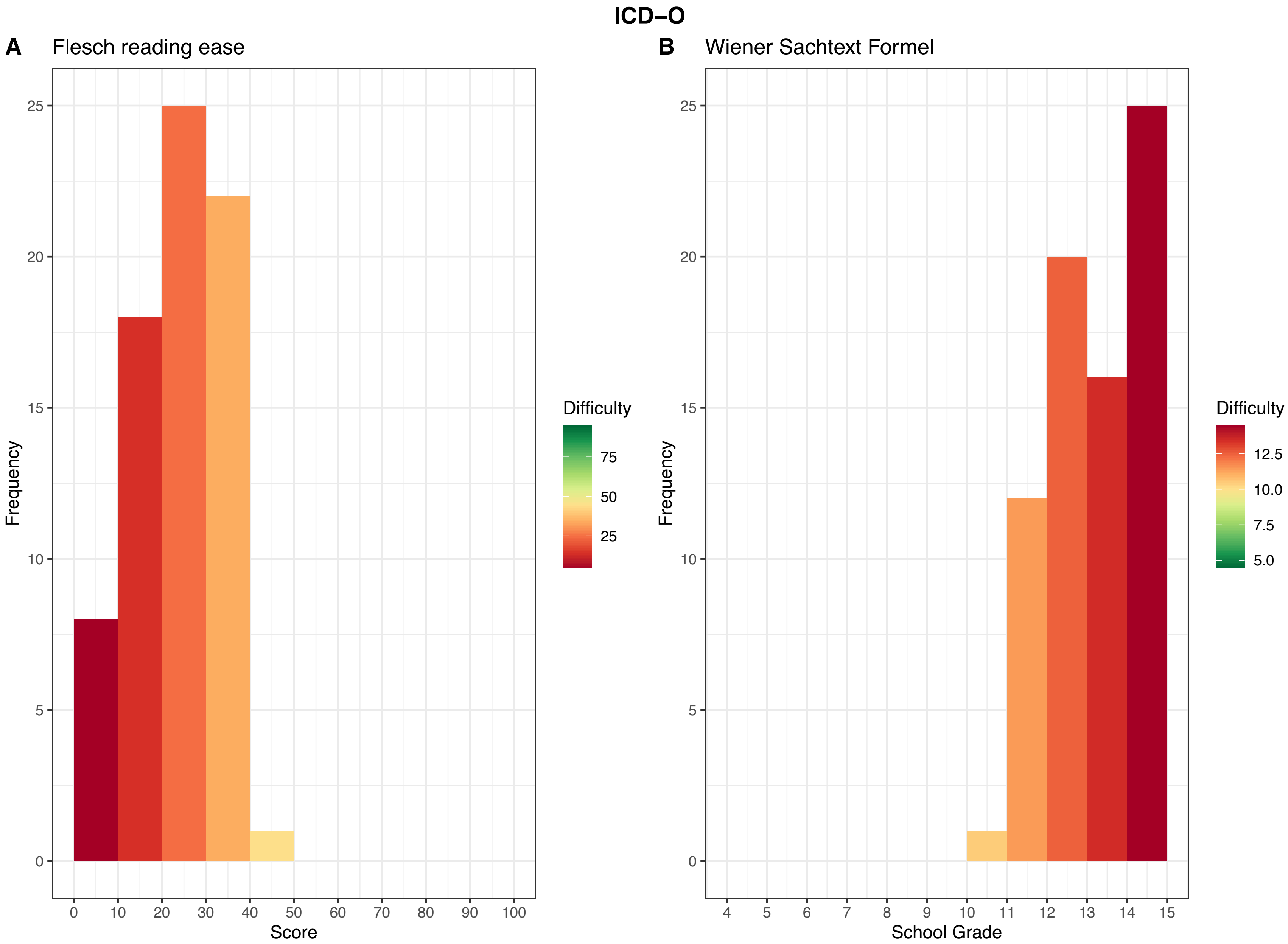
ICD–O
A
Flesch reading ease
Frequency
Score
Difficulty
75
50
25
B
Wiener Sachtext Formel
Frequency
School Grade
Difficulty
12.5
10.0
7.5
5.0

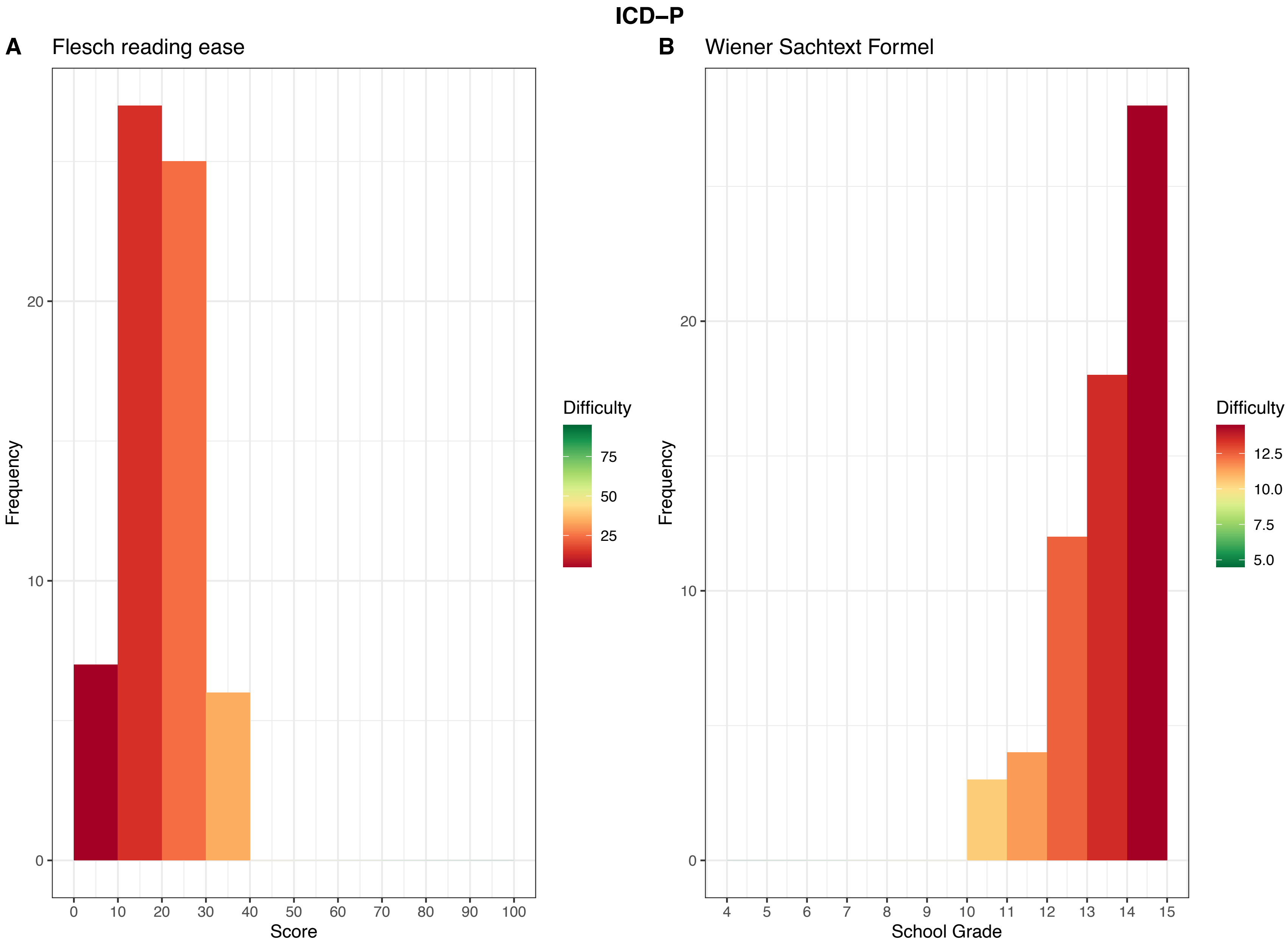
ICD–P
A
Flesch reading ease
Frequency
Score
Difficulty
75
50
25
B
Wiener Sachtext Formel
Frequency
School Grade
Difficulty
12.5
10.0
7.5
5.0

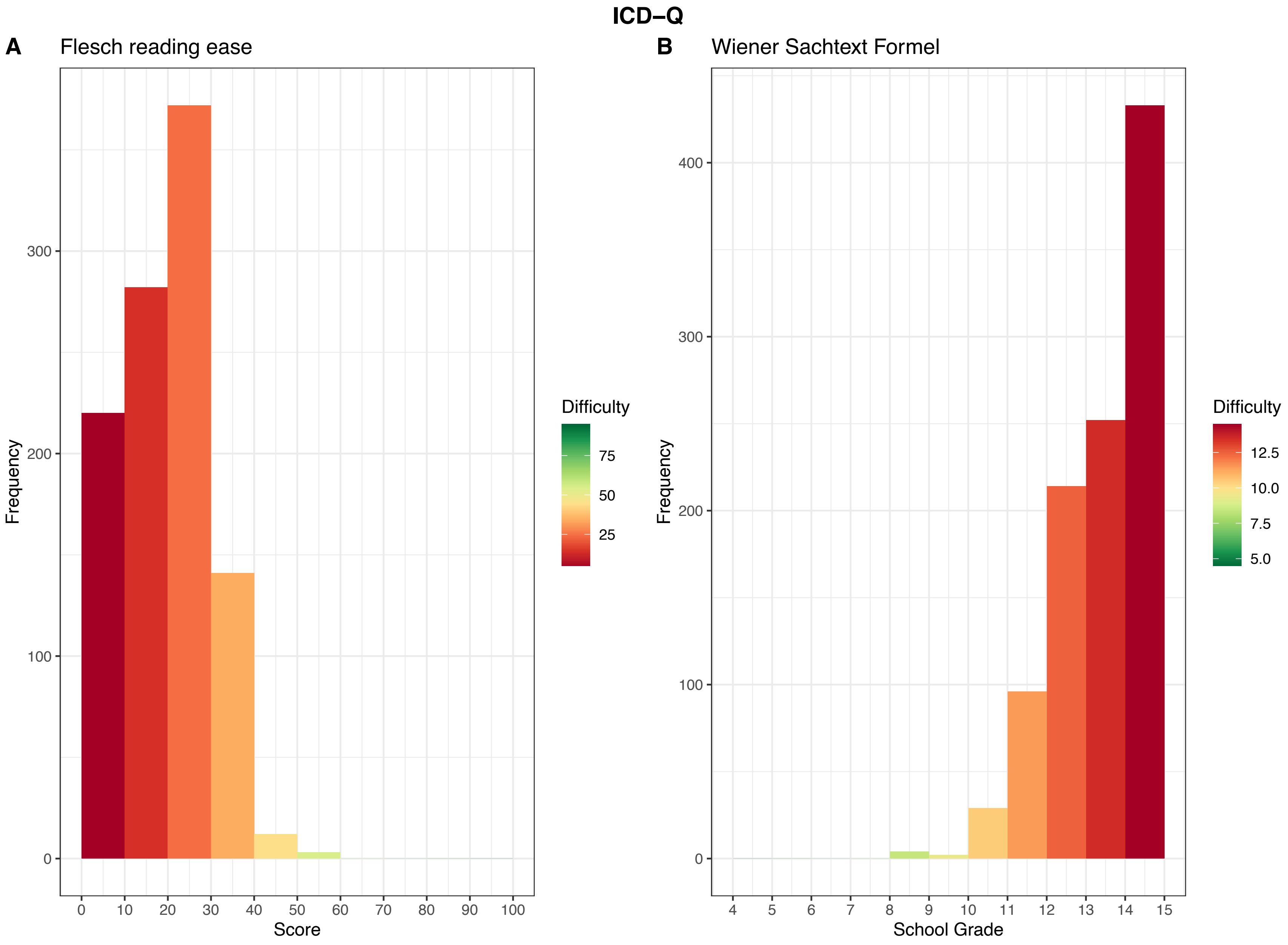
ICD–Q
A
Flesch reading ease
Frequency
Score
Difficulty
75
50
25
B
Wiener Sachtext Formel
Frequency
School Grade
Difficulty
12.5
10.0
7.5
5.0

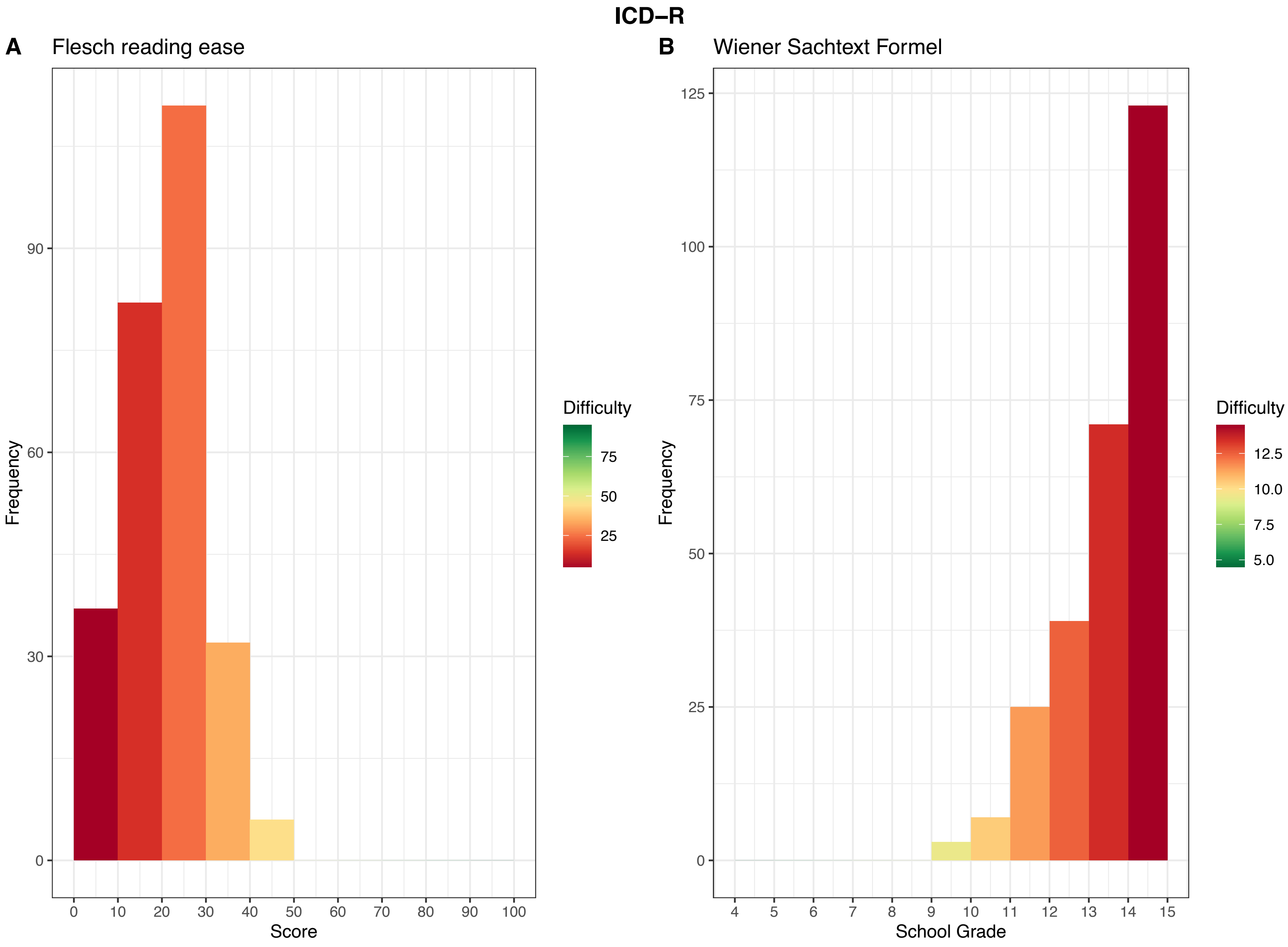
ICD–R
A
Flesch reading ease
Frequency
90
60
30
0
0
10
20
30
40
50
60
70
80
90
100
Score
Difficulty
75
50
25
B
Wiener Sachtext Formel
Frequency
125
100
75
50
25
0
4
5
6
7
8
9
10
11
12
13
14
15
School Grade
Difficulty
12.5
10.0
7.5
5.0

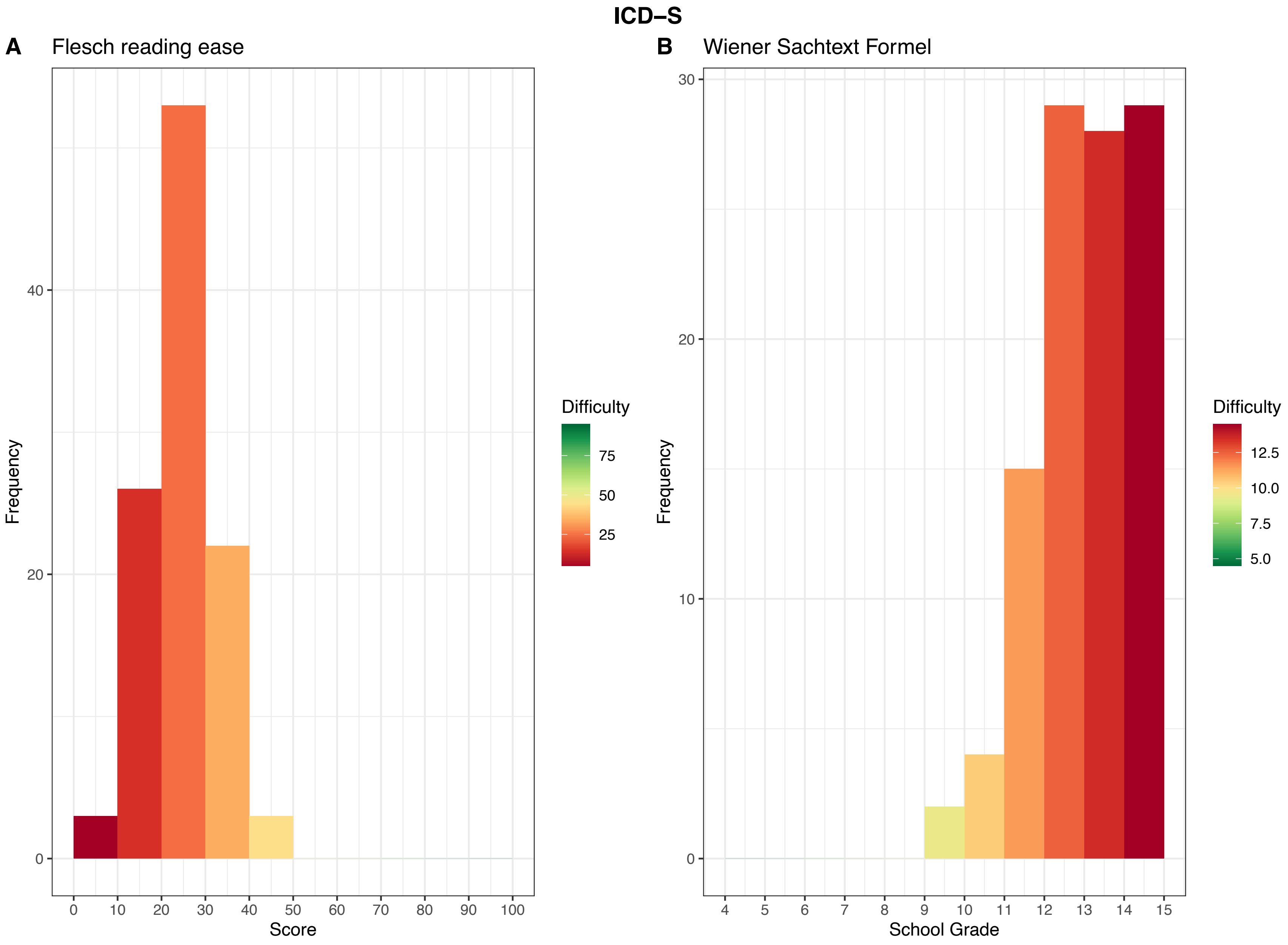

ICD–S
A
Flesch reading ease
Frequency
Score
Difficulty
75
50
25
B
Wiener Sachtext Formel
Frequency
School Grade
Difficulty
12.5
10.0
7.5
5.0

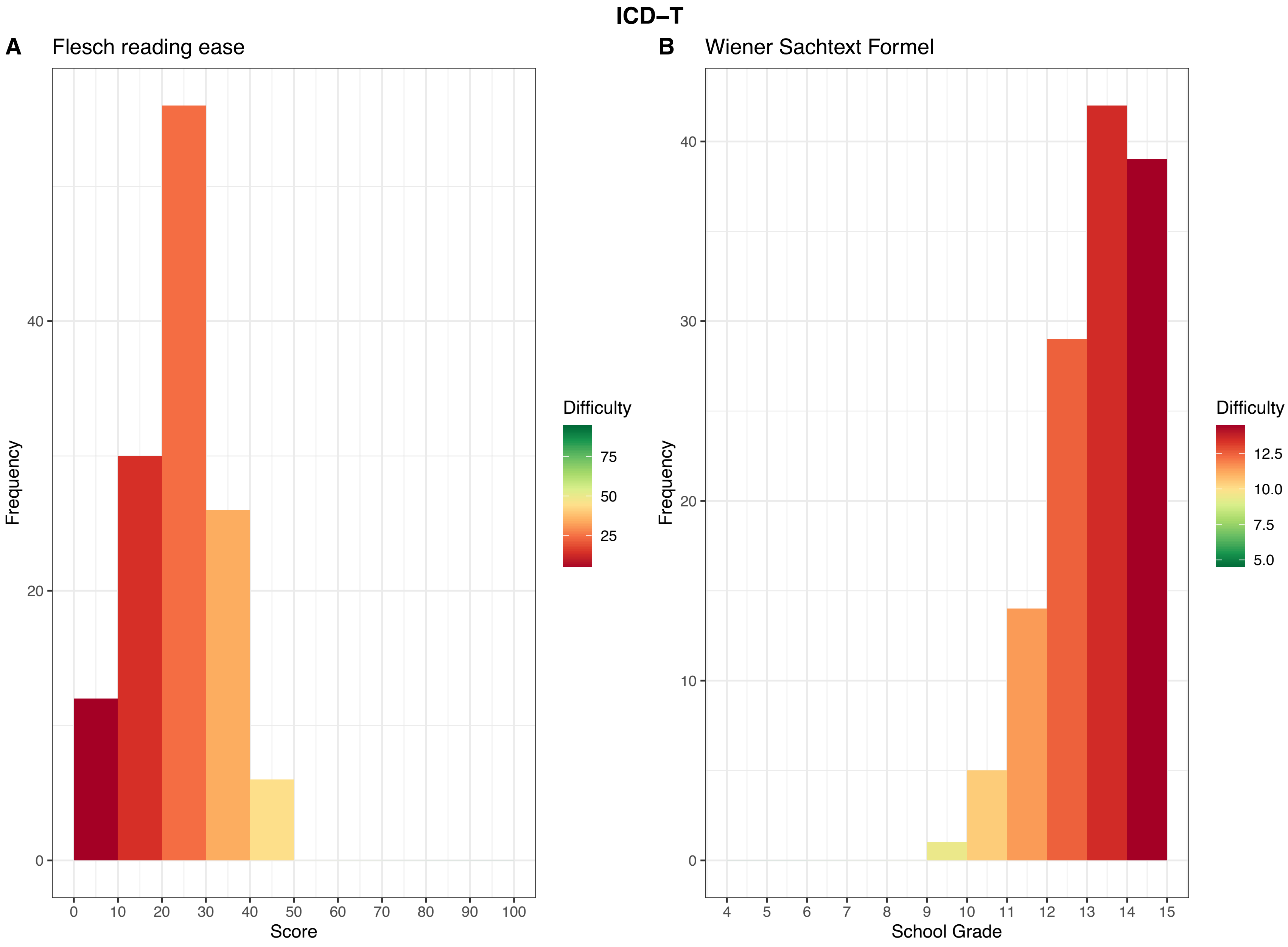
ICD–T
A
Flesch reading ease
Frequency
0
20
40
0
10
20
30
40
50
60
70
80
90
100
Score
Difficulty
75
50
25
B
Wiener Sachtext Formel
Frequency
0
10
20
30
40
4
5
6
7
8
9
10
11
12
13
14
15
School Grade
Difficulty
12.5
10.0
7.5
5.0

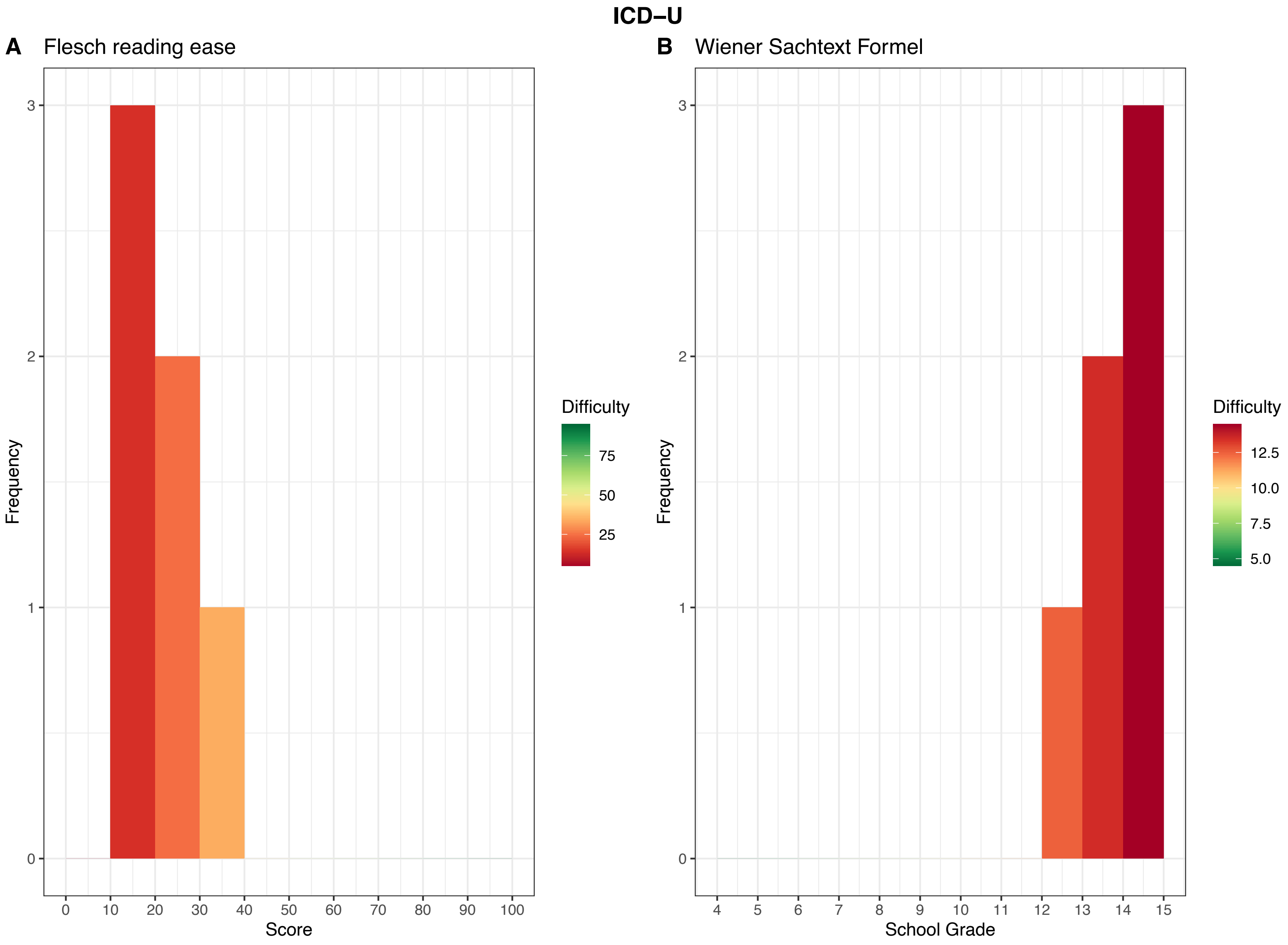
ICD–U
A Flesch reading ease
Frequency
3
2
1
0
0 10 20 30 40 50 60 70 80 90 100
Score
Difficulty
75
50
25
B Wiener Sachtext Formel
Frequency
3
2
1
0
4 5 6 7 8 9 10 11 12 13 14 15
School Grade
Difficulty
12.5
10.0
7.5
5.0

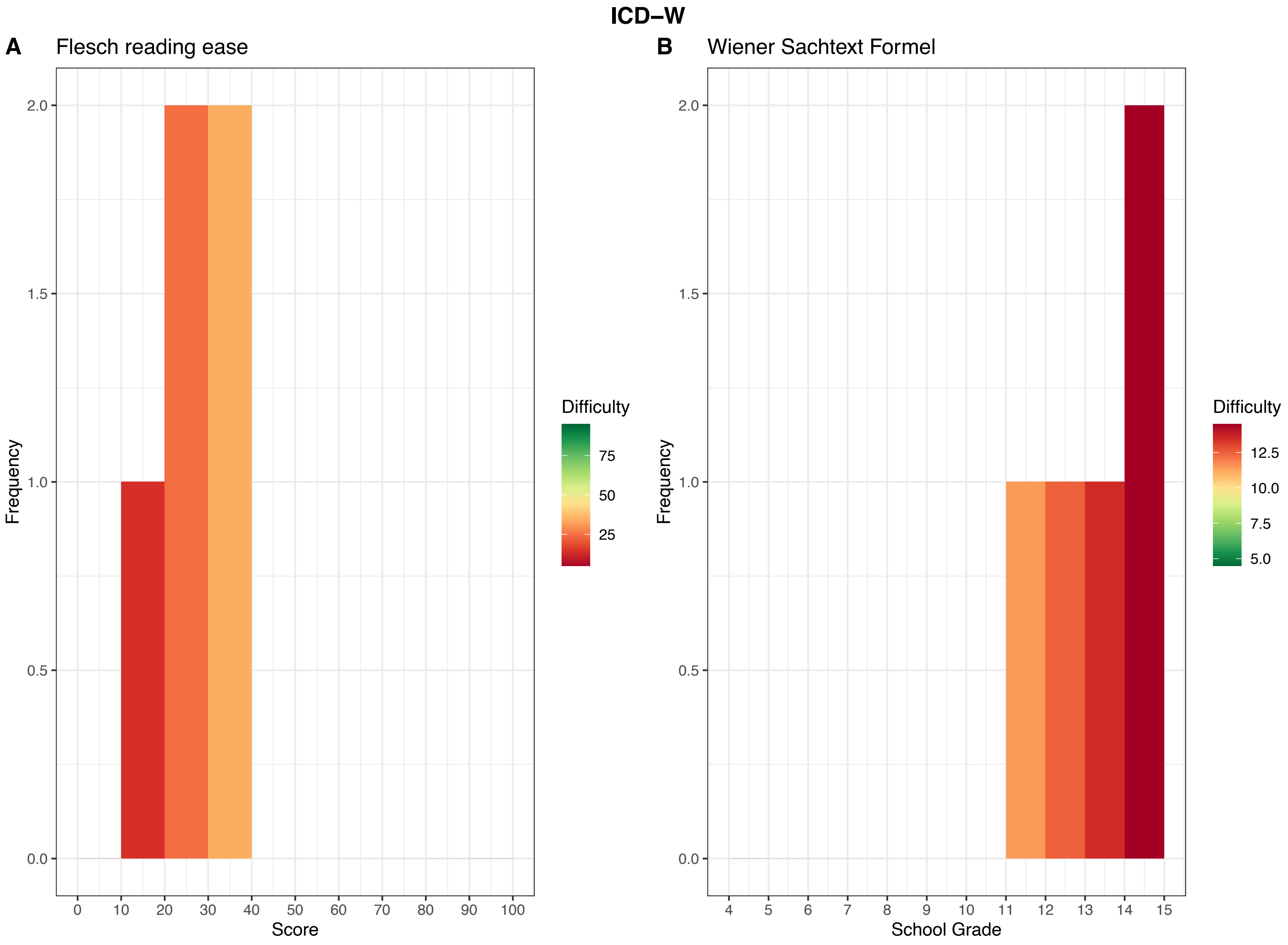

ICD–W
A
Flesch reading ease
Frequency
0.0
0.5
1.0
1.5
2.0
0
10
20
30
40
50
60
70
80
90
100
Score
Difficulty
75
50
25
B
Wiener Sachtext Formel
Frequency
0.0
0.5
1.0
1.5
2.0
4
5
6
7
8
9
10
11
12
13
14
15
School Grade
Difficulty
12.5
10.0
7.5
5.0

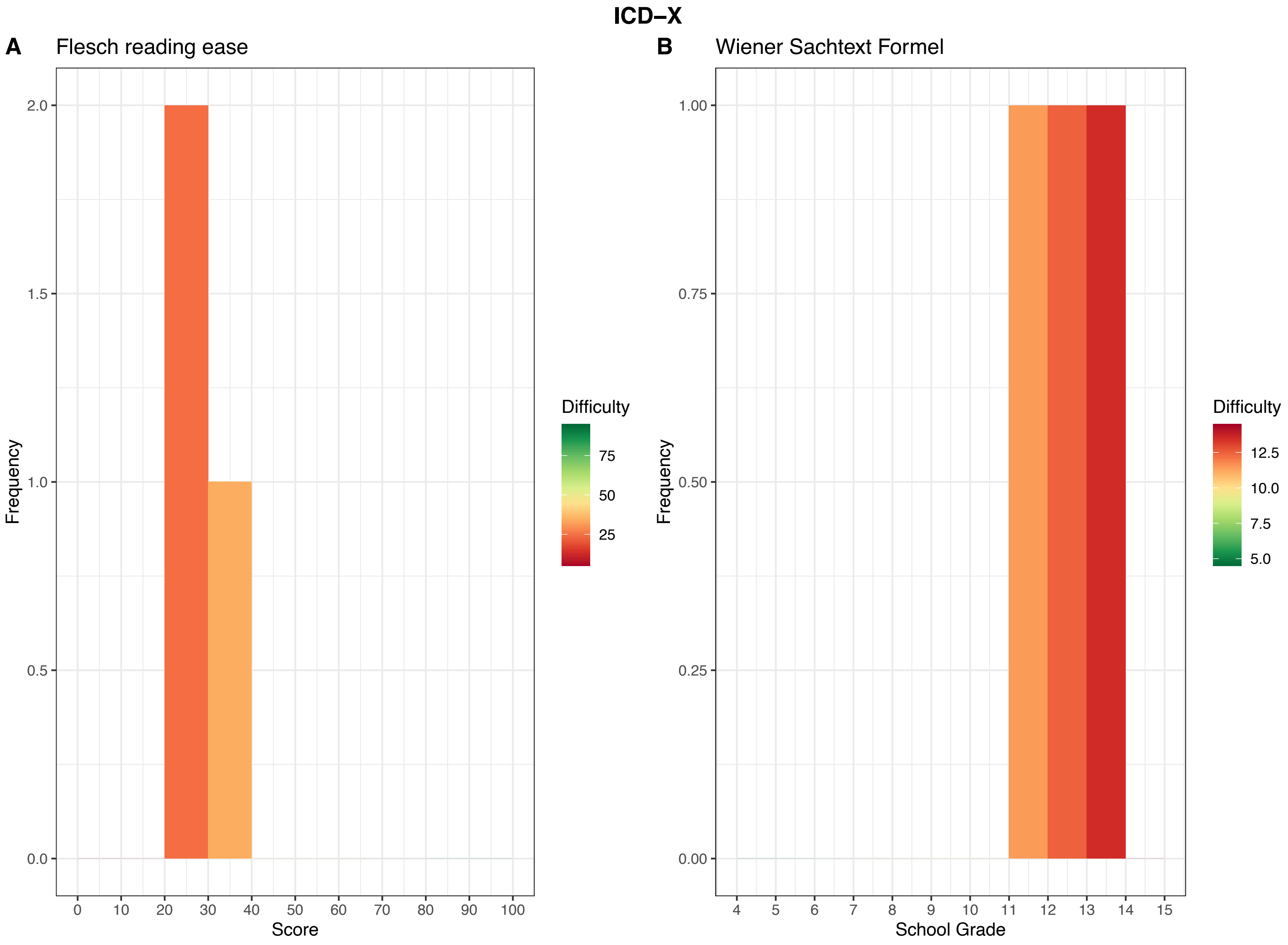

ICD–X
A
Flesch reading ease
Frequency
2.0
1.5
1.0
0.5
0.0
0 10 20 30 40 50 60 70 80 90 100
Score
Difficulty
75
50
25
B
Wiener Sachtext Formel
Frequency
1.00
0.75
0.50
0.25
0.00
4 5 6 7 8 9 10 11 12 13 14 15
School Grade
Difficulty
12.5
10.0
7.5
5.0

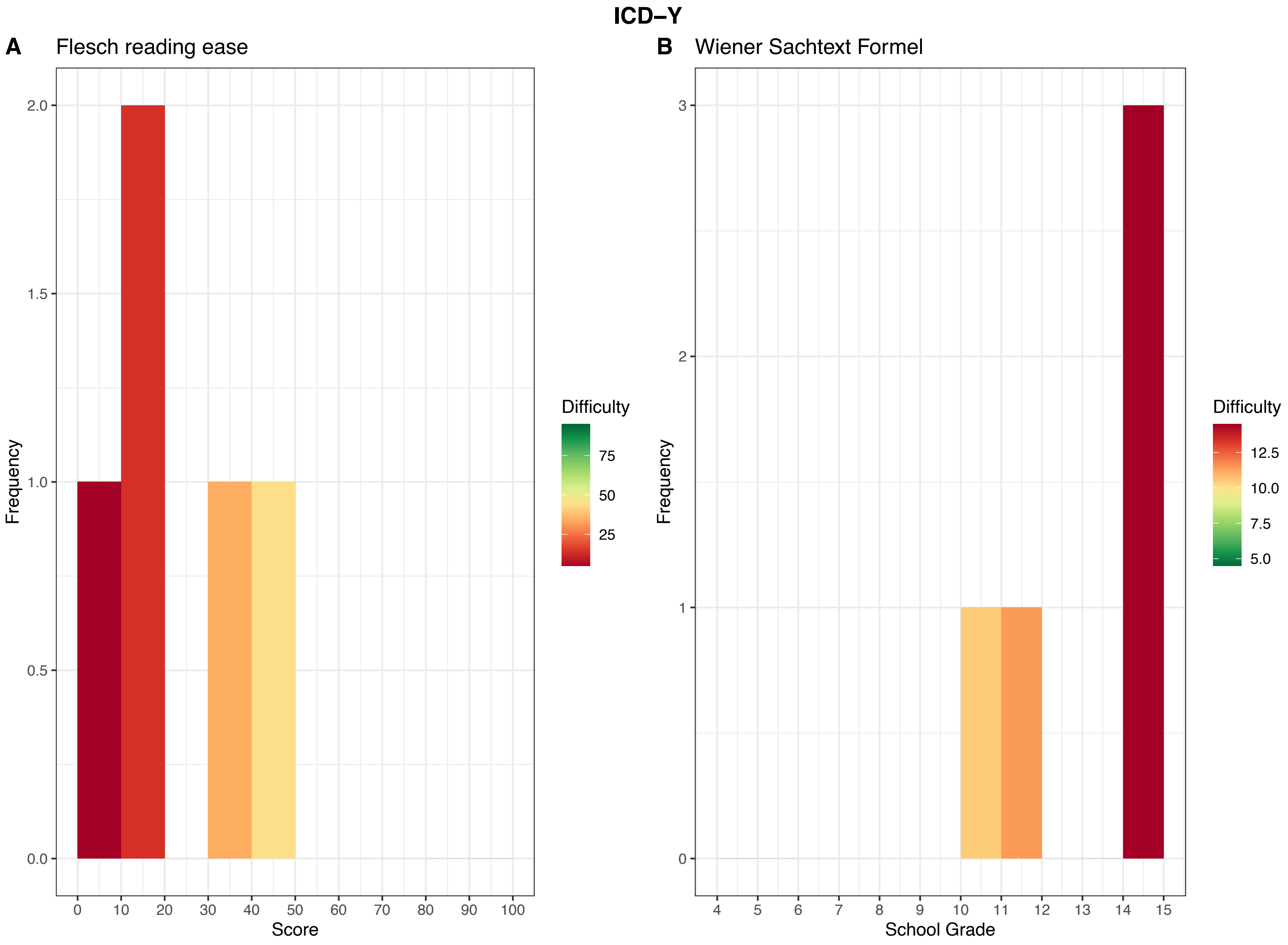
ICD–Y
A
Flesch reading ease
Frequency
2.0
1.5
1.0
0.5
0.0
0
10
20
30
40
50
60
70
80
90
100
Score
Difficulty
75
50
25
B
Wiener Sachtext Formel
Frequency
3
2
1
0
4
5
6
7
8
9
10
11
12
13
14
15
School Grade
Difficulty
12.5
10.0
7.5
5.0

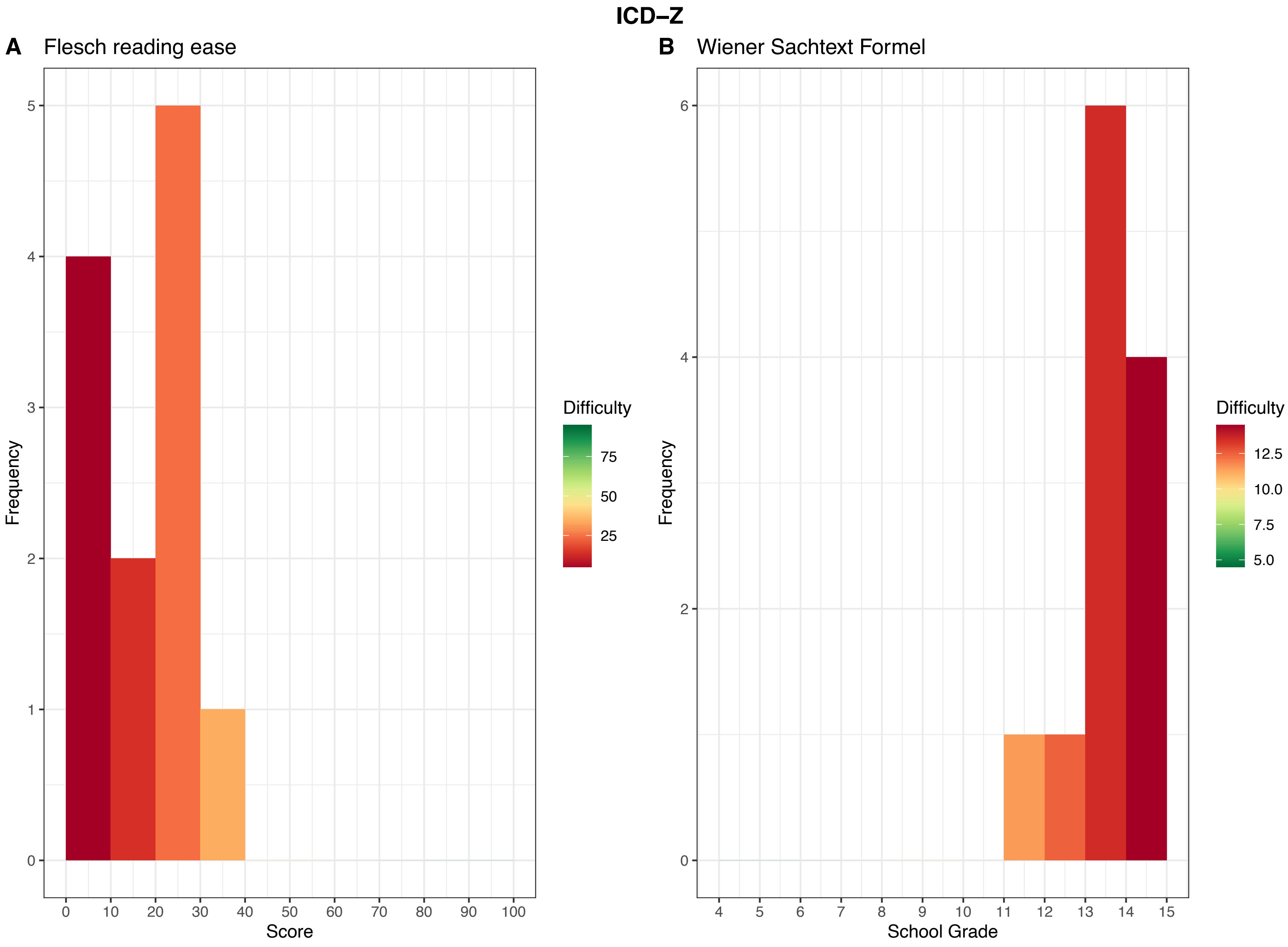
ICD–Z
A Flesch reading ease
Frequency
Score
0 10 20 30 40 50 60 70 80 90 100
0 1 2 3 4 5
Difficulty
75
50
25
B Wiener Sachtext Formel
Frequency
School Grade
4 5 6 7 8 9 10 11 12 13 14 15
0 2 4 6
Difficulty
12.5
10.0
7.5
5.0
