# Supplementary material for: Readability of English, German, and Russian Disease-Related Wikipedia Pages: Automated Computational Analysis
Source: J Med Internet Res. 2022 May 16;24(5):e36835. doi: 10.2196/36835 (PMC9152717; doi:10.2196/36835)
Supplement: Multimedia Appendix 6 [file jmir_v24i5e36835_app6.pdf]

Multimedia Appendix 6: Distributions of all computed readability metrics in Russian

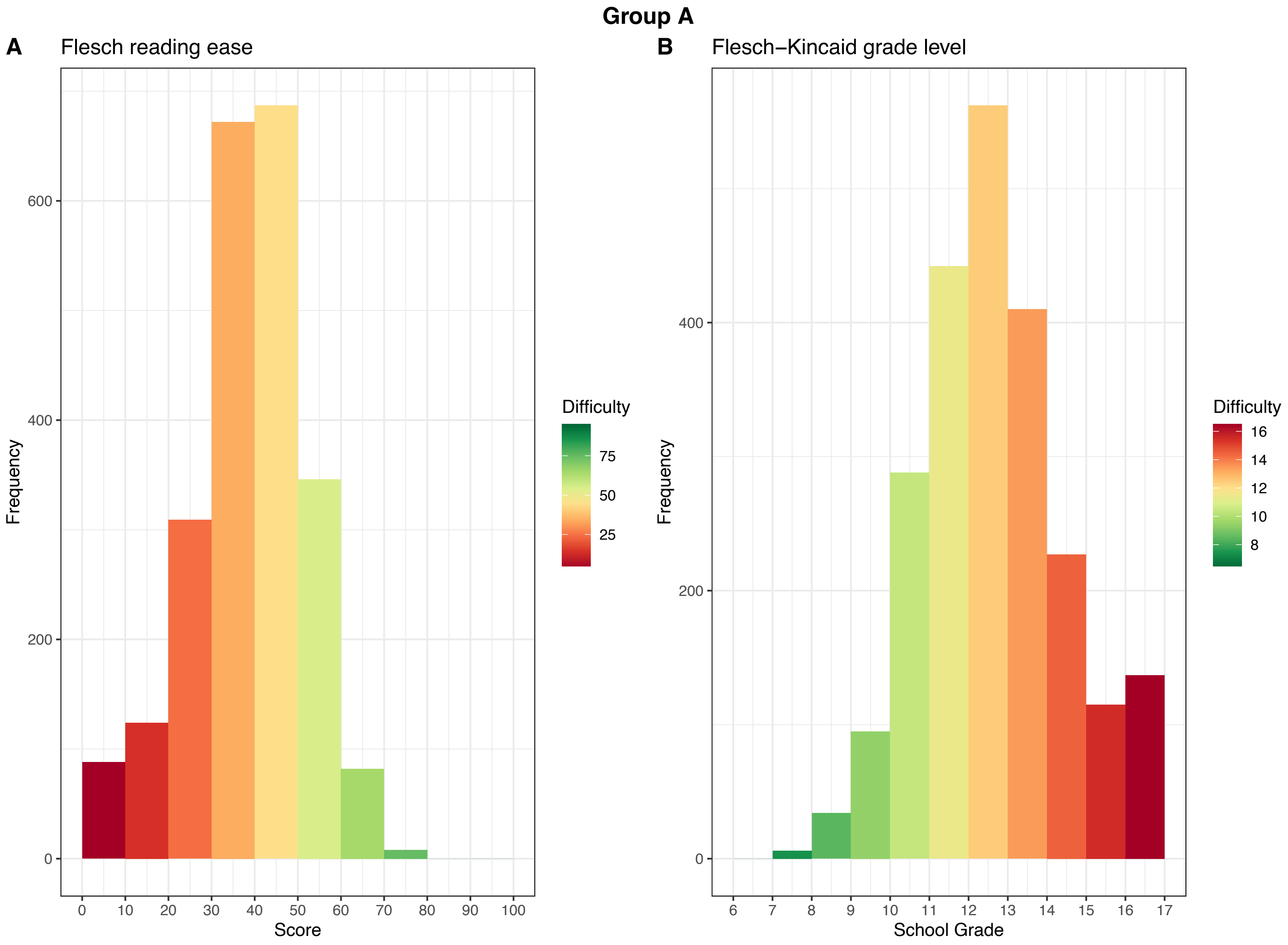

Group A
A
Flesch reading ease
Frequency
Score
0
200
400
600
0
10
20
30
40
50
60
70
80
90
100
Difficulty
75
50
25
B
Flesch–Kincaid grade level
Frequency
School Grade
0
200
400
6
7
8
9
10
11
12
13
14
15
16
17
Difficulty
16
14
12
10
8

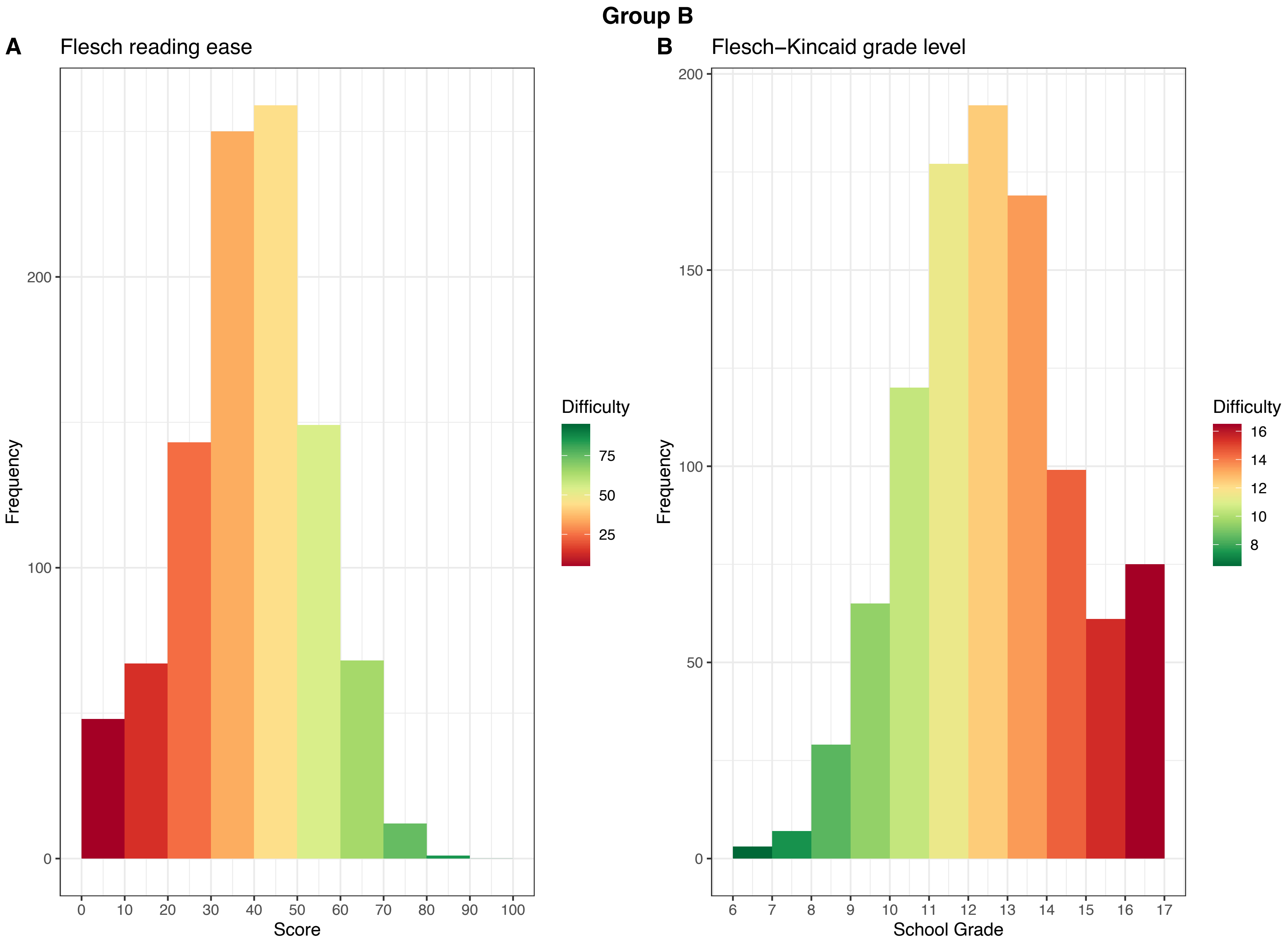
Group B
A
Flesch reading ease
Frequency
200
100
0
0 10 20 30 40 50 60 70 80 90 100
Score
Difficulty
75
50
25
B
Flesch−Kincaid grade level
Frequency
200
150
100
50
0
6 7 8 9 10 11 12 13 14 15 16 17
School Grade
Difficulty
16
14
12
10
8

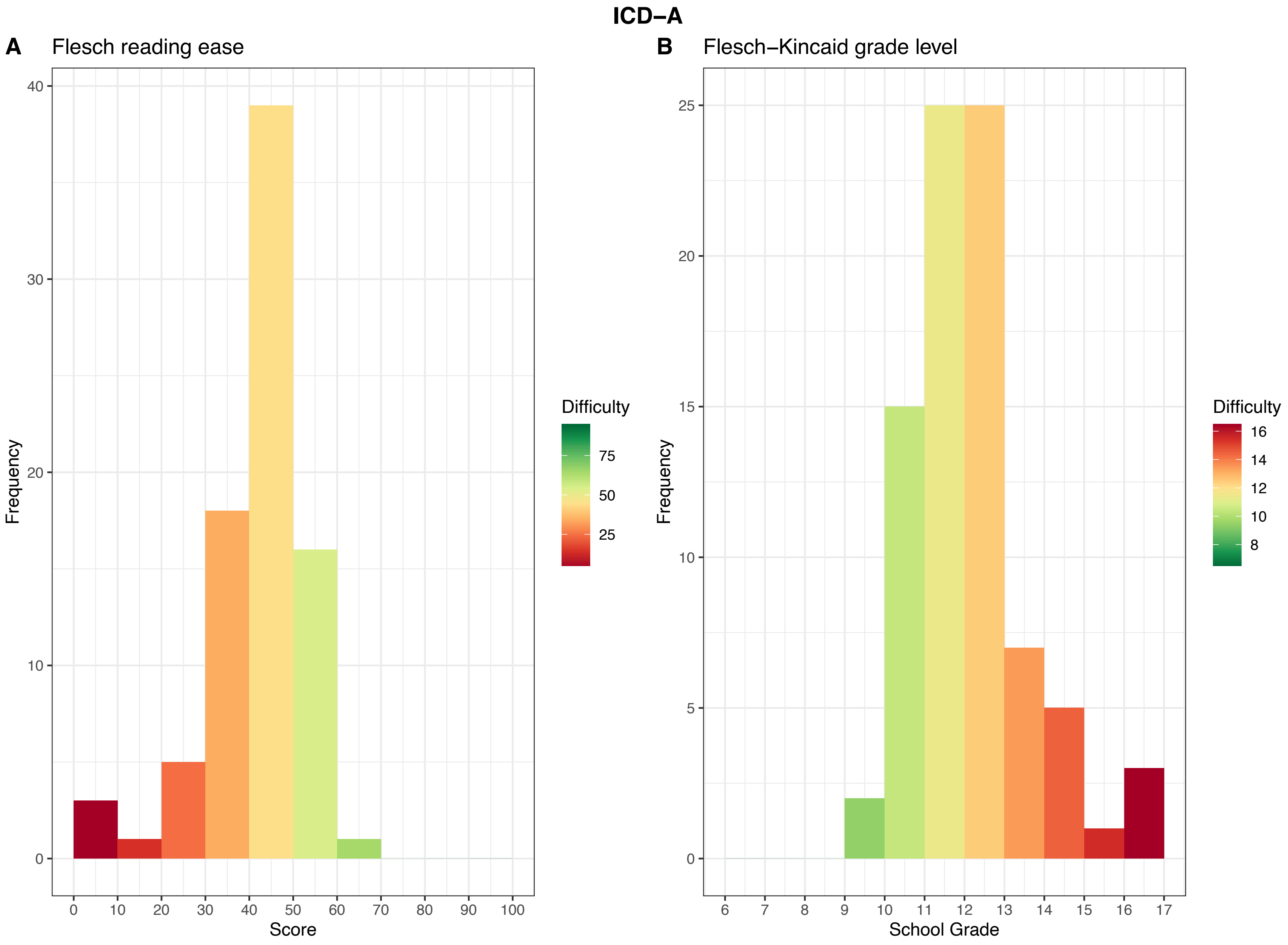
ICD–A
A
Flesch reading ease
Frequency
Score
Difficulty
75
50
25
0
10
20
30
40
50
60
70
80
90
100
B
Flesch–Kincaid grade level
Frequency
School Grade
Difficulty
16
14
12
10
8
25
20
15
5
6
7
9
11
13
17

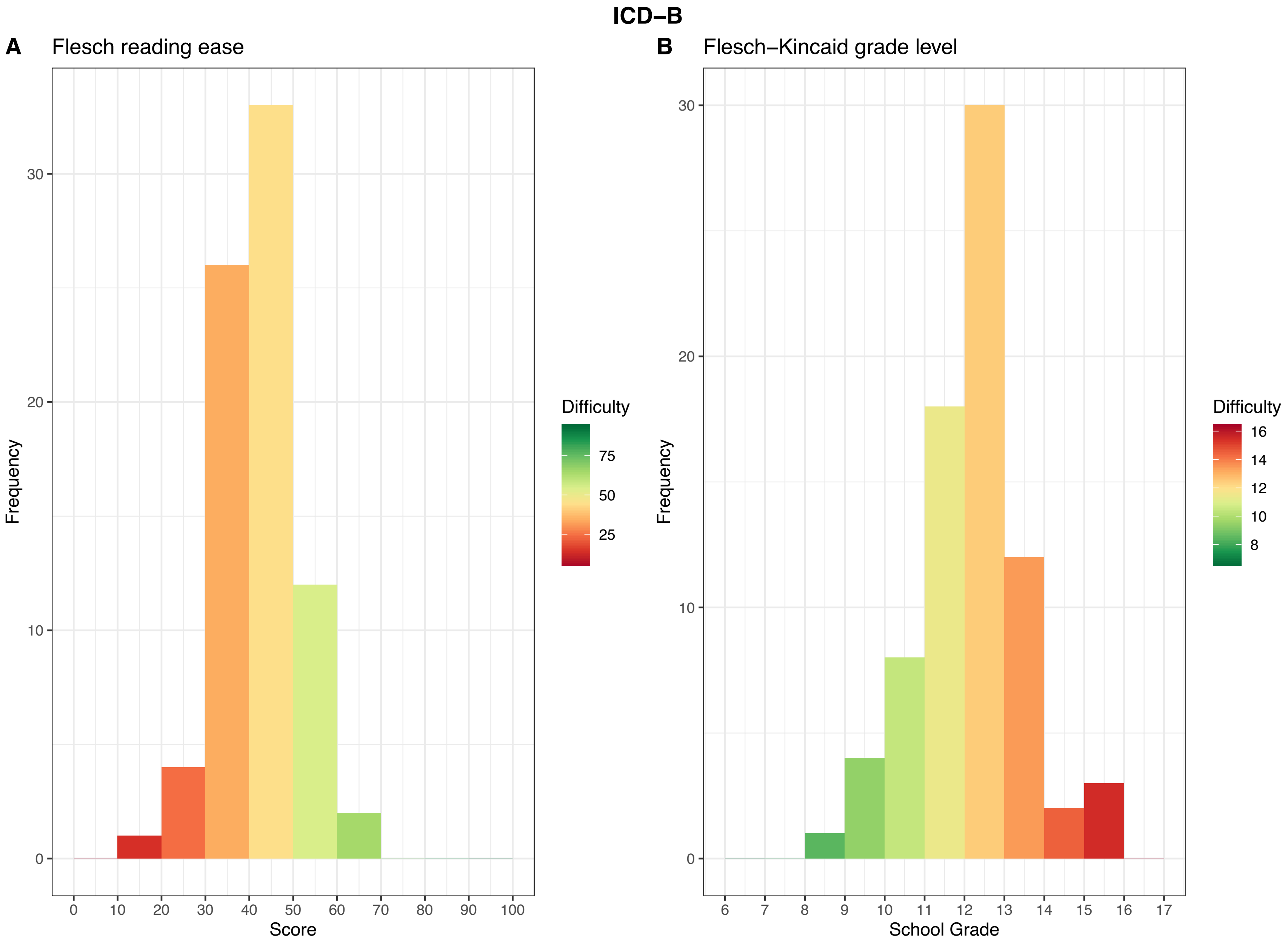

ICD–B
A
Flesch reading ease
Frequency
Score
0
10
20
30
40
50
60
70
80
90
100
Difficulty
75
50
25
B
Flesch–Kincaid grade level
Frequency
School Grade
6
7
8
9
10
11
12
13
14
15
16
17
Difficulty
16
14
12
10
8

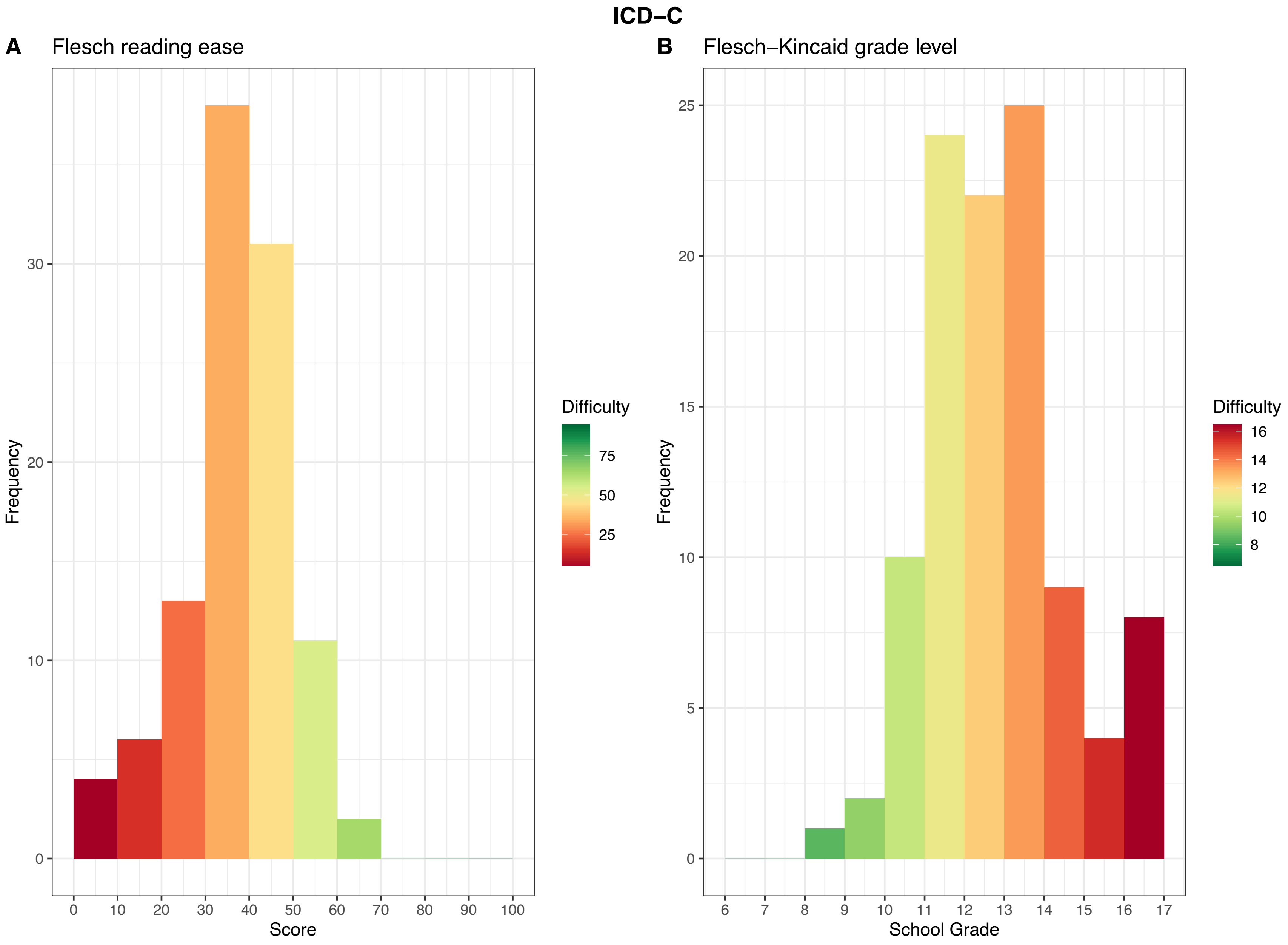
ICD–C
A Flesch reading ease
Frequency
Score
Difficulty
75
50
25
B Flesch–Kincaid grade level
Frequency
School Grade
Difficulty
16
14
12
10
8

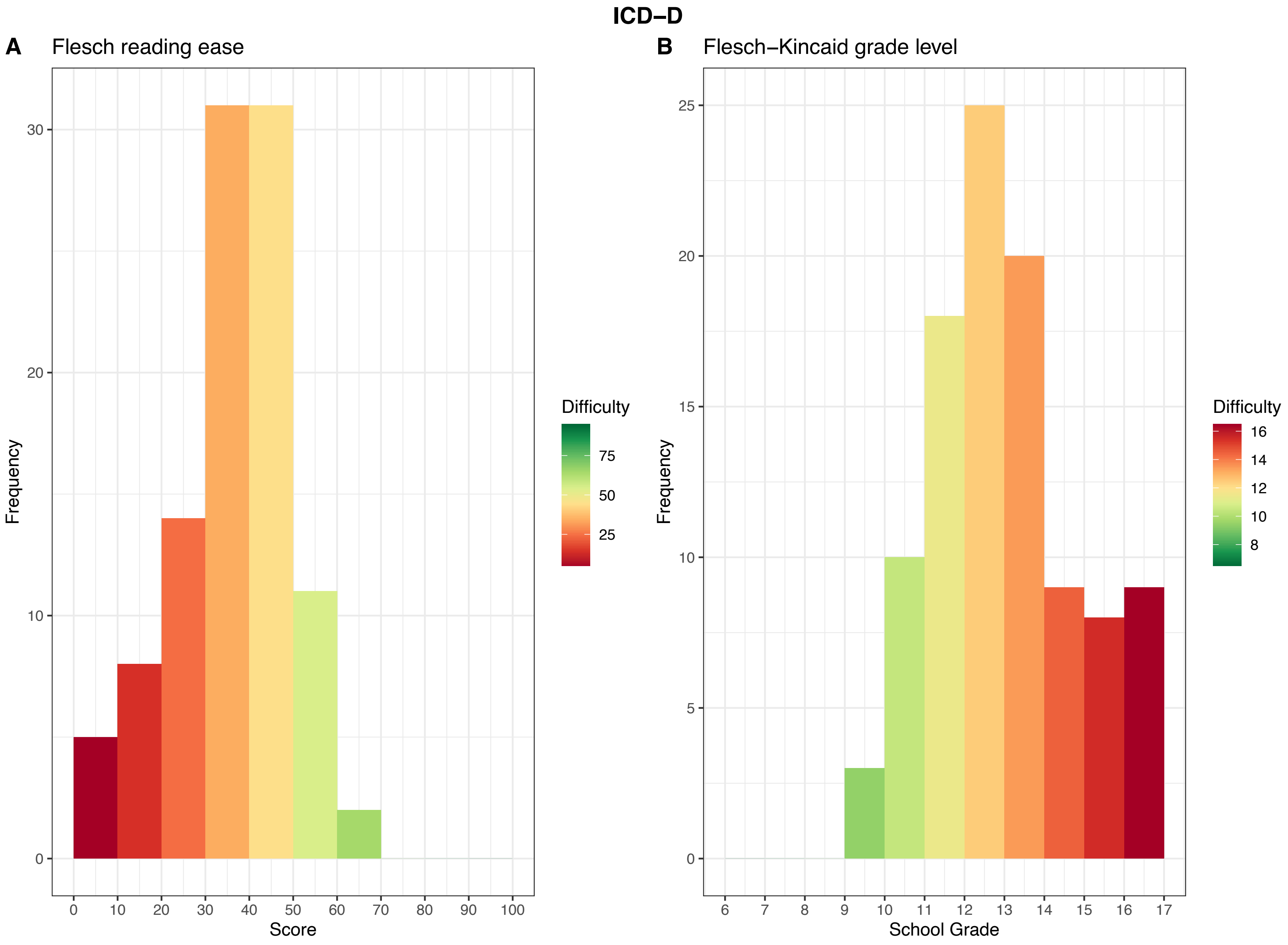
ICD–D
A Flesch reading ease
Frequency
Score
Difficulty
75
50
25
B Flesch–Kincaid grade level
Frequency
School Grade
Difficulty
16
14
12
10
8

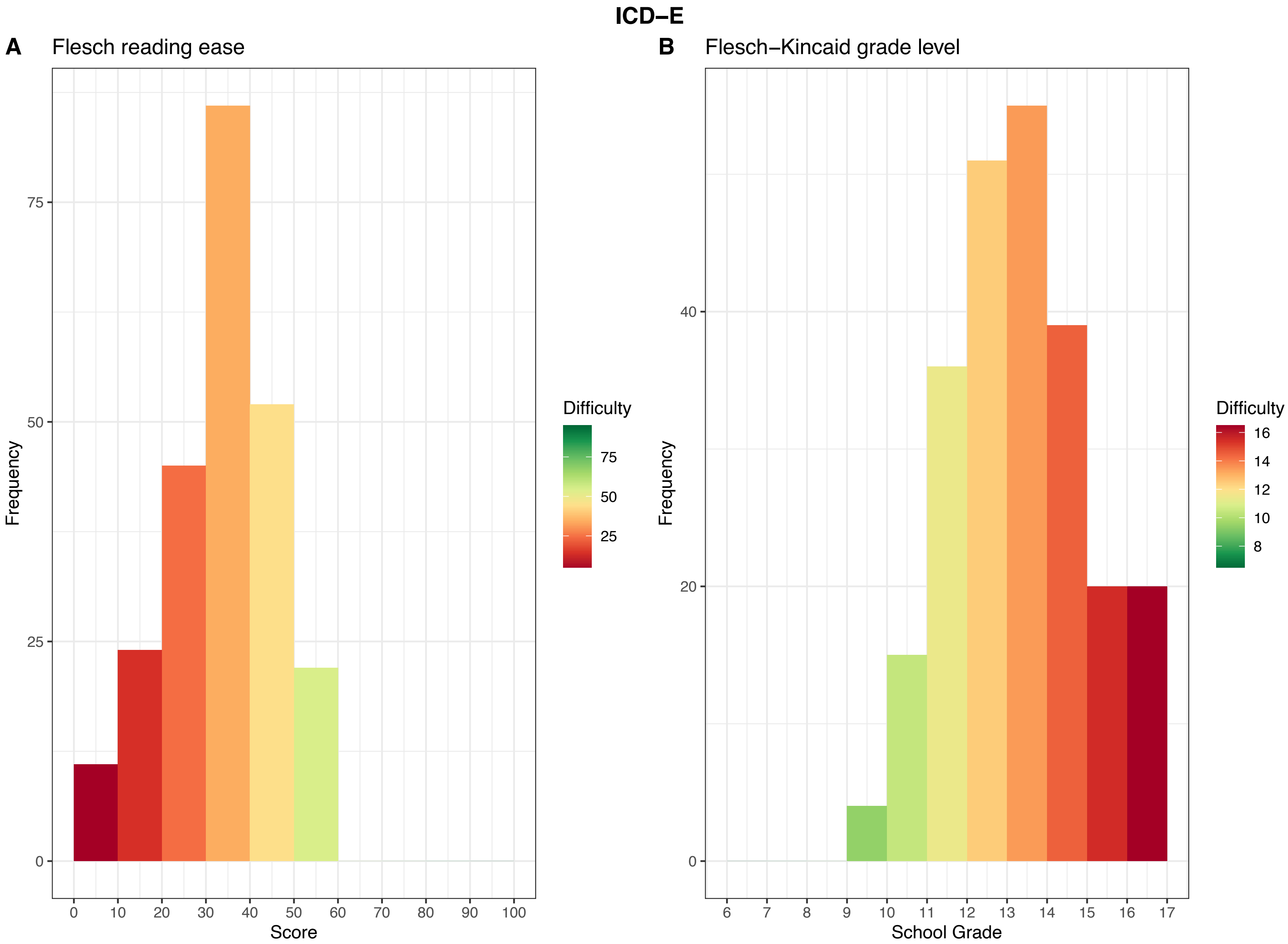

ICD–E
A Flesch reading ease
Frequency
Score
Difficulty
75
50
25
B Flesch–Kincaid grade level
Frequency
School Grade
Difficulty
16
14
12
10
8

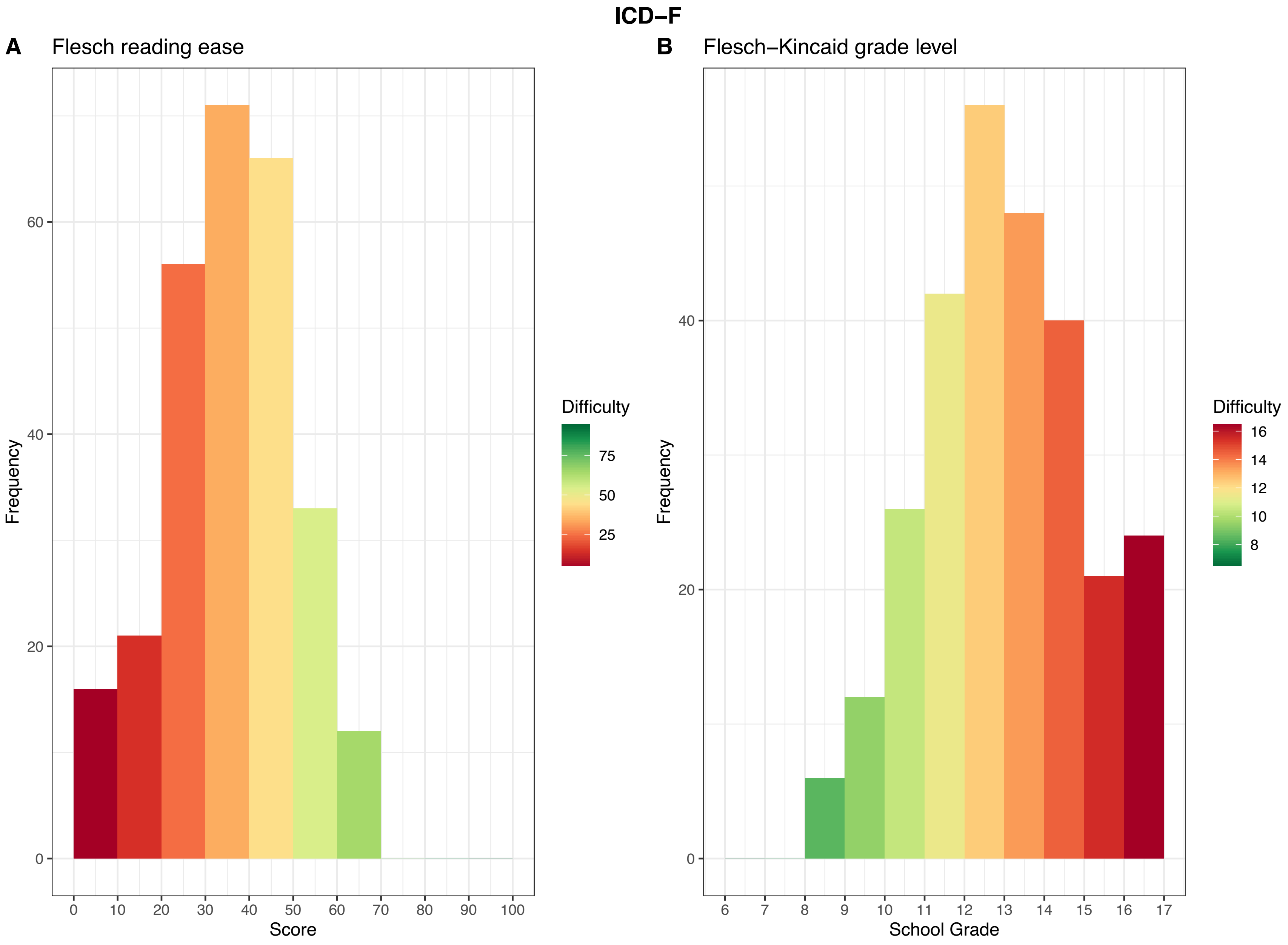
ICD–F
A
Flesch reading ease
Frequency
Score
0
20
40
60
0 10 20 30 40 50 60 70 80 90 100
Difficulty
75
50
25
B
Flesch–Kincaid grade level
Frequency
School Grade
0
20
40
6 7 8 9 10 11 12 13 14 15 16 17
Difficulty
16
14
12
10
8

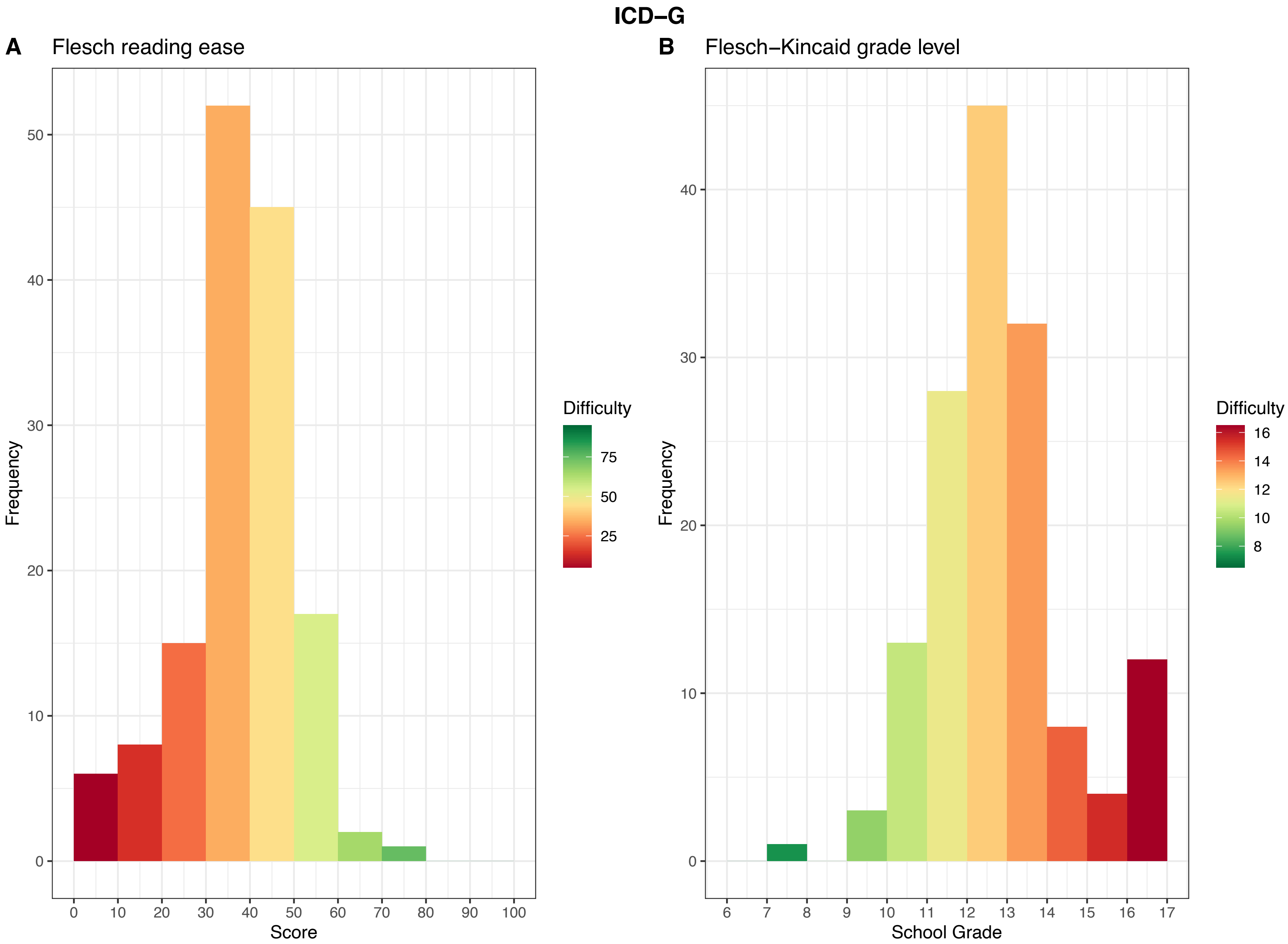
ICD–G
A
Flesch reading ease
Frequency
Score
Difficulty
75
50
25
B
Flesch–Kincaid grade level
Frequency
School Grade
Difficulty
16
14
12
10
8

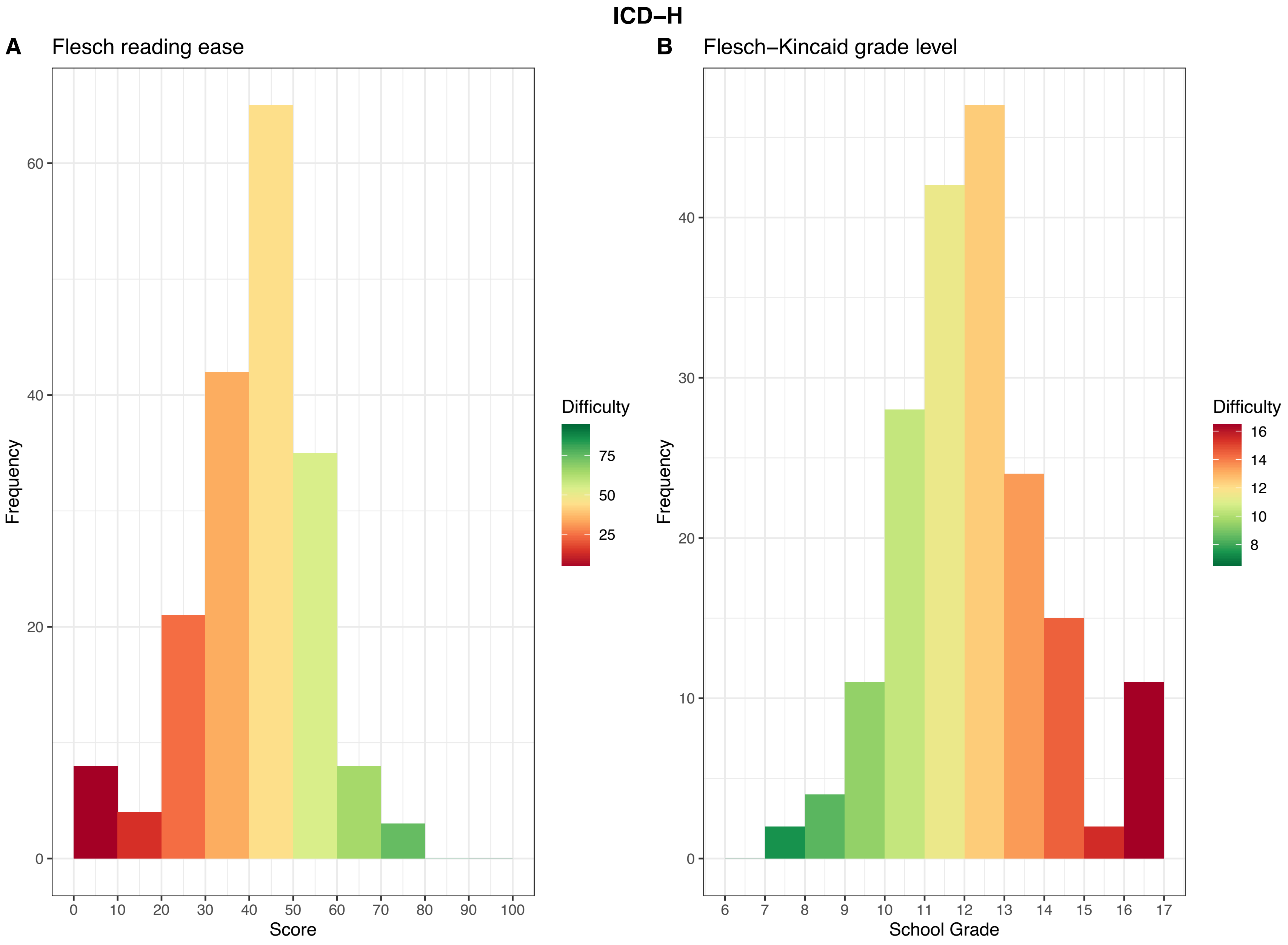

ICD−H
A
Flesch reading ease
Frequency
60
40
20
0
0
10
20
30
40
50
60
70
80
90
100
Score
Difficulty
75
50
25
B
Flesch−Kincaid grade level
Frequency
40
30
20
10
0
6
7
8
9
10
11
12
13
14
15
16
17
School Grade
Difficulty
16
14
12
10
8

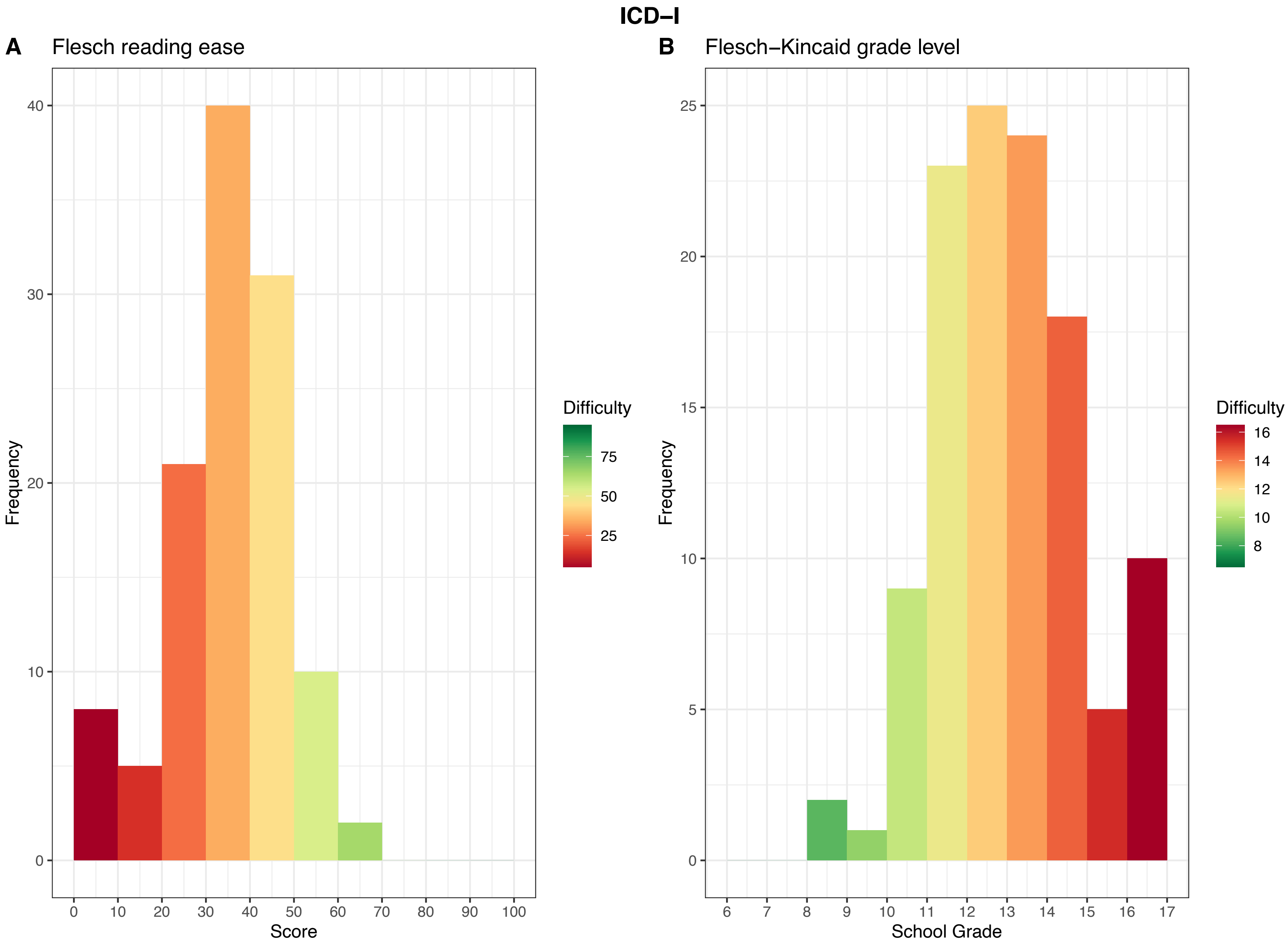

ICD–I
A
Flesch reading ease
Frequency
Score
Difficulty
75
50
25
B
Flesch–Kincaid grade level
Frequency
School Grade
Difficulty
16
14
12
10
8

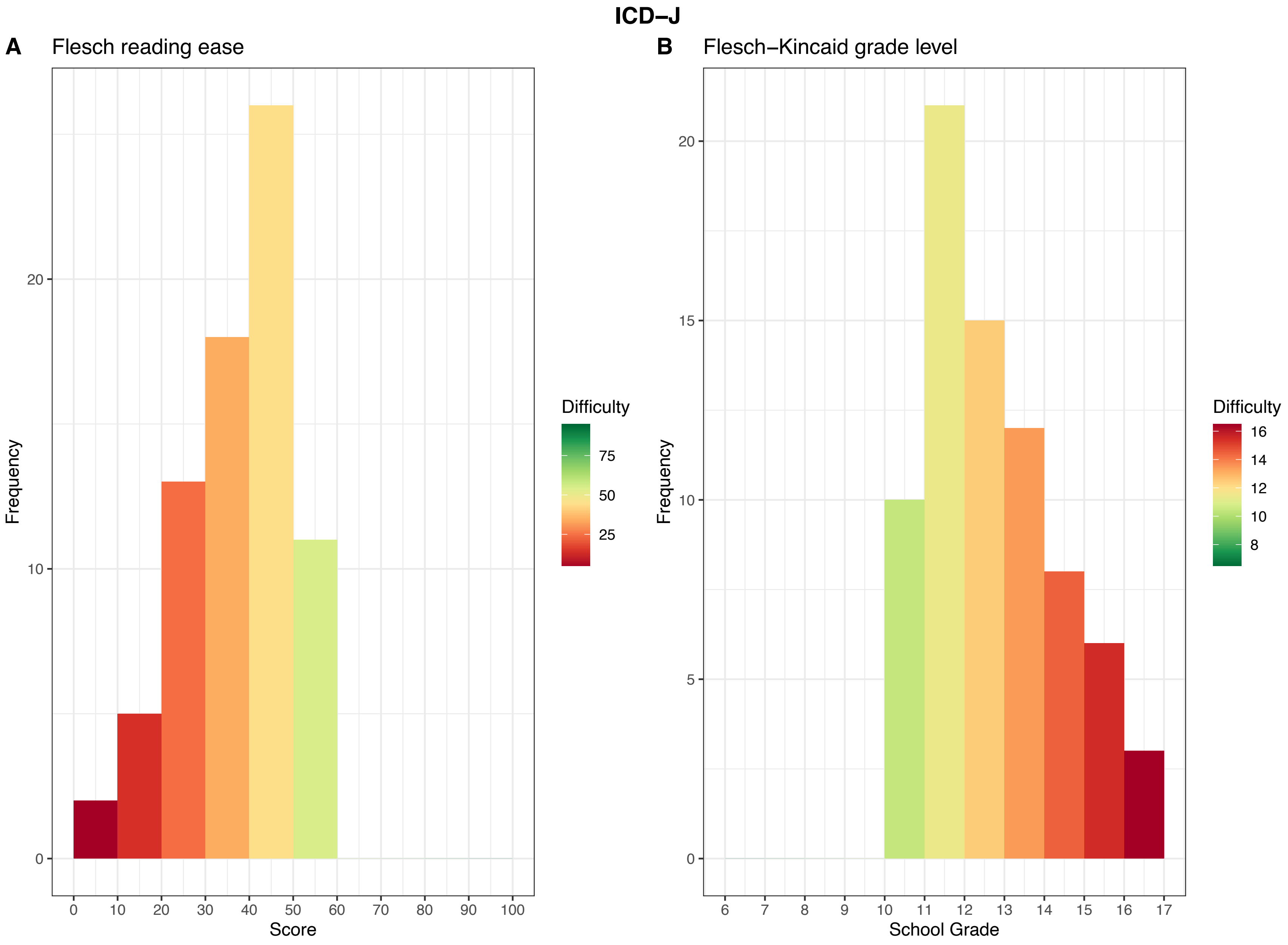
ICD–J
A
Flesch reading ease
Frequency
Score
Difficulty
75
50
25
B
Flesch–Kincaid grade level
Frequency
School Grade
Difficulty
16
14
12
10
8

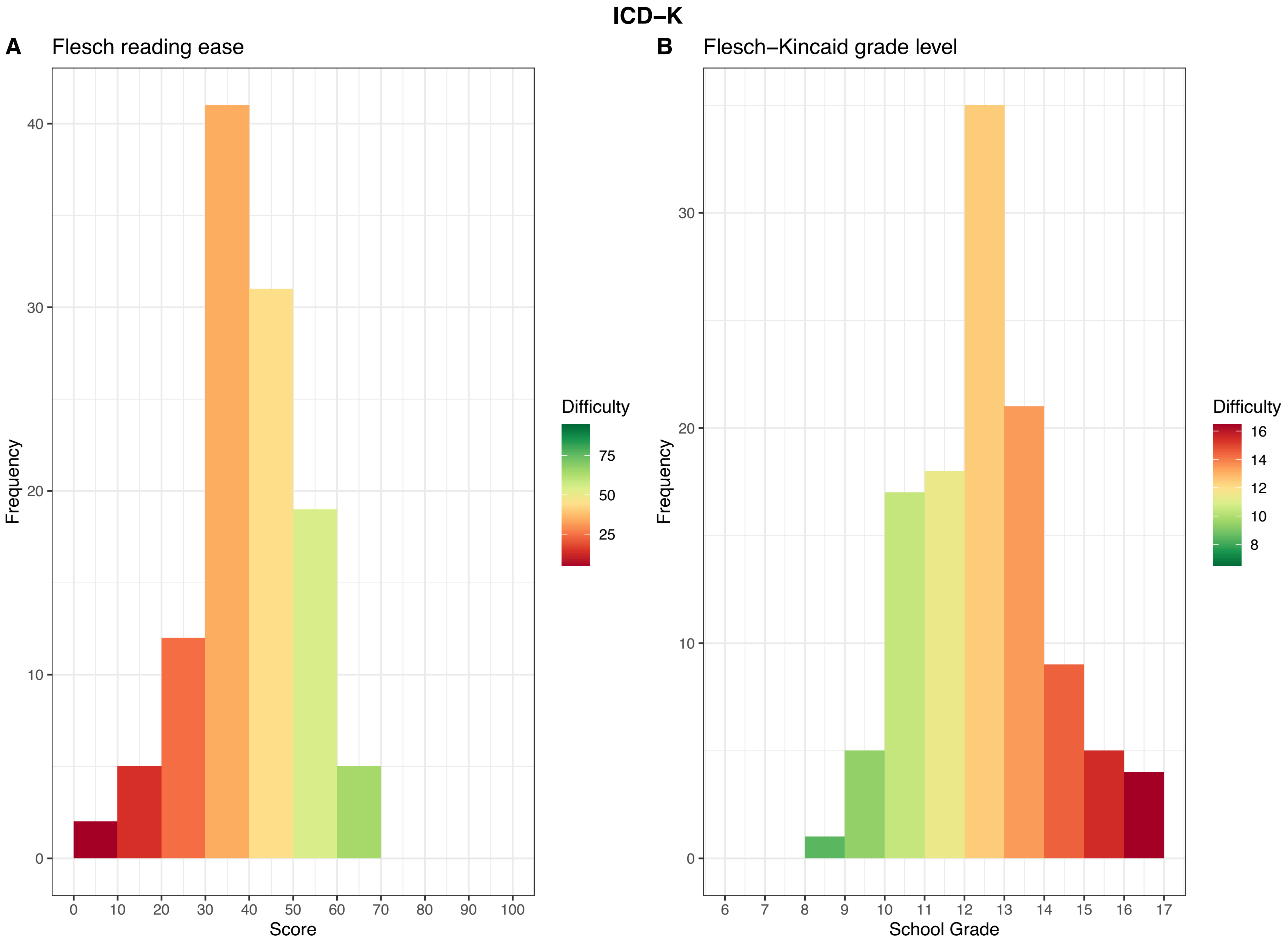
ICD–K
A
Flesch reading ease
Frequency
Score
0
10
20
30
40
0
10
20
30
40
50
60
70
80
90
100
Difficulty
75
50
25
B
Flesch–Kincaid grade level
Frequency
School Grade
0
10
20
30
6
7
8
9
10
11
12
13
14
15
16
17
Difficulty
16
14
12
10
8

ICD–L

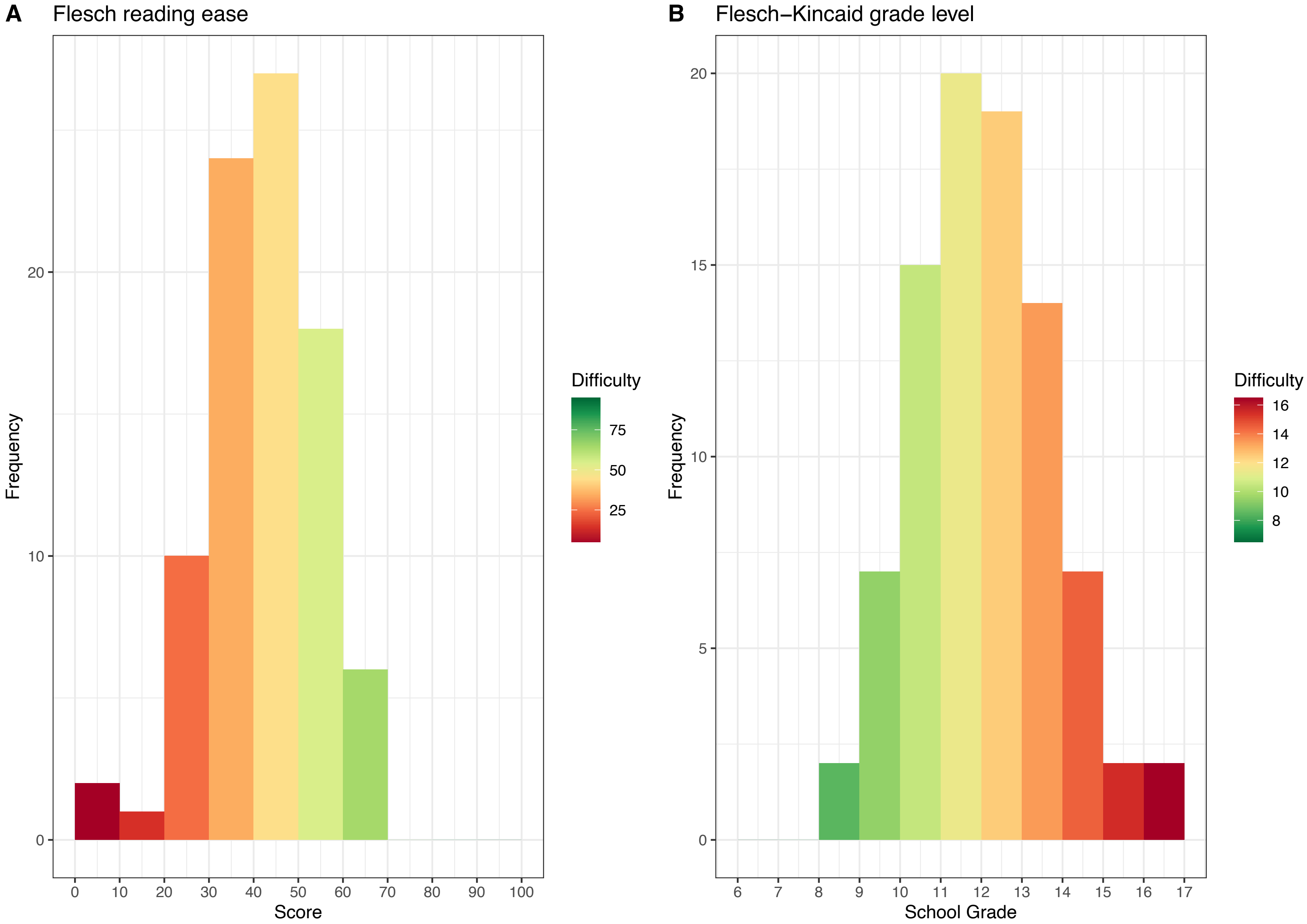

A
Flesch reading ease
Frequency
0
10
20
0
10
20
30
40
50
60
70
80
90
100
Score
Difficulty
75
50
25
B
Flesch–Kincaid grade level
Frequency
0
5
10
15
20
6
7
8
9
10
11
12
13
14
15
16
17
School Grade
Difficulty
16
14
12
10
8

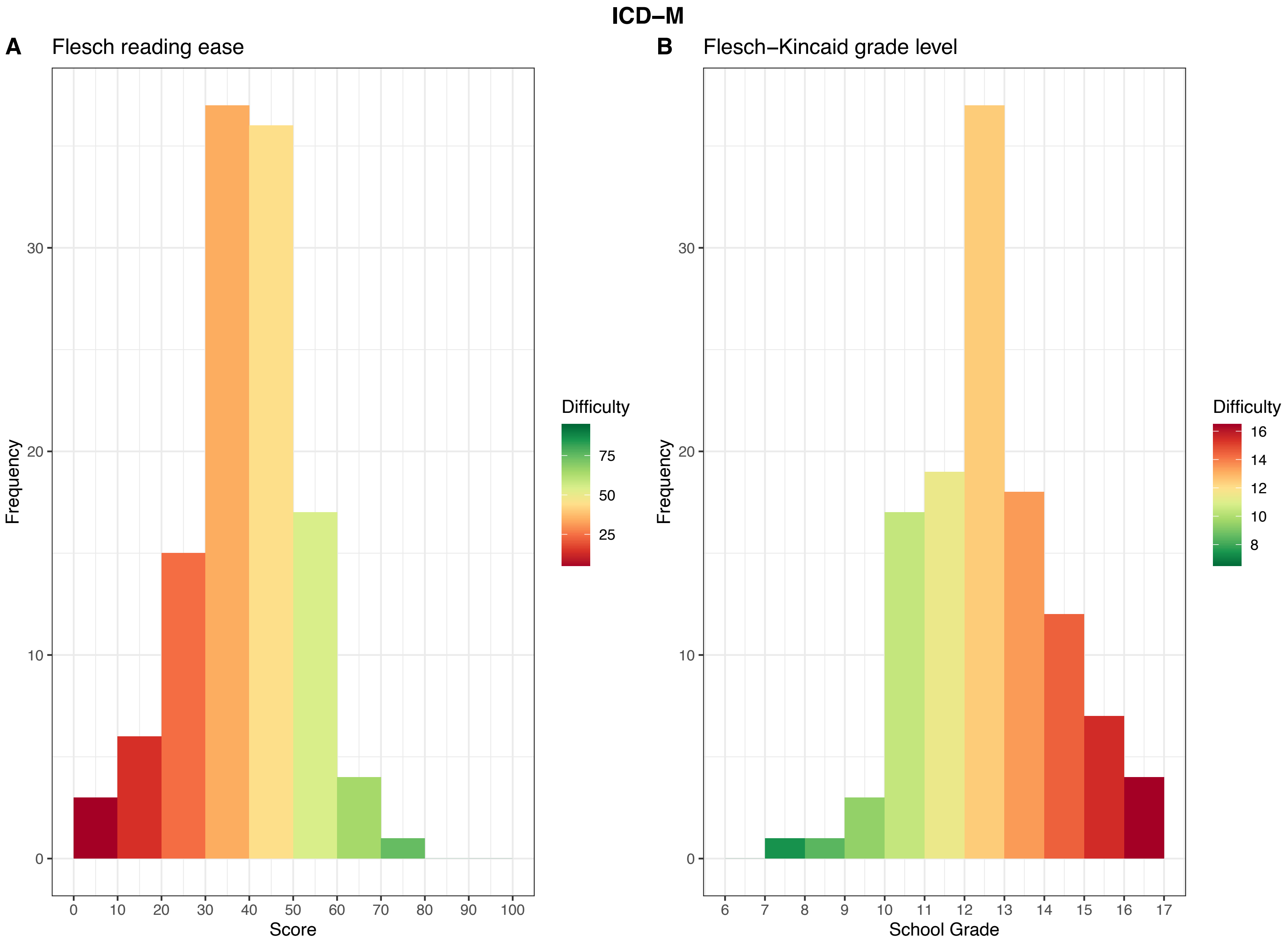

ICD–M
A
Flesch reading ease
Frequency
Score
Difficulty
75
50
25
B
Flesch–Kincaid grade level
Frequency
School Grade
Difficulty
16
14
12
10
8

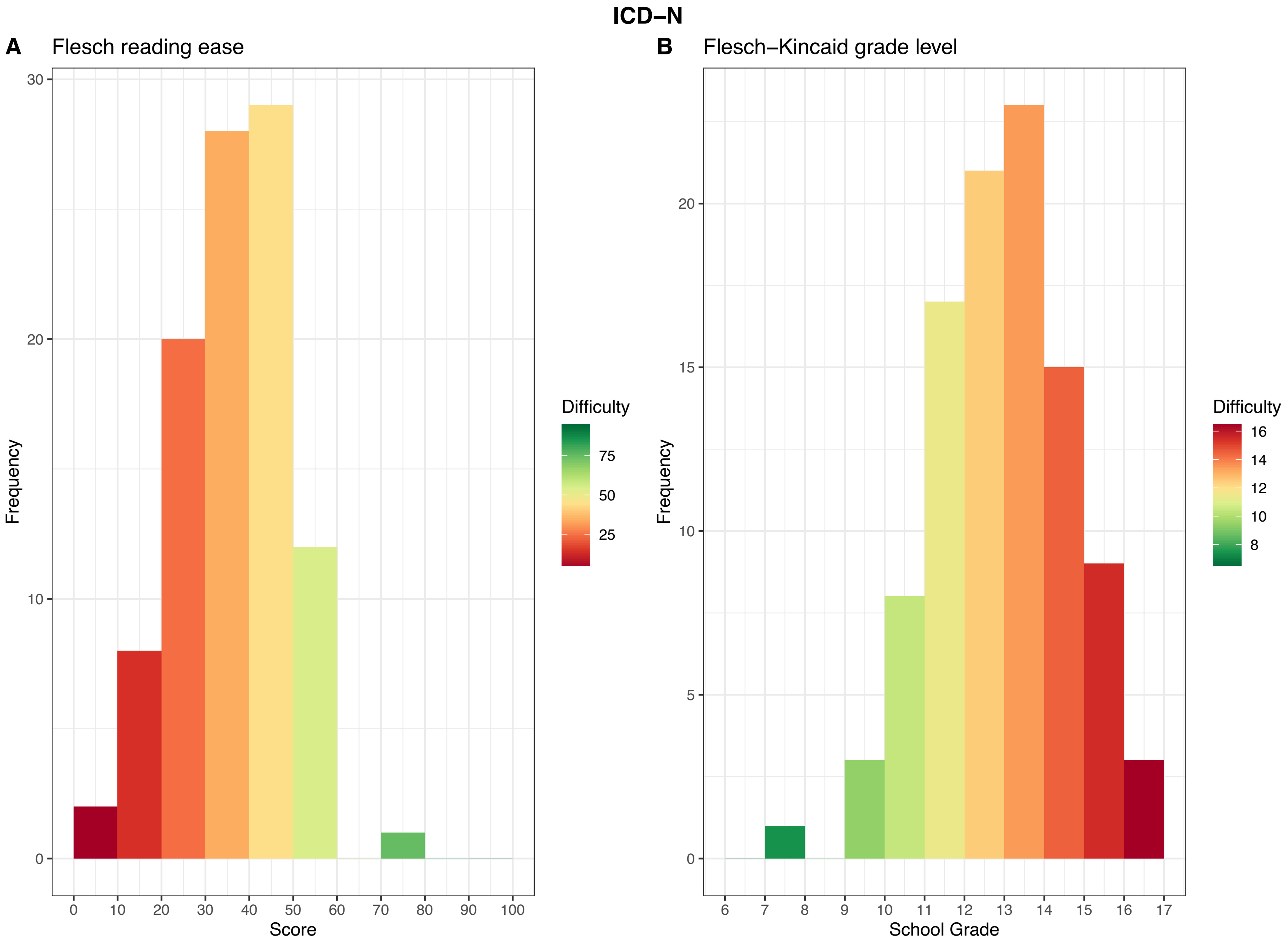
ICD–N
A
Flesch reading ease
Frequency
Score
Difficulty
75
50
25
B
Flesch–Kincaid grade level
Frequency
School Grade
Difficulty
16
14
12
10
8

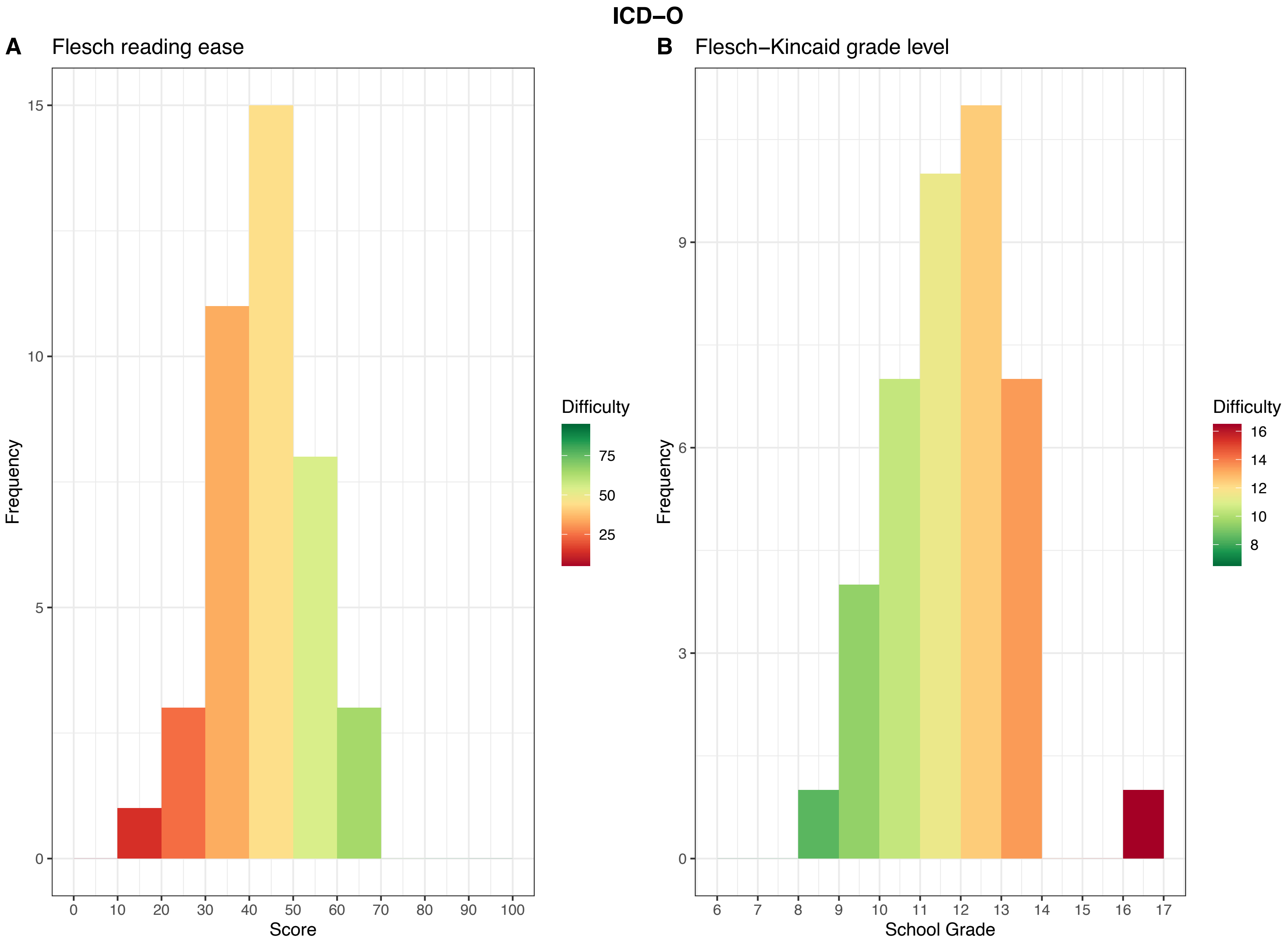
ICD−O
A
Flesch reading ease
Frequency
Score
Difficulty
75
50
25
B
Flesch−Kincaid grade level
Frequency
School Grade
Difficulty
16
14
12
10
8

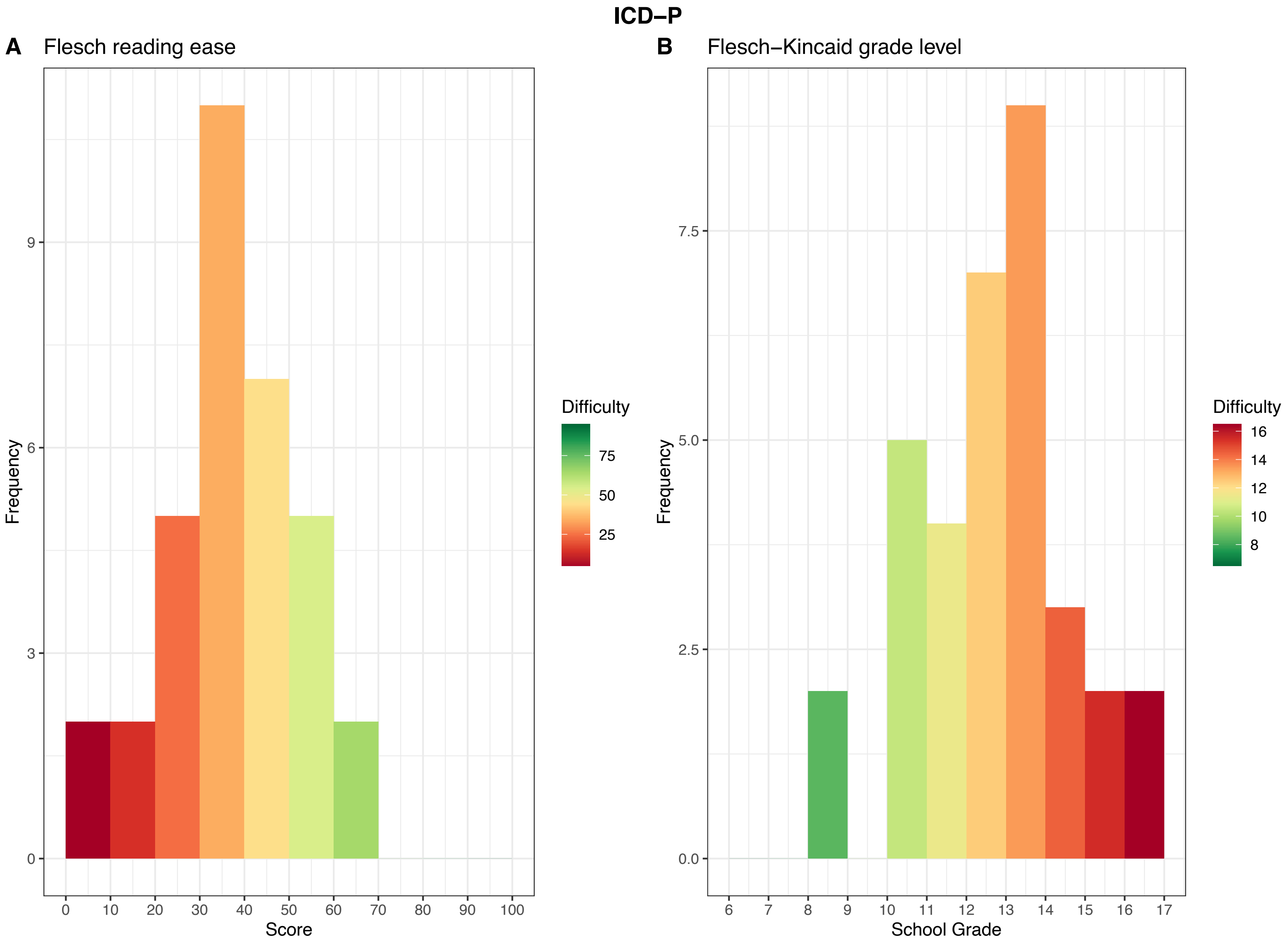
ICD–P
A Flesch reading ease
Frequency
Score
0
3
6
9
0 10 20 30 40 50 60 70 80 90 100
Difficulty
75
50
25
B Flesch–Kincaid grade level
Frequency
School Grade
0.0
2.5
5.0
7.5
6 7 8 9 10 11 12 13 14 15 16 17
Difficulty
16
14
12
10
8

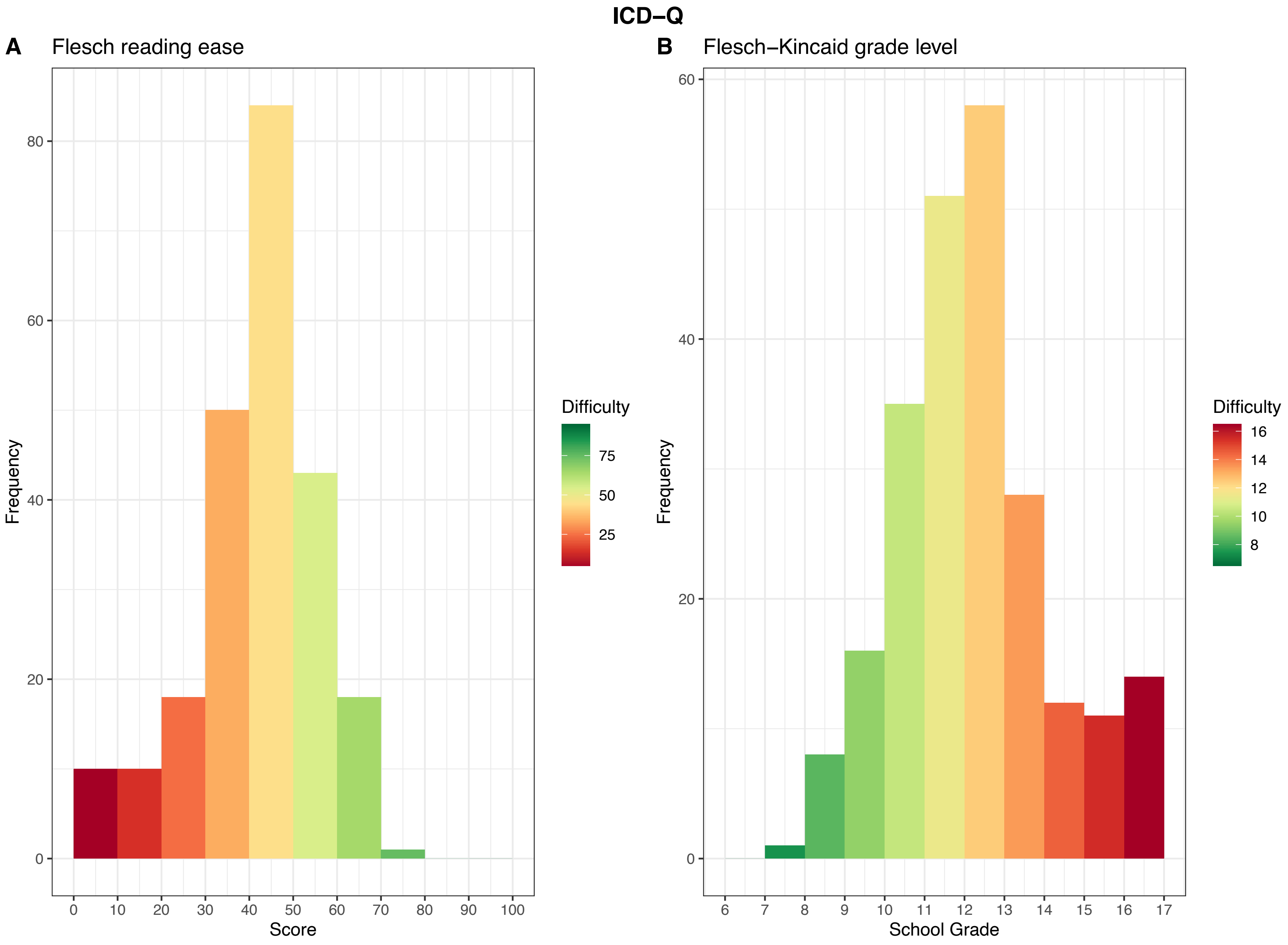

ICD–Q
A
Flesch reading ease
Frequency
Score
0
20
40
60
80
0
10
20
30
40
50
60
70
80
90
100
Difficulty
75
50
25
B
Flesch–Kincaid grade level
Frequency
School Grade
0
20
40
60
6
7
8
9
10
11
12
13
14
15
16
17
Difficulty
16
14
12
10
8

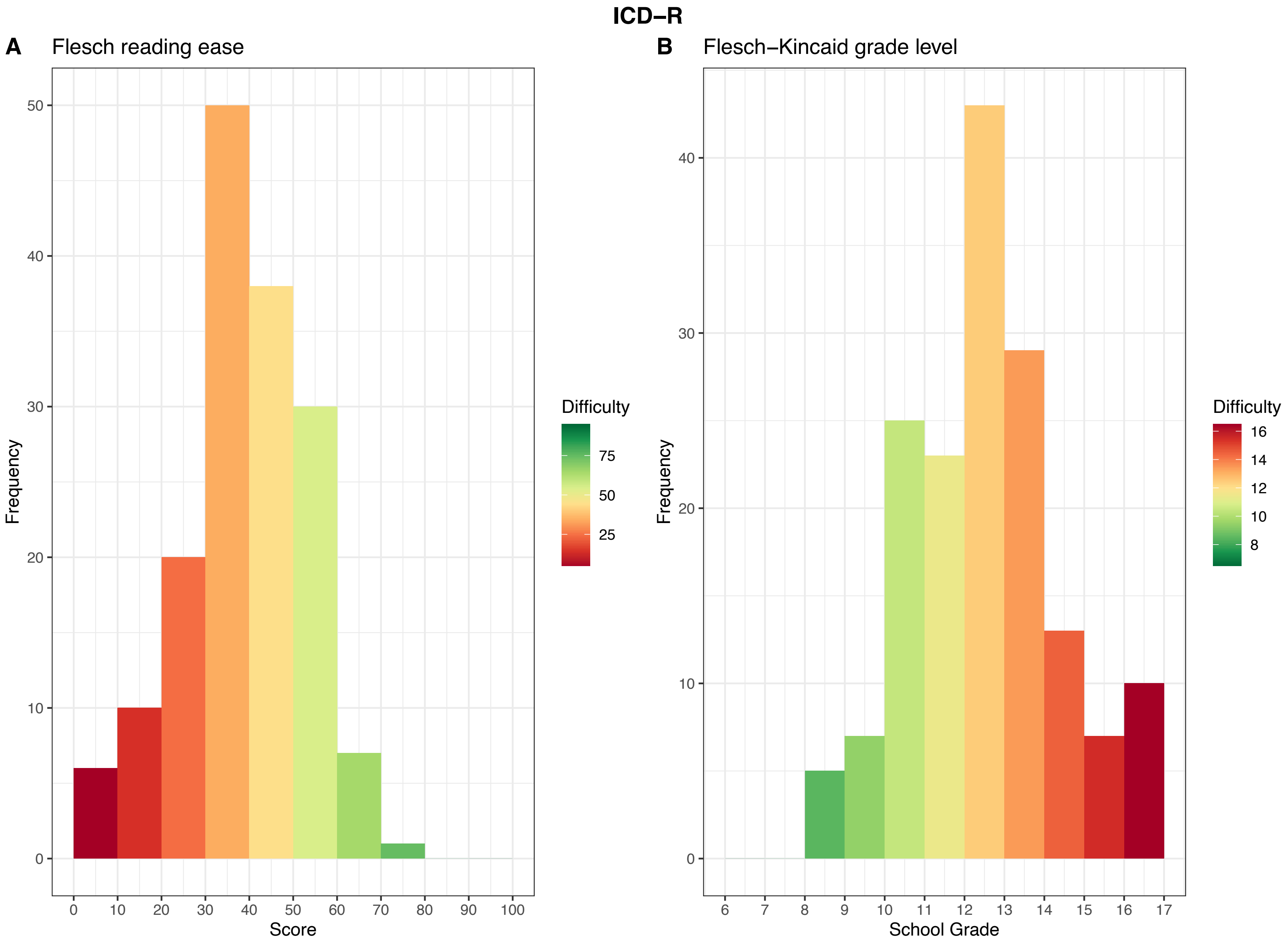

ICD–R
A
Flesch reading ease
Frequency
Score
0
10
20
30
40
50
Difficulty
75
50
25
B
Flesch–Kincaid grade level
School Grade
6
7
8
9
10
11
12
13
14
15
16
17
Difficulty
16
14
12
10
8

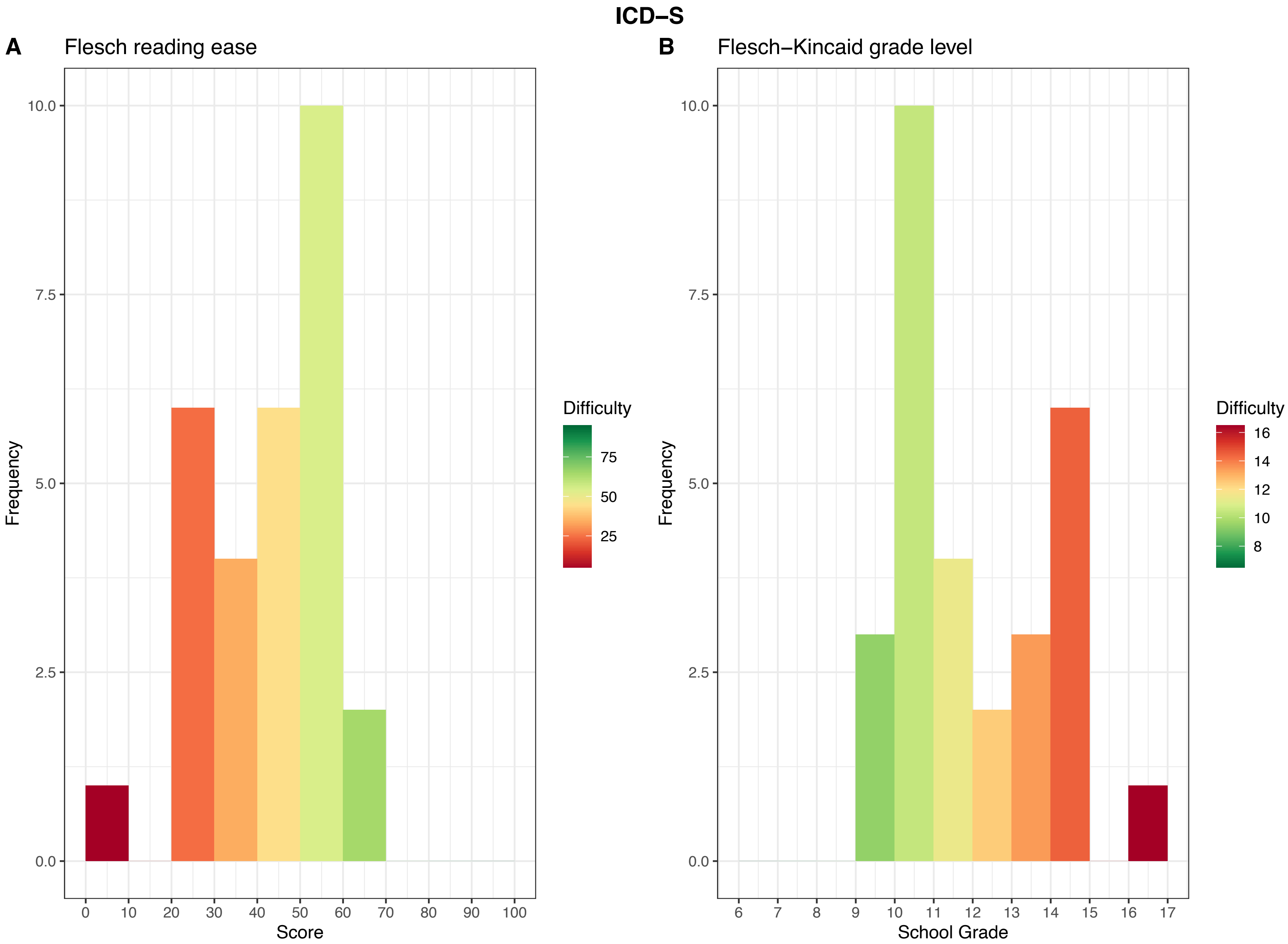
ICD–S
A
Flesch reading ease
Frequency
10.0
7.5
5.0
2.5
0.0
0
10
20
30
40
50
60
70
80
90
100
Score
Difficulty
75
50
25
B
Flesch–Kincaid grade level
Frequency
10.0
7.5
5.0
2.5
0.0
6
7
8
9
10
11
12
13
14
15
16
17
School Grade
Difficulty
16
14
12
10
8

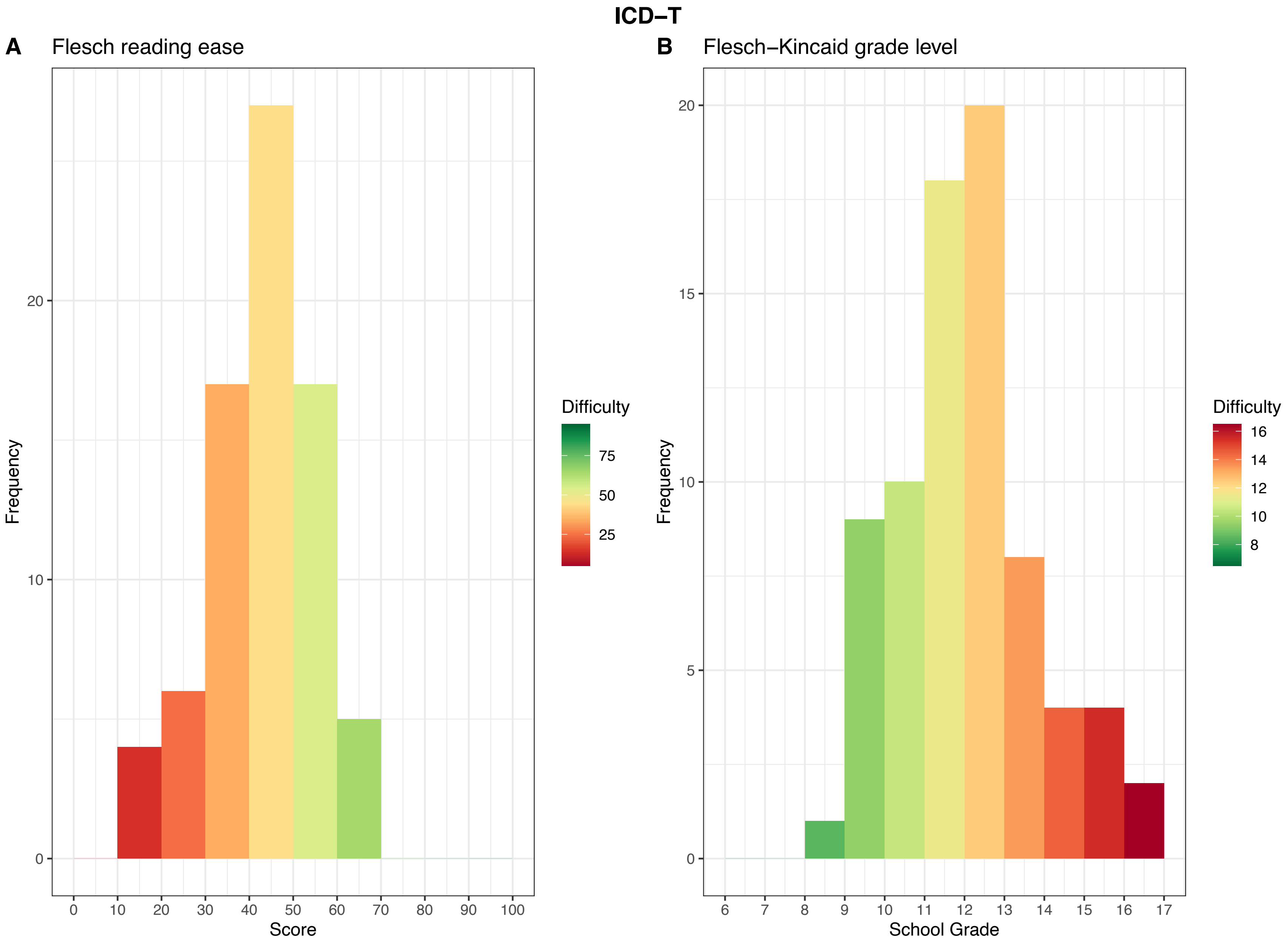
ICD–T
A
Flesch reading ease
Frequency
Score
0
10
20
30
40
50
60
70
80
90
100
Difficulty
75
50
25
B
Flesch–Kincaid grade level
Frequency
School Grade
6
7
8
9
10
11
12
13
14
15
16
17
Difficulty
16
14
12
10
8

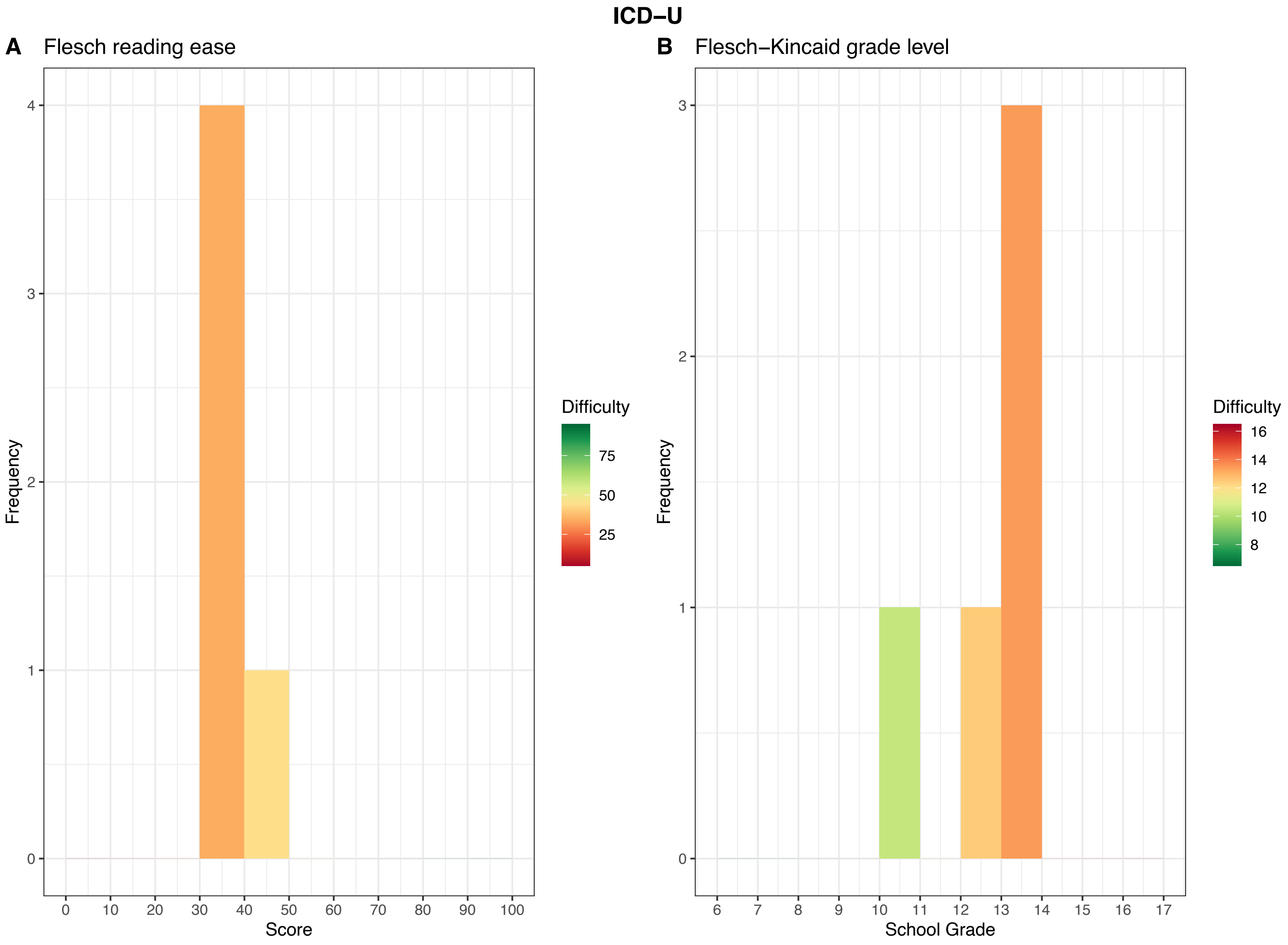
ICD−U
A Flesch reading ease
Frequency
Score
Difficulty
75
50
25
0
10
20
30
40
50
60
70
80
90
100
B Flesch−Kincaid grade level
Frequency
School Grade
Difficulty
16
14
12
10
8

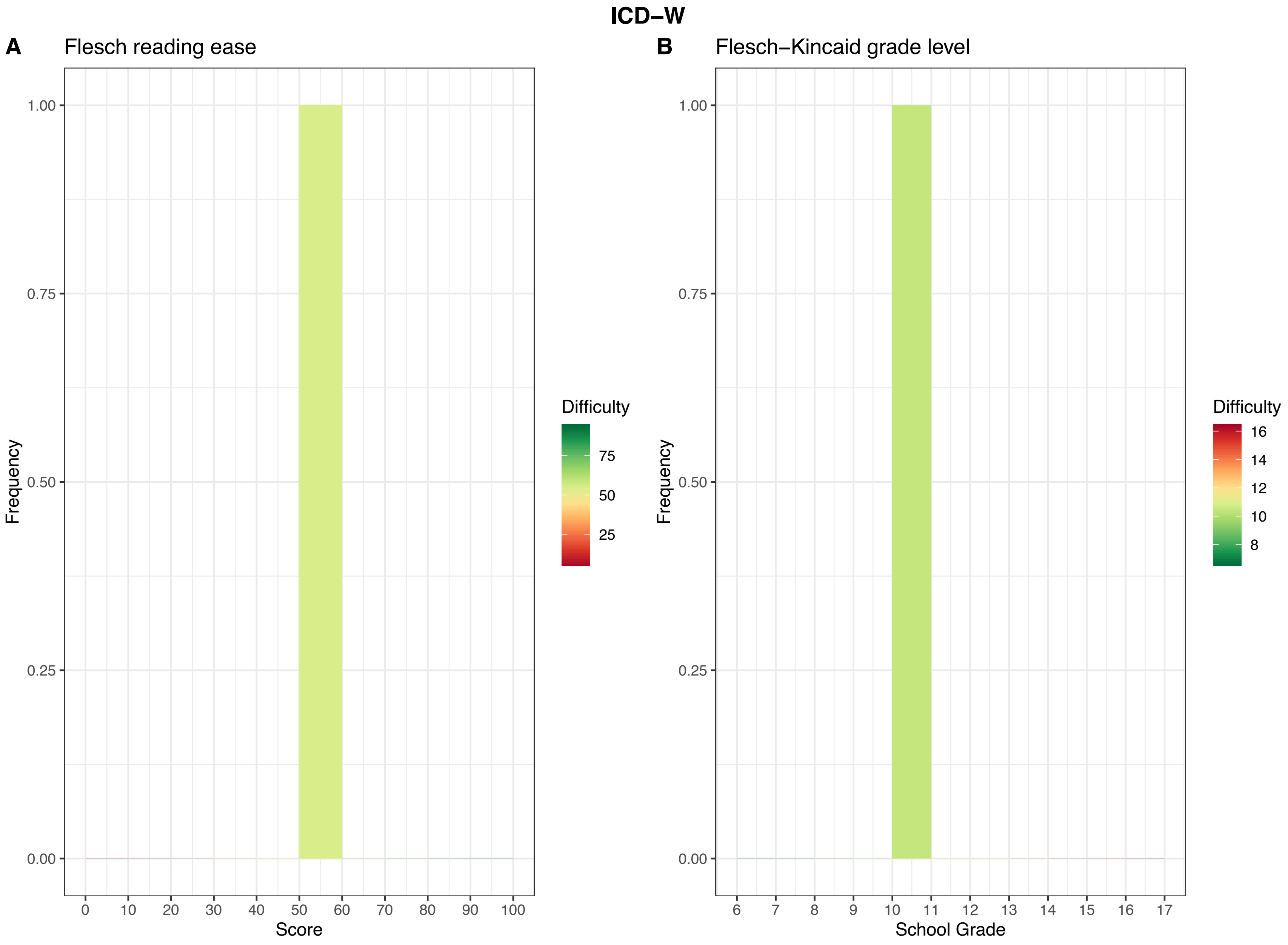
ICD–W
A
Flesch reading ease
Frequency
1.00
0.75
0.50
0.25
0.00
0
10
20
30
40
50
60
70
80
90
100
Score
Difficulty
75
50
25
B
Flesch–Kincaid grade level
Frequency
1.00
0.75
0.50
0.25
0.00
6
7
8
9
10
11
12
13
14
15
16
17
School Grade
Difficulty
16
14
12
10
8

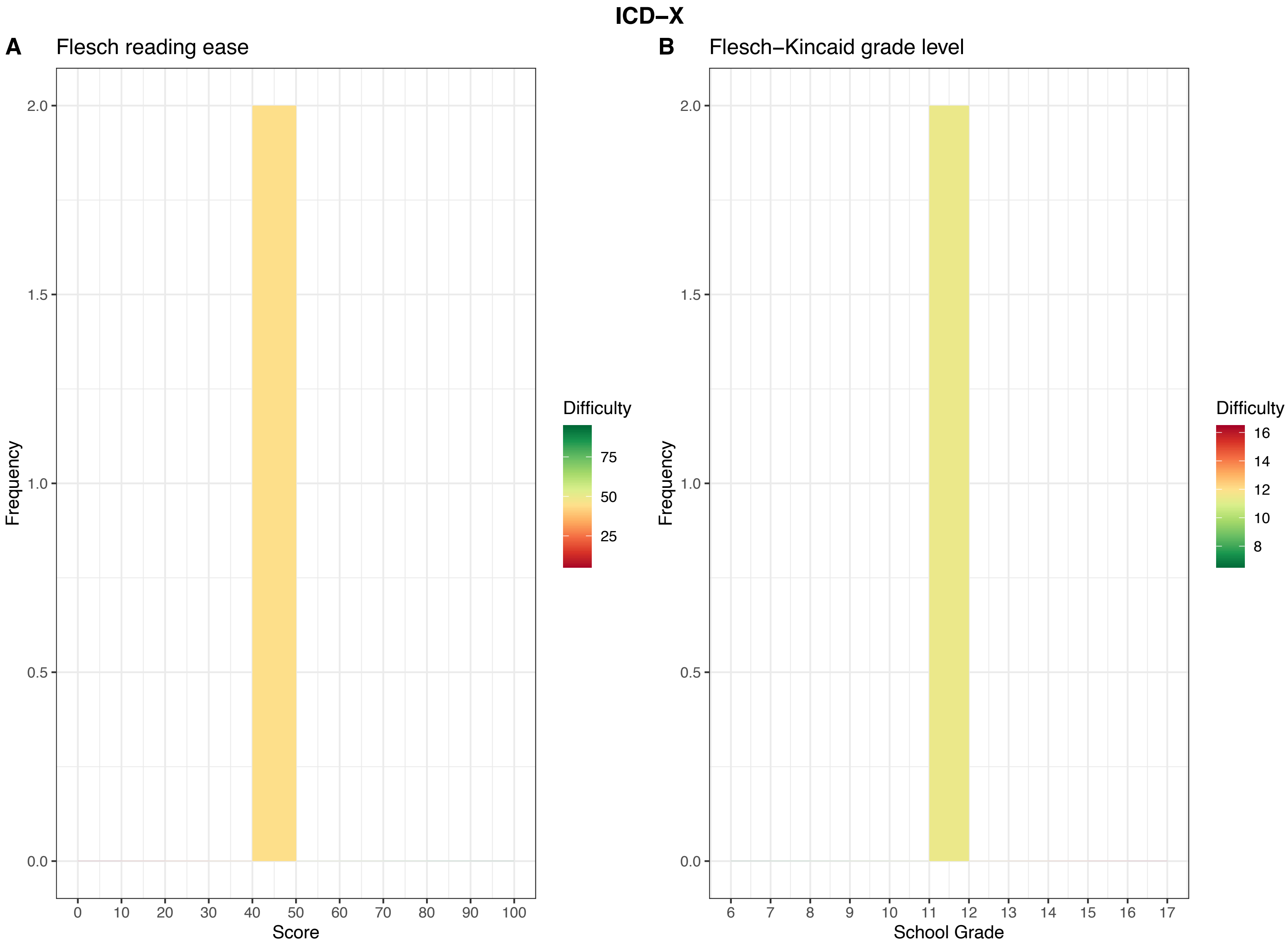

ICD−X
A
Flesch reading ease
Frequency
2.0
1.5
1.0
0.5
0.0
0
10
20
30
40
50
60
70
80
90
100
Score
Difficulty
75
50
25
B
Flesch−Kincaid grade level
Frequency
2.0
1.5
1.0
0.5
0.0
6
7
8
9
10
11
12
13
14
15
16
17
School Grade
Difficulty
16
14
12
10
8

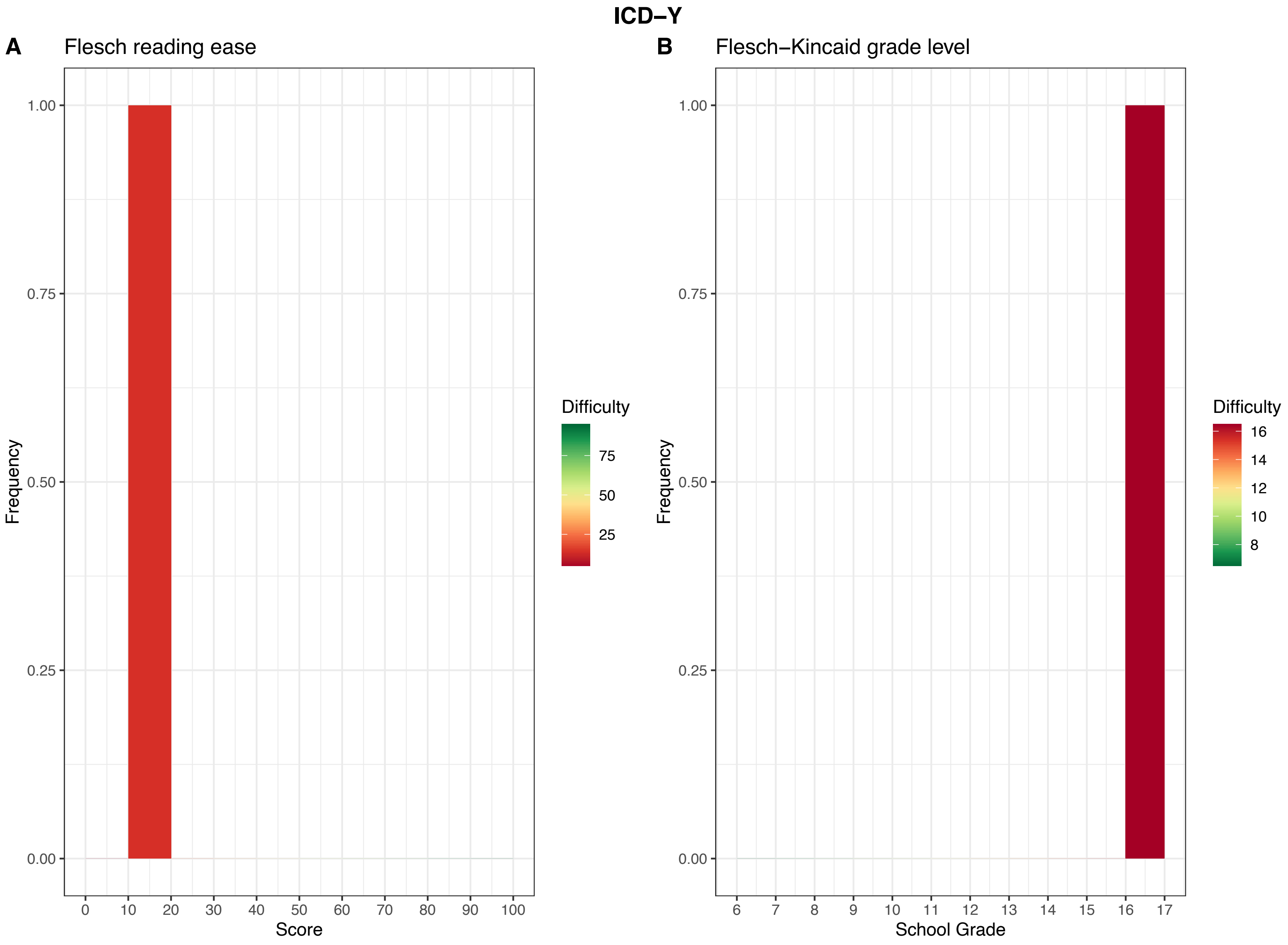

ICD–Y
A
Flesch reading ease
Frequency
1.00
0.75
0.50
0.25
0.00
0
10
20
30
40
50
60
70
80
90
100
Score
Difficulty
75
50
25
B
Flesch–Kincaid grade level
Frequency
1.00
0.75
0.50
0.25
0.00
6
7
8
9
10
11
12
13
14
15
16
17
School Grade
Difficulty
16
14
12
10
8

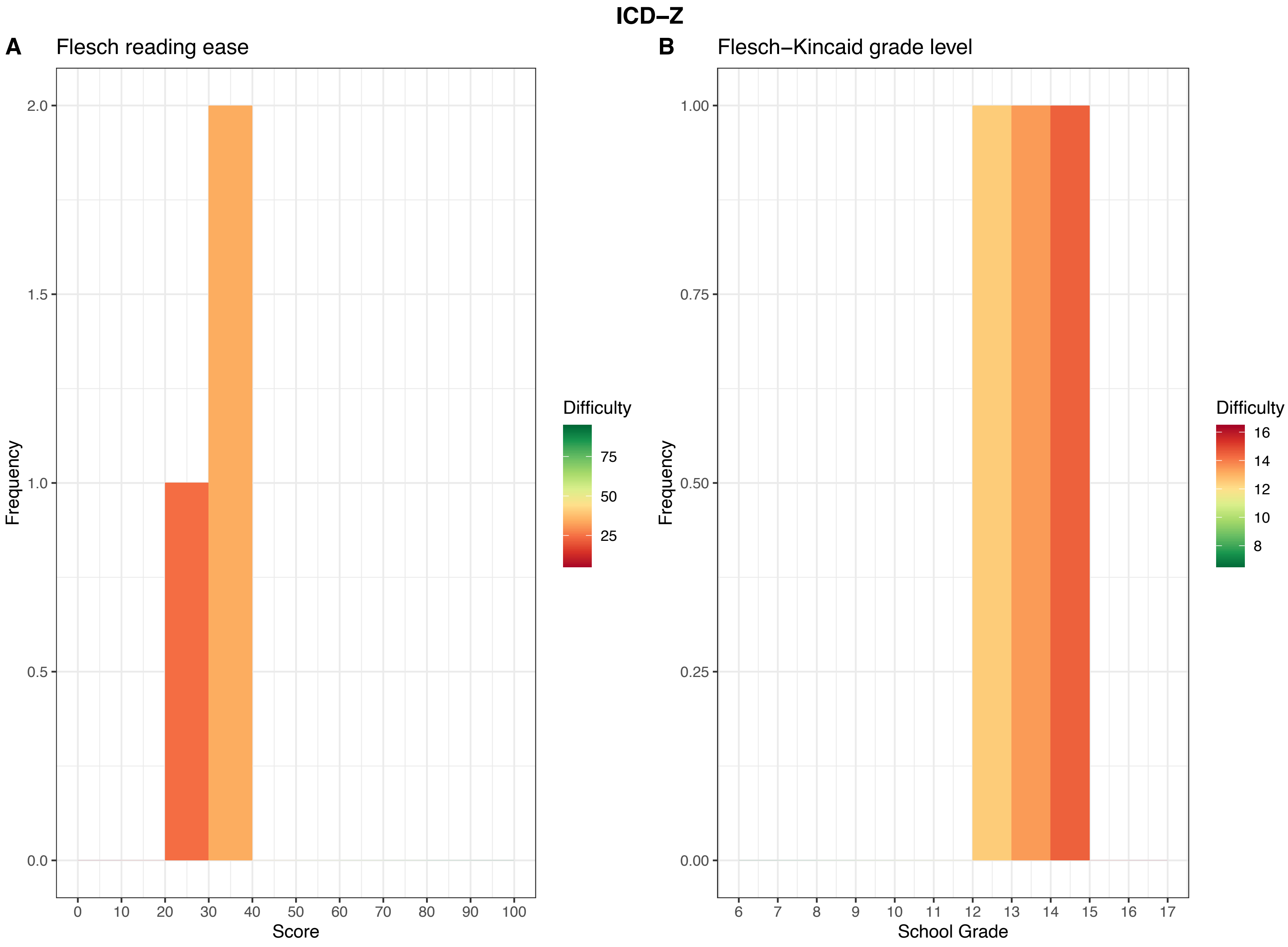

ICD–Z
A
Flesch reading ease
Frequency
2.0
1.5
1.0
0.5
0.0
0
10
20
30
40
50
60
70
80
90
100
Score
Difficulty
75
50
25
B
Flesch–Kincaid grade level
Frequency
1.00
0.75
0.50
0.25
0.00
6
7
8
9
10
11
12
13
14
15
16
17
School Grade
Difficulty
16
14
12
10
8
