# Supplementary material for: Readability of English, German, and Russian Disease-Related Wikipedia Pages: Automated Computational Analysis
Source: J Med Internet Res. 2022 May 16;24(5):e36835. doi: 10.2196/36835 (PMC9152717; doi:10.2196/36835)
Supplement: Multimedia Appendix 7 [file jmir_v24i5e36835_app7.pdf]

Multimedia Appendix 7: Boxplots with readability values for English sample

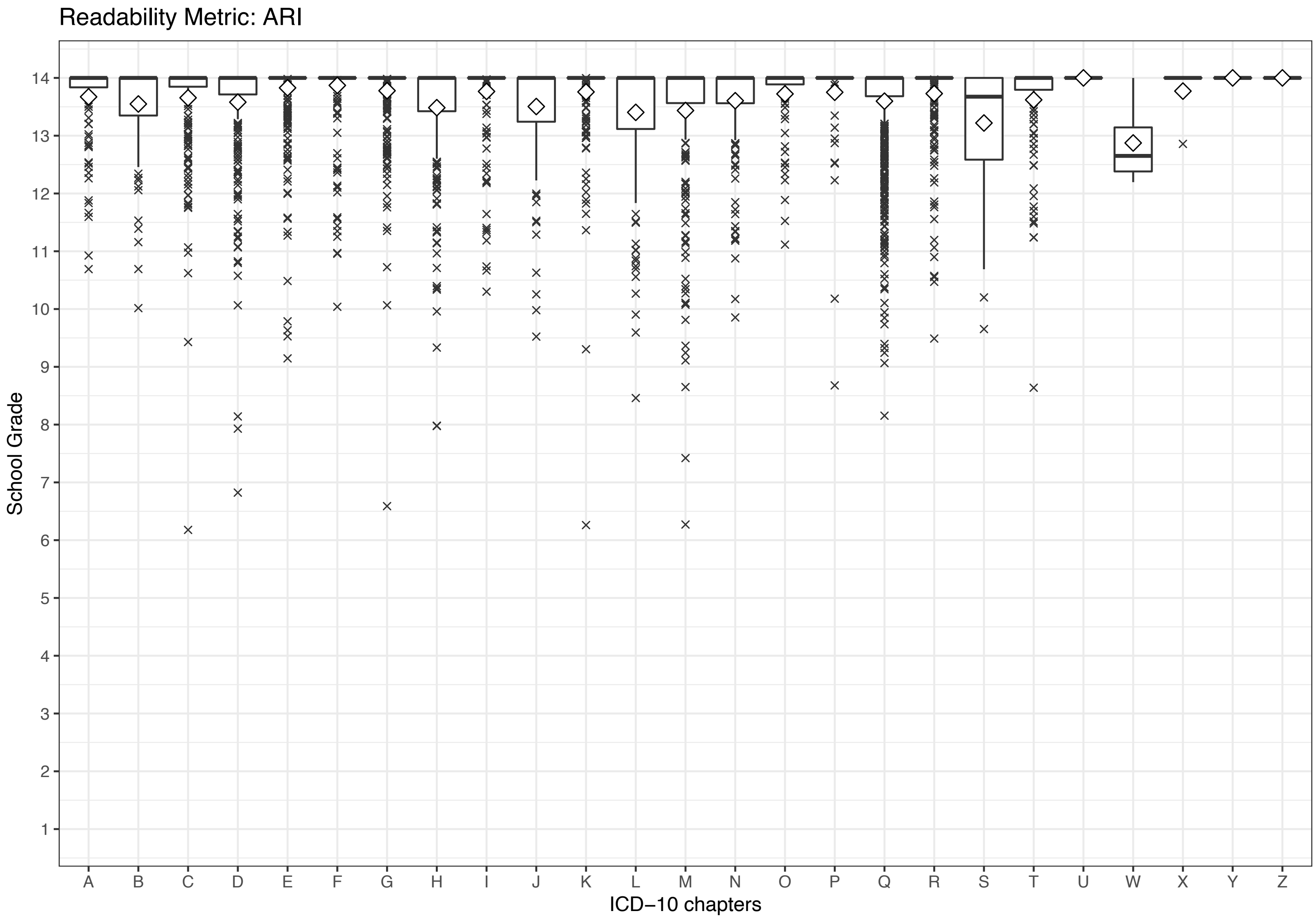

Readability Metric: ARI
School Grade
ICD−10 chapters
14
13
12
11
10
9
8
7
6
5
4
3
2
1
A B C D E F G H I J K L M N O P Q R S T U W X Y Z

Readability Metric: Coleman−Liau

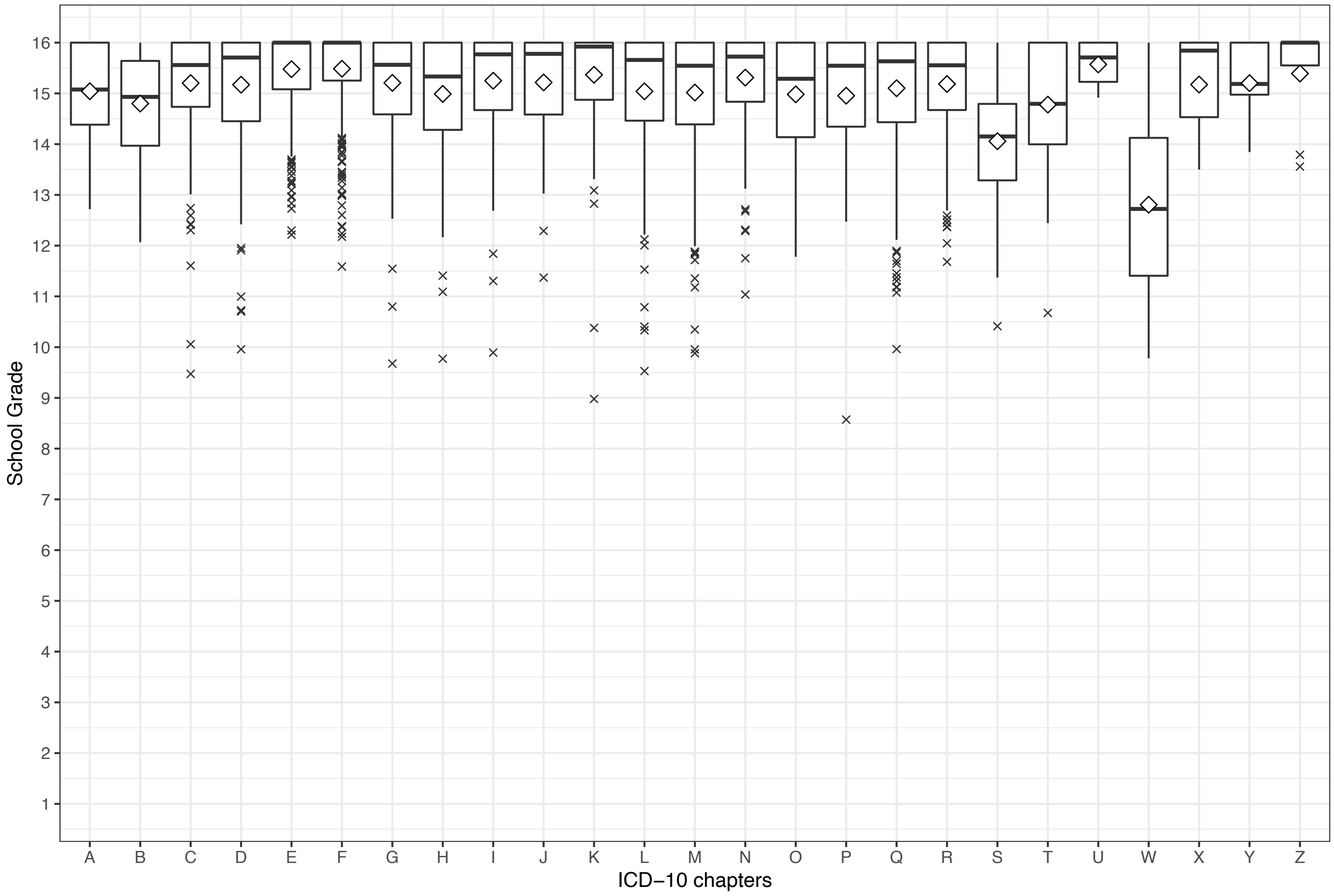

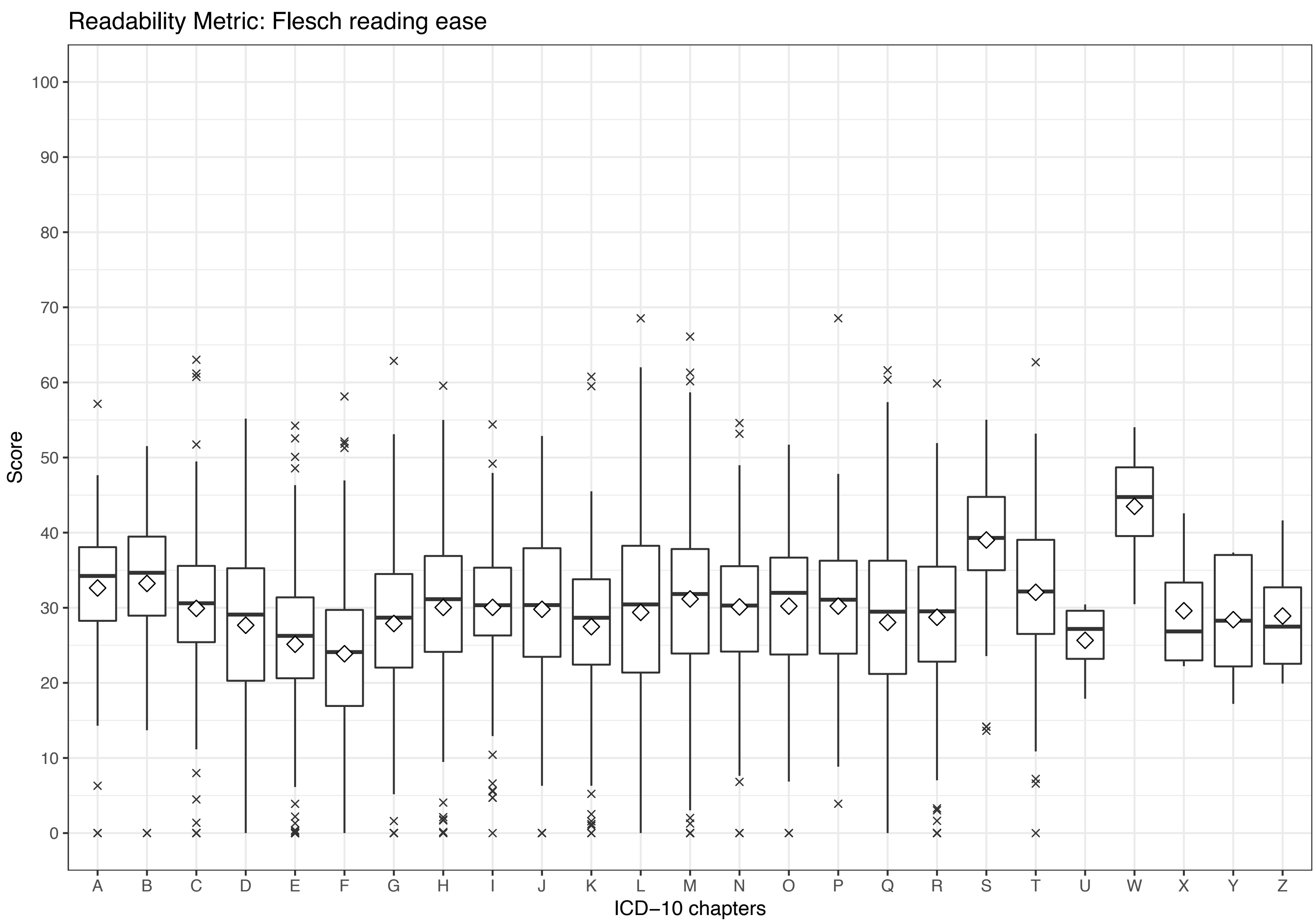
Readability Metric: Flesch reading ease
Score
0
10
20
30
40
50
60
70
80
90
100
A
B
C
D
E
F
G
H
I
J
K
L
M
N
O
P
Q
R
S
T
U
W
X
Y
Z
ICD−10 chapters

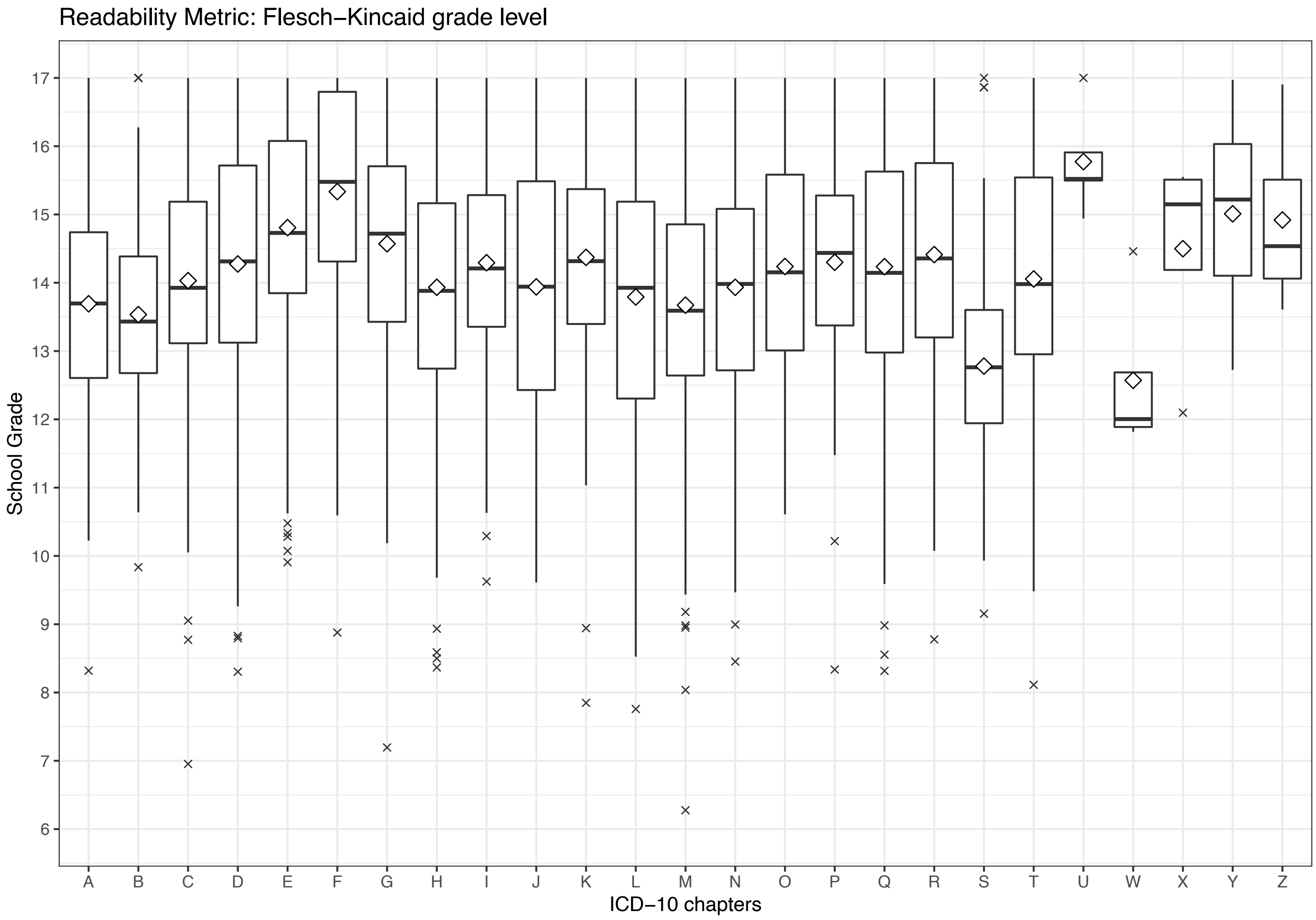
Readability Metric: Flesch−Kincaid grade level
School Grade
6
7
8
9
10
11
12
13
14
15
16
17
A B C D E F G H I J K L M N O P Q R S T U W X Y Z
ICD−10 chapters

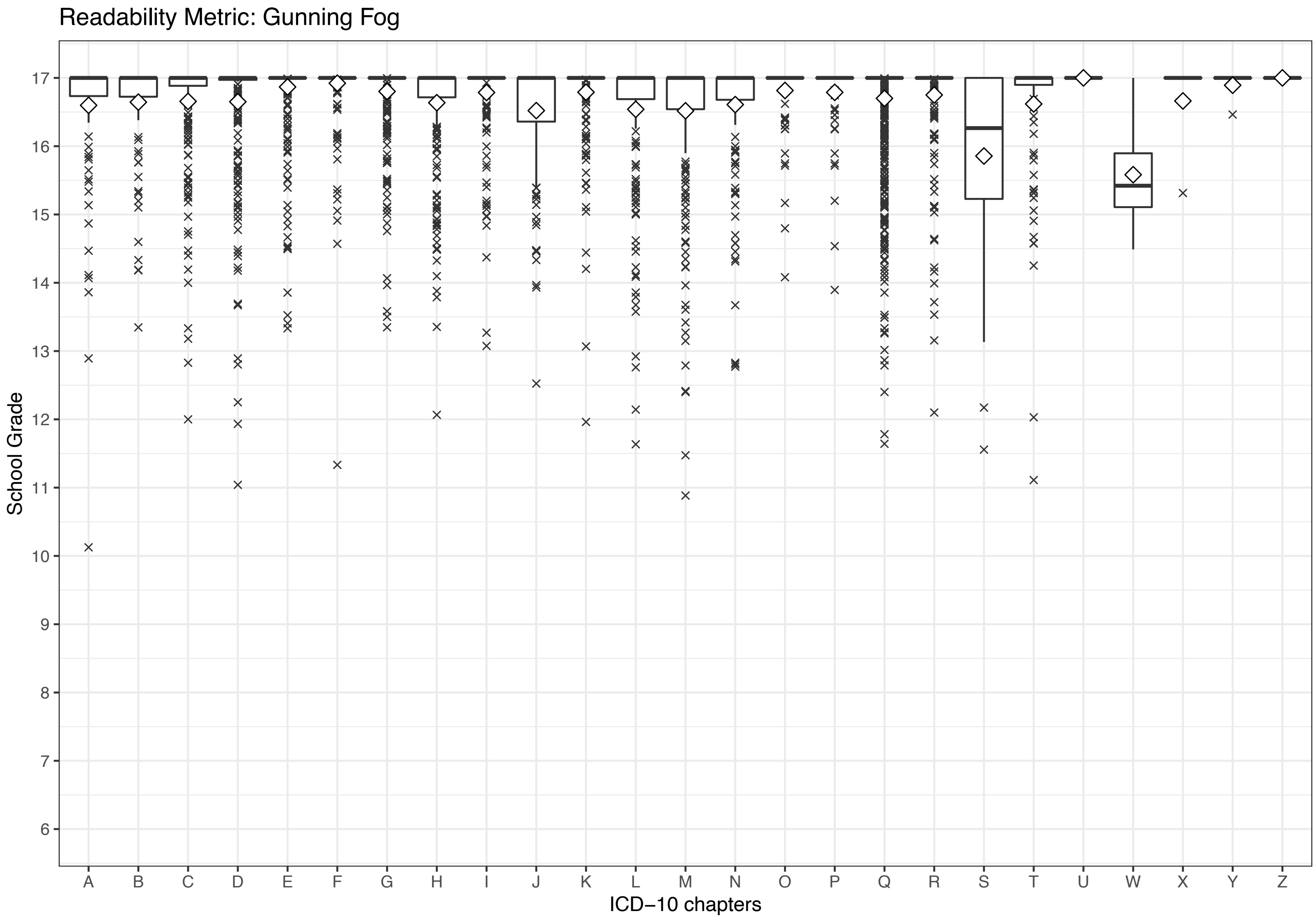
Readability Metric: Gunning Fog
School Grade
ICD−10 chapters
A B C D E F G H I J K L M N O P Q R S T U W X Y Z
6 7 8 9 10 11 12 13 14 15 16 17

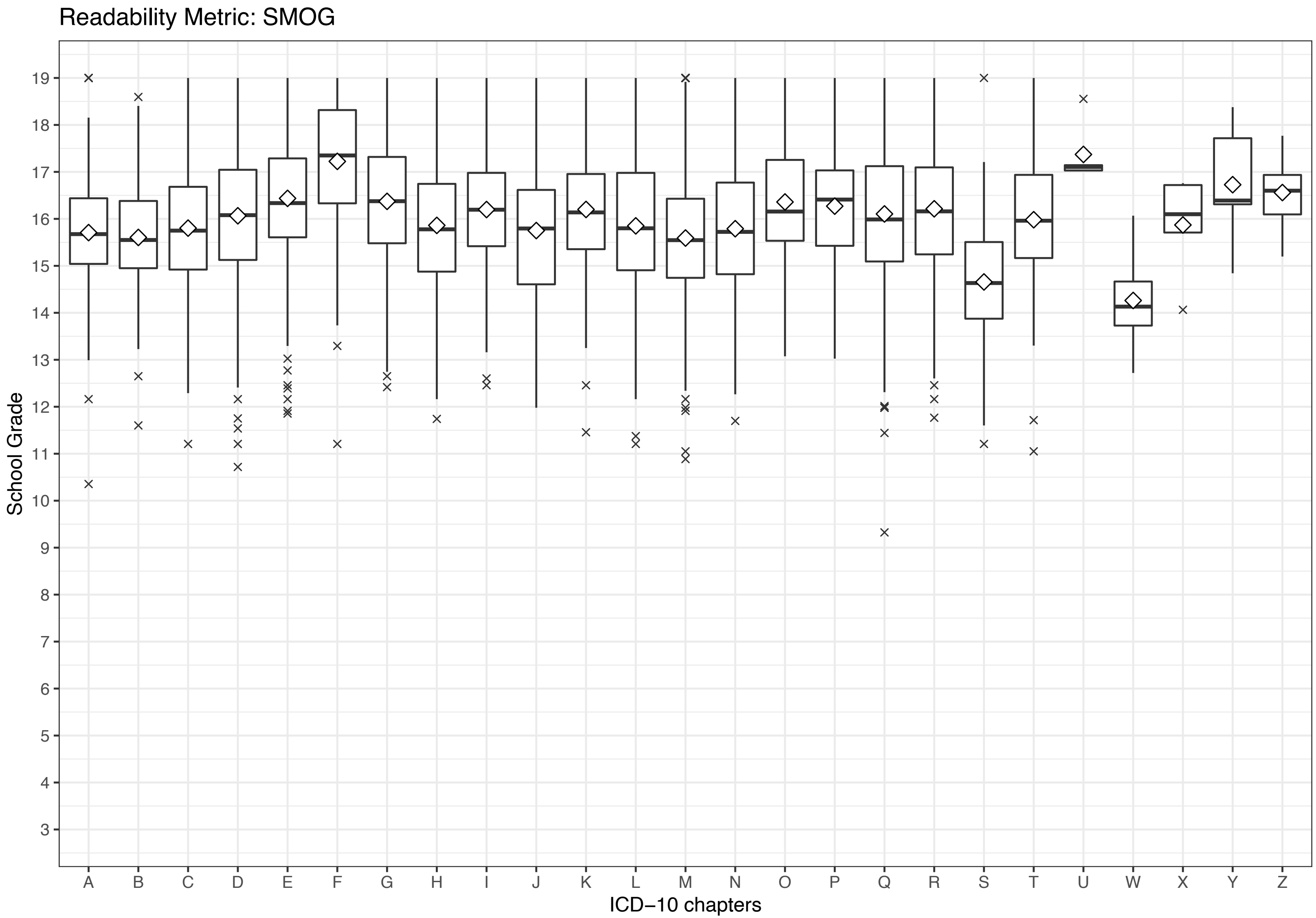
Readability Metric: SMOG
School Grade
ICD−10 chapters
A B C D E F G H I J K L M N O P Q R S T U W X Y Z
19 18 17 16 15 14 13 12 11 10 9 8 7 6 5 4 3

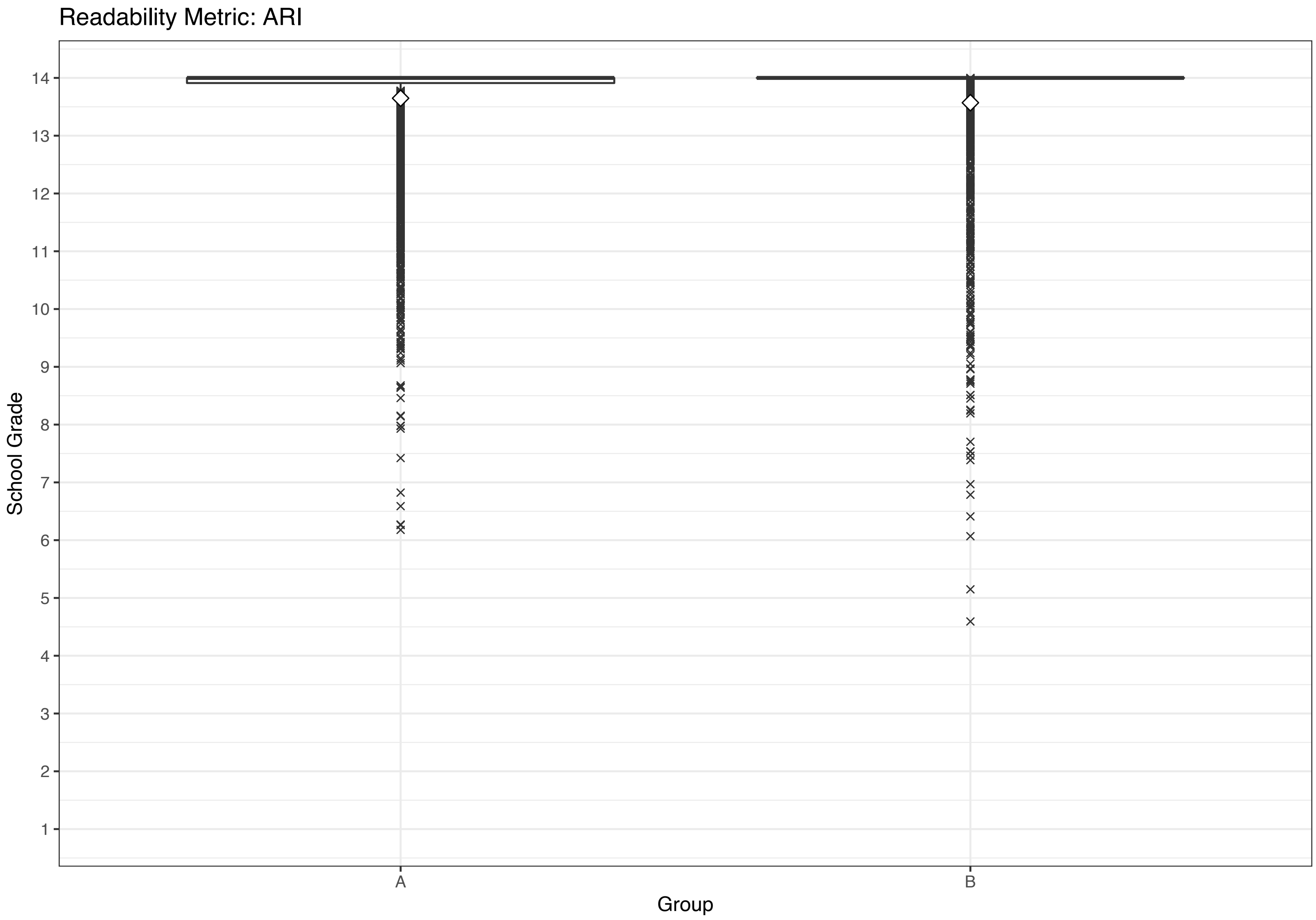
Readability Metric: ARI
School Grade
14
13
12
11
10
9
8
7
6
5
4
3
2
1
A
B
Group

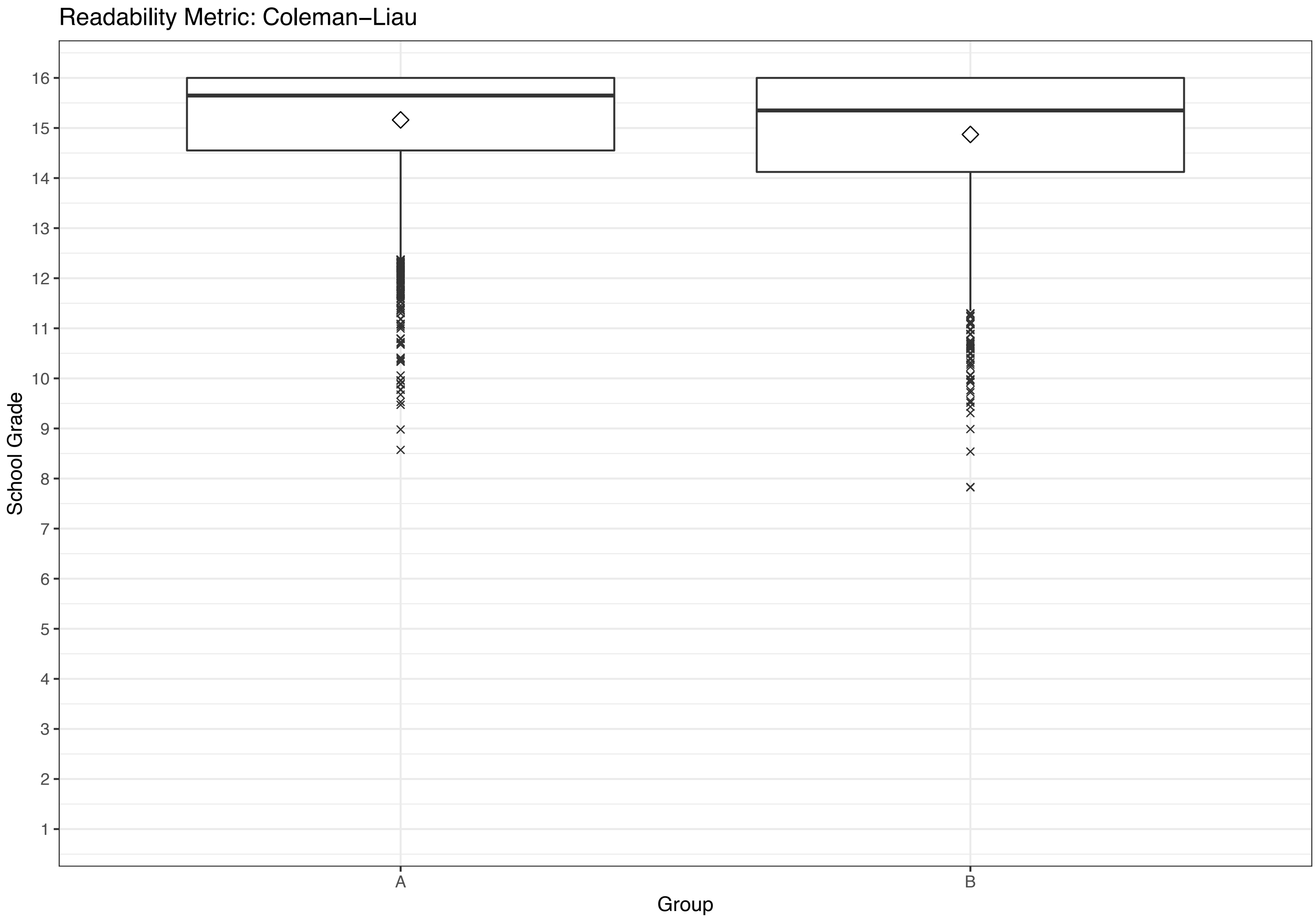
Readability Metric: Coleman−Liau
School Grade
16
15
14
13
12
11
10
9
8
7
6
5
4
3
2
1
A
B
Group

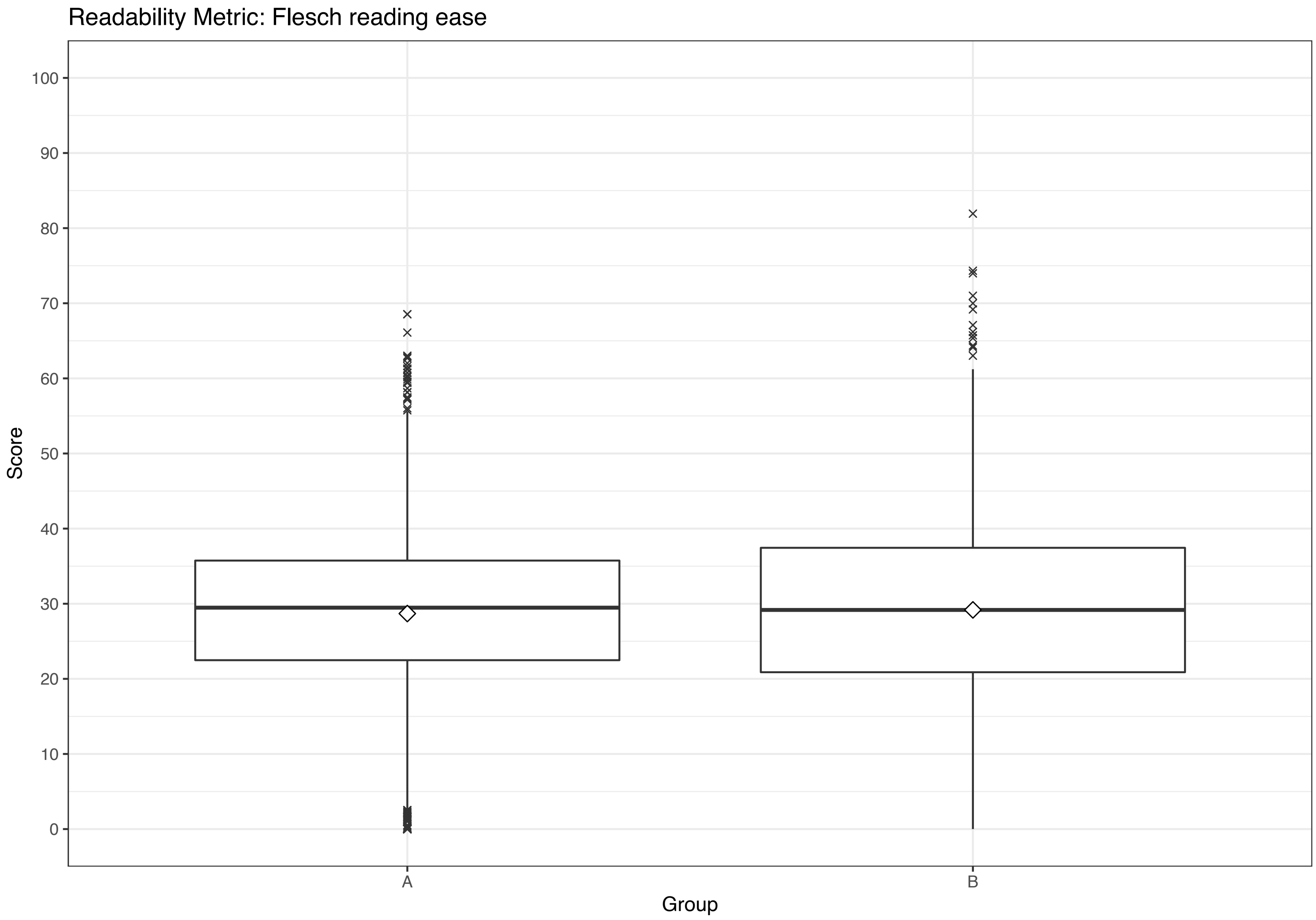
Readability Metric: Flesch reading ease
Score
100
90
80
70
60
50
40
30
20
10
0
A
B
Group

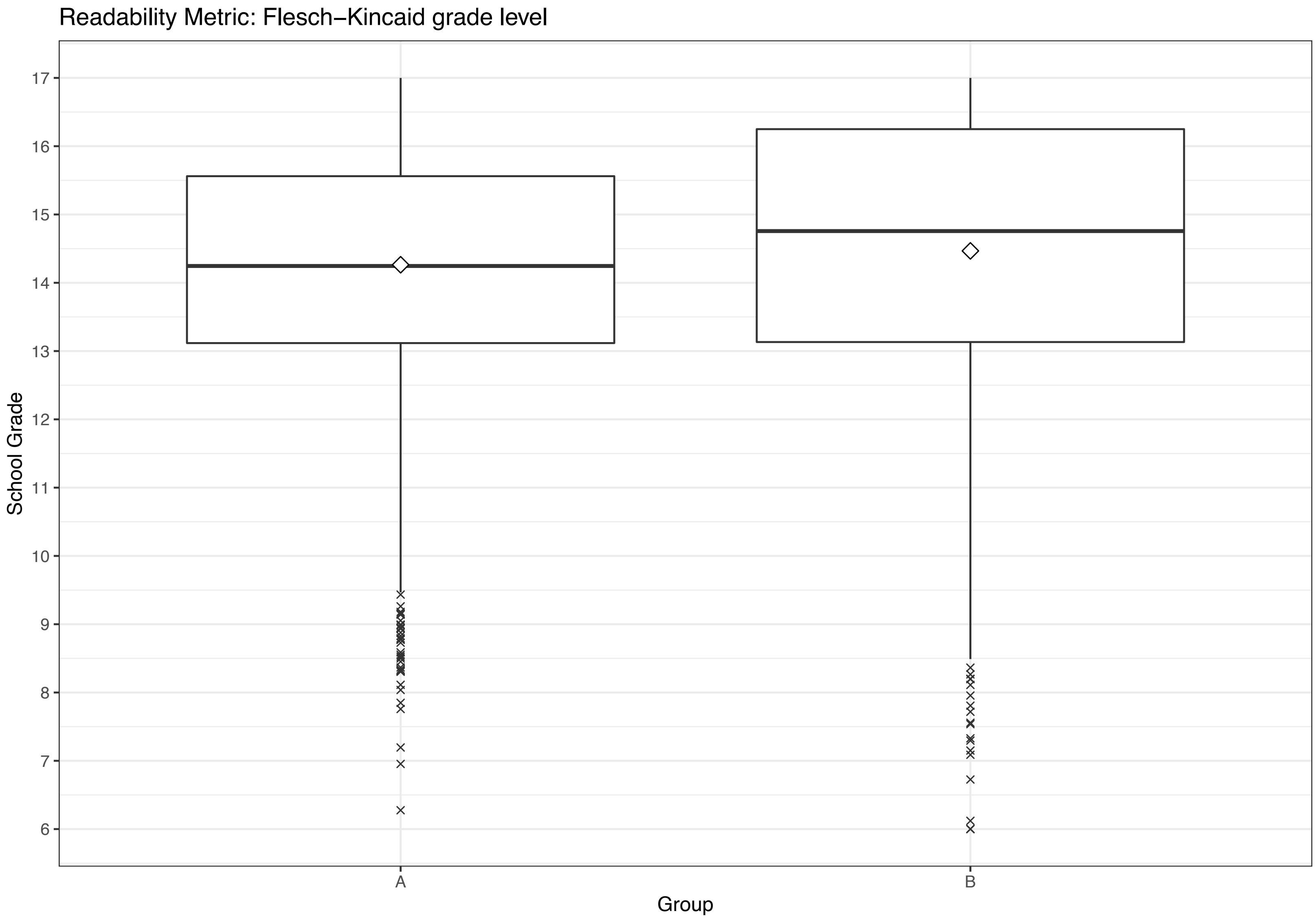

Readability Metric: Flesch−Kincaid grade level
School Grade
17
16
15
14
13
12
11
10
9
8
7
6
A
B
Group

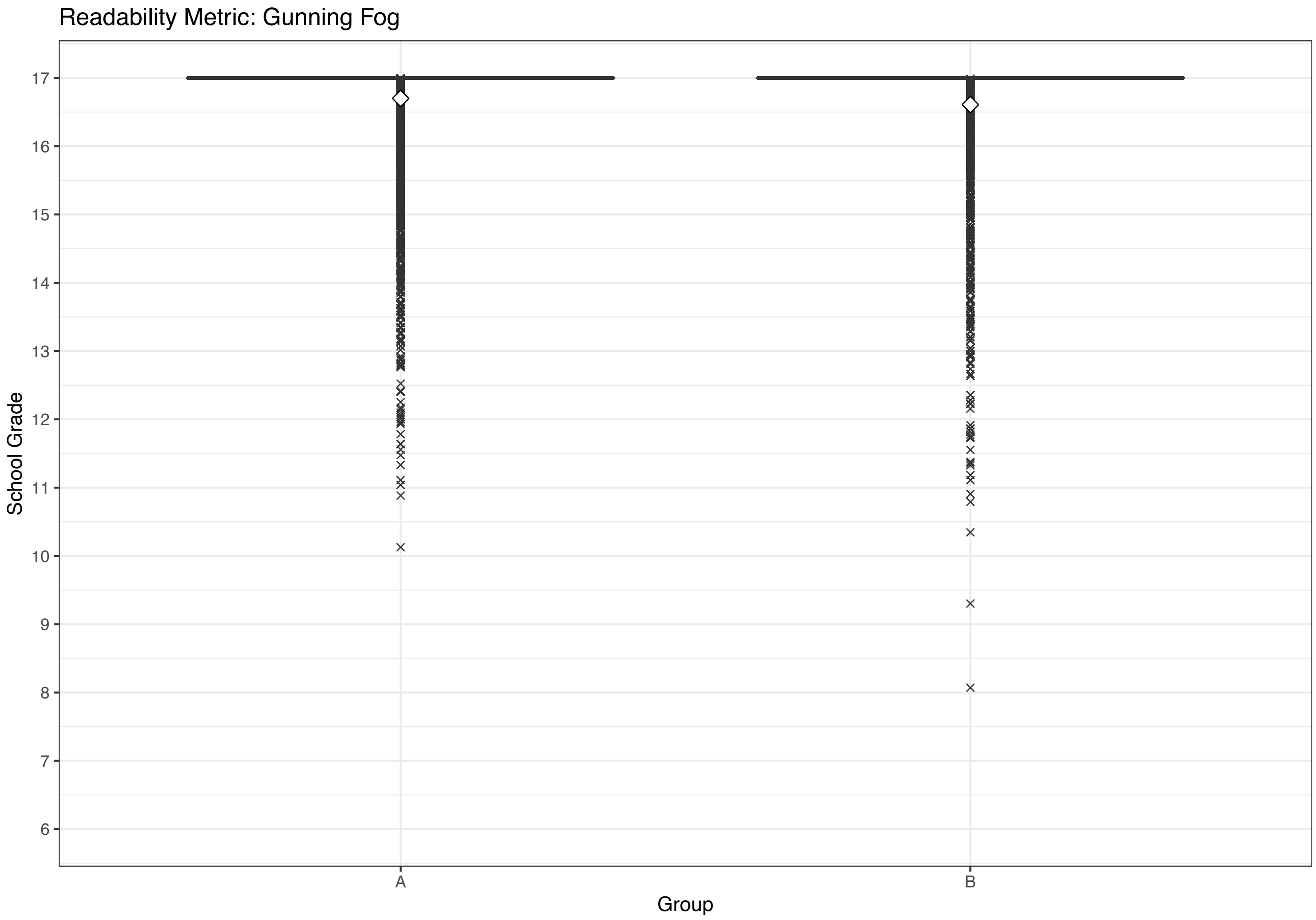
Readability Metric: Gunning Fog
School Grade
17
16
15
14
13
12
11
10
9
8
7
6
A
B
Group

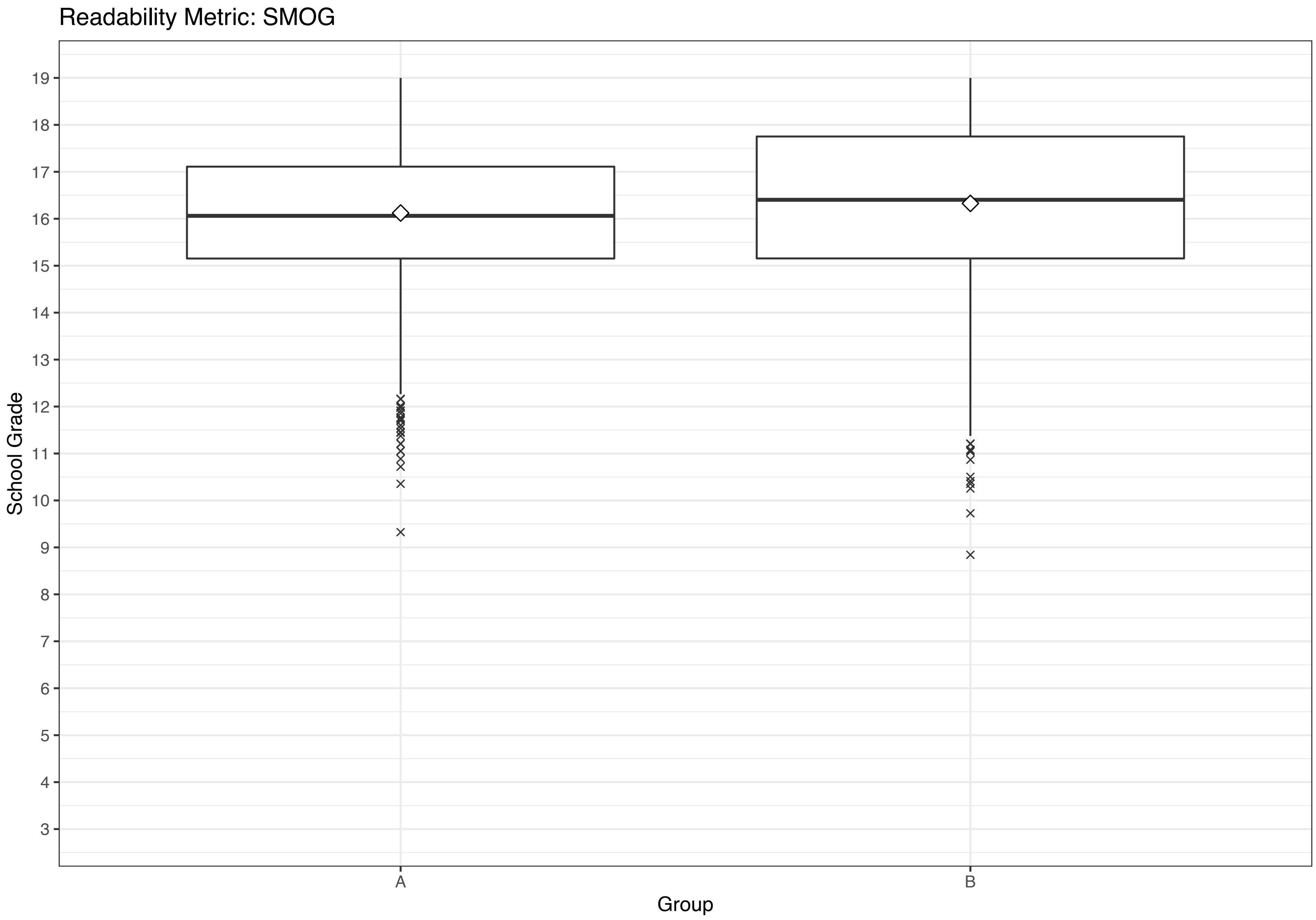
Readability Metric: SMOG
School Grade
19
18
17
16
15
14
13
12
11
10
9
8
7
6
5
4
3
A
B
Group
