# Supplementary material for: Readability of English, German, and Russian Disease-Related Wikipedia Pages: Automated Computational Analysis
Source: J Med Internet Res. 2022 May 16;24(5):e36835. doi: 10.2196/36835 (PMC9152717; doi:10.2196/36835)
Supplement: Multimedia Appendix 8 [file jmir_v24i5e36835_app8.pdf]

Multimedia Appendix 8: Boxplots with readability values for German sample

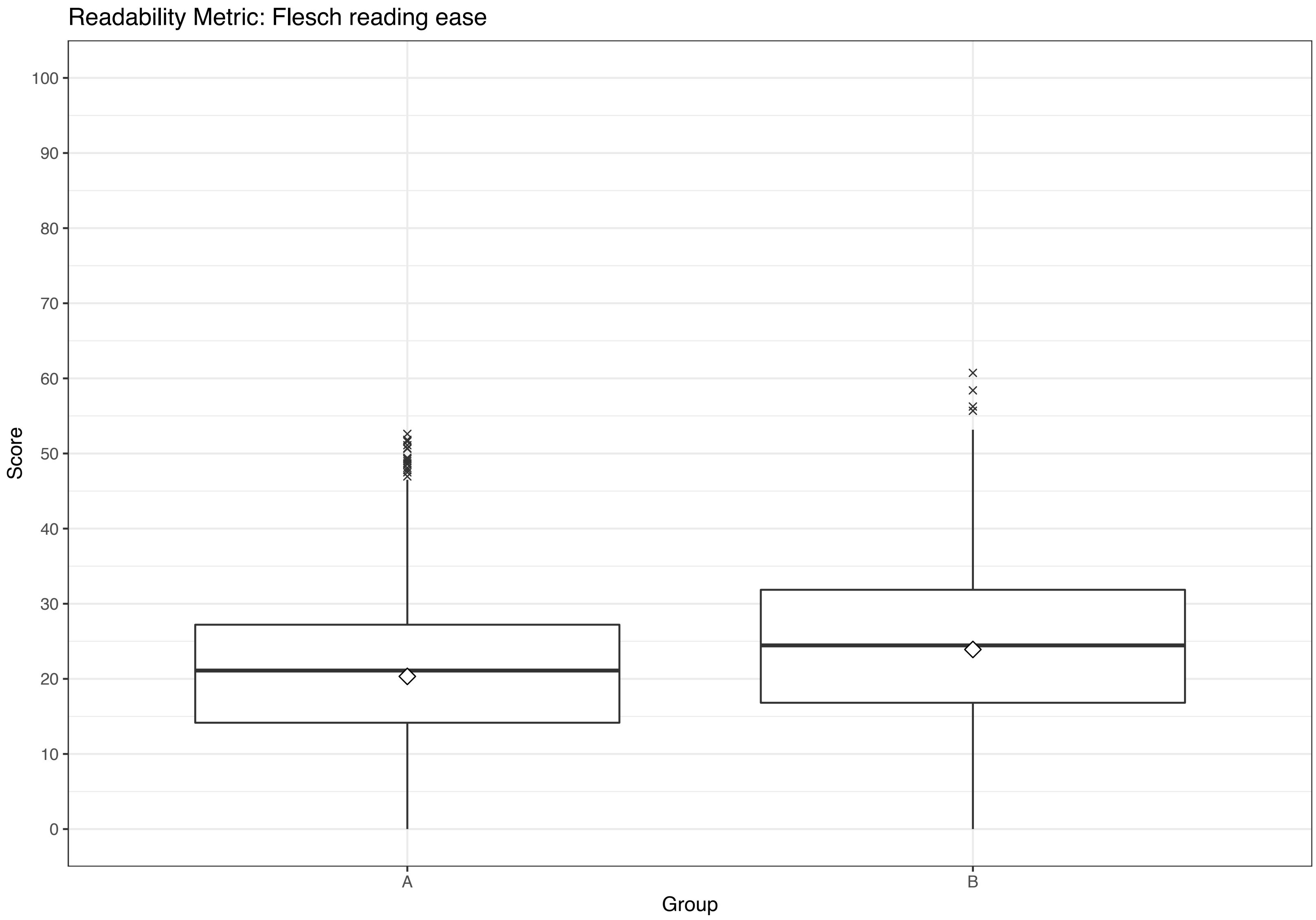
Readability Metric: Flesch reading ease
Score
100
90
80
70
60
50
40
30
20
10
0
A
B
Group

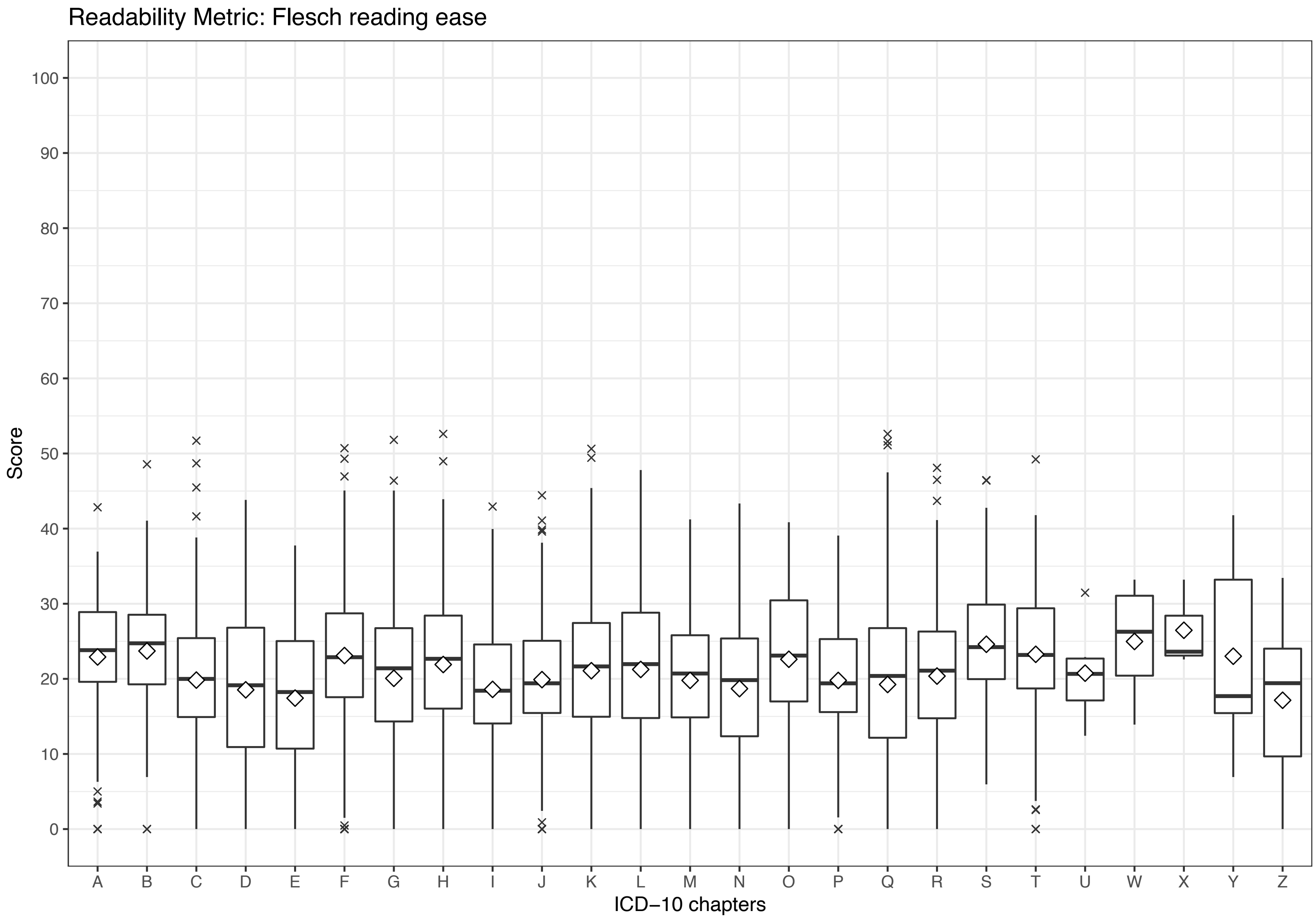
Readability Metric: Flesch reading ease
Score
0
10
20
30
40
50
60
70
80
90
100
A B C D E F G H I J K L M N O P Q R S T U W X Y Z
ICD−10 chapters

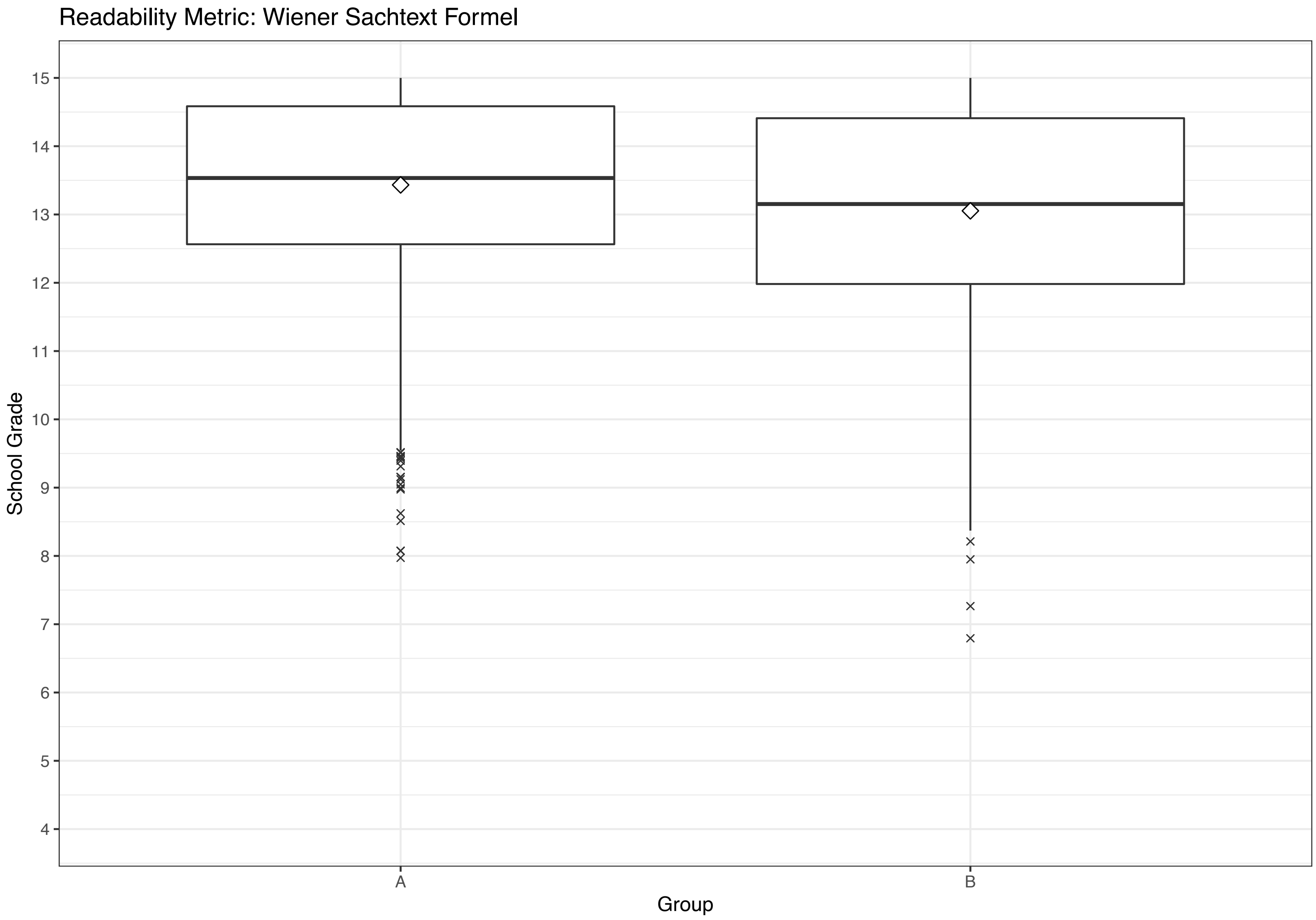
Readability Metric: Wiener Sachtext Formel
School Grade
15
14
13
12
11
10
9
8
7
6
5
4
A
B
Group

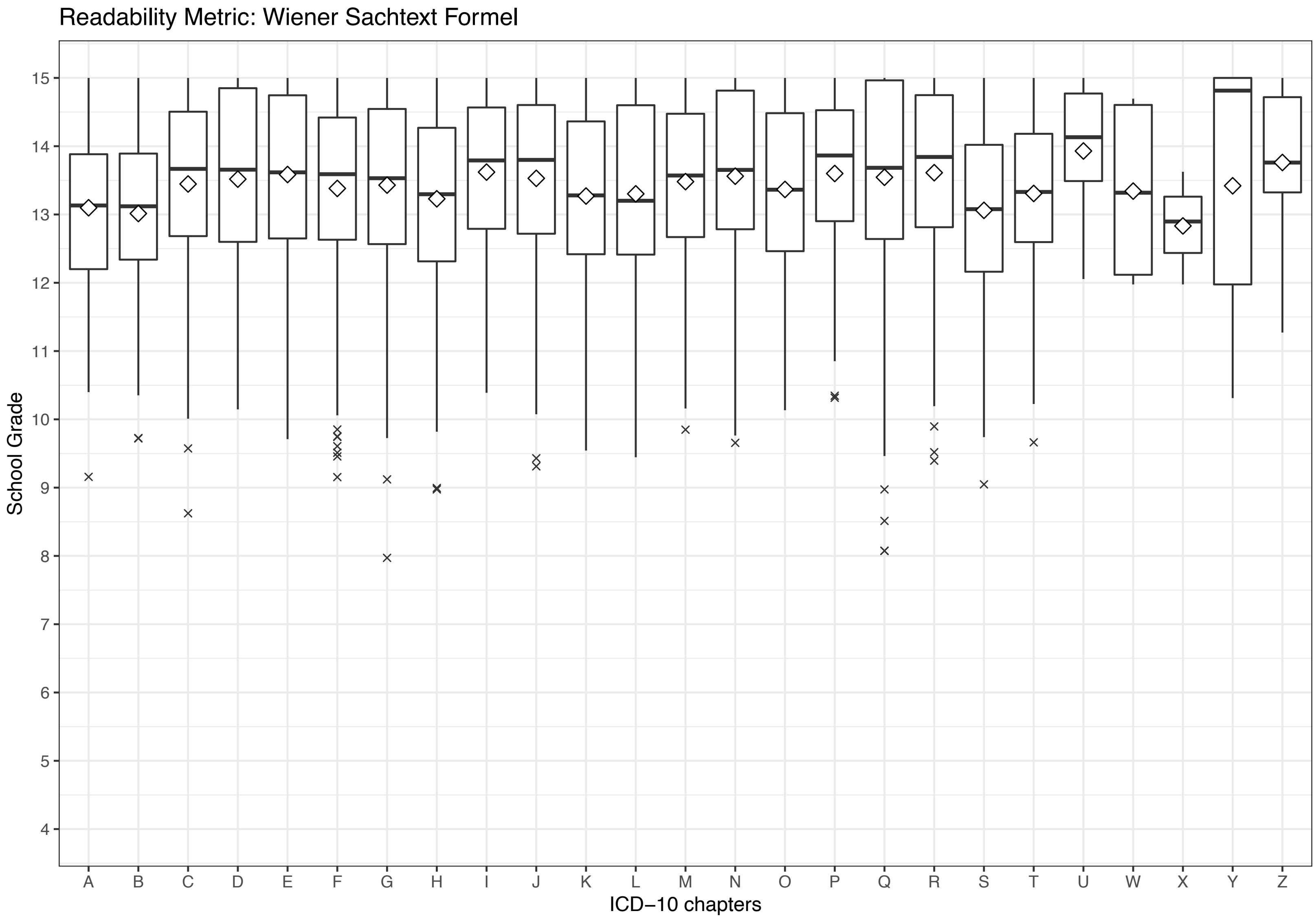
Readability Metric: Wiener Sachtext Formel
School Grade
ICD−10 chapters
4
5
6
7
8
9
10
11
12
13
14
15
A B C D E F G H I J K L M N O P Q R S T U W X Y Z
